# A Comparative Study of ^157^Gd and ^10^B Effect in a Carborane-Based Theranostic Agent for Membrane-Targeted Carbonic Anhydrase IX Inhibition and MRI-Guided Neutron Capture Therapy in Mesothelioma Treatment

**DOI:** 10.1021/acscentsci.5c01632

**Published:** 2025-10-01

**Authors:** Sahar Rakhshan, Alberto Lanfranco, Diego Alberti, Polyssena Renzi, Ayda Zarechian, Sabrina Elkhanoufi, Juan Carlos Cutrin, Nicoletta Protti, Simonetta Geninatti Crich, Annamaria Deagostino

**Affiliations:** † Department of Molecular Biotechnology and Health Sciences; 9314 University of Torino, via Nizza 52, 10126, Torino, Italy; ‡ Department of Chemistry, University of Torino, via P. Giuria 7, 10125, Torino, Italy; § Department of Physics, 19001 University of Pavia, via Agostino Bassi 6, Pavia 27100, Italy; ∥ Nuclear Physics National Institute (INFN), Unit of Pavia, via Agostino Bassi 6, Pavia 27100, Italy

## Abstract

This study investigates combined gadolinium and boron neutron capture therapy (GdBNCT) with carbonic anhydrase IX (CA IX) inhibition for mesothelioma treatment using a multifunctional platform (Gd-B-CA-SF). This platform incorporates a carborane cage for BNCT, a Gd complex for GdNCT/MRI, and a sulfamido group for CA IX inhibition. In vitro studies confirmed CA IX inhibition and selective binding of the compound to AB22 mesothelioma cells, with a 10-fold higher boron uptake compared to that in healthy Met-5a mesothelium. The combination of B and Gd in one molecule allows the exploration of potential additional effects coming from the nuclides ^10^B and ^157^Gd. Irradiation of AB22 cells showed complete tumor regrowth inhibition after treatment with ^157^Gd-^10^B-CA-SF, an effect strictly related to the presence of ^157^Gd and its localization on the cytosolic membrane. In vivo MRI studies confirmed higher accumulation of Gd-B-CA-SF in AB22 tumors in mice, demonstrating effective targeting. In vivo NCT experiments showed reduced tumor growth in ^157^Gd-^10^B-CA-SF treated mice, confirming the effectiveness of both ^157^Gd and ^10^B, even at relatively low concentrations. These results demonstrate the translational potential of Gd-B-CA-SF as a theranostic agent for an innovative approach to cancer treatment.

## Graphical abstract

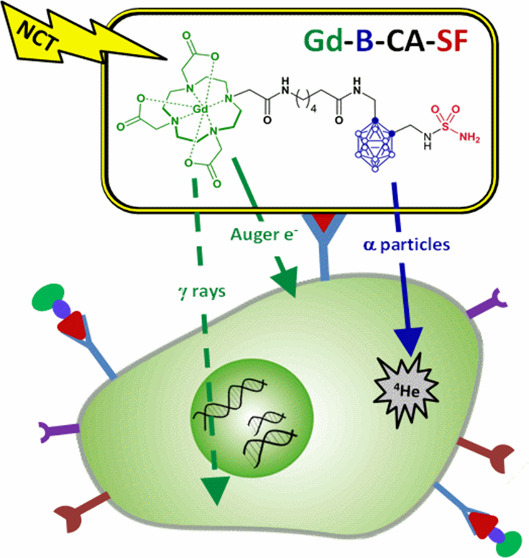

## Introduction

Alpha and Auger electron therapies^1,2^ are widely utilized as targeted radiation therapy for the treatment of cancer. These therapies employ high-energy α-particles (4He) and low-energy Auger electrons, respectively, resulting in high linear energy transfer (LET) due to the rapid deposition of energy in tissues. α-Particles have been shown to be compatible with the diameter of a mammalian cell, whereas Auger electrons with a shorter range of 1–500 nm can be beneficial, especially when the source is localized near the target DNA or on the cell membrane. Only selective targeting of the particles to tumor cells, without damaging the surrounding healthy tissue^3,4^ ensures safe treatment, avoiding the energy release widely across different organs.

Neutron Capture Therapy (NCT) is an alternative binary radiotherapy based on the capture reaction of thermal neutrons by various nuclides, mainly employed in the treatment of malignant tumors with the purpose of destroying tumor cells without compromising nearby or contiguous normal tissues.^5,6^ The main advantage of NCT over the targeted particle radiotherapies relies on the administration of nonradioactive nuclides activated on demand, only by neutron exposure. This ensures high specificity, particularly when combined with a noninvasive imaging method that can monitor the biodistribution of the agent. Two isotopes, ^10^B and ^157^Gd, are of major interest due to their significantly large NC cross sections. Boron NCT (BNCT) is without any doubt the most developed, thanks to the intrinsic properties of ^10^B. This isotope is, in fact, nonradioactive and nontoxic and upon NC undergoes fission generating α-particles and ^7^Li. Furthermore, a significant number of boron derivatives exhibit high chemical stability and versatility, offering the possibility of tuning their biological properties by proper design.^7–11^

Currently, two boron derivatives, namely boronophenylalanine (BPA) and mercaptoundecahydrododecaborate (BSH), are used in the clinic for the treatment of glioblastoma, malignant melanoma, and head and neck cancer.^12–20^ Unfortunately, some problems are still associated with BPA, including its low solubility in water, and BSH, which result in a low selectivity. Besides BPA and BSH, polynuclear boron derivatives have been considered as potential candidates for BNCT,^21^ in particular carboranes.^22^ Those boron clusters are characterized by a high boron content, chemical versatility, and *in vivo* stability,^23,24^ allowing them to be exploited not only as BNCT agents^11,25^ but also as tridimensional isosteres of aromatic rings in bioactive scaffolds^26–32^ and as smart materials.^33–37^

^157^Gd is a stable nuclide with the highest NC cross section among those used for NCT. The absorption of neutrons by ^157^Gd results in the emission of γ-rays and Auger electrons. The subsequent production of γ-rays can be considered not advantageous due to their longer action range with consequent lower selectivity;^38^ however, it can be beneficial when ^157^Gd is located outside of the nucleus. Moreover, Gd allows enhancement of the image contrast in magnetic resonance imaging (MRI), thus offering the possibility of tracing the *in vivo* biodistribution and properly designing the neutron irradiation therapy. Despite several Gd-containing agents being investigated for GdNCT, they proved to be inadequate for use in clinics due to their low accumulation in cancer cells and rapid excretion.^39,40^

To the best of our knowledge, although a synergistic combination of ^10^B and ^157^Gd NCT has been already hypothesized as an efficient strategy in cancer treatment, few examples of drug design focused on molecules containing both B and Gd^41,42^ have been reported, and most of them are oriented to MRI guided NCT applications.^43–52^ A few preliminary studies investigated the combination of BNCT and GdNCT employing systems containing ^10^B and ^157^Gd in two separate molecules.^41,42,53,54^ Combining BSH and some MRI contrast agents, Matsumura et al. observed an additive toxicity at low Gd concentrations, whereas higher levels of Gd quenched BNC.^54^ Similar effects have been observed *in vivo* by Lee and co-workers exploiting liposomes loaded with ^10^BSH, ^10^BPA, or a commercially available MRI contrast agent (Gd-DO3A-butrol).^41^ Recent advances show promising tumor eradication using GdNCT with inorganic nanoparticles; however, these nanoparticles may release toxic free metal, a risk that can be avoided by binding Gd to more stable chelating agents.^55^

The present study proposes a novel Gd-DOTA-sulfamidocarborane (Gd-B-CA-SF) derivative that incorporates both ^10^B and ^157^Gd within a single molecular structure with the aim of combining BNCT and GdNCT with the inhibition of the enzyme carbonic anhydrase IX (CA IX). This is the first example of a multivalent agent for MRI-guided treatment of mesothelioma (Scheme 1), which follows our recent studies on the use of BNCT associated with the inhibition of CA.^56–60^ A multidisciplinary approach involving surgical resection, chemotherapy, and radiation therapy is now considered the most efficient strategy for the treatment of asbestos related diseases,^58–61^ taking into account that a single therapeutic agent often results in limited clinical outcomes due to tumor heterogeneity and drug resistance.^62–64^ CAs are widespread zinc enzymes playing a central role in both transport and metabolic processes. In particular, for solid tumors, they have a fundamental role in maintaining the acidity of the tumor environment.^65,66^ Specifically, two isozymes, CA IX and CA II,^67^ have been prominently associated with cancer.^68–71^ Currently, many sulfonamide-based drugs are clinically used for the treatment of different pathological conditions,^72–76^ because these drugs are bable to inhibit CAs thanks to their ability to displace ligands at the zinc catalytic site. Therefore, sulfonamido (CA-SF) and arylureidosulfamido functionalized carboranes have been already proposed as both CA IX inhibitors^77–79^ and boron delivery agents.^80,81^ Despite the positive results achieved in inhibiting mesothelioma cell proliferation, the low solubility in water and the inability to monitor their biodistribution prior to treatment are the main limitations for the clinical translation of these compounds. Therefore, the aim of this study is to evaluate the effect of the simultaneous generation of α-particles, Auger electrons, and γ-rays, arising from a unique platform containing a carborane linked to a sulfamido group and a Gd-complex, Gd-B-CA-SF. All the natural abundance and enriched isotopic derivatives of Gd-B-CA-SF were prepared and tested in order to identify any additional or synergistic effects related to NC from the nuclides ^10^B and ^157^Gd.

**Scheme 1:**
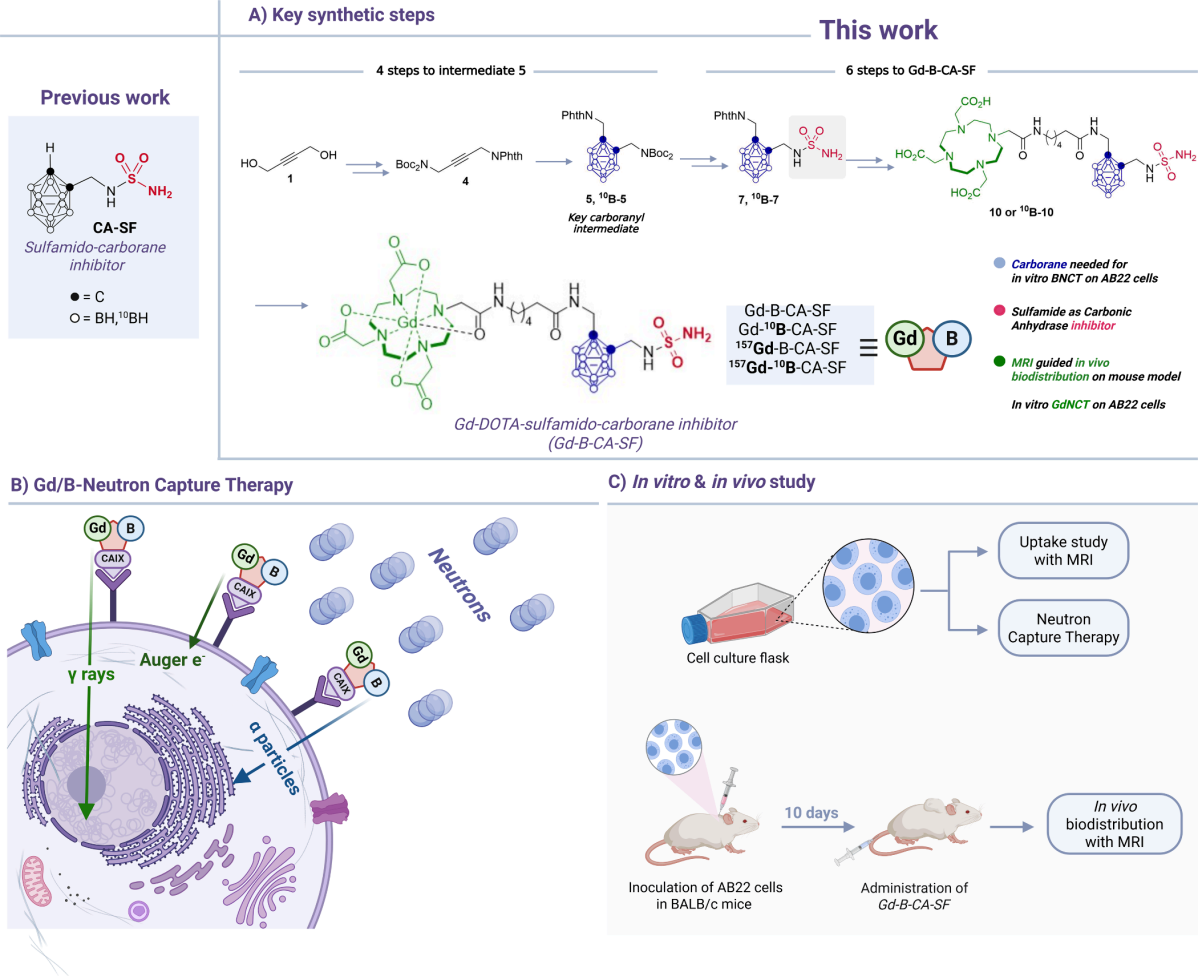
Application of a Multifunctional Platform (Gadolinium-DOTA-sulfamido-carborane, Gd-B-CA-SF) Combining Gadolinium and Boron Neutron Capture Therapy (GdBNCT) and Carbonic Anhydrase IX (CA IX) Inhibition for MRI-Guided Mesothelioma Treatment: (A) Key Steps and Intermediates for the Synthesis of Gd-B-CA-SF, Orthogonally Protected and Functionalized Carborane Cage for BNCT, Gd^3+^ Complex for GdNCT and MRI, Sulfamido Group for CA IX Inhibition and Targeting Moiety; (B) Schematic Illustration of the Neutron Irradiation Experiments of ^157^Gd-^10^B-CA-SF, and Simultaneous Generation of Alpha Particles, Auger Electrons and γ-Rays Thanks to the Presence of ^10^B and ^157^Gd; (C) Schematic Description of *In Vitro* and *In Vivo* Studies: Gd-B-CA-SF Selectively Targets Mesothelioma Cancer Cell and Tumor Model and It Produces a Contrast Enhancement Detectable by MRI

Scheme 1 was created with BioRender (https://biorender.com/hrbj8qf).

## Results and Discussion

### Chemistry: Synthetic Strategies to Obtain Gd-DOTA-sulfamido-carborane (Gd-B-CA-SF)

The theranostic Gd-B-CA-SF is composed of three different functional units: the sulfamido group as the targeting system and CA inhibitor, a carborane cage as the BNCT agent, and the Gd-DOTA complex with the dual role of GdNCT agent and MRI probe. The planned strategy is described in Scheme 2 and Scheme 3. The success of the Gd-B-CA-SF preparation hinged on developing a synthetic strategy to access the key intermediate *C*-(*N*-(1,3-dioxoisoindolin-2-yl))­methyl-*C*′-2-(*N*,*N*-di-*tert*-butoxycarbonyl)­aminomethyl-*o*-carborane **5**. Carborane **5** was designed to allow the selective functionalization of the carborane cage with both the sulfamido group and the Gd-DOTA complex. Thus, phthaloyl (Phth) and *tert*-butoxycarbonyl (Boc) groups were chosen to orthogonally protect diamino carborane **5**, considering the stability of the phthaloyl group in the reaction conditions suitable for Boc deprotection. Symmetric but-2-yne-1,4-diol **1** was chosen as the starting compound with the idea of exploiting a Mitsunobu reaction to synthesize 2-(4-hydroxybut-2-yn-1-yl)­isoindoline-1,3-dione **2** (Scheme 2), which was obtained, according to a published procedure, in a 64% yield.^82^ In contrast, the Mitsunobu reaction was demonstrated to be unsuitable for obtaining *tert*-butyl­(*tert*-butoxycarbonyl)­(4-(1,3-dioxoisoindolin-2-yl)­but-2-yn-1-yl)­carbamate **4**, due to the high basicity of di-*tert*-butoxycarbonylamine. So, a two-step strategy based on the mesylate **3**, quantitatively obtained from alkyne **2**, followed by a nucleophilic substitution with di-*tert*-butoxycarbonylamine, was applied to obtain protected alkyne **4** with an overall yield of 56%.^83^ Both methanesulfonyl- (Ms-) and *p*-toluenesulfonyl- (*p*-Ts-) derivatives can be exploited, leading to almost the same yields for the synthesis of **4**. Since the MsO activation was accomplished in quantitative yields, this latter was chosen as the leaving group in the optimized synthesis. Then the *o*-carboranyl cage was obtained by the dehydrogenative insertion reaction using the Sneddon protocol^84^ in a bmim^+^Cl^–^–PhMe biphasic system affording the orthogonally protected diamino-*o*-carborane **5**. Both natural and ^10^B-enriched derivatives were prepared and obtained in similar yields (41% and 42%, respectively).

**Scheme 2:**
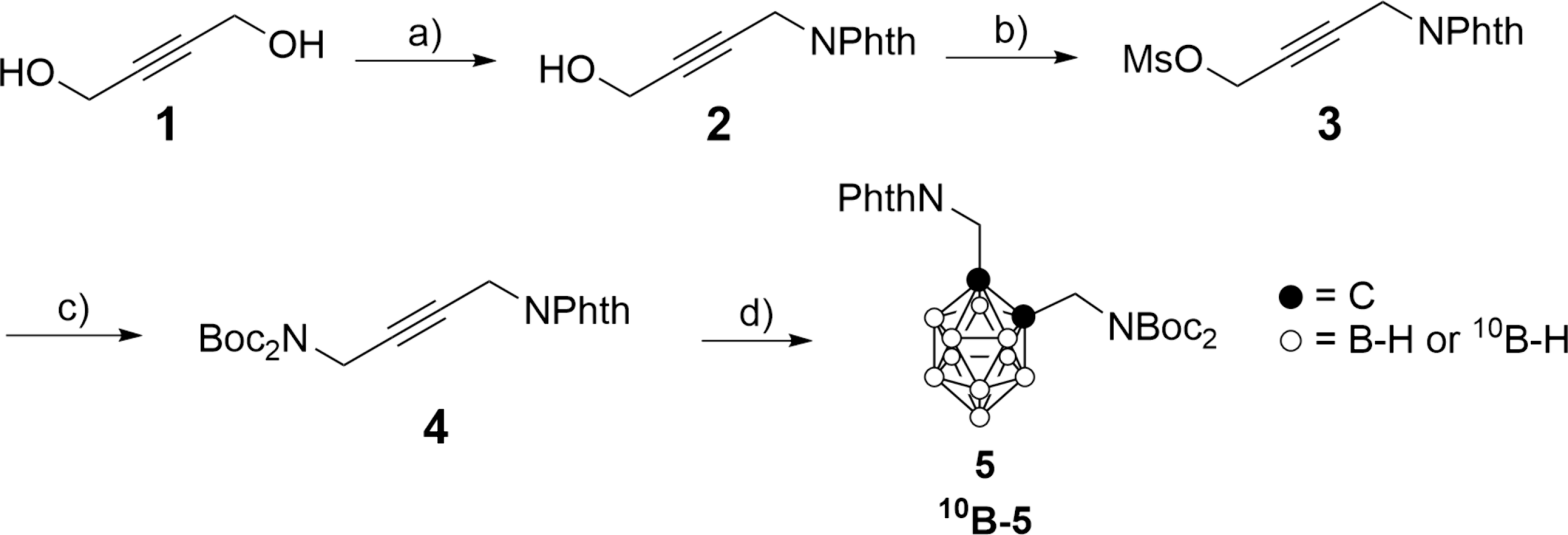
^a^

a Reaction conditions and yields: (a) PhthNH, PPh_3_, DIAD, anhydr. THF, 0 °C to RT, 16 h (64%); (b) MsCl, Et_3_N, anhydr. THF, 0 °C to RT, 1 h (98%); (c) NHBoc_2_, K_2_CO_3_, anhydr. DMF, RT to 60 °C, 16 h (56%); (d) B_10_H_14_ or ^10^B_10_H_14_, bmim^+^Cl^–^, anhydr. PhMe, 110 °C, 1.5 h (**5** 41%, ^10^B-**5** 42%).

**Scheme 3:**
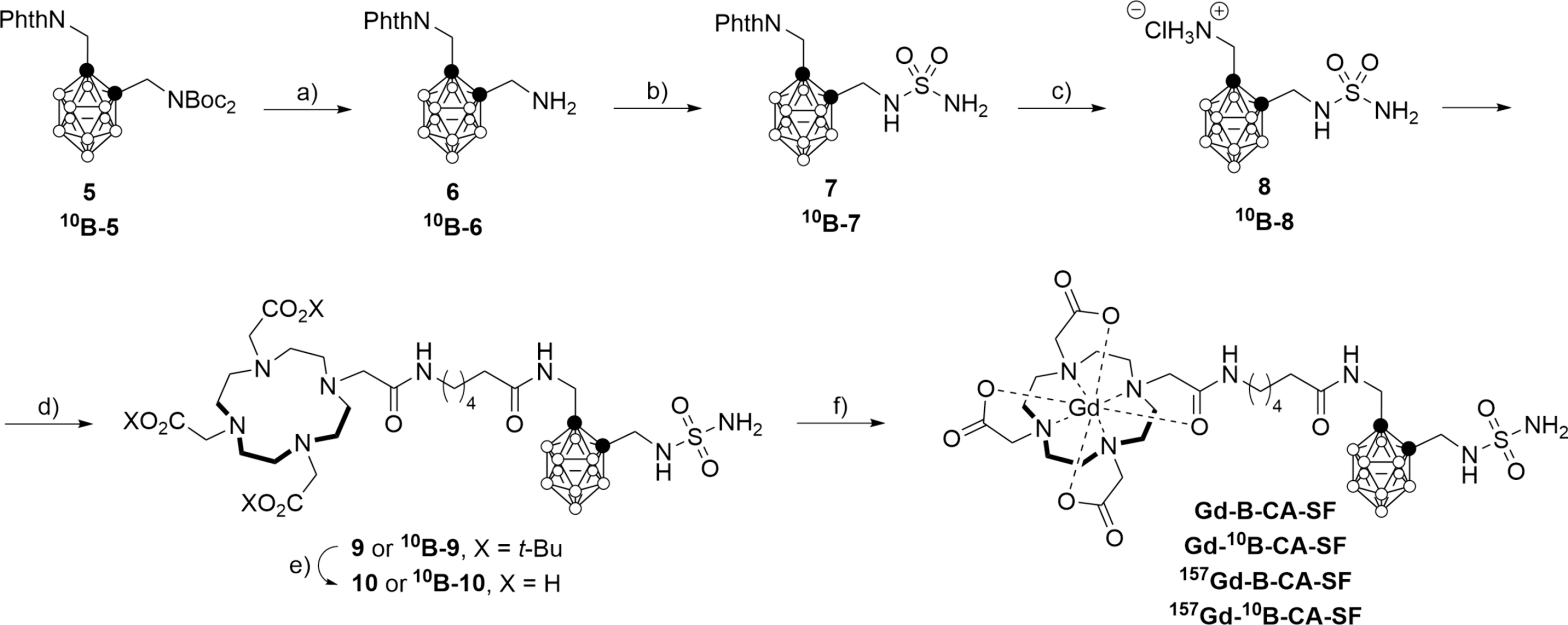
^a^

a Reaction conditions and yields: (a) TFA, CH_2_Cl_2_, RT, 2 h then aq. Na_2_CO_3_ (**6** quant., ^**10**^B-6 quant.); (b) SO_2_(NH_2_)_2_, anhydr. 1,4-dioxane, 110 °C, 2 h (**7** 93%, ^10^B-**7** 92%); (c) NaBH_4_, *i*-PrOH/H_2_O 4:1, RT, 2 h, then glacial AcOH/H_2_O/conc. HCl 4:1:1, 50 °C, 2 h, then CHCl_3_, RT (**8** 81%, ^10^B-**8** 90%); (d) DOTAMA­(O*t*-Bu)_3_-C_6_-COOH, CDMT, NMM, anhydr. CH_2_Cl_2_, 0 °C to RT, 48 h (**9** 49%, ^10^B-**9** 53%); (e) TFA/H_2_O 95:5% v/v, CH_2_Cl_2_ RT, 24 h then TFA, RT, 24 h (**10** quant., ^10^B-**10** quant.); (f) GdCl_3_, H_2_O, RT, 16 h.

As previously outlined, due to the Phth cleavage conditions, Boc groups must be removed first. Thus, under classic conditions, i.e., trifluoroacetic acid (TFA), the aminomethyl-*o*-carborane **6** was obtained in quantitative yields and isolated after an aqueous workup in basic conditions (Scheme 3). Compound **6** was immediately reacted with sulfamide after the evaporation of the solvent, thus minimizing the possibility of cluster degradation. Surprisingly, after 1 h of reaction, product **7** was obtained in 93% yield (92% in the case of ^10^B-**7**). Despite the harsh reaction conditions and the presence of a free amino group, no formation of *nido*-carborane was observed. Once the sulfamide moiety was incorporated, the Phth had to be cleaved, to link the ligand to the carborane scaffold. The usual two-step mild procedure using NaBH_4_ followed by heating in an acidic environment worked well. To prevent any undesired side reaction the second step was carried out at a lower temperature, i.e., 50 °C. Derivative **8** was isolated in 81% yield (80% in the case of ^10^B-**8**). The procedure reported by Kaminsky was efficiently applied to introduce the DOTAMA­(O*t*-Bu)_3_-C_6_-COOH ligand,^85^ by the activation of the carboxylic acid by 2-chloro-4,6-dimethoxy-1,3,5-triazine (CDMT) in the presence of *N*-methylmorpholine (NMM). As shown in Scheme 3, compound **9** was obtained in 49% yield, (53% in the case of ^10^B-**9**), a good result considering the steric hindrance of the macrocycle. Gd-B-CA-SF was obtained after treatment with TFA, to release the carboxylic functionalities, followed by complexation with GdCl_3_. In order to study the possible additive effect of BNCT and GdNCT, natural B/Gd abundance, ^10^B-enriched (^10^B), ^157^Gd-enriched (^157^Gd), and ^10^B/^157^Gd-enriched complexes (^10^B/^157^Gd) Gd-B-CA-SFs were prepared.

### Biology

#### Relaxometric Measurements

The efficiency of an MRI contrast agent is expressed in terms of its millimolar relaxivity (*R*_1p_) that corresponds to the paramagnetic contribution of the longitudinal relaxation rate (1/*T*_1_) of a 1 mM Gd complex solution. The *R*_1p_ of Gd-B-CA-SF measured at 21.5 MHz and 25 °C was 5.9 ± 0.3 s^–1^ mM^–1^. This value was higher than those of clinically used Gd-DOTA (4.7 mM^–1^ s^–1^) and GdHPDO3A (4.3 mM^–1^ s^–1^) complexes, thus indicating an enhanced capability for producing contrast in the MRI image. The increased molecular weight of the compound leads to a longer rotational correlation time for the complex, which causes the observed *R*_1p_ increase.

The increase in the rotational correlation time (τ_r_) was confirmed by the fitting of the Nuclear Magnetic Relaxation Dispersion (NMRD) profile of the free compound Gd-B-CA-SF, reporting the relaxation rates measured at varying magnetic fields (in 0.01–70 MHz) using a Fast Field Cycling relaxometer (Figure 1A). The data were analyzed using the Solomon–Bloembergen–Morgan model (inner sphere contribution) and the Hwang and Freed model (outer sphere contribution). Parameters obtained from the analysis of the NMRD profile data for Gd-B-CA-SF are reported in Table S1 in Supporting Information (SI). The profile of Gd-B-CA-SF alone (0.05 mM) was subsequently acquired in the presence of Bovine Carbonic Anhydrase II (BCA II, 0.5 mM) to demonstrate that the additional increase in *R*_1p_ results from the binding to such a large macromolecule (MW = 30 kDa). The relaxivity peak reached a maximum of 17.7 mM^–1^ s^–1^ (2.8 times higher than that of Gd-B-CA-SF alone), which remained relatively high even under increasing magnetic fields (2 times higher at 70 MHz, corresponding to 1.6 T). This characteristic is particularly beneficial for monitoring biodistribution by MRI, typically performed at magnetic field strengths >1.5 T. The specificity of the interaction between Gd-B-CA-SF and BCA II was confirmed by a competition assay in the presence of an excess of acetazolamide (AZ), a CA inhibitor approved for clinical applications that binds the Zn ion present at the active site of the enzyme. Figure 1B shows that the relaxation enhancement observed in the presence of BCA II disappeared when the sample was preincubated with an excess of AZ.

**Figure 1:**
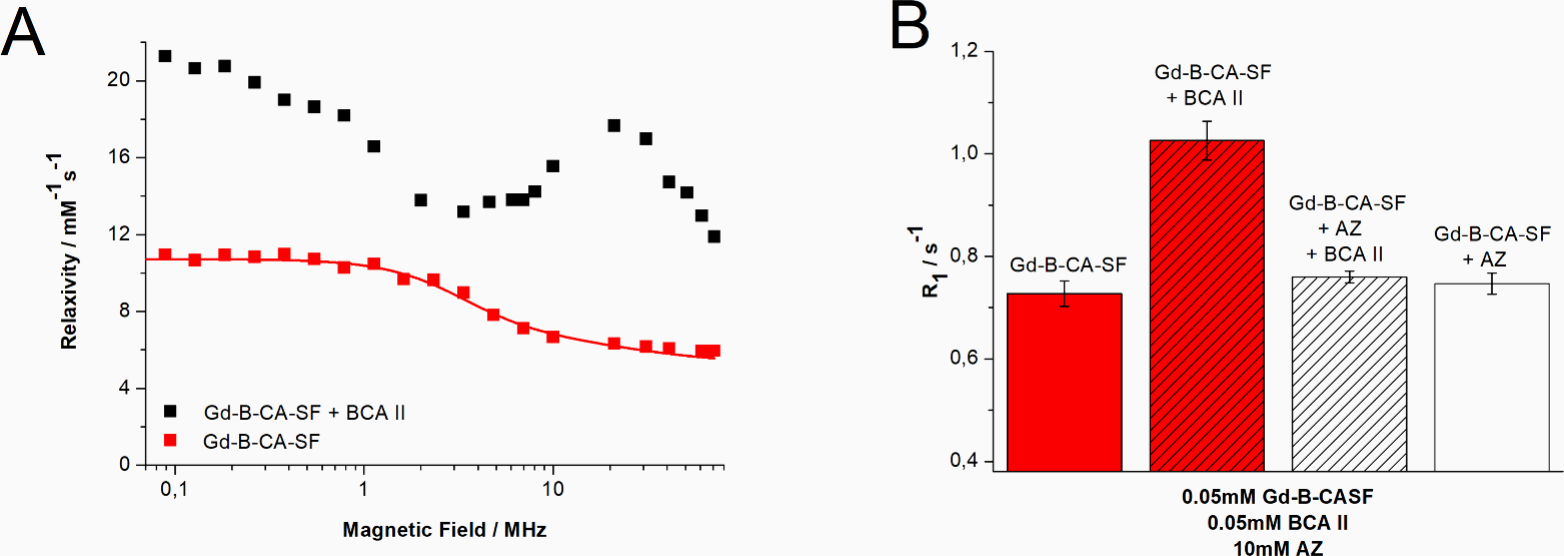
(A) NMRD profile (0.01–70 MHz) acquired at 25 °C of Gd-B-CA-SF alone (0.05 mM) or in the presence of BCA II 0.5 mM; (B) *R*_1_ observed at 21.5 MHz and 25 °C of Gd-B-CA-SF (0.05 mM) in the absence or in the presence of BCA II (0.05 mM). Moreover, *R*_1_ of Gd-B-CA-SF (0.05 mM) in the presence of both AZ (10 mM) and BCA II (0.05 mM) or only AZ (10 mM) was measured. The graph shows the mean ± SD of *R*_1_ evaluated on 3 independent experiments. Statistical analysis was performed by using Student’s *t* test: not significant (ns) *p* > 0.05, *n* = 3; ***p ≤ 0.001, *n* = 3.

#### Inhibition of Human CA II and CA IX and Bovine CA II by CA-SF and Gd-B-CA-SF

The esterase activity of CA II and CA IX has also been reported to be mediated by the zinc-based mechanism responsible for CO_2_ hydration.^86,87^ Consequently, the esterase activity inhibition of BCA II, HCA II, and HCA IX enzymes was evaluated indirectly using a quick colorimetric assay that employs 4-nitrophenyl acetate (*p*NPA) as a substrate, as previously described.^80^ Specifically, the hydrolysis of *p*NPA was monitored at 405 nm to detect 4-nitrophenol (*p*NP) formation. Employing the Michaelis–Menten model, the *V*_max_ and *K*_m_ for *p*NPA with HCA II at a concentration of 5 μg/mL (Table 1) were determined using a *p*NPA concentration range of 0.2–10 mM (see Figure S1, SI). The results confirmed that the esterase activity of human CA II was similar to that of bovine CA II.

**Table 1:** Comparison between the Esterase Activity of the HCA II, HCA IX, and BCA II Enzymes

	*p*NPA	
Enzymes	*K*_m_ (mM)	*V*_max_ (nmol/min)
HCA II	4.5 ± 1.5	1.93 ± 0.32
BCA II	4.2 ± 1.3 ^80^	1.00 ± 0.14 ^80^
HCA IX	7.5 ± 1.6 ^80^	0.36 ± 0.04 ^80^

In order to assess the IC_50_ values (Figure S2 in SI) and the inhibition constants (*K*_i_) of Gd-B-CA-SF for BCA II, HCA II, and HCA IX, the compound was preincubated with the enzymes at different concentrations before the addition of *p*NPA (0.5 mM) as the substrate for colorimetric analysis.

To evaluate the effect of the presence of the Gd-complex, the IC_50_ and *K*_i_ values for Gd-B-CA-SF were compared with those obtained with the precursor derivative CA-SF and the reference inhibitor acetazolamide (AZ). Given its poor solubility in water, CA-SF was first dissolved in DMSO before being combined with an excess of 2-hydroxypropyl-β-cyclodextrin (HP-β-CD in PBS) at a molar ratio of 1:5. In contrast, Gd-B-CA-SF exhibited sufficient solubility in aqueous solutions, allowing the analysis to be carried out without the need for any solubilizing agent.

The *K*_i_ values (Table 2) were calculated using the Cheng–Prusoff equation, as follows:

1 $$K_{i}={\mathrm{IC}}_{50}/\left(1+\left[S\right]/K_{m}\right)$$

where [S] represents the *p*NPA concentration and *K*_m_ is the Michaelis–Menten constant.

**Table 2:** IC_50_ and *K*_i_ Values for HCA II and HCA IX Inhibition by CA-SF and Gd-B-CA-SF

	BCA II		HCA II		HCA IX	
Inhibitor	IC_50_ [μM]	*K*_i_ [μM]	IC_50_ [μM]	*K*_i_ [μM]	IC_50_ [μM]	*K*_i_ [μM]
CA-SF + HP-β-CD	0.067 ± 0.009 ^80^	0.059 ± 0.002 ^80^	0.080 ± 0.004	0.070 ± 0.005	0.7 ± 0.5 ^80^	0.65 ± 0.15 ^80^
Gd-B-CA-SF	1.29 ± 0.15	1.15 ± 0.13	2.0 ± 0.8	1.80 ± 0.79	1.4 ± 0.7	1.30 ± 0.61
AZ	0.085 ± 0.003 ^80^	0.072 ± 0.002 ^80^	0.095 ± 0.002	0.083 ± 0.003	0.006 ± 0.002 ^80^	0.006 ± 0.003 ^80^

The results shown in Table 2 demonstrated that the conjugation with the Gd-complex leads to a reduction in the overall inhibitory effectiveness of the sulfamido-carborane (CA-SF) against BCA II, HCA II, and HCA IX, likely resulting from a reduced binding affinity for these enzymes. Nevertheless, when comparing HCA II and HCA IX, Gd-B-CA-SF shows a slightly greater selectivity for CA IX, our investigation’s primary focus.

#### Cell Viability Assay (MTT)

The MTT assay, under normoxic conditions (5% CO_2_, 37 °C), was then employed to evaluate the *in vitro* cytotoxicity of Gd-B-CA-SF, following the protocol reported by Azzi et al.^88^ Two different cell lines were considered in this study: AB22 (murine mesothelioma) and Met-5a (immortalized human mesothelium).^89^ As shown in Figure 2, the toxicity of Gd-B-CA-SF is significantly lower in both cell lines compared to that of CA-SF alone. However, a slightly higher cytotoxic effect was observed in the AB22 cancer cell line compared to the Met-5a healthy mesothelium (*p* values are reported in Table S2). This result is the consequence of the higher affinity shown by CA-SF for CA II that is expressed in both AB22 and Met-5a, while CA IX is overexpressed predominantly in cancer cells. Moreover, these data underline the critical role of the carborane moiety in enhancing the hydrophobic interactions between biologically active compounds and their receptors. Although CA-SF demonstrated a stronger overall ability to inhibit both the enzymes, it lacked specificity, as its increased inhibition of CA II impacts both normal and cancerous cells. In contrast, Gd-B-CA-SF provides better specificity by primarily targeting CA IX, the enzyme that is largely overexpressed in tumor cells.

**Figure 2:**
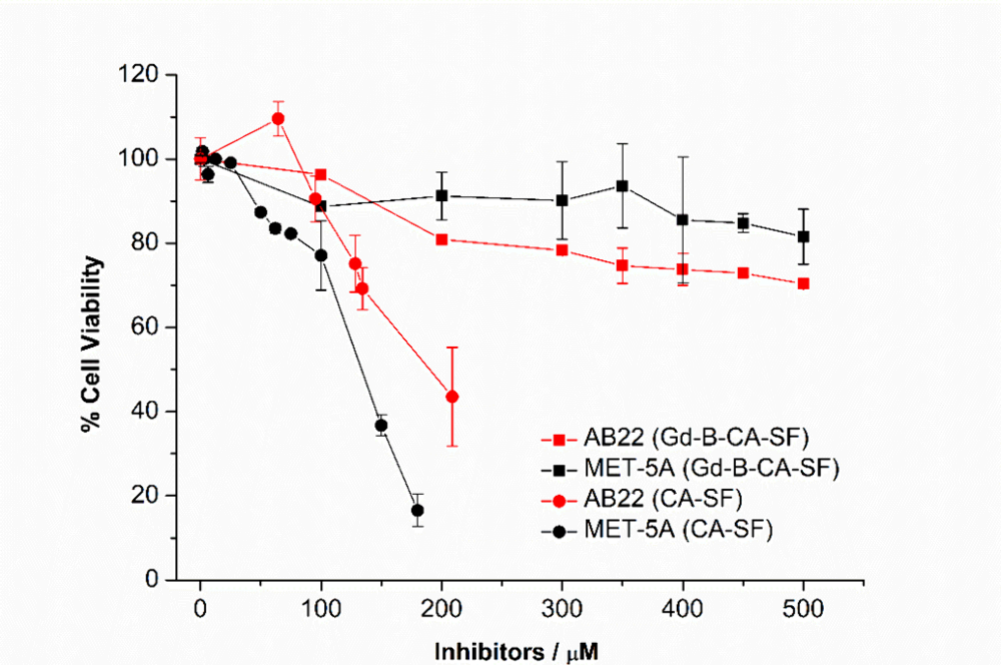
Percentage of viability (measured by MTT assay) of AB22 and Met-5a measured after 24 h of incubation with increasing concentrations of CA-SF or Gd-B-CA-SF. The viability of untreated control cells was set at 100%. Graphs show the mean ± SD of % viability evaluated in 3 independent experiments. Statistical analysis was performed by using Student’s *t* test with OriginPro 8.5 software: not significant (ns) *p* > 0.05, *p ≤ 0.05, **p ≤ 0.01, ***p ≤ 0.001.

#### Cell Uptake Experiment

To assess whether the levels of B and Gd uptake in AB22 and Met-5a were enough for an effective BNCT/GdNCT treatment, the cells were incubated for 24 h at 37 °C and 5% CO_2_ with increasing concentrations of Gd-B-CA-SF, and Gd-HPDO3A (trade name: Prohance, Bracco Imaging S.p.A.), a highly soluble and well-tolerated compound currently utilized in clinical settings, was incubated in the same conditions and used as a nonspecific reference compound. After incubation, the cells were washed with ice-cold PBS and detached, applying two different approaches: the first method used only EDTA for total collection (both membranes bound and internalized) and the second one used trypsin + EDTA for obtaining only the internalized portion. In fact, cell surface proteins and their ligands are mostly cleaved after trypsin treatment. After sonication for 30 s in ice, cell samples were mineralized to perform ICP-MS. The values of Gd obtained by ICP-MS were normalized to the total mg of proteins measured in the cells (by Bradford assay). AB22 exhibited greater uptake of both compounds compared to Met-5a. The uptake of Gd-B-CA-SF in AB22 was higher than that of Gd-HPDO3A due to the overexpression of CA IX in cancer cells^90^ (Figure 3A) (*p* = 0.0001). The lower uptake observed with the reference compound, Gd-HPDO3A, confirmed the specific binding of Gd-B-CA-SF. Additionally, comparison of the total cell-associated Gd-B-CA-SF with the internalized fraction in the AB22 cell line (Figure 3B) revealed a significant difference (*p* = 0.0011). This suggests that a substantial proportion of Gd-B-CA-SF remained bound to the cell surface rather than being fully internalized. This surface binding is presumably due to its interaction with CA IX, which is expressed on the extracellular surface of AB22 cancer cells. In contrast, this difference was not observed in the Met-5a cell line, further supporting the overexpression of CA IX only in AB22 cells.^90^ This observation is corroborated by the significantly (*p* = 0.0001) higher amount of Gd-B-CA-SF associated with AB22 cells compared to Met-5a cells. These findings indicate that Gd-B-CA-SF exhibits limited cellular internalization in both cells, likely due to its relatively high hydrophilicity.

**Figure 3:**
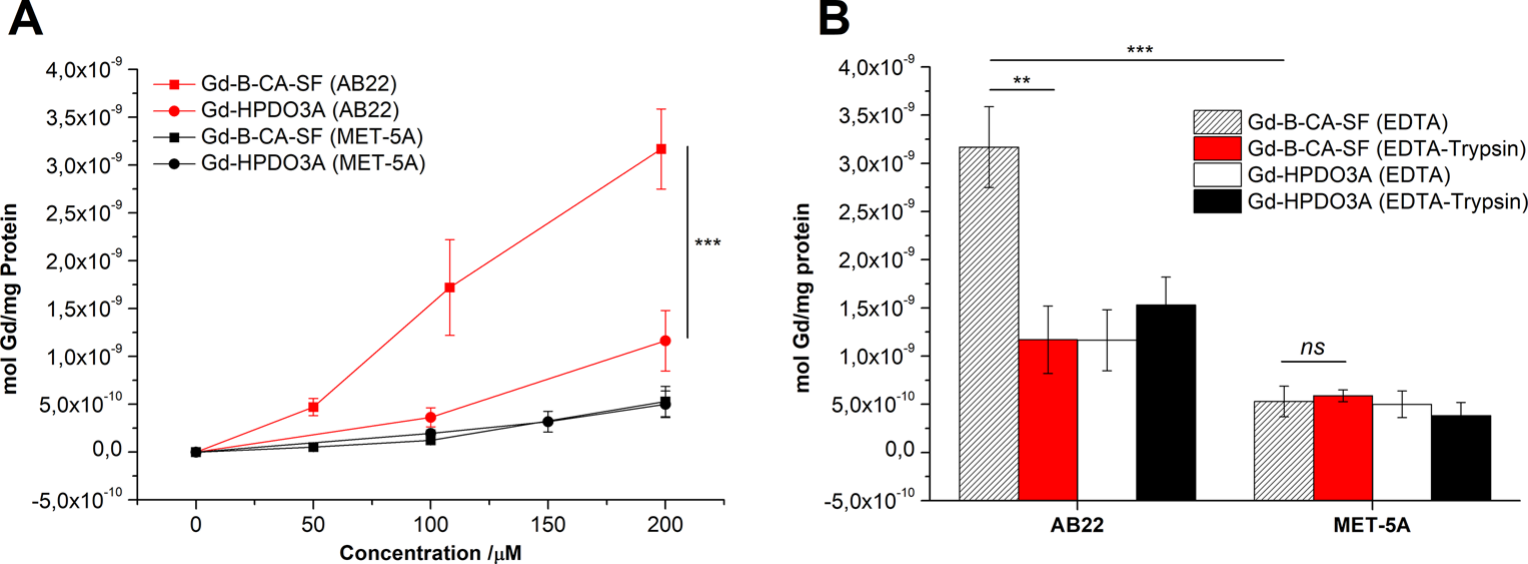
(A) Uptake studies on AB22 and Met-5a cells incubated in the presence of increasing concentration of Gd-B-CA-SF and Gd-HPDO3A and detached with EDTA (for 24 h at 37 °C, 5% CO_2_). (B) Comparison between AB22 and Met-5a cells incubated at 0.2 mM with Gd-B-CA-SF or Gd-HPDO3A and detached with EDTA or EDTA-Trypsin (for 24 h at 37 °C, 5% CO_2_). Graph and histogram show the mean ± SD of mol Gd/mg protein evaluated on three independent experiments. Statistical analysis was performed by using Student’s *t* test: **p ≤ 0.01; ***p ≤ 0.00.

To evaluate the binding specificity, a competition assay was performed by preincubating AB22 and Met-5a cells in the presence of an excess of AZ (4 mM) 1 h before the addition of 0.2 mM Gd-B-CA-SF (Figure 4). After a 6 h total incubation, the Gd uptake decreased significantly in only AB22 (*p* = 0.0185). These results suggest that AB22 cells exhibit a specific binding and internalization mechanism for Gd-B-CA-SF, whereas Met-5a cells do not demonstrate the same level of specificity.

**Figure 4:**
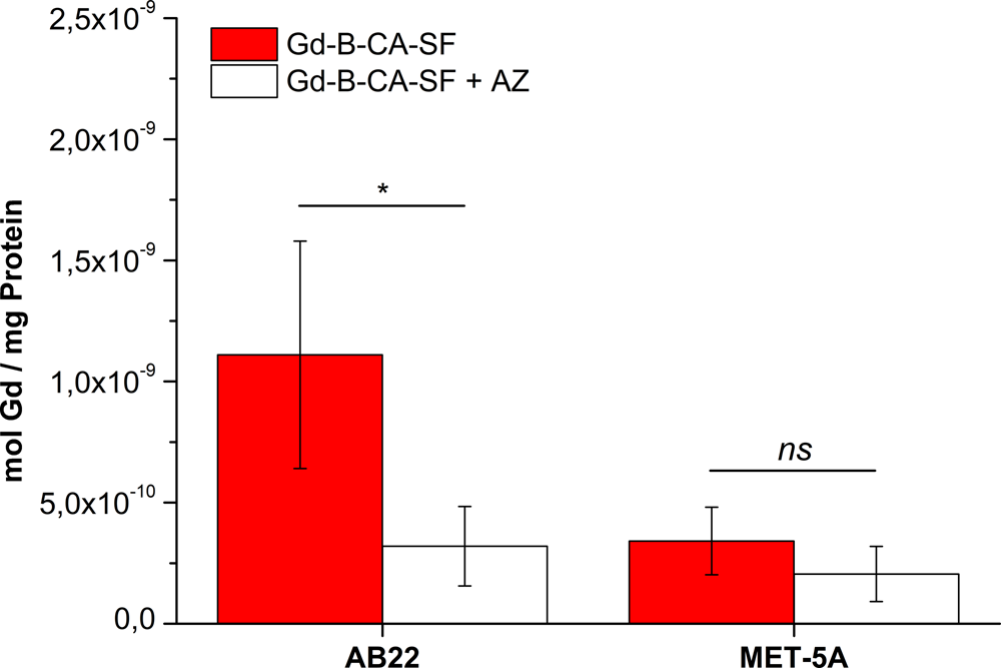
Competition assay performed by incubating AB22 and Met-5a for 6 h at 37 °C with 0.2 mM Gd-B-CA-SF in the absence or in the presence of 4 mM AZ. The histogram shows the mean ± SD of mol Gd/mg protein evaluated in three independent experiments. Statistical analysis was performed by using Student’s *t* test: not significant (ns) *p* > 0.05, *n* = 3; *p ≤ 0.05, *n* = 3.

As reported in Table 3, the calculated total boron content (both membrane-bound and internalized) in AB22 was sufficient (>20 μg/g) to perform BNCT when Gd-B-CA-SF was incubated at the highest concentration (0.2 mM) for 24 h. The μg of boron per g of tissue were determined considering a Gd/B ratio of 1:10 and a cell density of about 10^8^ cells/cm^3^, calculated using an average cell diameter of 15–20 μm.^91,92^

**Table 3:** Total and Internalized Boron in AB22 and Met-5a Cells

Cell line	Boron^a^ (EDTA)	Boron^b^ (EDTA-Trypsin)
AB22	21 ± 3	8 ± 3
Met-5a	2.5 ± 0.7	3 ± 1

a μg of boron/g of total tissue.

b μg of boron/g of intracellular tissue.

#### Magnetic Resonance Imaging (MRI)

To investigate whether the uptake of Gd-B-CA-SF by AB22 and Met-5a cells can generate a detectable MRI contrast, *T*_1_-weighted images were acquired at 7 T using glass capillaries filled with cell pellets. In Figure 5 is shown the well detectable signal intensity (SI) enhancement observed in AB22 cells (+57% ± 3) incubated for 24 h at 37 °C with 0.2 mM Gd-B-CA-SF compared to untreated AB22 CTRL cells. Interestingly, the contrast enhancement in AB22 cells was considerably higher than that observed in Met-5a cells under the same conditions (+14% ± 7), therefore showing that Gd-B-CA-SF preferentially accumulates in mesothelioma. This difference in uptake is consistent with the previous ICP-MS results shown above. This observation was confirmed by the differences in the *R*_1,obs_ measured by a saturation recovery sequence (Table S3 in SI).

**Figure 5:**
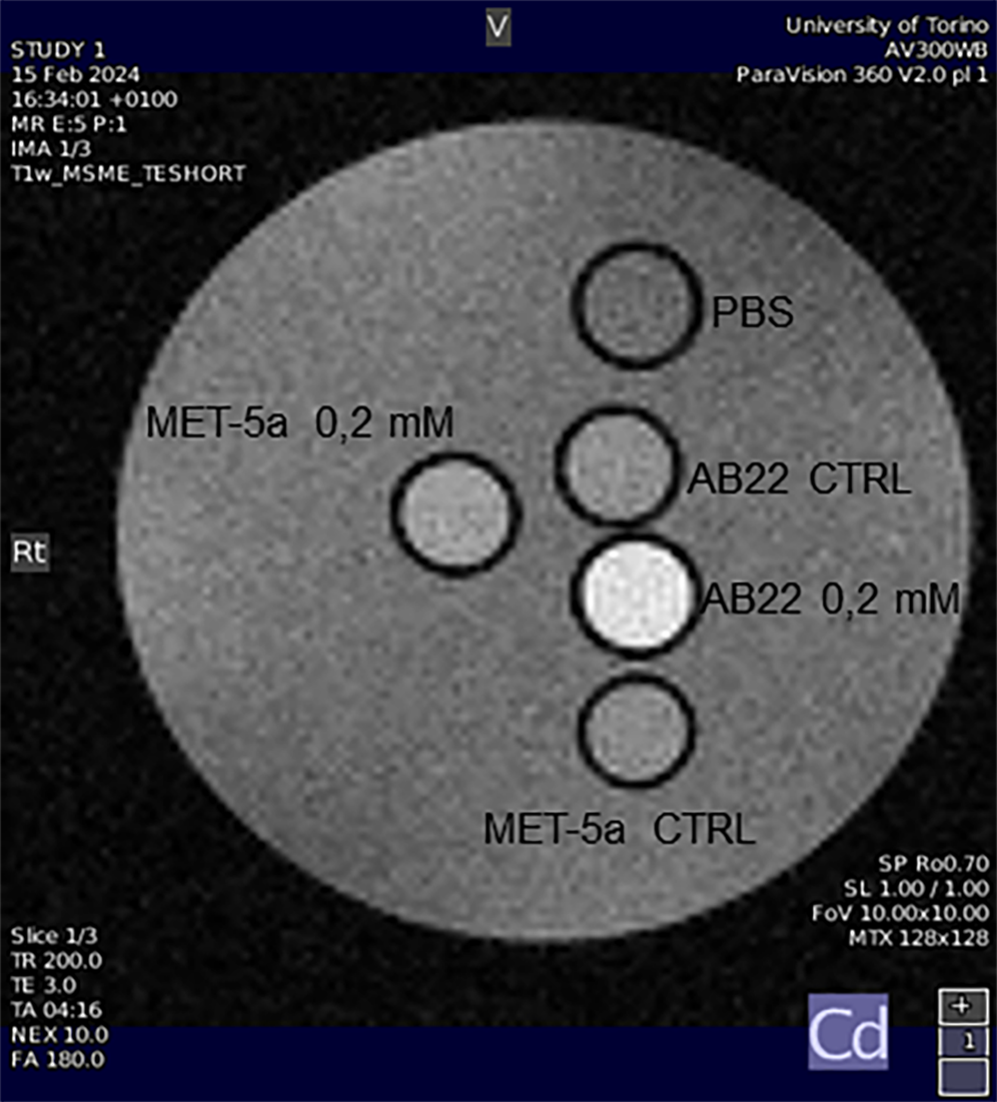
*T*_1_-weighted spin–echo MR image of an agar phantom containing glass capillaries filled with untreated AB22 and Met-5a cells (CTRL) or cells incubated with 0.2 mM Gd-B-CA-SF for 24 h at 37 °C.

#### *In Vitro* NCT Treatment of AB22 Cells

Based on previous observations, AB22 cells demonstrated higher Gd-B-CA-SF uptake and were selected for neutron irradiation studies. The coexistence of B and Gd within a single molecule enables the investigation of all possible combinations of both enriched and naturally occurring compounds, allowing for individual evaluations or a combined analysis to identify any additional or synergistic effects related to neutron capture from nuclides ^10^B and ^157^Gd.

The following irradiated and nonirradiated groups were established, where the treated cells were incubated for 24 h in the presence of 0.2 mM Gd-B-CA-SF.

Irradiated groups: untreated cells (CTRL IRR); cells treated with the compounds containing: (i) natural abundance B and Gd (Gd-B-CA-SF IRR), (ii) ^10^B-enriched (Gd-^10^B-CA-SF IRR), (iii) ^157^Gd-enriched (^157^Gd-B-CA-SF IRR), and (iv) both ^10^B- and ^157^Gd-enriched (^157^Gd-^10^B-CA-SF IRR).
Nonirradiated groups: untreated cells­(CTRL NN IRR) and cells treated with the natural abundance B and Gd compound (Gd-B-CA-SF NN IRR).

The irradiated groups were exposed to the radiation field in the thermal column of the TRIGA Mark II reactor at the University of Pavia (30 kW reactor power for 15 min). The percentage of viable cells was measured 24 h after exposure to neutron irradiation for both irradiated and nonirradiated groups, obtained using the trypan blue exclusion method. The viability (%) was calculated with respect to the untreated and nonirradiated control cells (CTRL NN IRR) that was set at 100%. The results (Figure 6A) indicated that, the day after irradiation, the % of viable cells was almost like the CTRL NN IRR reference in any conditions, with the exception of ^157^Gd-^10^B-CA-SF IRR, containing both ^157^Gd- and ^10^B-enriched isotopes, which exhibited a viability of approximately 64% (*p* = 0.0149).

**Figure 6:**
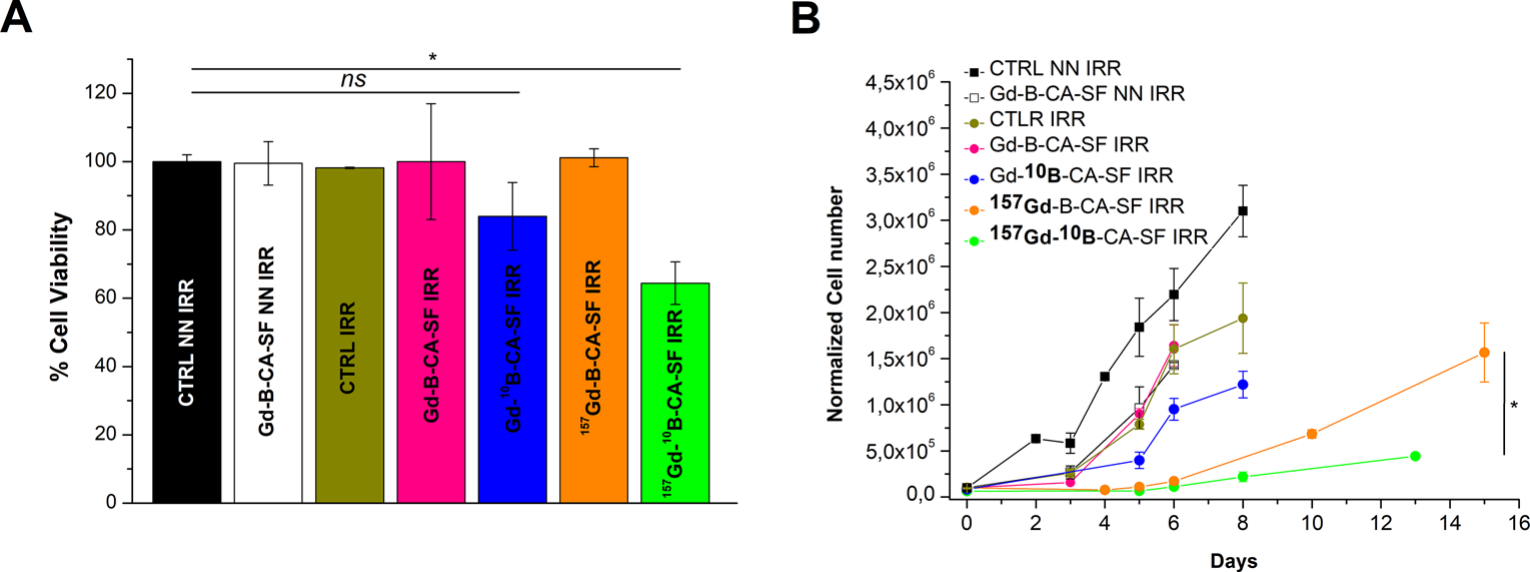
(A) Percentage viability of irradiated and nonirradiated groups of AB22 treated with Gd-B-CA-SF variants measured 24 h after neutron irradiation. The viability (%) was calculated with respect to that of the untreated and nonirradiated control cells (CTRL NN IRR) which was set at 100%. (B) Proliferation curves of AB22 cells surviving neutron irradiation monitored within 15 days postirradiation. The histogram shows the mean ± SD of % cell viability. Graph shows the mean ± SD of the normalized cell number. Statistical analysis was performed by using Student’s *t* test: not significant (ns) *p* > 0.05, *n* = 2; *p ≤ 0.05, *n* = 2. (See details in Table S4).

Long-term cytotoxic effects were subsequently assessed following cell proliferation for 15 days. A marked inhibition of cell proliferation was observed in irradiated AB22 cells treated with ^157^Gd-B-CA-SF and even more in those irradiated and treated with ^157^Gd-^10^B-CA-SF (Figure 6B). Additionally, a decrease in cell proliferation rate was observed in the nonirradiated Gd-B-CA-SF (Gd-B-CA-SF NN IRR), and this effect remained unchanged even when irradiated (Gd-B-CA-SF IRR). This indicates that in the absence of enriched isotopes the NCT effect is negligible, and the observed reduction in cell proliferation is mainly attributed to the carbonic anhydrase inhibition. Another interesting consideration is that AB22 treated with ^157^Gd-B-CA-SF exhibited slower cell regrowth upon irradiation with respect to those treated with Gd-^10^B-CA-SF. This finding is consistent with the explanation that α-particles primarily exert their effects through direct DNA damage,^93^ which is negligible when ^10^B is localized on the outer cell membrane, as previously indicated. Conversely, the literature reported that targeting the cell membrane effectively induces cancer cell death via Auger electrons^43–45^ produced, along with γ-rays, during the nuclear reaction involving ^157^Gd and neutrons. The synergistic effect of Auger electrons with γ-rays characterized by their extended range of action can further improve the toxicity, reaching directly to the nucleus of the cell. Scheme 4 shows the experimental workflow.

**Scheme 4:**
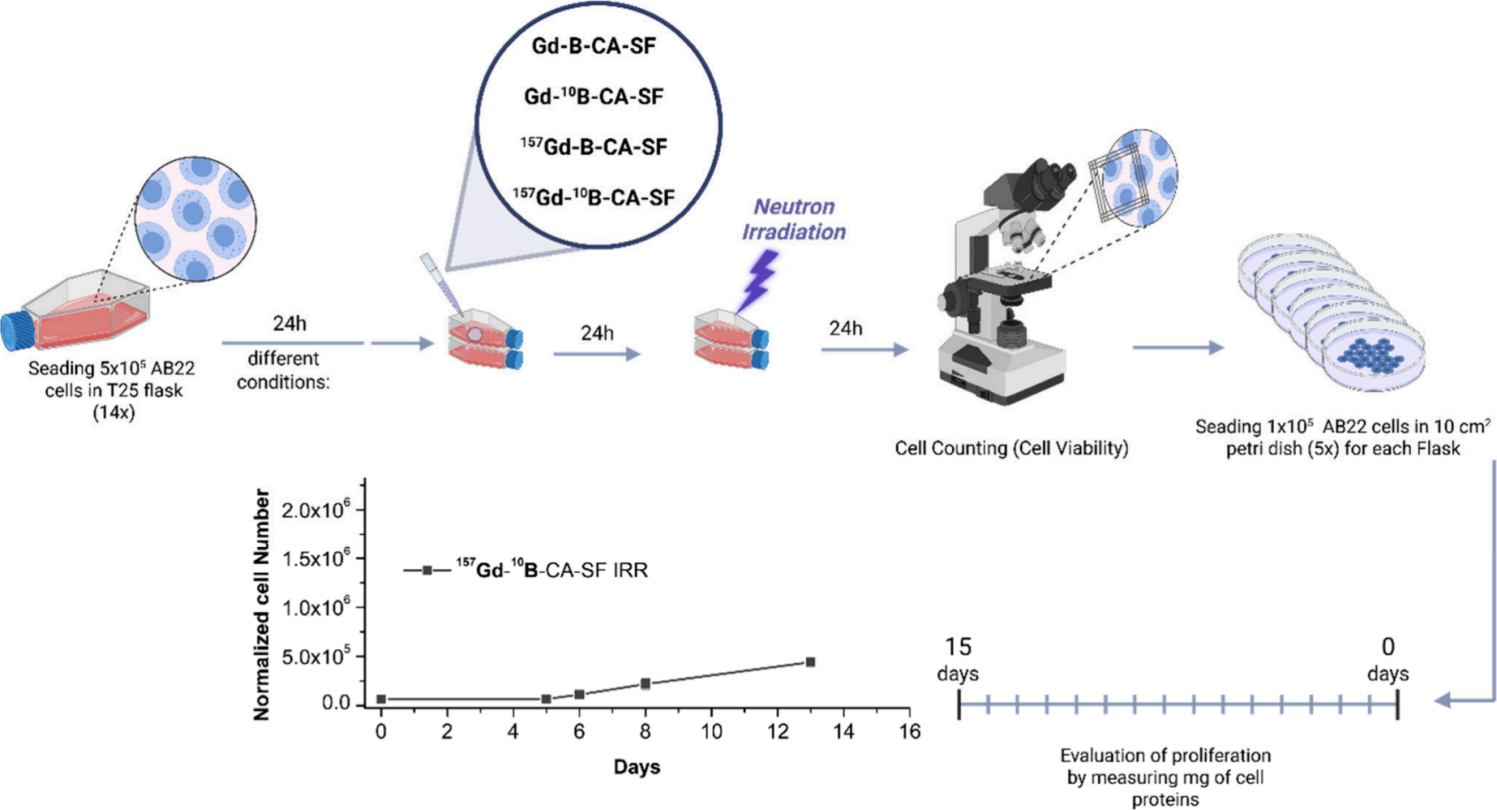
Schematic Workflow of the *In Vitro* Irradiation Experiment, Summarizing The Main Steps: Seeding of Cells, Incubation with Different Derivatives of Gd-B-CA-SF, Neutron Irradiation, Assessment of Cell Viability and Proliferation Rate

Created with BioRender (https://BioRender.com/3ce47v4).

#### *In Vivo* Biodistribution by MRI and ICP-MS

*In vivo* biodistribution experiments were performed using a syngeneic mouse model, achieved by implanting AB22 cells subcutaneously at the lower neck region of BALB/C mice. After 10 days, the AB22 tumors reached a volume of approximately 152 ± 24.9 mm^3^. At that time, the tumor-bearing mice intravenously received a bolus of Gd-B-CA-SF or Gd-HPDO3A (used as nonspecific control reference) at a dose of 0.1 and 1 mmol/kg as expressed in terms of gadolinium and boron content, respectively. *T*_1_-weighted MR-images were acquired before and 1, 3.5, 6, and 24 h after Gd-B-CA-SF or Gd-HPDO3A administration. Scheme 5 shows the *in vivo* biodistribution experimental workflow. Figure 7A shows the characteristic axial images acquired at the level of implanted tumors 3.5 h post-treatment, and Figure 7B depicts the signal intensity (SI) enhancement expressed as a percentage for every time interval. The data presented in Figure 7B demonstrate a significantly higher accumulation of Gd-B-CA-SF in tumors compared to the nonspecific agent Gd-HPDO3A, thereby confirming its effective targeting of pathological cells *in vivo*.

**Scheme 5:**
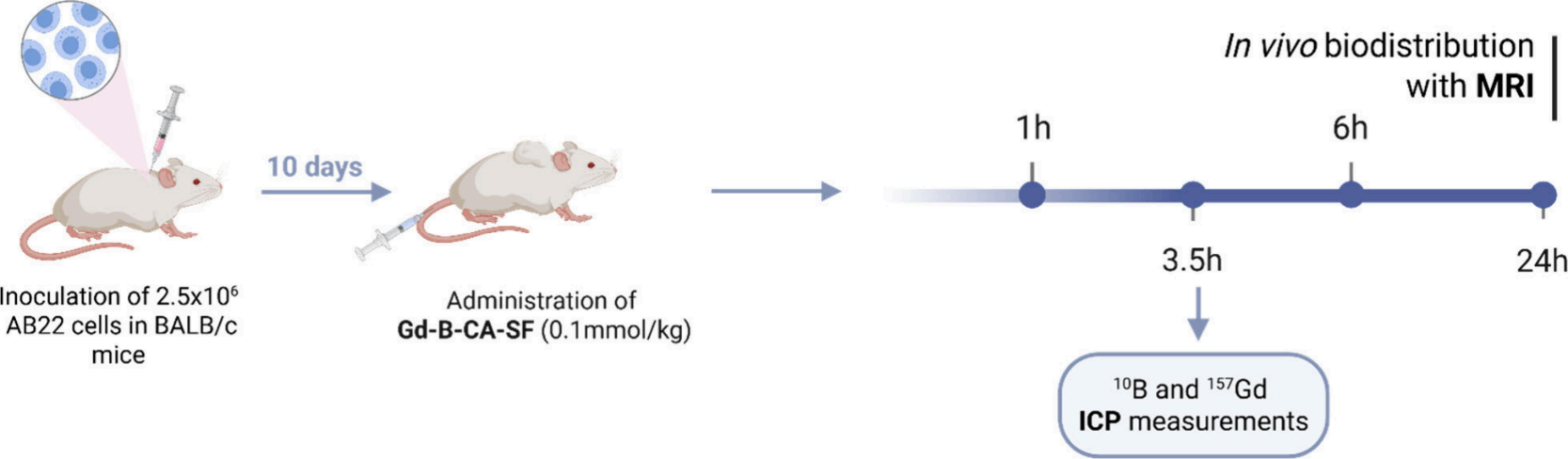
Experimental Workflow for the *In Vivo* Biodistribution Study^a^

a Mice were inoculated with AB22 cells and allowed to establish tumors, followed by administration of Gd-B-CA-SF. Biodistribution was evaluated at 1, 3.5, 6, and 24 h postinjection using MRI, and at 3.5 h using ICP-MS.

Created by BioRender (https://BioRender.com/3hivxyl).

**Figure 7:**
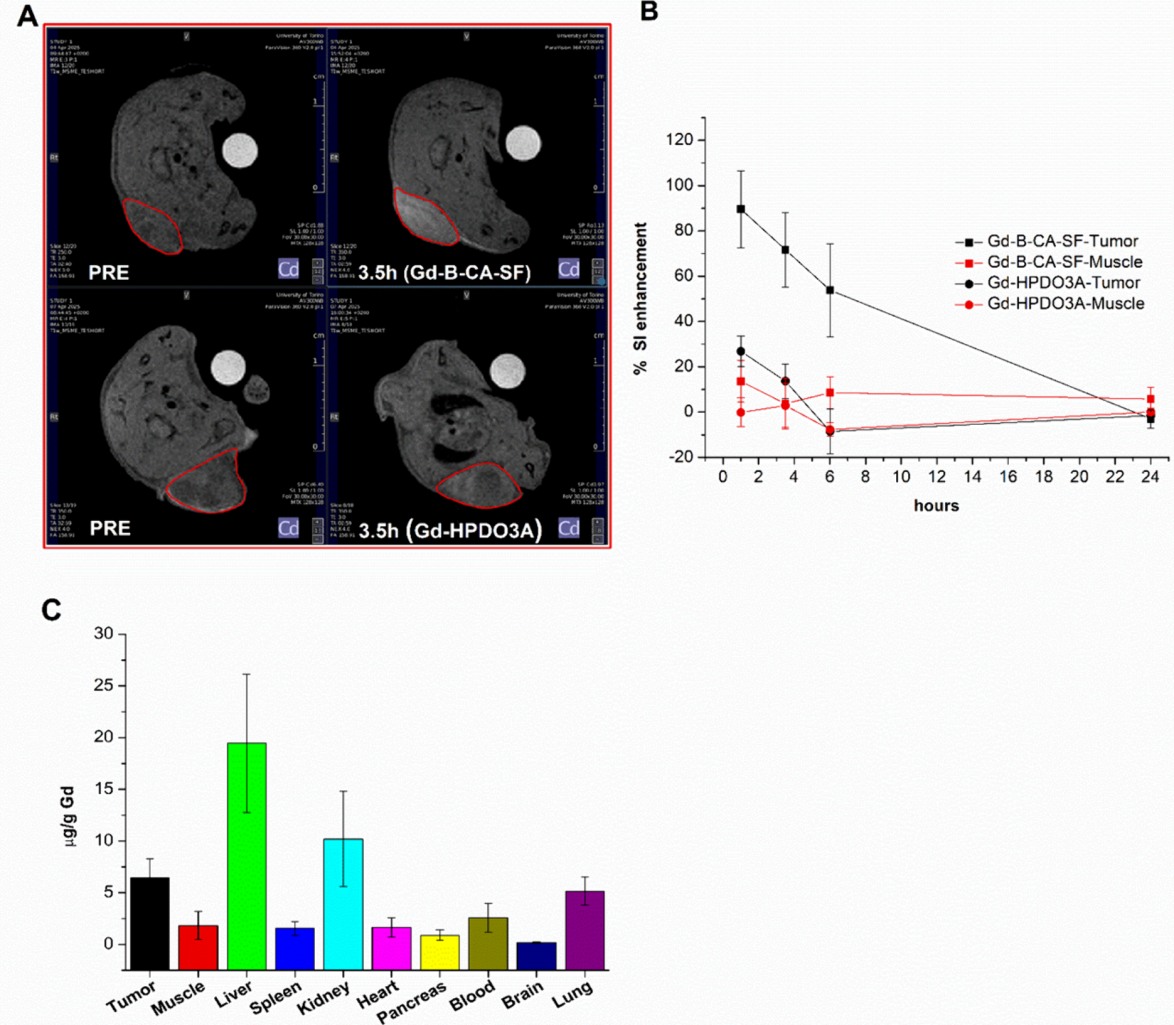
(A) *T*_1_-weighted spin–echo MR images (7 T) acquired before (PRE) and 3.5 h after Gd-B-CA-SF or Gd-HPDO3A administration. (B) MRI signal intensity (SI) enhancements (%) measured in tumor and muscle over time after the administration of Gd-B-CA-SF or Gd-HPDO3A. Graph shows the mean ± SD of % normalized signal enhancement. Statistical analysis was performed by using Student’s *t* test. See the details in Table S5. (C) Gd biodistribution at 3.5 h in AB22 tumor bearing mice after the administration of Gd-B-CA-SF. Gd concentration was determined by ICP-MS. Error bars indicate the SD.

Moreover, the Gd and B concentrations (Table 4) in the tumor, surrounding muscle, and liver were calculated from the Gd-induced relaxation enhancement observed after Gd-B-CA-SF *in vivo* administration as described in SI.

**Table 4:** Biodistribution of Boron Atoms in Mesothelioma Tumor Bearing Mice

Time (h) post injection	Tumor [Boron] μg/g	Liver [Boron] μg/g	Muscle [Boron] μg/g
1	13.0 ± 2.6	57 ± 17	2.00 ± 0.94
3.5	9.8 ± 2.5	29 ± 10	0.48 ± 1.50
6	7.3 ± 2.9	6.5 ± 5.4	1.10 ± 0.94
24	–0.36 ± 0.51	0.58 ± 0.61	0.69 ± 1.50

Although the maximum boron concentration measured in the tumor was about 13 ppm, the tumor/healthy muscle ratio was 6.5/1, a value extremely high allowing one to perform BNCT avoiding healthy tissue damage. The high liver uptake of Gd-B-CA-SF was expected due to the highly amphiphilic character of the compound. This drawback can be overcome using a neutron shield placed on the mice abdomen and, in the case of human patients, through collimated neutron beams that can significantly decrease the amount of neutrons absorbed by the liver.

Then the *in vivo* biodistribution was assessed by ICP-MS by measuring μg of Gd per g of tissue in different organs, 3.5 h after the administration of 0.1 mmol/kg of Gd-B-CA-SF. Figure 7C shows that only the liver and kidneys accumulate more Gd than the tumors and that the low Gd concentration found in the brain allows us to exclude Gd retention in this organ.

Finally, the concentrations of Gd and B in the main target organs, measured by ICP-MS, were compared with those determined by MRI in the same mice (*n* = 3) at 3.5 h postadministration (Table 5).

**Table 5:** Biodistribution of Gd and B Contents in AB22 Mesothelioma Tumor-Bearing Mice 3.5 h after Gd-B-CA-SF Administration, As Determined by Both MRI and ICP-MS^a^

	Gd (μg/g) by MRI	Gd (μg/g) by ICP-MS	B (μg/g) by MRI	B (μg/g) by ICP-MS
Tumor	16 ± 1.49	6.42 ± 1.85	10.97 ± 1.02	4.25 ± 0.88
Muscle	1.15 ± 2.12	1.84 ± 1.37	0.79 ± 1.45	1.64 ± 1.18
Liver	70.24 ± 32.33	19.45 ± 6.69	48.16 ± 22.17	12.46 ± 5.30
Kidney	13.02 ± 1.55	10.20 ± 4.62	8.93 ± 1.06	5.59 ± 2.42

a Values are presented as mean ± SD.

Table 5 shows that the concentrations of both Gd and B measured in the tumor, liver, and kidneys determined by ICP-MS were lower than those determined by MRI. However, quantitative comparisons between *in vivo* and *ex vivo* modalities are subject to methodological challenges.^94^ Indeed, *ex vivo* determination by ICP-MS has limitations due to sample processing and sampling, as subsequent tissue processing can lead to partial tissue loss, in particular in the peripheral area. Moreover, the millimolar relaxivity (*R*_1p_) of the Gd-complex can vary slightly within different tissue compartments, introducing further variability. This issue could be addressed by performing tailored *in vivo* studies to determine contrast enhancement and Gd concentrations in various tissues. However, by measuring both Gd and B concentrations in tumor and other tissues, we were able to confirm that the molar B/Gd ratio remains about 10 also after *in vivo* administration, thus confirming the Gd-B-CA-SF complex stability.

#### Toxicological Analysis

##### Evaluation of Renal Function

The evaluation of renal function was performed through the analysis of BUN and creatinine, which revealed comparable values between the control group (treated with vehicle (PBS)) and the group treated with Gd-B-CA-SF. These findings suggest that the administration of Gd-B-CA-SF does not adversely affect kidney function (Table 6).

**Table 6:** Blood Biochemical Values of BUN and Creatinine in BALB/c Mice 24 h after Treatment with Gd-B-CA-SF or PBS^a^

	Gd-B-CA-SF	PBS
BUN (mg/dL)	18 ± 1	16 ± 2
Creatinine (mg/dL)	<0.4	<0.4

a *n* = 3. Data are presented as mean ± SD.

##### Morphology

Histological analyses were also performed with treated and untreated mice. The liver showed a well-preserved structure of the acinar architecture in the treated mice (see Figure 8). No signs of damage were observed at the level of the portal triad, the hepatic cords, and the sinusoids. No difference was detected between the mice treated with Gd-B-CA-SF and those treated with PBS. In the kidney, cortex, outer and inner medulla, and papilla were well preserved in both groups of mice. Compared with the kidneys from the vehicle group, a scanty number of apical vacuoles were observed in cells of the convoluted tubules from mice treated with Gd-B-CA-SF. The glomeruli were histologically normal, and no signs of interstitial inflammation were observed. In the brain, the hippocampus and neuron cortex layers were well organized. No signs of damage were observed either in the glial elements or in the white matter (Figure 8).

**Figure 8:**
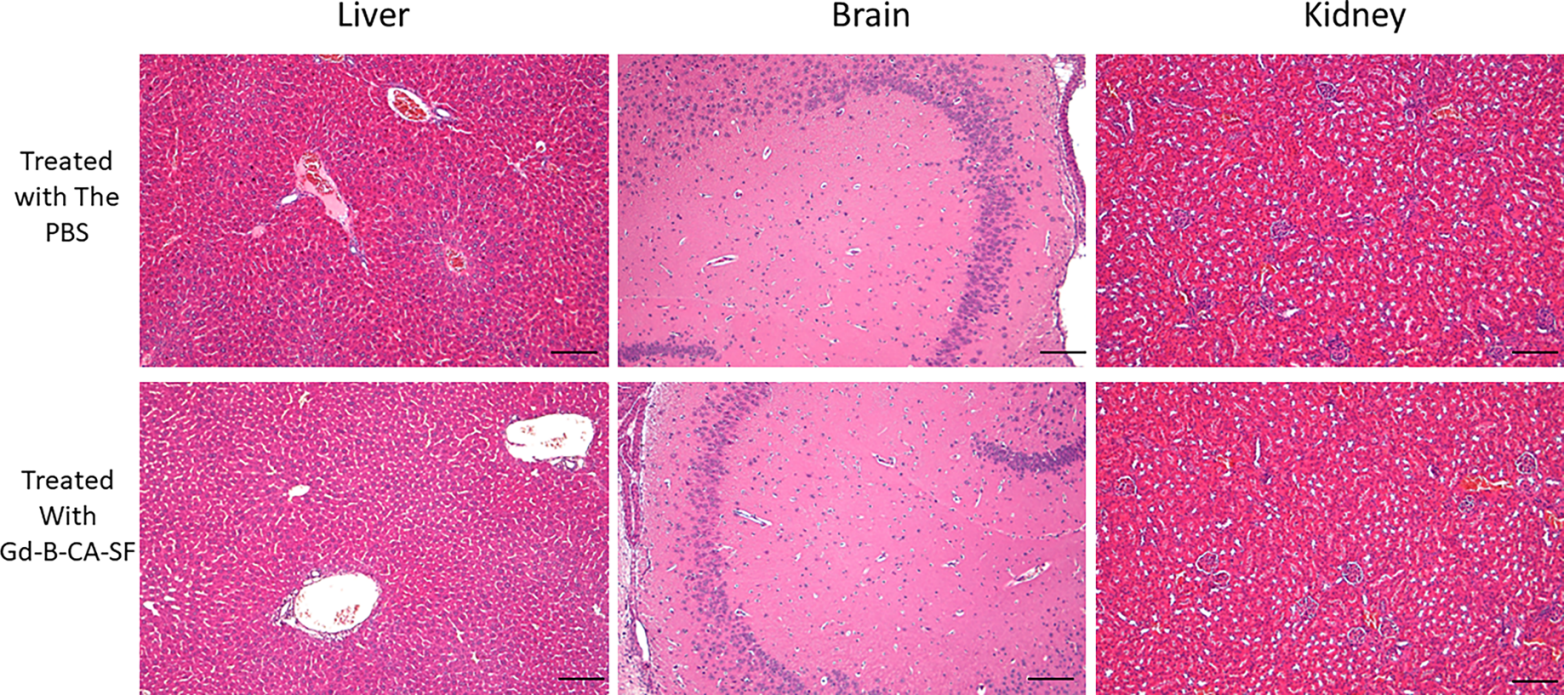
Representative images of excised liver, brain, and kidney from mice treated with PBS (vehicle) and Gd-B-CA-SF, Scale Bars = 200 μm, Magnification (H&E × 10).

#### *In Vivo* Tumor Growth Assessment upon NCT Application

AB22 tumor bearing mice (*n* = 16) were prepared as described previously, and when tumors reached a volume of approximately 55 ± 17 mm^3^, they were utilized for he NCT study. From the cell studies reported above it was determined that the compound containing both enriched ^157^Gd and ^10^B (^157^Gd-^10^B-CA-SF) was the most promising derivative, showing the highest toxicity in AB22 after NCT. To assess the *in vivo* efficacy of ^157^Gd-^10^B-CA-SF on AB22 tumor growth exploiting the benefit of NCT, two separate groups of AB22 tumor-bearing mice were established and irradiated for 15 min in the thermal column of the TRIGA Mark II reactor (reactor power 250 kW): one group received ^157^Gd-^10^B-CA-SF at a dose of 0.1 mmol Gd/kg and 1 mmol B/kg (^157^Gd-^10^B-CA-SF IRR group, *n* = 5), while the other group was not treated with the compound but subjected to thermal neutron irradiation (CTRL IRR group, *n* = 5). NCT was performed 3.5 h after ^157^Gd-^10^B-CA-SF injection as at this time the highest tumor to surrounding healthy tissue (muscle) boron ratio (20/1) was obtained. Moreover, the amount of boron in the liver was dramatically reduced after 3.5 h compared to 1 h (Table 4). These groups were compared to the control not irradiated group (CTRL NN IRR group, *n* = 6). The weight of the three groups was monitored until the end of the experiment (Figure S3). Tumor volumes were measured for the following 22 days by analyzing *T*_2_ weighted MRI images acquired at 7 T (Scheme 6, *in vivo* irradiation experimental workflow).

**Scheme 6:**
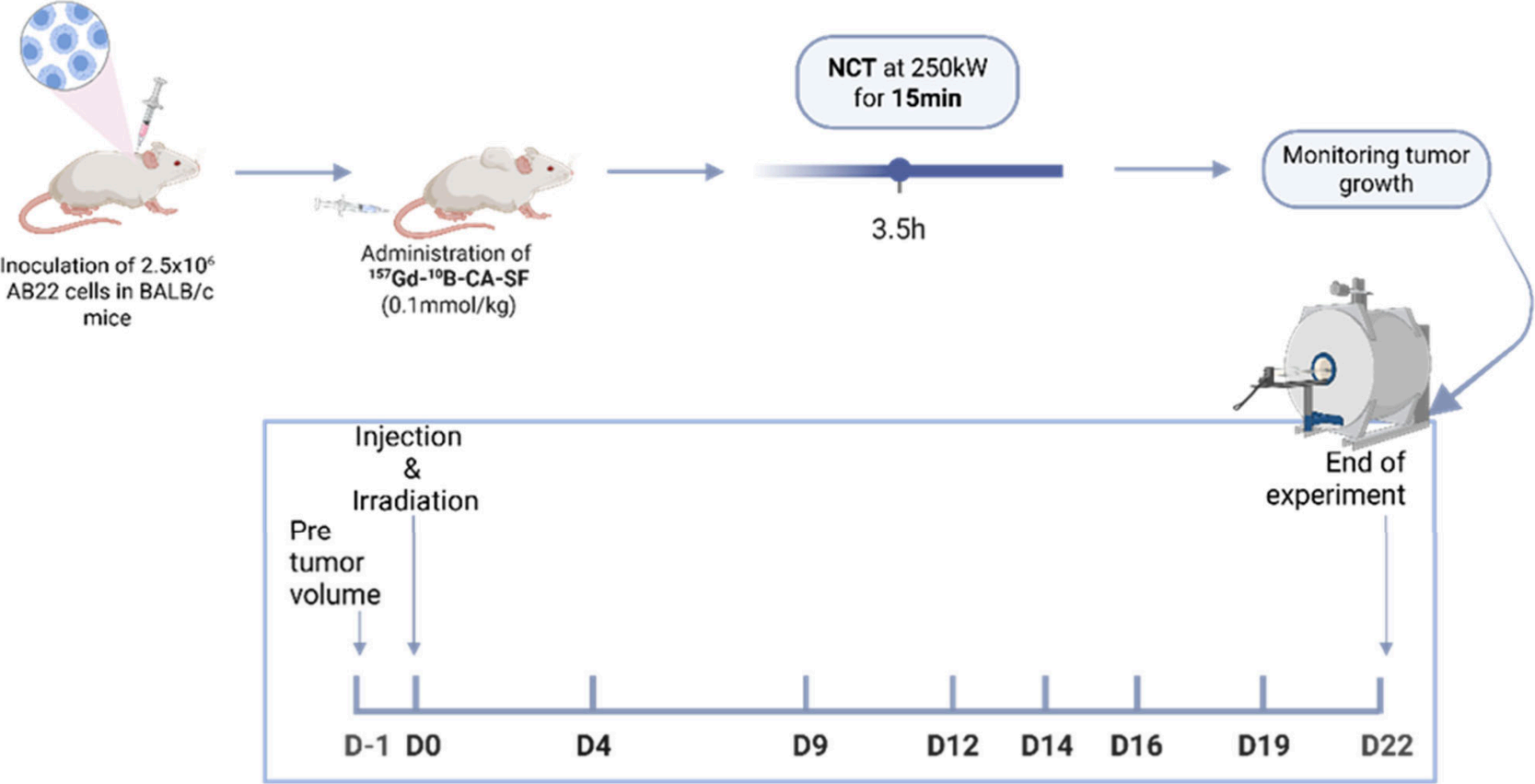
Schematic Workflow of the *In Vivo* Irradiation Experiment, Summarizing the Main Steps: Inoculating Mice with AB22 Cells, Incubation with ^157^Gd-^10^B-CA-SF, Neutron Irradiation (3.5 h Postinjection for 15 min), and Evaluating the Tumor Growth

Created by BioRender (https://BioRender.com/q6xy41k).

Figure 9 shows that the administration of ^157^Gd-^10^B-CA-SF combined with NCT significantly inhibits mouse tumor growth compared to the CTRL NN IRR group (*p* = 0.01 at the end point). In contrast, the treatment response in the CTRL IRR group was more heterogeneous, showing a slight reduction in tumor growth that was not statistically significant compared to that in the CTRL NN IRR group (Table S6). This emphasizes that the presence of both ^157^Gd and ^10^B in the tumor, even at relatively low concentrations, was highly effective.

**Figure 9:**
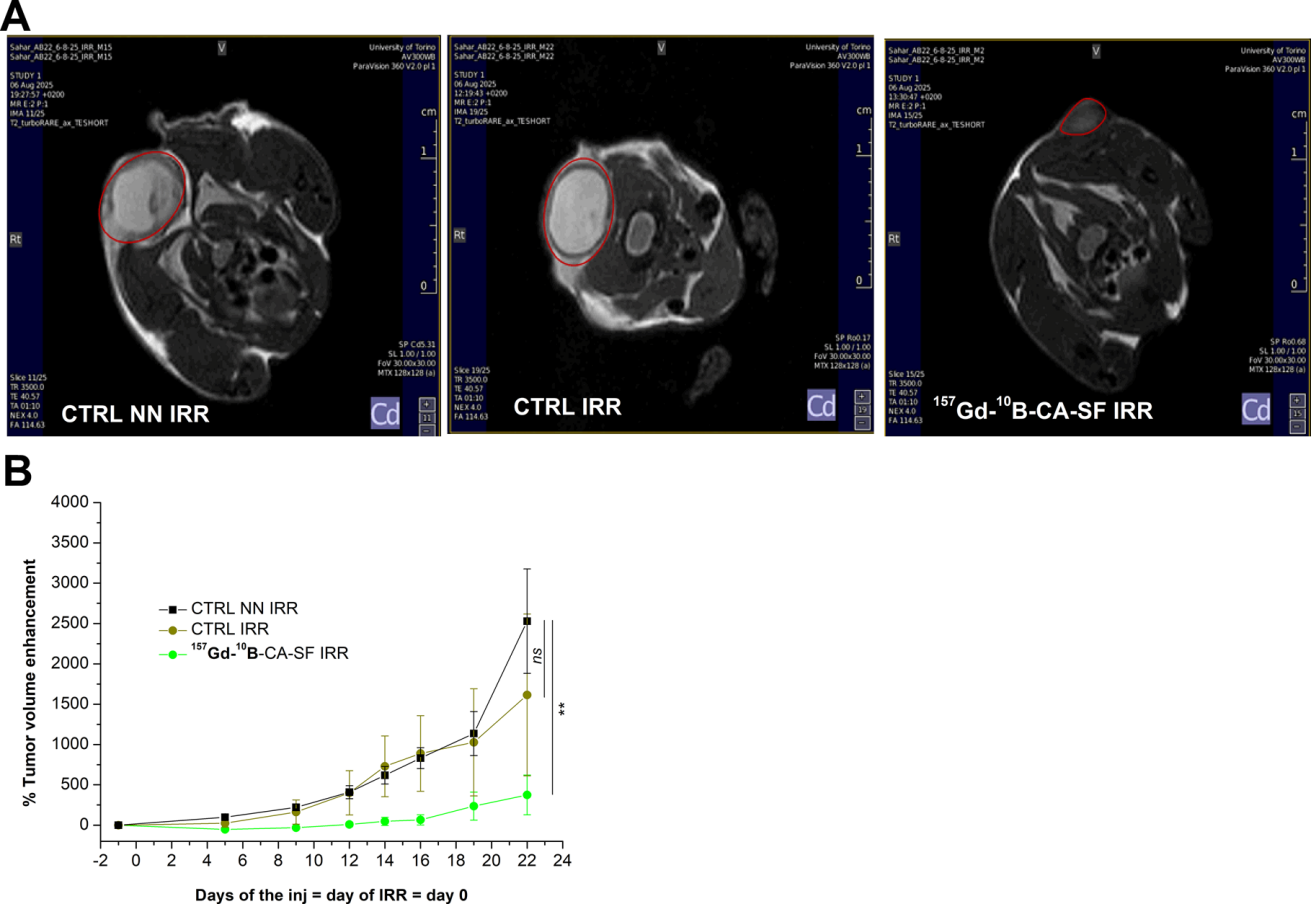
Tumor-growth evaluation performed after ^157^Gd-^10^B-CA-SF treatment combined with NCT. (A) Representative *T*_2_ weighted images (7 T): Control not irradiated, control irradiated, and ^157^Gd-^10^B-CA-SF treated and irradiated mice, monitored by MRI (7 T) on day 14 from administration and irradiation. (B) Tumor volume enhancement % measured by *T*_2_-weighted MRI on CTRL NN IRR, CTRL IRR, and ^157^Gd-^10^B-CA-SF IRR mice. Statistical analysis was performed by using Student’s *t* test: not significant (ns) *p* > 0.05 and **p ≤ 0.01. (Details in Table S6).

## Conclusions

Identifying innovative targeting methods has become critically important due to the recent impetus in the application of neutron capture therapy (NCT) in clinical settings, driven by the establishment of new accelerator-based neutron sources in various countries. Indeed, the exclusive utilization of reactors as sources of neutrons has historically constrained the extensive integration of NCT into standard treatment protocols.^95–97^ Both the development of more selective delivery agents and the generation of an epithermal neutron beam with definite features are necessary for NCT to be considered in the clinic. Therefore, this study explored the potential of a novel multivalent agent, Gd-sulfamido-carborane (Gd-B-CA-SF), for MRI-guided treatment of mesothelioma by integrating BNCT, GdNCT, and carbonic anhydrase (CA) inhibition. To our knowledge this is the first case where the effect of B and Gd natural abundance or enriched in ^10^B or/and ^157^Gd isotopes, contained in the same molecule targeted to the cell membrane, was studied. The conjugation of CA-SF, containing the sulfamido group able to bind the enzyme active site, to a Gd-complex offers two significant advantages: it allows the observation of biodistribution using MRI and enhances hydrophilicity, leading to improved solubility in aqueous environments. The biological evaluation demonstrated that Gd-B-CA-SF selectively targets cancer cells, particularly those overexpressing CA IX, while sparing healthy cells. Moreover, neutron irradiation experiments highlighted the therapeutic efficacy of Gd-B-CA-SF, particularly when enriched with ^157^Gd and ^10^B. The additional effect could be attributed to the localized damage induced on the cell membrane by Auger electrons and γ-rays emitted during neutron capture by ^157^Gd, contributing to increased damage even when Gd-B-CA-SF predominantly remains at the cell surface.

This finding was further validated *in vivo* in the same subcutaneous mesothelioma tumor model established under the neck. Although relatively low concentrations of ^157^Gd and ^10^B were detected in the tumor region (16 and 10 μg/g, respectively), a pronounced reduction in tumor growth was observed. This effect was evident compared with the CTRL IRR and statistically significant relative to the CTRL NN IRR. These results demonstrate that the therapeutic efficacy of irradiated ^157^Gd-^10^B-CA-SF arises from its specific binding to carbonic anhydrase, together with the synergistic action of Auger electrons, γ-rays, and α-particles. Moreover, due to the selectivity, the compound predominantly accumulates in the tumor after the liver and kidney (which can be shielded during NCT), thereby minimizing potential off-target effects.

Compared to nanoparticles, which often face limitations such as poor tissue penetration, slower clearance, and variable biodistribution, the relatively low molecular weight of Gd-B-CA-SF offers distinct advantages, improved solubility, and more efficient tissue penetration and diffusion. These properties, combined with its dual capacity to provide targeted therapy and enable real-time imaging, represent a powerful strategy for personalized medicine. This integrated theranostic approach allows precise monitoring of biodistribution, accurate evaluation of therapeutic response, and optimized treatment planning that are fundamental for BNCT. These findings highlight its translational potential as a theranostic agent for future clinical applications in cancer therapy.

## Supplementary Material



## Notes

Experimental details, materials, and methods, ^1^H NMR spectra for all compounds, DEPT, COSY, and ^13^C NMR spectra for all new compounds; biology experimental details (PDF)
