# Supplementary material for: A Comparative Study of 157Gd and 10B Effect in a Carborane-Based Theranostic Agent for Membrane-Targeted Carbonic Anhydrase IX Inhibition and MRI-Guided Neutron Capture Therapy in Mesothelioma Treatment
Source: ACS Cent Sci. 2025 Oct 1;11(11):2215–29. doi: 10.1021/acscentsci.5c01632 (PMC12670288; doi:10.1021/acscentsci.5c01632)

A Comparative Study of ^{157}Gd and ^{10}B Effect in a Carborane-Based Theranostic Agent for Membrane-Targeted CA IX Inhibition and MRI-Guided Neutron Capture Therapy in Mesothelioma Treatment

Sahar Rakhshan,[a‡] Alberto Lanfranco,[b‡] Diego Alberti,[a] Polyssena Renzi,[b] Ayda Zarechian,[a] Sabrina Elkhanoufi[a], Juan Carlos Cutrin [a‡], Nicoletta Protti,[c,d] Simonetta Geninatti Crich,[a*] Annamaria Deagostino.[b*]

(a) Department of Molecular Biotechnology and Health Sciences; University of Torino, via Nizza 52, 10126, Torino, Italy. (b) Department of Chemistry, University of Torino, via P. Giuria 7, 10125, Torino, Italy. (c) Department of Physics, University of Pavia, via Agostino Bassi 6, Pavia 27100, Italy. (d) Nuclear Physics National Institute (INFN), Unit of Pavia, via Agostino Bassi 6, Pavia 27100, Italy

E-mail: annamaria.deagostino@unito.it; simonetta.geninatti@unito.it

Chemistry 3

General 3

Procedures 4

2-(4-Hydroxybut-2-yn-1-yl)isoindoline-1,3-dione (2). 4

4-(1,3-Dioxoisoindolin-2-yl)but-2-yn-1-yl methanesulfonate (3). 4

tert-Butyl(*tert*-butoxycarbonyl)(4-(1,3-dioxoisoindolin-2-yl)but-2-yn-1-yl)carbamate (4). 4

General procedure for the ionic liquid-mediated dehydrogenative insertion reaction. 5

C-(*N*-(1,3-dioxoisoindolin-2-yl))methyl-*C'*-2-(*N*,*N*-di-*tert*-butoxycarbonyl)aminomethyl-*o*-carborane (5). 5

^{10}B-enriched-*C*-(*N*-(1,3-dioxoisoindolin-2-yl))methyl-*C'*-2-(*N*,*N*-di-*tert*-butoxycarbonyl)aminomethyl-*o*-carborane (^{10}B-5). 5

General procedure for the Boc group removal. 6

C-(*N*-(1,3-dioxoisoindolin-2-yl))methyl-*C'*-2-aminomethyl-*o*-carborane trifluoroacetic acid salt (6•TFA). 6

^{10}B-enriched-*C*-(*N*-(1,3-dioxoisoindolin-2-yl))methyl-*C'*-2-aminomethyl-*o*-carborane trifluoroacetic acid salt (^{10}B-6•TFA). 6

General procedure for the sulfamidic group installation. 7

C-(*N*-(1,3-dioxoisoindolin-2-yl))methyl-*C'*-(sulfamoylamino)methyl-*o*-carborane (7). 7

^{10}B-enriched-*C*-(*N*-(1,3-dioxoisoindolin-2-yl))methyl-*C'*-(sulfamoylamino)methyl-*o*-carborane (^{10}B-7). 8

General procedure for the phthaloyl group removal. 8

C-aminomethyl-*C'*-(sulfamoylamino)methyl-*o*-carborane hydrochloric salt (8). 8

^{10}B-enriched-*C*-aminomethyl-*C'*-(sulfamoylamino)methyl-*o*-carborane hydrochloric salt (^{10}B-8). 9

General procedure for the amide formation. 9

C-(6-(2-(4,7,10-tris(2-(*tert*-butoxy)-2-oxoethyl)-1,4,7,10-tetraazacyclododecan-1-yl)acetamido)hexanamido)methyl-*C'*-(sulfamoylamino)methyl-*o*-carborane (9). 9

^{10}B-enriched-*C*-(6-(2-(4,7,10-tris(2-(*tert*-butoxy)-2-oxoethyl)-1,4,7,10-tetraazacyclododecan-1-yl)acetamido)hexanamido)methyl-*C'*-(sulfamoylamino)methyl-*o*-carborane (^{10}B-9). 10

General procedure for *tert*-butyl esters hydrolysis. **10**

C-(6-(2-(4,7,10-tris-carboxymethyl-1,4,7,10-tetraazacyclododecan-1-yl)acetamido)hexanamido)methyl-*C'*-(sulfamoylamino)methyl-*o*-carborane (10). 11

^{10}B-enriched-*C*-(6-(2-(4,7,10-tris-carboxymethyl-1,4,7,10-tetraazacyclododecan-1-yl)acetamido)hexanamido)methyl-*C'*-(sulfamoylamino)methyl-*o*-carborane (^{10}B-10). 11

Biology **12**

Gd-B-CA-SF complexation **12**

Determination of CA Esterase Activity **12**

Relaxometric measurements **13**

Nuclear Magnetic Resonance Dispersion (NMRD) profiles. **14**

Cell lines **14**

MTT Assay **14**

Uptake Experiments **15**

Inductively coupled plasma mass spectrometry (ICP-MS) **16**

Competition Assay **16**

Evaluation of *in vitro* Gd-B-CA-SF uptake by magnetic resonance imaging (MRI) **16**

Cell Irradiation **17**

Cell viability and Proliferation Assay **17**

Experimental Mice and Induction of Transplantable Tumors **18**

***In vivo* MRI evaluation** **18**

Gd and B Concentration Determination by MRI **19**

Toxicological studies **20**

***In vivo* Irradiation** **21**

References **23**

Chemistry

General

Flasks and all equipment used for the generation and reaction of moisture-sensitive compounds were dried by electric heat gun under N_2. All commercially available reagents and solvents were used as received. Anhydrous solvents were purchased from Sigma-Aldrich and Acros. THF was distilled from $LiAlH_4$ then benzophenone ketyl and CH_2Cl_2 from CaH_2 prior to use. *n*-BuLi (2.5 M in hexanes) was obtained from Sigma-Aldrich. Decaborane was bought from KATCHEM spol. s. r. o. Products were purified by preparative column chromatography on Macherey-Nagel silica gel for flash chromatography, 0,04-0,063 mm/230-400 mesh. When specified, silica gel is deactivated with 1% of Et_3N. C_{18}-Reversed phase silica gel (fully end-capped) was purchased from Sigma-Aldrich. Reactions were monitored by TLC using Silica gel on TLC-PET foils Merck, layer thickness 0.2 mm, medium pore diameter 60 Å or Nano silica gel RP-18W (C_{18}) on TLC-aluminium foils Macherey-Nagel, layer thickness 0.15 mm, medium pore diameter 60 Å. Carboranes and their derivatives were visualized on TLC plates using a 5% $PdCl_2$ aqueous solution in HCl. ^1H NMR spectra were recorded at NMR Jeol ECZR 600 MHz or Bruker NeoAvance 400 MHz, ^{13}C NMR spectra at 150 MHz and 100 MHz, ^{11}B NMR spectra at 192.5 MHz, in $CDCl_3$, CD_3OD or acetone-*d6*. Data were reported as follows: chemical shifts in ppm from tetramethylsilane as the internal standard, integration, multiplicity (s = singlet, d = doublet, t = triplet, q = quartet, dd = double-doublet, m = multiplet, br = broad), coupling constants (Hz), and assignment. ^{13}C NMR and ^{11}B NMR spectra were measured with complete proton decoupling. DEPT experiments were carried out with a DEPT 135 sequence. Chemical shifts were reported in ppm from the residual solvent as an internal standard. The MS flow-injection analyses were run on a "Orbitrap IQ-X high resolution" mass spectrometer (Thermo Fisher Scientific, Rodano, Italy), equipped with a heated electrospray ionization source (HESI). Diluted samples were injected into the flowing solvent (MeOH or ACN:H_2O, 90:10 (v/v) acidified with 0.1% formic acid) at a flow rate of 100 µL/min and delivered directly to the ESI source by a syringe pump at constant flow (20-50 µL/min). The tuning parameters adopted for the ESI source were: ion spray voltage 3.3 kV and tube lens voltage 60%. The ion transfer tube and the vaporizer temperature were maintained at 275 °C and 290 °C, respectively. Sheath and auxiliary gases were set at 40 and 10 arb (arbitrary unit), respectively. The mass accuracy of the recorded ions (*vs*. the calculated ones) was <5 mmu (milli-mass units). Analyses were run using full MS at 150-2000*m/z* in the positive ion mode. IR spectra were recorded on a Perkin Elmer BX FT-IR. Melting point analyses were carried out with a SMP3 Bibbi Stuart Scientific system. MR image was acquired on a Bruker Avance Neo 300 MHz spectrometer (7 T) equipped with a Micro 2.5 microimaging probe (Bruker BioSpin, Ettlingen, Germany). Inductively coupled plasma mass spectrometry (ICP-MS) (Element-2; Thermo-Finnigan, Rodano (MI), Italy) was used to measure the amount of boron in cell samples.

Procedures

2-(4-Hydroxybut-2-yn-1-yl)isoindoline-1,3-dione (2).[1]

In a dried round bottom flask under nitrogen atmosphere, but-2-yne-1,4-diol **1** (1.5 eq., 20 mmol, 1.2 g) was reacted with phthalimide (1.0 eq., 14 mmol, 2.0 g) and PPh_3 (1.0 eq., 14 mmol, 3.6 g) suspended in 80 mL of freshly distilled THF. The mixture was cooled to 0 °C and DIAD (1.0 eq., 14 mmol, 2.9 g, 2.8 mL) was added dropwise over a period of 1 hour, then the reaction was stirred overnight at room temperature. The solvent was evaporated under reduced pressure to obtain a crude yellow solid, purified on deactivated flash silica gel (PE:AcOEt 60:40) to yield **2** as a white solid (1.9 g, 64%). Spectroscopic data are consistent with those reported in literature.

^{1}H NMR (400 MHz; $CDCl_3$, Me_4Si) δ 7.91-7.84 (2H, m, Phth-*H*), 7.77-7.70 (2H, m, Phth-*H*), 4.49 (2H, t, *J* = 2.0 Hz, PhthNC*H*$_2$C≡C), 4.24 (2H, dt, *J* = 6.9, 1.9 Hz, HOC*H*$_2$C≡C), 1.62 (1H, t, *J* = 6.2 Hz, HOC*H*$_2$C≡C).

R_f: 0.42 (PE:AcOEt 60:40), UV, $KMnO_4$ and vanillin active.

4-(1,3-Dioxoisoindolin-2-yl)but-2-yn-1-yl methanesulfonate (3).

In a dried round bottom flask under nitrogen atmosphere, 2-(4-hydroxybut-2-yn-1-yl)isoindoline-1,3-dione **2** (1.0 eq., 9.0 mmol, 1.9 g) was dissolved in 80 mL of freshly distilled THF, then Et_3N (1.1 eq., 9.9 mmol, 1.0 g, 1.4 mL) was added dropwise. The mixture was cooled to 0 °C, then MsCl (1.1 eq., 9.9 mmol, 1.1 g, 0.77 mL) was added dropwise and the reaction was stirred at room temperature for 1 hour. The solvent was evaporated under reduced pressure and the crude was partitioned between AcOEt (50 mL) and H_2O (50 mL). The organic phase was separated and washed with H_2O (2 x 15 mL) and brine (1 x 15 mL), dried over anhydrous Na_2SO_4 and evaporated under reduced pressure affording a crude solid, which was purified on deactivated flash silica gel (PE:AcOEt 60:40) to yield **3** as a white solid (2.6 g, 98%). **3** can be used in the following step without further purifications.

^{1}H NMR (400 MHz; $CDCl_3$, Me_4Si) δ 7.91-7.84 (2H, m, Phth-*H*), 7.78-7.71 (2H, m, Phth-*H*), 4.83 (2H, s, MsOC*H*$_2$C≡C), 4.52 (2H, s, PhthNC*H*$_2$C≡C), 3.13 (3H, s, C*H*$_3$SO$_2$OCH$_2$).

^{13}C NMR (100 MHz, $CDCl_3$, Me_4Si) δ 167.0 (Cq), 134.5 (CH), 132.0 (Cq), 123.8 (CH), 83.7 (Cq), 75.6 (Cq), 57.6 (CH_2), 39.2 (CH_3), 27.2 (CH_2).

R_f: 0.71 (CH_2Cl_2:MeOH 99:1), UV and $KMnO_4$ active.

m.p.: 125.7-127.2 °C.

ν_{max} (neat)/cm^{-1} 3026, 2969, 1771, 1713, 1420, 1392, 1347, 1173, 1117, 939, 722.

ESI HRMS: $C_{13}H_{11}NO_5S$ calcd: 316.0250 $[M\text{-}Na]^+$, found: 316.0249 $[M\text{-}Na]^+$.

tert-Butyl(*tert*-butoxycarbonyl)(4-(1,3-dioxoisoindolin-2-yl)but-2-yn-1-yl)carbamate (4).

In a dried round bottom flask under nitrogen atmosphere, 4-(1,3-dioxoisoindolin-2-yl)but-2-yn-1-yl methanesulfonate **3** (1.0 eq., 6.4 mmol, 1.9 g), di-*tert*-butyl iminodicarboxylate (1.1 eq., 7.0 mmol, 1.5 g) and K_2CO_3 (3.0 eq., 19 mmol, 2.7 g) were suspended in 60 mL of anhydrous DMF. The mixture was stirred overnight at 60 °C then the solvent was evaporated under reduced pressure. The crude was partitioned between AcOEt (50 mL) and H_2O (50 mL). The organic phase was separated and washed with H_2O (1 x 20 mL) and brine (1 x 20 mL), dried over anhydrous Na_2SO_4 and evaporated under reduced pressure affording a crude, which was purified on deactivated flash silica gel (PE:AcOEt 85:15) to yield **4** as a white solid (1.5 g, 56%).

^{1}**H NMR** (400 MHz; $CDCl_3$, Me_4Si) *δ* 7.90-7.84 (2H, m, Phth-*H*), 7.76-7.71 (2H, m, Phth-*H*), 4.45 (2H, t, *J* = 2.0 Hz, PhthNC*H*$_2$C≡C), 4.32 (2H, t, *J* = 2.0 Hz, Boc_2NC*H*$_2$C≡C), 1.48 (18H, s, (OC(C*H*$_3$)$_3$)$_2$).

^{13}**C NMR** (100 MHz, $CDCl_3$, Me_4Si) *δ* 167.1 (Cq), 151.7 (Cq), 134.3 (CH), 132.2 (Cq), 123.6 (CH), 83.1 (Cq), 79.2 (Cq), 75.9 (Cq), 36.1 (CH_2), 28.2 (CH_3), 27.4 (CH_2).

R$_f$: 0.39 (PE:AcOEt 70:30), UV and $KMnO_4$ active.

m.p.: 110.5-111.3 °C.

***ν*$_{max}$** (neat)/cm^{-1} 3025, 2968, 2939, 1771, 1748, 1713, 1420, 1392, 1368, 1347, 1236, 1173, 1117, 939, 722.

ESI HRMS: $C_{22}H_{26}N_2O_6$ calcd: 437.1683 [M-Na]$^+$, found: 437.1679 [M-Na]$^+$.

General procedure for the ionic liquid-mediated dehydrogenative insertion reaction.[2]

In a dried heavy wall tube with screw cap under nitrogen atmosphere, the appropriate alkyne (1.0 eq.) and decaborane (1.2 eq.) were suspended in anhydrous toluene (5 mL/mmol). Then, bmim$^+$Cl$^-$ (0.60 eq.) was added and the mixture was vigorously stirred at 110 °C for 1.5 hours. After cooling to room temperature, the solvent was evaporated under reduced pressure and the crude was purified on deactivated flash silica gel.

C-(*N*-(1,3-dioxoisoindolin-2-yl))methyl-*C'*-2-(*N*,*N*-di-*tert*-butoxycarbonyl)aminomethyl-*o*-carborane (5).

According to the described general procedure, *tert*-butyl(*tert*-butoxycarbonyl)(4-(1,3-dioxoisoindolin-2-yl)but-2-yn-1-yl)carbamate **4** (1.50 mmol, 0.62 g), decaborane (1.80 mmol, 0.22 g) and bmim$^+$Cl$^-$ (0.90 mmol, 0.16 g) were reacted at 110 °C in 7,5 mL of anhydrous toluene to obtain a crude oil, purified on deactivated flash silica gel (PE:AcOEt 85:15 or CH_2Cl_2 100%) to yield **5** as a white solid (0.33 g, 41%).

^{1}**H NMR** (400 MHz; $CDCl_3$, Me_4Si) *δ* 7.92-7.87 (2H, m, Phth-*H*), 7.81-7.75 (2H, m, Phth-*H*), 4.90 (2H, s, PhthNC*H*$_2$), 4.79 (2H, s, Boc_2NC*H*$_2$), 3.10-1.35 (10H, br, B-*H*), 1.55 (18H, s, (OC(C*H*$_3$)$_3$)$_2$).

^{13}**C NMR** (100 MHz, $CDCl_3$, Me_4Si) *δ* 167.5 (Cq), 152.5 (Cq), 134.8 (CH), 131.6 (Cq), 124.1 (CH), 84.2 (Cq), 81.1 (Cq), 79.9 (Cq), 47.5 (CH_2), 40.3 (CH_2), 28.1 (CH_3).

^{11}**B NMR** (192.5 MHz, $CDCl_3$, Me_4Si) *δ* -5.02, -9.67, -11.44, -14.04.

R$_f$: 0.53 (PE:AcOEt 70:30), UV and $PdCl_2$ active.

m.p.: 186-187 °C (degradation, bubbles).

***ν*$_{max}$** (neat)/cm^{-1} 2981, 2935, 2577, 1778, 1722, 1698, 1416, 1392, 1368, 1354, 1338, 1233, 1126, 909, 855, 731, 717.

ESI HRMS: $C_{22}H_{36}B_{10}N_2O_6$ calcd: 555.3469 [M-Na]$^+$, found: 555.3465 [M-Na]$^+$.

^{10}B-enriched-*C*-(*N*-(1,3-dioxoisoindolin-2-yl))methyl-*C'*-2-(*N*,*N*-di-*tert*-butoxycarbonyl)aminomethyl-*o*-carborane (^{10}B-5).

According to the described general procedure, *tert*-butyl(*tert*-butoxycarbonyl)(4-(1,3-dioxoisoindolin-2-yl)but-2-yn-1-yl)carbamate **4** (1.43 mmol, 0.59 g), ^{10}B-enriched-decaborane (1.71 mmol, 0.20 g) and bmim$^+$Cl$^-$ (0.86 mmol, 0.15 g) were reacted at 110 °C in 7,5 mL of anhydrous toluene to obtain a crude oil, purified on deactivated flash silica gel (PE:AcOEt 85:15 or CH_2Cl_2 100%) to yield **^{10}B-5** as a white solid (0.31 g, 42%).

^{1}**H NMR** (400 MHz; $CDCl_3$, Me_4Si) *δ* 7.92-7.87 (2H, m, Phth-*H*), 7.81-7.75 (2H, m, Phth-*H*), 4.90 (2H, s, PhthNC*H*$_2$), 4.79 (2H, s, Boc_2NC*H*$_2$), 2.75-1.70 (10H, br, B-*H*), 1.55 (18H, s, (OC(C*H*$_3$)$_3$)$_2$).

^{13}**C NMR** (100 MHz, $CDCl_3$, Me_4Si) *δ* 167.5 (Cq), 152.5 (Cq), 134.8 (CH), 131.6 (Cq), 124.0 (CH), 84.2 (Cq), 81.2 (Cq), 79.9 (Cq), 47.5 (CH_2), 40.3 (CH_2), 28.2 (CH_3).
R$_f$: 0.53 (PE:AcOEt 70:30), UV and $PdCl_2$ active.
m.p.: 185-187 °C (degradation, bubbles).
***ν*$_{max}$** (neat)/cm^{-1} 2981, 2936, 2578, 1778, 1721, 1699, 1415, 1392, 1368, 1355, 1338, 1233, 1126, 908, 855, 730, 717.
ESI HRMS: $C_{22}H_{36}{}^{10}B_{10}N_2O_6$ calcd: 547.3759 [M-Na]$^+$, found: 547.3755 [M-Na]$^+$.

General procedure for the Boc group removal.

In a round bottom flask, the appropriate Boc protected amine (1.0 eq.) was dissolved in CH_2Cl_2, then an excess of trifluoroacetic acid (TFA) was added dropwise. The solution was stirred at room temperature for 2 hours. The solvents were evaporated under reduced pressure to afford the desired ammonium salt, which was used without further purifications.

_C_-(_N_-(1,3-dioxoisoindolin-2-yl))methyl-_C′_-2-aminomethyl-_o_-carborane trifluoroacetic acid salt (6•TFA).

According to the described general procedure, *C*-(*N*-(1,3-dioxoisoindolin-2-yl))methyl-*C′*-2-(*N*,*N*-di-*tert*-butoxycarbonyl)aminomethyl-*o*-carborane **5** (1.0 mmol, 0.54 g) was reacted with 2.5 mL of TFA in 5.0 mL of CH_2Cl_2 to afford **6•TFA** as a white solid (0.45 g, 99%), which was used without further purifications. Due to the instability of **6•TFA**, the spectroscopic characterization was carried out on the hydrochloric acid salt. *C*-(*N*-(1,3-dioxoisoindolin-2-yl))methyl-*C′*-2-(*N*,*N*-di-*tert*-butoxycarbonyl)aminomethyl-*o*-carborane **5** (0.056 mmol, 0.030 g) was dissolved in 4 mL of 1,4-dioxane, then 2 mL of 37% v/v HCl were added dropwise. The mixture was stirred at room temperature until completion, then the solvents were removed under reduced pressure. The solid was taken up with CH_2Cl_2 and filtered affording *C*-(*N*-(1,3-dioxoisoindolin-2-yl))methyl-*C′*-2-aminomethyl-*o*-carborane hydrochloric acid salt **6•HCl** as a white solid (0.018 g, 85%).

^{1}**H NMR** (400 MHz; CD_3OD) *δ* 7.99-7.93 (2H, m, Phth-*H*), 7.93-7.88 (2H, m, Phth-*H*), 4.60 (2H, s, PhthNC*H*$_2$), 4.38 (2H, s, $^+H_3NCH_2$), 3.10-1.40 (10H, br, B-*H*).

^{13}**C NMR** (100 MHz, CD_3OD) *δ* 169.1 (Cq), 136.4 (CH), 132.6 (Cq), 125.0 (CH), 81.3 (Cq), 77.0 (Cq), 42.9 (CH_2), 41.3 (CH_2).

^{11}**B NMR** (128 MHz, CD_3OD) *δ* -3.43, -8.77, -9.70, -11.20, -12.33.

Rf: 0.10 (CH_2Cl_2:MeOH 95:5), UV and $PdCl_2$ active.

m.p.: 227-229 °C (degradation, brown solid).

***ν*$_{max}$** (neat)/cm^{-1} 2970, 2588, 1776, 1718, 1399, 1368, 1357, 1133, 717.

ESI HRMS: $C_{12}H_{20}B_{10}N_2O_2$ calcd: 333.2601 [M-H]$^+$, found: 333.2598 [M-H]$^+$.

^{10}B-enriched-_C_-(_N_-(1,3-dioxoisoindolin-2-yl))methyl-_C′_-2-aminomethyl-_o_-carborane trifluoroacetic acid salt (^{10}B-6•TFA).

According to the described general procedure, ^{10}B-enriched-*C*-(*N*-(1,3-dioxoisoindolin-2-yl))methyl-*C′*-2-(*N*,*N*-di-*tert*-butoxycarbonyl)aminomethyl-*o*-carborane **^{10}B-5** (1.0 mmol, 0.54 g) was reacted with 2.5 mL of TFA in 5.0 mL of CH_2Cl_2 to afford **^{10}B-6•TFA** as a white solid (0.45 g, 99%), which was used without further purifications. Due to the instability of **^{10}B-6•TFA**, the spectroscopic characterization was carried out on the hydrochloric acid salt. *C*-(*N*-(1,3-dioxoisoindolin-2-yl))methyl-*C′*-2-(*N*,*N*-di-*tert*-butoxycarbonyl)aminomethyl-*o*-carborane **^{10}B-5** (0.056 mmol, 0.030 g) was dissolved in 4 mL of 1,4-dioxane, then 2 mL of 37% v/v HCl were added dropwise. The mixture was stirred at room temperature until

completion, then the solvents were removed under reduced pressure. The solid was taken up with CH_2Cl_2 and filtered affording *C*-(*N*-(1,3-dioxoisoindolin-2-yl))methyl-*C′*-2-aminomethyl-*o*-carborane hydrochloric acid salt **^{10}B-6•HCl** as a white solid (0.018 g, 85%).

^{1}H NMR (400 MHz; CD_3OD) δ 7.99-7.93 (2H, m, Phth-*H*), 7.93-7.88 (2H, m, Phth-*H*), 4.60 (2H, s, PhthNC*H*$_2$), 4.38 (2H, s, $^+H_3NCH_2$), 2.89-1.67 (10H, br, B-*H*).

^{13}C NMR (100 MHz, CD_3OD) δ 169.1 (Cq), 136.3 (CH), 132.6 (Cq), 125.0 (CH), 81.3 (Cq), 77.0 (Cq), 42.9 (CH_2), 41.3 (CH_2).

R$_f$: 0.10 (CH_2Cl_2:MeOH 95:5), UV and $PdCl_2$ active.

m.p.: 227-229 °C (degradation, brown solid).

***ν*$_{max}$** (neat)/cm^{-1} 2971, 2586, 1776, 1717, 1398, 1368, 1355, 1133, 717.

ESI HRMS: $C_{12}H_{20}{}^{10}B_{10}N_2O_2$ calcd: 325.2891 [M-H]$^+$, found: 325.2887 [M-H]$^+$.

General procedure for the sulfamidic group installation.[3]

In a dried round bottom flask under nitrogen atmosphere, the appropriate *C*-aminomethyl-*o*-carborane (1.0 eq.) was suspended in anhydrous 1,4-dioxane. Sulfamide (5.0 eq.) was added and the mixture was stirred at 110 °C until the total disappearance of the reagent spot on TLC plate was observed (about 2 hours). The solvent was evaporated under reduced pressure and the crude was dissolved in AcOEt (20 mL) and 10% aqueous $KHSO_4$ (20 mL). The organic phase was separated and the aqueous layer was extracted with AcOEt (2 x 10 mL). The combined organic phases were dried over anhydrous Na_2SO_4 and evaporated under reduced pressure. The crude was then purified on flash silica gel.

C-(*N*-(1,3-dioxoisoindolin-2-yl))methyl-*C′*-(sulfamoylamino)methyl-*o*-carborane (7).

C-(*N*-(1,3-dioxoisoindolin-2-yl))methyl-*C′*-2-aminomethyl-*o*-carborane trifluoroacetic acid salt **6•TFA** (1.0 eq., 0.18 mmol, 0.080 g) was partitioned between CH_2Cl_2 (20 mL) and 10% aqueous Na_2CO_3 (20 mL) and transferred to an extraction funnel. The organic phases was separated and washed with 10% aqueous Na_2CO_3 (2 x 10 mL), brine (1 x 10 mL), dried over anhydrous Na_2SO_4 and evaporated under reduced pressure affording *C*-(*N*-(1,3-dioxoisoindolin-2-yl))methyl-*C′*-2-aminomethyl-*o*-carborane **6** (0.061 g, 99%) as a white solid. According to the described general procedure, *C*-(*N*-(1,3-dioxoisoindolin-2-yl))methyl-*C′*-2-aminomethyl-*o*-carborane **6** (0.18 mmol, 0.061 g) was reacted with sulfamide (0.92 mmol, 0.088 g) in 10 mL of anhydrous 1,4-dioxane. The crude solid obtained after extraction was purified on flash silica gel (CH_2Cl_2:MeOH 98:2) to yield **7** as a white solid (0.070 g, 93%).

^{1}H NMR (400 MHz; acetone-*d6*) δ 8.01-7.89 (4H, m, Phth-*H*), 6.76 (1H, t, *J* = 7.5 Hz, N*H*SO$_2$NH$_2$), 6.28 (2H, s, NHSO$_2$N*H*$_2$), 4.62 (2H, s, PhthNC*H*$_2$), 4.34 (2H, d, *J* = 7.6 Hz, C*H*$_2$NHSO$_2$NH$_2$), 3.25-1.40 (10H, br, B-*H*).

^{13}C NMR (100 MHz, acetone-*d6*) δ 168.1 (Cq), 135.9 (CH), 132.5 (Cq), 124.6 (CH), 82.1 (Cq), 79.1 (Cq), 47.1 (CH_2), 40.9 (CH_2).

^{11}B NMR (192.5 MHz, acetone-*d6*) δ -5.03, -5.61, -10.76, -11.47, -12.85.

R$_f$: 0.44 (CH_2Cl_2:MeOH 95:5), UV and $PdCl_2$ active.

m.p.: 78.2-80.4 °C.

***ν*$_{max}$** (neat)/cm^{-1} 3278, 2924, 2581, 1776, 1716, 1397, 1355, 1159, 1130, 717.

ESI HRMS: $C_{12}H_{21}B_{10}N_3O_4S$ calcd: 412.2329 [M-H]$^+$, found: 412.2327 [M-H]$^+$.

^{10}B-enriched-*C*-(*N*-(1,3-dioxoisoindolin-2-yl))methyl-*C′*-(sulfamoylamino)methyl-*o*-carborane (^{10}B-7).

^{10}B-enriched-*C*-(*N*-(1,3-dioxoisoindolin-2-yl))methyl-*C′*-2-aminomethyl-*o*-carborane trifluoroacetic acid salt ^{10}B-6•TFA (1.0 eq., 0.19 mmol, 0.10 g) was partitioned between CH_2Cl_2 (20 mL) and 10% aqueous Na_2CO_3 (20 mL) and transferred to an extraction funnel. The organic phases was separated and washed with 10% aqueous Na_2CO_3 (2 x 10 mL), brine (1 x 10 mL), dried over anhydrous Na_2SO_4 and evaporated under reduced pressure affording ^{10}B-enriched-*C*-(*N*-(1,3-dioxoisoindolin-2-yl))methyl-*C′*-2-aminomethyl-*o*-carborane **^{10}B-6** (0.058 g, 95%) as a white solid. According to the described general procedure, ^{10}B-enriched-*C*-(*N*-(1,3-dioxoisoindolin-2-yl))methyl-*C′*-2-aminomethyl-*o*-carborane **^{10}B-6** (0.18 mmol, 0.058 g) was reacted with sulfamide (0.90 mmol, 0.086 g) in 10 mL of anhydrous 1,4-dioxane. The crude solid obtained after extraction was purified on flash silica gel (CH_2Cl_2:MeOH 98:2) to yield **^{10}B-7** as a white solid (0.067 g, 92%).

^{1}H NMR (400 MHz; $CDCl_3$, Me_4Si) δ 7.94-7.89 (2H, m, Phth-*H*), 7.84-7.79 (2H, m, Phth-*H*), 5.61 (1H, t, *J* = 7.4 Hz, N*H*SO_2NH_2), 4.85 (2H, s, $NHSO_2NH_2$), 4.47 (2H, s, PhthNC*H*$_2$), 4.27 (2H, d, *J* = 7.4 Hz, C*H*$_2$$NHSO_2NH_2$), 3.08-1.45 (10H, br, B-*H*).

^{13}C NMR (100 MHz, acetone-*d6*) δ 168.2 (Cq), 135.9 (CH), 132.5 (Cq), 124.6 (CH), 82.1 (Cq), 79.1 (Cq), 47.1 (CH_2), 40.9 (CH_2).

R$_f$: 0.44 (CH_2Cl_2:MeOH 95:5), UV and $PdCl_2$ active.

m.p.: 78.0-79.6 °C.

***ν*$_{max}$** (neat)/cm^{-1} 3281, 2924, 2580, 1776, 1715, 1398, 1355, 1160, 1131, 717.

ESI HRMS: $C_{12}H_{21}{}^{10}B_{10}N_3O_4S$ calcd: 404.2619 [M-H]$^+$, found: 404.2614 [M-H]$^+$.

General procedure for the phthaloyl group removal.[4]

In a round bottom flask, the appropriate *C*-(*N*-(1,3-dioxoisoindolin-2-yl))methyl-*o*-carborane (1.0 eq.) was suspended in a mixture of *i*-PrOH:H_2O 4:1. $NaBH_4$ (5.0 eq.) was added and the reaction was stirred at room temperature until the total disappearance of the reagent spot on TLC plate was observed (about 2 hours). The solvent was removed under reduced pressure, then the crude was partitioned in H_2O (20 mL) and AcOEt (20 mL). The organic phase was separated and the aqueous layer was extracted with AcOEt (2 x 10 mL). The combined organic phases were dried over anhydrous Na_2SO_4 and evaporated under reduced pressure. The crude was then dissolved in a glacial AcOH:H_2O:37% v/v HCl 4:1:1 mixture and heated at 50 °C for 2 hours. The solvent was removed under reduced pressure and the residue was sonicated with few mL of $CHCl_3$. The product was recovered by filtration and washing with few mL of $CHCl_3$.

C-aminomethyl-*C′*-(sulfamoylamino)methyl-*o*-carborane hydrochloric salt (8).

According to the described general procedure, *C*-(*N*-(1,3-dioxoisoindolin-2-yl))methyl-C′-(sulfamoylamino)methyl-o-carborane **7** (0.25 mmol, 0.10 g) was reacted with $NaBH_4$ (1.2 mmol, 0.046 g) in 10 mL of *i*-PrOH:H_2O 4:1. The crude solid obtained after extraction was dissolved in 12 mL of a glacial AcOH:H_2O:37% v/v HCl 4:1:1 mixture. The treatment with $CHCl_3$ afforded **8** as a white solid (0.063 g, 81%).

^{1}H NMR (400 MHz; CD_3OD) δ 4.03 (2H, s, $^+H_3NCH_2$), 3.94 (2H, s, C*H*$_2$$NHSO_2NH_2$), 3.10-1.45 (10H, br, B-*H*).

^{13}C NMR (100 MHz, CD_3OD) δ 82.0 (Cq), 75.2 (Cq), 47.5 (CH_2), 42.7 (CH_2).

^{11}B NMR (192.5 MHz, CD_3OD) δ -3.85, -5.22, -10.70, -12.16.

R$_f$: 0.17 (CH_2Cl_2:MeOH 88:12), $PdCl_2$ active.

m.p.: 187-188 °C (degradation, yellow solid).

***ν*$_{max}$** (neat)/cm^{-1} 3172, 2970, 2592, 1341, 1159, 914.

ESI HRMS: $C_4H_{19}B_{10}N_3O_2S$ calcd: 282.2274 [M-H]$^+$, found: 282.2271 [M-H]$^+$.

^{10}B-enriched-*C*-aminomethyl-*C′*-(sulfamoylamino)methyl-*o*-carborane hydrochloric salt (^{10}B-8).

According to the described general procedure, *C*-(*N*-(1,3-dioxoisoindolin-2-yl))methyl-C′-(sulfamoylamino)methyl-o-carborane **^{10}B-7** (0.17 mmol, 0.069 g) was reacted with $NaBH_4$ (0.86 mmol, 0.032 g) in 10 mL of *i*-PrOH:H_2O 4:1. The crude solid obtained after extraction was dissolved in 12 mL of a glacial AcOH:H_2O:37% v/v HCl 4:1:1 mixture. The treatment with $CHCl_3$ afforded **^{10}B-8** as a white solid (0.048 g, 90%).

^{1}H NMR (400 MHz; CD_3OD) δ 4.03 (2H, s, $^+H_3NCH_2$), 3.95 (2H, s, $CH_2NHSO_2NH_2$), 3.10-1.45 (10H, br, B-*H*).

^{13}C NMR (100 MHz, CD_3OD) δ 82.0 (Cq), 75.2 (Cq), 47.5 (CH_2), 42.7 (CH_2).

R_f: 0.17 (CH_2Cl_2:MeOH 88:12), $PdCl_2$ active.

m.p.: 186-188 °C (degradation, yellow solid).

***ν*$_{max}$** (neat)/cm^{-1} 3170, 2972, 2588, 1341, 1160, 914.

ESI HRMS: $C_4H_{19}{}^{10}B_{10}N_3O_2S$ calcd: 274.2564 [M-H]$^+$, found: 274.2561 [M-H]$^+$.

General procedure for the amide formation.[5]

In a dried 25 mL three necked round bottom flask under nitrogen atmosphere, the appropriate acid (1.10 eq.), and CDMT (1.00 eq.) were dissolved in freshly distilled CH_2Cl_2. The mixture was cooled to 0 °C then NMM (1.10 eq) was added dropwise. The reaction was stirred at 0 °C until the total disappearance of CDMT spot on TLC was observed (PE:Et_2O 50:50). In a second dried 25 mL round bottom flask under nitrogen atmosphere, the appropriate C-aminomethyl-C′-(sulfamoylamino)methyl-o-carborane hydrochloric salt (1.00 eq.) was suspended in freshly distilled CH_2Cl_2, then NMM (1.10 eq.) was added dropwise. The mixture was stirred at room temperature for 15 minutes, then added dropwise to the other solution at 0 °C. The reaction was stirred at 0 °C for 1 hour then to room temperature for 24 hours, then quenched with H_2O. The volatiles were removed under reduced pressure, then the crude was dissolved in $CHCl_3$ and washed with H_2O (2 x 10 mL). The organic phase was washed with 10% aqueous citric acid (2 x 10 mL) and back extracted with $CHCl_3$ (5 x 10 mL). The combined organic phase were then washed with brine (1 x 10 mL), saturated aqueous $NaHCO_3$ (2 x 10 mL), brine (1 x 10 mL), dried over anhydrous Na_2SO_4 and evaporated under reduced pressure. The crude was purified on flash silica gel.

***C*-(6-(2-(4,7,10-tris(2-(*tert*-butoxy)-2-oxoethyl)-1,4,7,10-tetraazacyclododecan-1-yl)acetamido)hexanamido)methyl-*C′*-(sulfamoylamino)methyl-*o*-carborane (9).**

According to the described general procedure, 6-(2-(4,7,10-tris(2-(*tert*-butoxy)-2-oxoethyl)-1,4,7,10-tetraazacyclododecan-1-yl)acetamido)hexanoic acid (0.15 mmol, 0.10 g), and CDMT (0.14 mmol, 0.024 g) were reacted with NMM (0.14 mmol, 0.015 g, 0.017 mL) in 3 mL of freshly distilled CH_2Cl_2. *C*-aminomethyl-*C′*-(sulfamoylamino)methyl-*o*-carborane hydrochloric salt **8** (0.14 mmol, 0.044 g) and NMM (0.15 mmol, 0.015 g, 0.017 mL) in 3 mL of freshly distilled CH_2Cl_2 were added dropwise to the other solution. The crude obtained after the aqueous work-up was purified on flash silica gel (CH_2Cl_2:MeOH 92:8) to yield **9** as a white solid (0.064 g, 49%).

^{1}H NMR (600 MHz; $CDCl_3$, Me_4Si) δ 8.58 (1H, t, *J* = 6.7 Hz, C(=O)N*H*CH_2C_{cage}), 7.71 (1H, t, *J* = 6.7 Hz, C(=O)N*H*CH_2CH_2), 7.01 (1H, t, *J* = 4.6 Hz, CH_2N*H*SO_2NH_2), 5.71 (2H, s, $CH_2NHSO_2NH_2$), 4.11 (2H, s, C(=O)NHCH_2C_{cage}), 4.00 (2H, d, *J* = 4.6 Hz, $CH_2NHSO_2NH_2$), 3.28-3.22 (2H, m, NHCH_2CH_2), 3.65-1.60 (34H, br, B-*H* and NCH_2C(=O)O*t*-Bu and NCH_2C(=O)NH and NCH_2CH_2N), 2.34 (2H, t, *J* = 7.1 Hz, CH_2CH_2C(=O)NH),

1.75-1.68 (2H, m, $NHCH_2CH_2CH_2CH_2CH_2C(=O)$), 1.60-1.53 (2H, m, $NHCH_2CH_2CH_2CH_2CH_2C(=O)$), 1.46 (9H, s, $OC(CH_3)_3$), 1.45 (18H, s, $(OC(CH_3)_3)_2$), 1.43-1.36 (2H, m, $NHCH_2CH_2CH_2CH_2CH_2C(=O)$).

^{13}C NMR (150 MHz, $CDCl_3$, Me_4Si) δ 174.2 (Cq), 172.5 (Cq), 171.6 (Cq), 82.1 (Cq), 82.0 (Cq), 81.2 (Cq), 79.0 (Cq), 56.3 (Cq), 55.9 (Cq), 55.7 (br, CH_2), 46.2 (CH_2), 41.6 (CH_2), 38.9 (CH_2), 36.1 (CH_2), 28.2 (CH_3), 28.1 (CH_3), 28.0 (CH_2), 26.2 (CH_2), 24.9 (CH_2).

^{11}B NMR (192.5 MHz, $CDCl_3$, Me_4Si) δ -4.46, -11.04.

R_f: 0.15 (CH_2Cl_2:MeOH 90:10), Ce-Mo, ninhydrin and $PdCl_2$ active.

m.p.: 122-124 °C (degradation, yellow gummy solid).

$\boldsymbol{\nu}_{max}$ (neat)/cm^{-1} 3231, 2972, 2578, 1726, 1675, 1546, 1450, 1367, 1304, 1227, 1161, 755.

ESI HRMS: $C_{38}H_{80}B_{10}N_8O_{10}S$ calcd: 949.6794 $[M-H]^+$, found: 949.6794 $[M-H]^+$.

^{10}B-enriched-*C*-(6-(2-(4,7,10-tris(2-(*tert*-butoxy)-2-oxoethyl)-1,4,7,10-tetraazacyclododecan-1-yl)acetamido)hexanamido)methyl-*C′*-(sulfamoylamino)methyl-*o*-carborane (^{10}B-9).

According to the described general procedure, 6-(2-(4,7,10-tris(2-(*tert*-butoxy)-2-oxoethyl)-1,4,7,10-tetraazacyclododecan-1-yl)acetamido)hexanoic acid (0.17 mmol, 0.12 g), and CDMT (0.15 mmol, 0.027 g) were reacted with NMM (0.17 mmol, 0.017 g, 0.019 mL) in 3 mL of freshly distilled CH_2Cl_2. ^{10}B-enriched-*C*-aminomethyl-*C′*-(sulfamoylamino)methyl-*o*-carborane hydrochloric salt **^{10}B-8** (0.15 mmol, 0.048 g) and NMM (0.17 mmol, 0.017 g, 0.019 mL) in 3 mL of freshly distilled CH_2Cl_2 were added dropwise to the other solution. The crude obtained after the aqueous work-up was purified on flash silica gel (CH_2Cl_2:MeOH 92:8) to yield **^{10}B-9** as a white solid (0.075 g, 53%).

^{1}H NMR (600 MHz; $CDCl_3$, Me_4Si) δ 8.65 (1H, t, J = 6.6 Hz, $C(=O)NHCH_2C_{cage}$), 7.79 (1H, t, J = 5.4 Hz, $C(=O)NHCH_2CH_2$), 7.08 (1H, t, J = 7.2 Hz, $CH_2NHSO_2NH_2$), 5.62 (2H, s, $CH_2NHSO_2NH_2$), 4.11 (2H, brs, $C(=O)NHCH_2C_{cage}$), 3.99 (2H, d, J = 7.1 Hz, $CH_2NHSO_2NH_2$), 3.28-3.22 (2H, m, $NHCH_2CH_2$), 3.65-1.60 (34H, br, B-*H* and $NCH_2C(=O)Ot$-Bu and $NCH_2C(=O)NH$ and NCH_2CH_2N), 2.36 (2H, t, J = 6.8 Hz, $CH_2CH_2C(=O)NH$), 1.76-1.69 (2H, m, $NHCH_2CH_2CH_2CH_2CH_2C(=O)$), 1.61-1.54 (2H, m, $NHCH_2CH_2CH_2CH_2CH_2C(=O)$), 1.46 (9H, s, $OC(CH_3)_3$), 1.45 (18H, s, $(OC(CH_3)_3)_2$), 1.43-1.37 (2H, m, $NHCH_2CH_2CH_2CH_2CH_2C(=O)$).

^{13}C NMR (150 MHz, $CDCl_3$, Me_4Si) δ 174.3 (Cq), 172.5 (Cq), 171.7 (Cq), 82.1 (Cq), 82.0 (Cq), 81.3 (Cq), 79.0 (Cq), 56.3 (Cq), 55.9 (Cq), 55.7 (br, CH_2), 46.2 (CH_2), 41.5 (CH_2), 38.8 (CH_2), 36.2 (CH_2), 28.2 (CH_3), 28.1 (CH_3), 27.9 (CH_2), 26.1 (CH_2), 24.9 (CH_2).

R_f: 0.15 (CH_2Cl_2:MeOH 90:10), Ce-Mo, ninhydrin and $PdCl_2$ active.

m.p.: 123-124 °C (degradation, yellow gummy solid).

$\boldsymbol{\nu}_{max}$ (neat)/cm^{-1} 3231, 2970, 2581, 1726, 1673, 1545, 1450, 1367, 1305, 1227, 1161, 756.

ESI HRMS: $C_{38}H_{80}{}^{10}B_{10}N_8O_{10}S$ calcd: 941.7085 $[M-H]^+$, found: 941.7076 $[M-H]^+$.

General procedure for *tert*-butyl esters hydrolysis.

In a 8 mL screw cap vial, the appropriate *tert*-butyl ester (1.0 eq.) was dissolved in CH_2Cl_2, then a solution of 95% v/v trifluoroacetic acid and 5% v/v H_2O was added dropwise. The mixture was stirred at room temperature for 24 hours. The solvent was evaporated under a nitrogen flux through a needle, then an excess of pure trifluoroacetic acid was added and the mixture was stirred for additional 24 hours. The solvent was evaporated under a nitrogen flux through a needle, then Et_2O was added and the suspension was sonicated. The precipitate was filtered and washed with Et_2O to yield the desired carboxylic acid, which was

used without further purifications. Alternatively, the products can be purified on C_{18}-Reversed phase silica gel (fully end-capped) using ACN:H_2O 80:20 as the eluent.

_C_-(6-(2-(4,7,10-tris-carboxymethyl-1,4,7,10-tetraazacyclododecan-1-yl)acetamido)hexanamido)methyl-_C′_-(sulfamoylamino)methyl-_o_-carborane (10).

According to the described general procedure, *C*-(6-(2-(4,7,10-tris(2-(*tert*-butoxy)-2-oxoethyl)-1,4,7,10-tetraazacyclododecan-1-yl)acetamido)hexanamido)methyl-*C′*-(sulfamoylamino)methyl-*o*-carborane **9** (0.067 mmol, 0.063 g) was reacted with 0.50 mL of a 95:5 %v/v trifluoroacetic acid:H_2O solution in 0.50 mL of CH_2Cl_2. The solvent was evaporated and 0.50 mL of trifluoroacetic acid were added. Evaporation of the solvent and treatment with Et_2O yielded **10** as a white solid (0.052 g, quantitative), which was used without further purifications. In addition, the purification on C_{18}-Reversed phase silica gel (fully end-capped) using ACN:H_2O 80:20 as the eluent (R_f = 0.24) afforded **10** as a white solid (0.049 g).

^{1}H NMR (400 MHz; CD_3OD) δ 4.08 (2H, s, C(=O)NHC*H*$_2$C$_{cage}$), 3.99 (2H, s, C*H*$_2$NHSO$_2$NH$_2$), 3.88-3.65 (8H, brm, NC*H*$_2$COOH and NC*H*$_2$C(=O)NH), 3.42-3.10 (18H, brm, NC*H*$_2$C*H*$_2$N2H and NHC*H*$_2$C*H*$_2$), 2.85-1.40 (10H, br, B-*H*), 2.27 (2H, t, *J* = 7.2 Hz, CH$_2$C*H*$_2$C(=O)NH), 1.69-1.60 (2H, m, NHCH$_2$C*H*$_2$CH$_2$CH$_2$CH$_2$C(=O)), 1.60-1.49 (2H, m, NHCH$_2$CH$_2$CH$_2$C*H*$_2$CH$_2$C(=O)), 1.44-1.34 (2H, m, NHCH$_2$CH$_2$C*H*$_2$CH$_2$CH$_2$C(=O)).

^{13}C NMR (100 MHz, CD_3OD) δ 176.2 (Cq), 163.1 (Cq), 162.7 (Cq), 119.7 (Cq), 116.8 (Cq), 81.5 (Cq), 81.5 (Cq), 56.1 (CH_2), 55.6-54.9 (br, CH_2), 51.6-50.7 (br, CH_2), 47.1 (CH_2), 42.5 (CH_2), 40.2 (CH_2), 36.4 (CH_2), 29.7 (CH_2), 27.4 (CH_2), 26.2 (CH_2).

^{11}B NMR (192.5 MHz, CD_3OD) δ -5.39, -11.03.

m.p.: 134-135 °C (degradation, yellow gummy solid).

***ν*$_{max}$** (neat)/cm^{-1} 3090, 2944, 2871, 2581, 1670, 1546, 1458, 1353, 1200, 1163, 721.

ESI HRMS: $C_{26}H_{56}B_{10}N_8O_{10}S$ calcd: 781.4916 [M-H]$^+$, found: 781.4911 [M-H]$^+$.

^{10}B-enriched-_C_-(6-(2-(4,7,10-tris-carboxymethyl-1,4,7,10-tetraazacyclododecan-1-yl)acetamido)hexanamido)methyl-_C′_-(sulfamoylamino)methyl-_o_-carborane (^{10}B-10).

According to the described general procedure, ^{10}B-enriched-*C*-(6-(2-(4,7,10-tris(2-(*tert*-butoxy)-2-oxoethyl)-1,4,7,10-tetraazacyclododecan-1-yl)acetamido)hexanamido)methyl-*C′*-(sulfamoylamino)methyl-*o*-carborane **^{10}B-9** (0.084 mmol, 0.079 g) was reacted with 0.50 mL of a 95:5 %v/v trifluoroacetic acid:H_2O solution in 0.50 mL of CH_2Cl_2. The solvent was evaporated and 0.50 mL of trifluoroacetic acid were added. Evaporation of the solvent and treatment with Et_2O yielded **^{10}B-10** as a white solid (0.065 g, quantitative), which was used without further purifications. In addition, the purification on C_{18}-Reversed phase silica gel (fully end-capped) using ACN:H_2O 80:20 as the eluent (R_f = 0.24) afforded **^{10}B-10** as a white solid (0.061 g).

^{1}H NMR (400 MHz; CD_3OD) δ 4.08 (2H, s, C(=O)NHC*H*$_2$C$_{cage}$), 3.99 (2H, s, C*H*$_2$NHSO$_2$NH$_2$), 3.88-3.64 (8H, brm, NC*H*$_2$COOH and NC*H*$_2$C(=O)NH), 3.39-3.14 (18H, brm, NC*H*$_2$C*H*$_2$N2H and NHC*H*$_2$C*H*$_2$), 2.70-1.40 (10H, br, B-*H*), 2.27 (2H, t, *J* = 7.3 Hz, CH$_2$C*H*$_2$C(=O)NH), 1.70-1.60 (2H, m, NHCH$_2$C*H*$_2$CH$_2$CH$_2$CH$_2$C(=O)), 1.60-1.50 (2H, m, NHCH$_2$CH$_2$CH$_2$C*H*$_2$CH$_2$C(=O)), 1.44-1.34 (2H, m, NHCH$_2$CH$_2$C*H*$_2$CH$_2$CH$_2$C(=O)).

^{13}C NMR (100 MHz, CD_3OD) δ 176.2 (Cq), 163.1 (Cq), 162.8 (Cq), 119.7 (Cq), 116.8 (Cq), 81.5 (Cq), 81.5 (Cq), 56.1 (CH_2), 55.6-55.1 (br, CH_2), 51.6-50.7 (br, CH_2), 47.2 (CH_2), 42.6 (CH_2), 40.2 (CH_2), 36.4 (CH_2), 29.7 (CH_2), 27.4 (CH_2), 26.2 (CH_2).

m.p.: 135-136 °C (degradation, yellow gummy solid).

***ν*$_{max}$** (neat)/cm^{-1} 3096, 2940, 2872, 2579, 1672, 1545, 1458, 1352, 1198, 1161, 721.

ESI HRMS: $C_{26}H_{56}{}^{10}B_{10}N_8O_{10}S$ calcd: 773.5207 [M-H]$^+$, found: 773.5201 [M-H]$^+$.

Biology

Gd-B-CA-SF complexation

The B-CA-SF or ^{10}B-CA-SF ligand was dissolved in H_2O at a concentration of 1 mM and was titrated with increasing concentration of $GdCl_3$ (natural abundant or 90% enriched in **^{157}Gd**) to obtain all the possible combinations of both enriched and natural occurring compounds: Gd-B-CA-SF, Gd-**^{10}B**-CA-SF, **^{157}Gd**-B-CA-SF, **^{157}Gd-^{10}B**-CA-SF. The longitudinal water proton relaxation rate ($1/T_1$) was measured at each addition of $GdCl_3$ at 21.5 MHz, 25 °C by a Stelar Spin Master relaxometer (Stelar, Mede (PV), Italy). The $1/T_1$ increased linearly with the concentration of $GdCl_3$ until a discontinuity in the slope was detected, which indicated that all the ligand was saturated. After this point, an additional contribution to the overall relaxation rate was observed, which could be attributed to the free Gd complex. On the base of these relaxometric titrations, all the different Gd-B-CA-SF compounds were complexed. The Gd and B concentrations were double checked by inductively coupled plasma mass spectrometry (ICP-MS) (see below). Finally, Gd-B-CA-SF compounds were lyophilized and stored at -20 °C until further analyses were carried out.

Determination of CA Esterase Activity

Recombinant Bovine or human carbonic anhydrase II (BCA II or HCA II) and IX (HCA IX) enzymes and *p*-nitrophenyl acetate (*p*NPA) were purchased from Sigma Aldrich. All the assay experiments were performed in 96-well flat-bottom plates (VWR International S.r.l. Milano, Italy). The CA esterase activity was determined using an ester substrate (*p*NPA) which is hydrolyzed in the chromophore 4-nitrophenol (4-NP) that can be easily quantified spectrophotometrically. The presence of a specific CA inhibitor reduces the enzyme activity, thereby causing a decrease in the observed absorbance. To measure the affinity of the *p*NPA substrate for the enzymes, *p*NPA (dissolved in acetone) was incubated in a range of 0.2 - 10 mM in the presence of a fixed concentration of the enzyme BCA II or HCA II (5 µg/mL (166 nM)) or HCA IX (5 µg/mL (119 nM), in a final volume of 100 µL in a reaction mixture buffer (12.5 mM Tris, 75 mM NaCl, pH 7.5). The concentration of acetone in the final volume was 5% for [*p*NPA] ≤ 2 mM and 10% [*p*NPA] > 2 mM. The binding affinity (**Fig S1**) was determined by measuring the formation of 4-NP at 405 nm, utilizing the GloMax® Discover Microplate Reader (Promega Corporation, Milano, Italy), at room temperature, for 1 hour (BCA II, HCA II) and 2 hours (HCA IX).

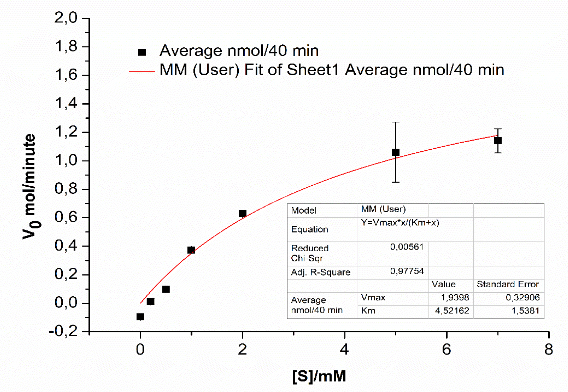

Fig S1 HCA II enzymes at a concentration of 5 µg/mL (0.166) were titrated with the substrate *p*NPA, ranging from 0.2 mM to 10 mM, in acetone (5–10%) to account for its limited solubility in aqueous solutions. The V_{max} and K_m values were determined using the Michaelis-Menten equation, and the curve was generated through least squares non-linear fitting in Origin software. The data represent two independent experiments performed in triplicate.

In order to use the same conditions of the experiment with CA inhibitors (*vide infra*), the enzyme solutions were pre-incubated at 25 °C for 15 minutes (for BCA II and HCA II) and at 37 °C for 1 hour (for HCA IX). Data were analyzed with OriginPro 8.5 software. The values for V_{max} and K_m were determined using the Michaelis-Menten equation. The inhibition of BCA II, HCA II and HCA IX esterase activity was assessed by incubating BCA II, HCA II (5 µg/mL, 166 nM) or HCA IX (5 µg/mL, 119 nM) with two different carbonic anhydrase inhibitors (**Fig S2**): CA-SF was tested in a concentration range of 0.01–2 µM for BCA II or HCA II and 0.1–20 µM for HCA IX, while Gd-B-CA-SF was studied in a concentration range of 0.1–4 µM for BCA II or HCA II and 0.1–50 µM for HCA IX, in the presence of *p*NPA substrate (0.5 mM) in 100 µL of final volume in a reaction mixture buffer (12.5 mM Tris, 75 mM NaCl, pH 7.5). The Gd-B-CA-SF was dissolved in phosphate Buffer Saline (PBS 1X) and CA-SF, previously dissolved in DMSO (Sigma-Aldrich), was then mixed with HP-β-CD (in PBS) at a molar ratio of 1:5 (CA inhibitor:HP-β-CD) due to its low water solubility. As mentioned before, the pre-incubation with enzymes was performed at 25 °C for 15 minutes (for B CA II and HCA II) and at 37 °C for 1 hour (for HCA IX). The esterase activity was assessed by measuring the absorbance at 405 nm at regular intervals of 10 minutes over 180 minutes at room temperature.

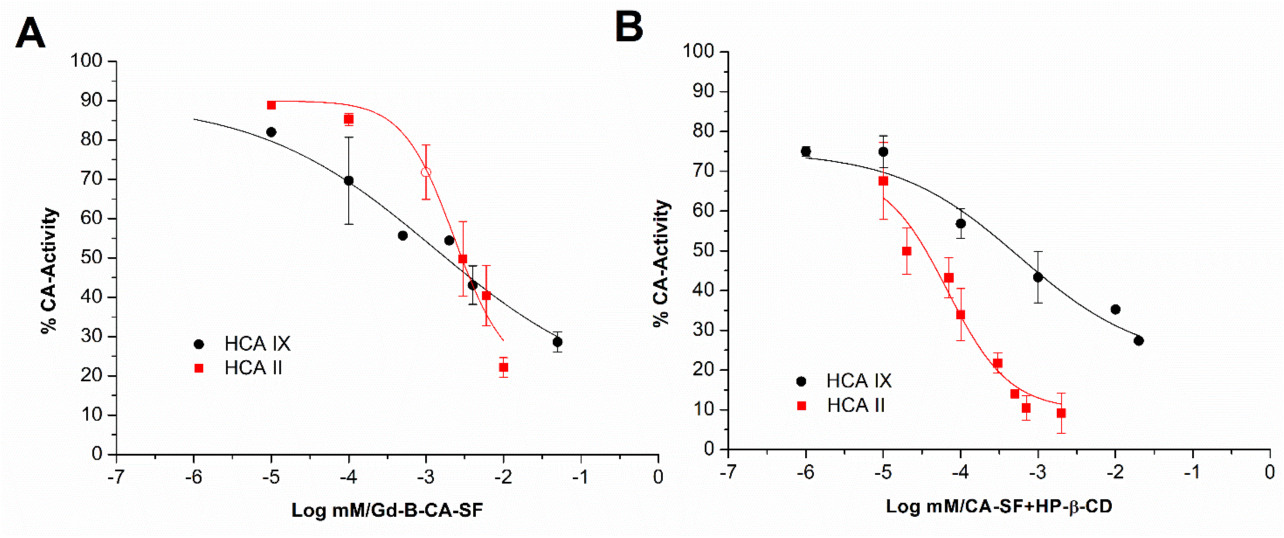

Fig S2 Inhibition of esterase activity by **A)** Gd-B-CA-SF and **B)** CA-SF against H CA II and H CA IX in a dose-dependent manner, with corresponding IC_{50} values presented in Table 2.

The esterase activity (%) in the presence or in the absence of the CA inhibitors was calculated through kinetic activity curves by plotting the absorbance (405 nm) on the Y-axis and time (minutes) on the X-axis and the IC_{50} values were calculated by using OriginPro 8.5 software. The dissociation constant (K_{diss}) was calculated using the following formula:

$$K_{diss} = \left(\frac{IC_{50}}{1+Substrate\ concentration}\right)/K_m \qquad \text{Eq } (\mathbf{1})$$

The percentage of CA inhibition was calculated by setting to 100% the esterase activity in the absence of inhibitors.

Relaxometric measurements

The water proton $1/T_1$ longitudinal relaxation rates (21.5 MHz, 25 °C) were measured with a Stelar Spinmaster Spectrometer. ^{1}H spin-lattice relaxation times T_1 were acquired by the standard inversion recovery (IR/S) method. The temperature was controlled with a Stelar VTC-91 airflow heater equipped with a copper–constantan thermocouple (uncertainty of 0.1 ±°C). To measure the Gd concentration in Gd-B-CA-

SF solutions, a glass vial containing 100 mL of HCl 37% and 100 mL of Gd-B-CA-SF in PBS solution was placed in an oven at 110 °C for 16 hours. Upon this treatment, all Gd is dissolved as the free aquo-ion. The Gd^{3+} concentration was measured using a Stelar Spinmaster Spectrometer and determined by referring to a calibration curve generated from standard $GdCl_3$ solutions ranging from 0.01 to 2 mM. To measure the relaxation enhancement induced by the binding with BCA II, Gd-B-CA-SF dissolved in PBS at a final concentration of 50 µM was measured alone or after the incubation for 30 minutes at room temperature with 50 µM BCA II in PBS. Moreover, Gd-B-CA-SF underwent a 30-minutes preincubation at room temperature with 10 mM acetazolamide (AZ) (stock solution in DMSO) before introducing the B CA II enzyme using the same experimental condition described above. As the control reference, Gd-B-CA-SF was incubated for 30 minutes at RT in the presence of 10 mM AZ.

Nuclear Magnetic Resonance Dispersion (NMRD) profiles.

^{1}H-NMRD profiles were measured at 25 °C on a Stelar (Mede (PV), Italy) SmarTracer relaxometer by measuring T_1 at magnetic field strengths from 2.4×10^{-4} to 0.235 T (corresponding to proton Larmor frequencies from 0.01 to 10 MHz). This relaxometer works under complete computer control with an absolute uncertainty in R_1 of ±1%. Additional points, from 0.5 T (21 MHz) to 1.7 T (70 MHz), were collected on a manually tunable Stelar Spinmaster spectrometer working under a variable magnetic field. The temperature was controlled by a Stelar VTC-91 airflow heater equipped with a copper–constantan thermocouple; the actual temperature in the probe head was measured with a Fluke 52 k/j digital thermometer (Fluke AG, Zürich, Switzerland), with an uncertainty of ±0.2 °C. The data were analyzed through Origin 8.5 software using the Solomon-Bloembergen-Morgan model (inner sphere contribution) and the Hwang and Freed model (outer sphere contribution).

Table S1 Parameters obtained from the analysis of the NMRD profile (298 K) data for Gd-B-CA-SF.

Δ^2 ($\cdot 10^{19}$ s^{-2})	τ_V (ps)	τ_R (ps)	τ_M (ns)*	r (Å) *	q (Å) *	a (Å) *	D (cm^2 s^{-1}) *	A/ħ (10^6 rad s^{-1*})	S*
1.45±0.3	29± 6	120 ± 8	130±6	3.1	1	3.6	$2.2 \cdot 10^{-5}$	0	3.5

Cell lines

AB22 murine mesothelioma and Met-5a immortalized human mesothelium cell lines were obtained from Sigma-Aldrich. AB22 cells were cultured in RPMI 1640 medium (Lonza) supplemented with 25 mM Hepes, 10% (v/v) fetal bovine serum (FBS), and 2 mM L-glutamine. Met-5a cells were cultured in Medium 199 with Earle's salts (Lonza), supplemented with 10% (v/v) FBS, 1:1000 trace element B, 870 nM bovine insulin, 3.3 nM epidermal growth factor, and 400 nM hydrocortisone. Mediums were supplemented with 100 U/mL penicillin and 100 U/mL streptomycin. Cell lines were maintained at 37 °C in a 5% CO_2 incubator.

MTT Assay

The MTT assay was used to assess the reduction of succinate dehydrogenase in metabolically active cell mitochondria, leading to the formation of purple formazan crystals. Following the protocol outlined by Azzi *et al.*,[6] the assay was performed as follows: AB22 and Met-5a cells were seeded at densities of 8×10^3 and 9×10^3 cells per well, respectively, in a 96-well microtiter plate. After 24 hours of incubation at 37 °C with 5% CO_2, the cells were exposed to increasing concentrations (0 – 0.5 mM) of Gd-B-CA-SF (stock solution in PBS

1X) and (0 - 0.21 mM) of CA-SF (stock solution in DMSO) for 24 hours under normoxic conditions (37 °C and 5% CO_2). The concentration of PBS 1X and DMSO in the cell medium did not exceed 10% and 0.4% (v/v), respectively, across highest tested concentration of the inhibitors. After the incubation, the medium was removed, and each well was treated with thiazolyl blue tetrazolium bromide (Sigma-Aldrich) prepared at a concentration of 5 mg/mL in filtered PBS and then given to the cells diluted in medium at a concentration of 0.45 mg/mL. The cells were then incubated for 4 hours at 37 °C with 5% CO_2. Afterward, the medium was removed, and 150 µL of DMSO were added to each well to dissolve the formazan crystals produced due to live cell metabolism. The microplate was incubated at room temperature for 30 minutes, and absorbance was measured at 560 nm using a GloMax® Discover Microplate Reader (Promega Corporation, Milano, Italy). Cell viability was calculated as the percentage of viable cells in the treated samples relative to the non-treated control cells set at 100%. Statistical significance between different cell lines and compounds was assessed using p-values (Table S2). All Student's *t*-test statistical analyses in this study were performed using OriginPro 8.5 software.

Table S2 Student's t-test statistical analysis based on results shown in Figure 2. In red, not significant (ns) $p > 0.05$ and in orange $^{**}p \leq 0.01$.

**Cell viability Assay (MTT)**	AB22 (CA-SF) 200 µM	AB22 (Gd-B-CA-SF) 200 µM	AB22 (Gd-B-CA-SF) 500 µM	Met-5a (Gd-B-CA-SF) 200 µM
AB22 (CA-SF) 200 µM		0.0776		
MET-5A (CA-SF) 200 µM	0.1425			0.0045
Met-5a (Gd-B-CA-SF) 200 µM		0.1287		
Met-5a (Gd-B-CA-SF) 500 µM			0.105	

Uptake Experiments

For *in vitro* uptake experiments, AB22 and Met-5a cells were seeded in 6 cm diameter dishes at densities of 7×10^5 and 8×10^5 cells, respectively. After 24 hours at 37 °C and 5% CO_2 in a cell incubator, the cells were incubated for additional 24 hours with increasing concentrations (0 - 0.2 mM) of Gd-B-CA-SF or Gd-HPDO3A, provided by Bracco SpA (Colleretto Giacosa, TO, Italy). At the end of the incubation, the cells were washed three times with ice-cold PBS, detached using 0.02% EDTA or 0.05% trypsin/0.02% EDTA (Lonza), collected in 200 µL of PBS and then sonicated at 30% power for 30 seconds on ice. The protein content of each cell sample was determined by the Bradford assay (BioRad), using bovine serum albumin as a standard. Finally, the Gd content internalized by each cell sample was measured by ICP-MS and normalized to the total protein content.

Inductively coupled plasma mass spectrometry (ICP-MS)

Gd and Boron content of cell samples from *in vitro* uptake experiments, *in vivo* biodistribution and from Gd-B-CA-SF stock solutions were determined using inductively coupled plasma mass spectrometry (ICP-MS) (Element-2; Thermo-Finnigan, Rodano (MI), Italy). Cell samples and Gd-B-CA-SF stock solutions digestion was performed using a high-performance Microwave Digestion System (ETHOS UP Milestone, Bergamo, Italy) after the addition of concentrated HNO_3 (70%) to cell lysates or Gd-B-CA-SF stock solution (1:1), in a final volume of 0.4 mL. The amount of Gd and Boron in cell samples measured by ICP-MS, was normalised to the protein content of each cell sample. For *in vivo* biodistribution study, the mice blood was collected and centrifuged at 2300 rpm for 8 minutes to split the plasma part over whole blood; the tumors, organs and healthy tissues (muscle) were explanted. All was weighted, dissolved in HNO_3 (70%) and digested. Both Gd and boron standard solution was analyzed during sample runs to check for variations in the systematic bias. The calibration curves were obtained using four absorption standard solutions (Sigma-Aldrich) in the range 0.1-0.005 µg Gd/mL and 0.1–0.004 µg B/mL. The amounts of Gd and Boron in organs, plasma, healthy tissues, and tumors measured by ICP-MS were normalized to the weight of each sample.

Competition Assay

To assess the specificity of Gd-B-CA-SF on mesothelioma and healthy mesothelium cell lines, AB22 and Met-5a cells were seeded in 6 cm dishes at densities of 7×10^5 and 8×10^5 cells per dish, respectively. After 24 hours of keeping the dishes at 37 °C with 5% CO_2, cells were pre-treated with AZ (Sigma-Aldrich) (stock solution in DMSO) at a concentration of 4 mM for 1 hour at 37°C, 5% CO_2 with or without the subsequent addition of 0.2 mM Gd-B-CA-SF, that was incubated for other 5 hours at 37°C with 5% CO_2. In all the experimental conditions (untreated control cells and treated ones) the amount of DMSO was kept at 0.4%. After the incubation, the cells were detached with 0.02% EDTA, collected in 200 µL of PBS (1X), and sonicated on ice at 30% power for 30 seconds. Protein and Gd contents, in each cell sample, were quantified as described above.

Evaluation of *in vitro* Gd-B-CA-SF uptake by magnetic resonance imaging (MRI)

MR images were acquired using a Bruker Avance Neo 300 MHz spectrometer (7 T) equipped with a Micro 2.5 microimaging probe (Bruker BioSpin, Ettlingen, Germany). Following the procedure described above for the *in vitro* uptake experiments, AB22 and Met-5a cells were incubated for 24h at 37 °C, 5% CO_2 in the presence of 0.2mM Gd-B-CA-SF. After the incubation, the cells were washed with ice-cold PBS, detached with EDTA, resuspended in 50 µL of PBS and their pellets were collected at the bottom of glass capillaries. The glass capillaries containing AB22 and Met-5a cells were placed in an agar phantom, and MR imaging was performed using a standard T_1-weighted multi-slice spin-echo sequence (TR/TE/NEX = 200/3.0/10.0, FOV = 1 cm) (**Table S3**). T_1 relaxation times were calculated using a standard Saturation Recovery Spin Echo sequence.

Table S3 R_1 values for cell pellets were measured by acquiring T_1-weighted spin-echo MR images at 7 T

Conditions	R_1 (S^{-1}) ± SD
AB22-Control	0.453 ± 0.009
AB22-Gd-B-CA-SF (0.2 mM)	0.734 ± 0.026
Met-5a-Control	0.47 ± 0.01
Met-5a-Gd-B-CA-SF (0.2 mM)	0.531 ± 0.004

Cell Irradiation

AB22 cells (7 × 10^5) were seeded into fourteen T25 flasks. After 24 hours of incubation at 37 °C, 5% CO_2, the cells were incubated for other 24 hours at 37 °C, 5% CO_2 with 0.2 mM of Gd-B-CA-SF compound containing all the potential combinations of enriched and natural abundance Gd and B. Two flasks were designated for each condition as follows: Irradiated group: untreated cells (CTRL IRR); cells treated with the compounds containing: i) natural abundant B and Gd (Gd-B-CA-SF IRR), ii) ^{10}B enriched (Gd-^{10}B-CA-SF IRR), iii) ^{157}Gd enriched (^{157}Gd-B-CA-SF IRR), and iv) both ^{10}B and ^{157}Gd enriched (^{157}Gd-^{10}B-CA-SF IRR). Not irradiated group: untreated cells (CTRL NN IRR) and cells treated with the natural abundant B and Gd compound (Gd-B-CA-SF NN IRR). The irradiated group was irradiated for 15 minutes at 30 kW reactor power in the thermal column of the TRIGA Mark II reactor at the University of Pavia, Italy. After the irradiation, the medium of all flasks (including irradiated and not irradiated) was renewed, and all flasks, including the non-irradiated group, were incubated in a humidified atmosphere at 37 °C with 5% CO_2 for 24 hours.

Cell viability and Proliferation Assay

The day after irradiation, all the irradiated and non-irradiated cells were detached using 0.05% trypsin and 0.02% EDTA, and their viability was evaluated with the trypan blue exclusion test. The initial cell viability was expressed as the percentage of live cells compared to the non-irradiated untreated cells (CTRL NN IRR) set to 100%. Subsequently, the proliferation assay was performed by seeding 1 × 10^5 AB22 cells from each flask into 10 cm culture dishes. Their cell growth was monitored over 15 days post-neutron irradiation. At predetermined time intervals, cells were washed with PBS, detached using 0.05% trypsin and 0.02% EDTA, and transferred to Falcon tubes. The cells were then sonicated for 30 seconds at 30% power on ice. The total protein concentration of the cell lysates was measured using the Bradford assay, with a calibration curve [(mg protein)/(number of cells)]. Based on the relationship between mg of protein and cell number, it has been determined that for AB22 cells, 1 mg of protein corresponds to 1.65 × 10^6 cells. Statistical comparisons between cell incubation conditions were performed on days 6 and 13/15 using p-values (Table S4).

Table S4 Student's t-test statistical analysis based on results shown in Figure 6B. In red, not significant (ns) $p > 0.05$, n=2; in yellow *$p \leq 0.05$, n=2; in orange **$p \leq 0.01$ n=2; and in green ***$p \leq 0.001$ n=2.

**Proliferation (day 6)**	CTRL NN IRR	CTRL IRR	Gd-B-CA-SF NN IRR	Gd-B-CA-SF IRR	Gd-^{10}B-CA-SF IRR	^{157}Gd-B-CA-SF IRR
CTRL IRR	0.0357					
Gd-B-CA-SF NN IRR	0.0369	0.4363				
Gd-B-CA-SF IRR	0.1120	0.8705	0.3119			
Gd-^{10}B-CA-SF IRR	0.0076	0.0346	0.0313	0.0605		
^{157}Gd-B-CA-SF IRR	0.0011	0.0020	0.0003	0.0112	0.0115	
^{157}Gd-^{10}B-CA-SF IRR	0.0010	0.0017	0.0003	0.0103	0.0099	0.0227
**Proliferation (day 13/15)**	CTRL NN IRR	CTRL IRR	Gd-B-CASF NN IRR	Gd-B-CASF IRR	Gd-^{10}B-CASF IRR	^{157}Gd-B-CASF IRR
^{157}Gd-^{10}B-CA-SF IRR						0.0387

Experimental Mice and Induction of Transplantable Tumors

7/8-weeks-old adult male Balb/c mice were purchased from Envigo and maintained in a specific pathogen-free environment at the animal facility of the Department of Molecular Biotechnology and Health Sciences, University of Turin, Italy. The care and handling of the animals followed the guidelines outlined in the EU Directive 2010/63/EU for animal experiments. The experimental protocol was approved by the Italian Ministry of Health under Authorization Number n° 245/2024-PR (prot. CC652.216).

AB22 cells were cultured as previously described, and Tumors were induced by subcutaneous injection of 2.5×10^6 AB22 cells in a final volume of 0.15 mL GFNR Matrigel (CORNING Ref. 354,234): PBS (1:1 v/v) into the neck of each mouse.

In vivo MRI evaluation

Prior to the MRI acquisition, animals were anesthetized by intramuscle injection of tiletamine/zolazepam (Zoletil 100; Virbac, Milan, Italy) at 10 mg/kg and xylazine (Rompun; Bayer, Milan, Italy) at 2.6 mg/kg.

The tumor volume was measured using T_2-weighted MRI images obtained with a rapid acquisition with refocused echoes (RARE) sequence protocol (TR = 3500 ms; TE = 40.57 ms; number of slices = 20; slice thickness = 1 mm; FOV = 30 × 30 mm; matrix = 128 × 128). Tumor volume was calculated using ITK-SNAP software by manually drawing a region of interest (ROI) encompassing the entire tumor region. The MRI study was carried out on mice with Tumor volumes less than 200 mm^3, which represents half of the maximum volume permitted by the experimental animal protocol approved by the Italian Ministry of Health (Authorization Number 658/2021-PR).

Gd-B-CA-SF or Gd-HPDO3A were administered *via* tail vein injection at a dose of 0.1 mmol Gd/kg. MR images at 7 T were acquired before and at 1, 3.5, 6, and 24 hours post-Gd-B-CA-SF administration using a T_1-weighted, fat-suppressed, RARE sequence (TR = 350 ms; TE = 3.0 ms; NEX = 4; number of slices = 20; slice thickness = 1 mm; FOV = 30 × 30 mm; matrix = 128 × 128). Mean signal intensity (SI) values were determined in manually drawn ROIs on the muscle, liver and the whole tumor, across all time intervals. SI values were normalized using a tube containing a standard Gd-HPDO3A solution. The percentage SI enhancement was calculated using the following equation:

$$\%\, Normalized\ signal\ intensity\ enhancement = \frac{SI\ POST - SI\ PRE}{SI\ PRE} \times 100 \qquad \text{Eq } \mathbf{(2)}$$

Gd and B Concentration Determination by MRI

The concentrations of Gd and B atoms were determined using Eq. (3), derived from the R1POST and R1PRE relaxation rates:

$$[B]\mu g/g = 100 \times \frac{R1\ POST - R1\ PRE}{r_{1p}} \qquad \text{Eq } \mathbf{(3)}$$

Where r1p are the millimolar relaxivities measured at 7 T corresponding to 5.12 and 3.7 $mM^{-1}s^{-1}$ for Gd-B-CA-SF and Gd-HPDO3A, respectively.

PRE-contrast R1 (R1PRE) maps were obtained using a saturation recovery sequence, while post-contrast R1 (R1POST) maps were calculated using Eq. (3), which relates the PRE- and POST-contrast SI ratios in manually drawn ROIs on T_1-weighted images:

$$\frac{SI\ PRE}{SI\ POST} = \frac{1 - expexp\,(-TR\ - TER1\ PRE) exp\,(-TE \times R2)}{1 - expexp\,(-TR - TER1\ POST)\ exp\,(-TE \times R2)} \qquad \text{Eq } \mathbf{(4)}$$

where TR is the repetition time, TE is the echo time, and R1 and R2 are the water proton relaxation rates.

The signal intensities of tumor and muscle in mice administered Gd-B-CA-SF or Gd-HPDO3A were statistically evaluated, and significance was determined using p-values (Table S5).

Table S5: student's t-test statistical analysis based on results shown in Figure 7B. In red, not significant (ns) $p > 0.05$, n=5; in yellow *$p \leq 0.05$, n=5; in orange **$p \leq 0.01$, n=5; and in green ***$p \leq 0.001$, n=5

***In vivo* biodistribution**	Gd-B-CA-SF Tumor 1h	Gd-B-CA-SF Tumor 3.5h	Gd-B-CA-SF Tumor 6h	Gd-B-CA-SF Tumor 24h
Gd-B-CA-SF Muscle 1h	2.1579E-5			
Gd-B-CA-SF Muscle 3.5h		8.2703E-5		
Gd-B-CA-SF Muscle 6h			0.0019	
Gd-B-CA-SF Muscle 24h				0.0160
***In vivo* biodistribution**	Gd-HPDO3A Tumor 1h	Gd-HPDO3A Tumor 3.5h	Gd-HPDO3A Tumor 6h	Gd-HPDO3A Tumor 24h
Gd-HPDO3A Muscle 1h	1.9741E-4			
Gd-HPDO3A Muscle 3.5h		0.0852		
Gd-HPDO3A Muscle 6h			0.8725	
Gd-HPDO3A Muscle 24h				0.2583

Toxicological studies

To evaluate the *in vivo* safety of Gd-B-CA-SF, biochemical determinations in plasma samples and morphological evaluations were performed. Twenty-four hours after administration of Gd-B-CA-SF (0.1 mmol/kg of Gd) (n=3) or vehicle (PBS) (n=2), mice were anesthetized with tiletamine/zolazepam as described above. Blood samples were collected from the caudal vena cava via midline laparotomy, and plasma was separated by centrifugation at 2300 rpm for 8 min at 4 °C. Plasma samples (100 µL) were diluted

1:10 in normal saline and analyzed for renal function indicators, including blood urea nitrogen (BUN) and creatinine, by spectrophotometric analysis using a veterinary chemistry analyzer (IDEXX Catalyst™) with the Chem 17 CLIP panel.

For morphological evaluations Mice were sacrificed by exsanguination and handled liver, kidney and brain were fixed in buffered 4% formaldehyde solution overnight at 4°C. The liver was divided in their lobes, the kidney was cut sagittal, and the brain was sectioned at level of the hippocampus region. Then, four-micron dewaxed sections from them were stained with haematoxylin-eosin and observed under a Leica DM500 microscope (Leica Microsystem, Heerbrugg, Switzerland).

In vivo Irradiation

Treatment for mesothelioma-bearing mice was carried out, by using **^{157}Gd and ^{10}B** enriched compounds (**^{157}Gd-^{10}B-**CA-SF), in the thermal column of the TRIGA Mark II reactor at Pavia University (Italy). The animal irradiation position was characterized in terms of neutron spectrum and background photon dose.[7] The irradiation facility designed for TAOrMINA treatment was originally intended to treat multiple liver metastases with NCT. The animal irradiation chamber is 1 m long, 40×20 cm^2 in cross section, and begins around 1.3 m from the reactor core center. To perform neutron irradiation, animals are positioned near the end of the chamber, with the reactor working at maximum power, where the in air thermal neutron flux is roughly 1.2×10^{10} n/cm^2 s. By doing this, the thermal neutron flow is maximized and the radiation duration is minimized (never exceeding 15 minutes).

Sixteen AB22 tumor-bearing mice, with a tumor volume of 55 ± 17 mm^3, were divided into three groups: 1) control (CTRL NN IRR, n = 6) non treated and not irradiated animals; 2) control irradiated with neutrons (CTRL IRR, n = 5); 3) treated with **^{157}Gd-^{10}B-**CA-SF and irradiated with neutrons (**^{157}Gd-^{10}B-**CA-SF IRR, n = 5). Treated and irradiated group, was treated with a dose of **^{157}Gd-^{10}B-**CA-SF IRR (0.1 mmol Gd/kg and 1 mmol B/kg) administered in the tail vein 3.5 hours before the neutron irradiation. Because the neutron field of the TRIGA Mark II is not collimated, the animals are exposed to it during the radiation treatment. To reduce the amount of neutron exposure to vital organs, a neutron absorber shield made of 95% ^{6}Li-enriched Li_2CO_3 powder was used. Lithium-6 is the ideal isotope for developing effective neutron shields for *in vivo* research because it doesn't produce secondary gamma radiation after thermal neutron capture. The treatment plan was created using the Monte Carlo N-Particles (MCNP) simulation code. By activating Cu wires using the Westcott formalism, neutron flux measurements were used to validate the simulation. Five mice were irradiated simultaneously according to the protocol used for the irradiation experiments. Two Li_2CO_3 neutron shield units which covered the head and abdomen regions protected each mouse from the radiation. To ensure that the tumors are exposed to the neutron flux directly, the units are kept about 1 cm apart.

After irradiation, mice weights (Fig S3) and tumor growth monitored for 22 days using T2-weighted MRI, as described above. Tumor volume was calculated using ITK-SNAP software by manually drawing a region of interest (ROI) encompassing the entire tumor. Tumor volume enhancement (%) was determined using the following formula:

$$Tumor\ volume\ enhancement\ \% = \frac{\text{Tumor volume (time = n)} - \text{Tumor volume (time = 0)}}{\text{Tumor volume (time = 0)}} \times 100 \qquad \text{Eq } \mathbf{(5)}$$

where time = 0 corresponds to the tumor volume before the first treatment, and time = n indicates the volume measured at subsequent time points. Statistical significance between different groups was assessed using p-values (Table S6).

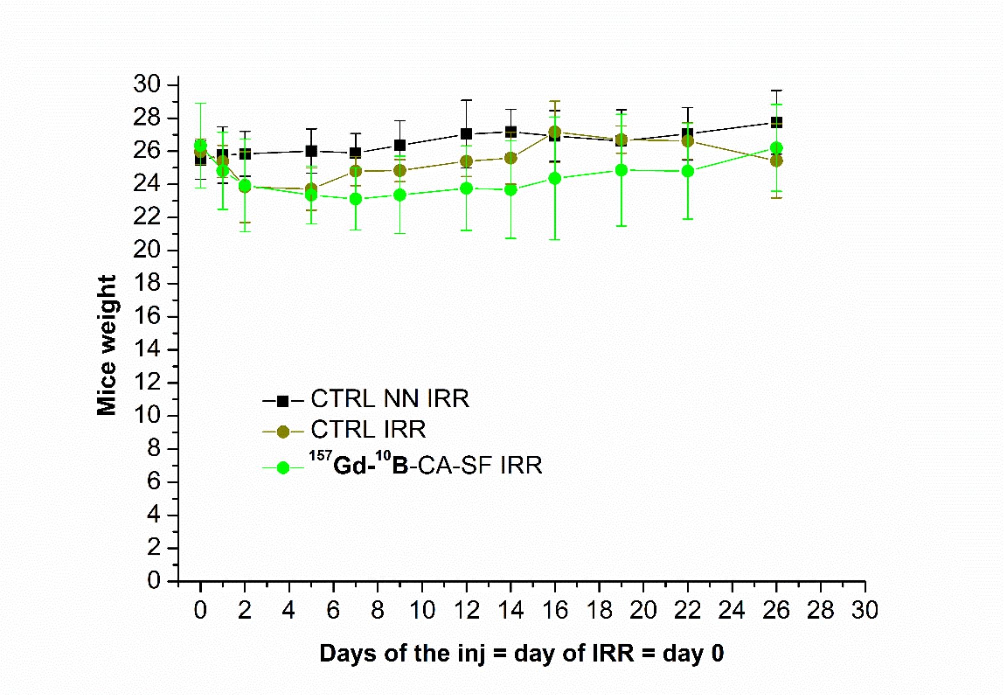

Fig S3 Mouse body weight was measured before and after treatment with ^{157}Gd-^{10}B-CA-SF and irradiation, as well as in control groups (irradiated and non-irradiated). Error bars represent the standard deviation (SD).

Table S6 Statistical significance was determined using an unpaired student's t-test calculated with OriginPro8.5 ($P \leq 0.05$ is significant). In red, not significant (ns) $p > 0.05$, n=5; in yellow $^*p \leq 0.05$, n=5; in orange $^{**}p \leq 0.01$, n=5; and in green $^{***}p \leq 0.001$.

	P-value (day 12)	P-value (day 14)	P-value (day 16)	P-value (day 19)	P-value (day 22)
CTRL NN IRR vs ^{157}Gd-^{10}B-CA-SF IRR	0.002	0.001	0.0009	0.02	0.01
CTRL IRR vs ^{157}Gd-^{10}B-CA-SF IRR	0.2	0.1	0.1	0.2	0.2
CTRL NN IRR vs CTRL IRR	0.9	0.7	0.8	0.8	0.4

References

[]

1. Thomson, D. W.; Commeureuc, A. G. J.; Berlin, S.; Murphy, J. A., Efficient Route to the Pineal Hormone Melatonin by Radical-Based Indole Synthesis. *Synth. Commun.* **2003,** *33* (20), 3631-3641.

2. Li, Y.; Carroll, P. J.; Sneddon, L. G., Ionic-Liquid-Promoted Decaborane Dehydrogenative Alkyne-Insertion Reactions: A New Route to o-Carboranes. *Inorg. Chem.* **2008,** *47* (20), 9193-9202.

3. Alberti, D.; Michelotti, A.; Lanfranco, A.; Protti, N.; Altieri, S.; Deagostino, A.; Geninatti Crich, S., In vitro and in vivo BNCT investigations using a carborane containing sulfonamide targeting CAIX epitopes on malignant pleural mesothelioma and breast cancer cells. *Sci. Rep.* **2020,** *10* (1), 19274.

4. Wilson, J. G.; Anisuzzaman, A. K. M.; Alam, F.; Soloway, A. H., Development of carborane synthons: synthesis and chemistry of (aminoalkyl)carboranes. *Inorg. Chem.* **1992,** *31* (10), 1955-1958.

5. Kaminski, Z. J., 2-Chloro-4,6-Dimethoxy-1,3,5-Triazine - a New Coupling Reagent for Peptide Synthesis. *Synthesis* **1987,** (10), 917-920.

6. Azzi, E.; Alberti, D.; Parisotto, S.; Oppedisano, A.; Protti, N.; Altieri, S.; Geninatti-Crich, S.; Deagostino, A., Design, synthesis and preliminary in-vitro studies of novel boronated monocarbonyl analogues of Curcumin (BMAC) for antitumor and β-amiloyd disaggregation activity. *Bioorg. Chem.* **2019,** *93*, 103324-103334.

7. Bortolussi, S.; Protti, N.; Ferrari, M.; Postuma, I.; Fatemi, S.; Prata, M.; Ballarini, F.; Carante, M. P.; Farias, R.; González, S. J.; Marrale, M.; Gallo, S.; Bartolotta, A.; Iacoviello, G.; Nigg, D.; Altieri, S., Neutron flux and gamma dose measurement in the BNCT irradiation facility at the TRIGA reactor of the University of Pavia. *Nucl. Instrum. Meth. B* **2018,** *414*, 113-120.

NMR spectra

2-(4-Hydroxybut-2-yn-1-yl)isoindoline-1,3-dione (2).

^{1}H NMR (400 MHz, $CDCl_3$)

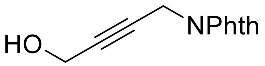

2

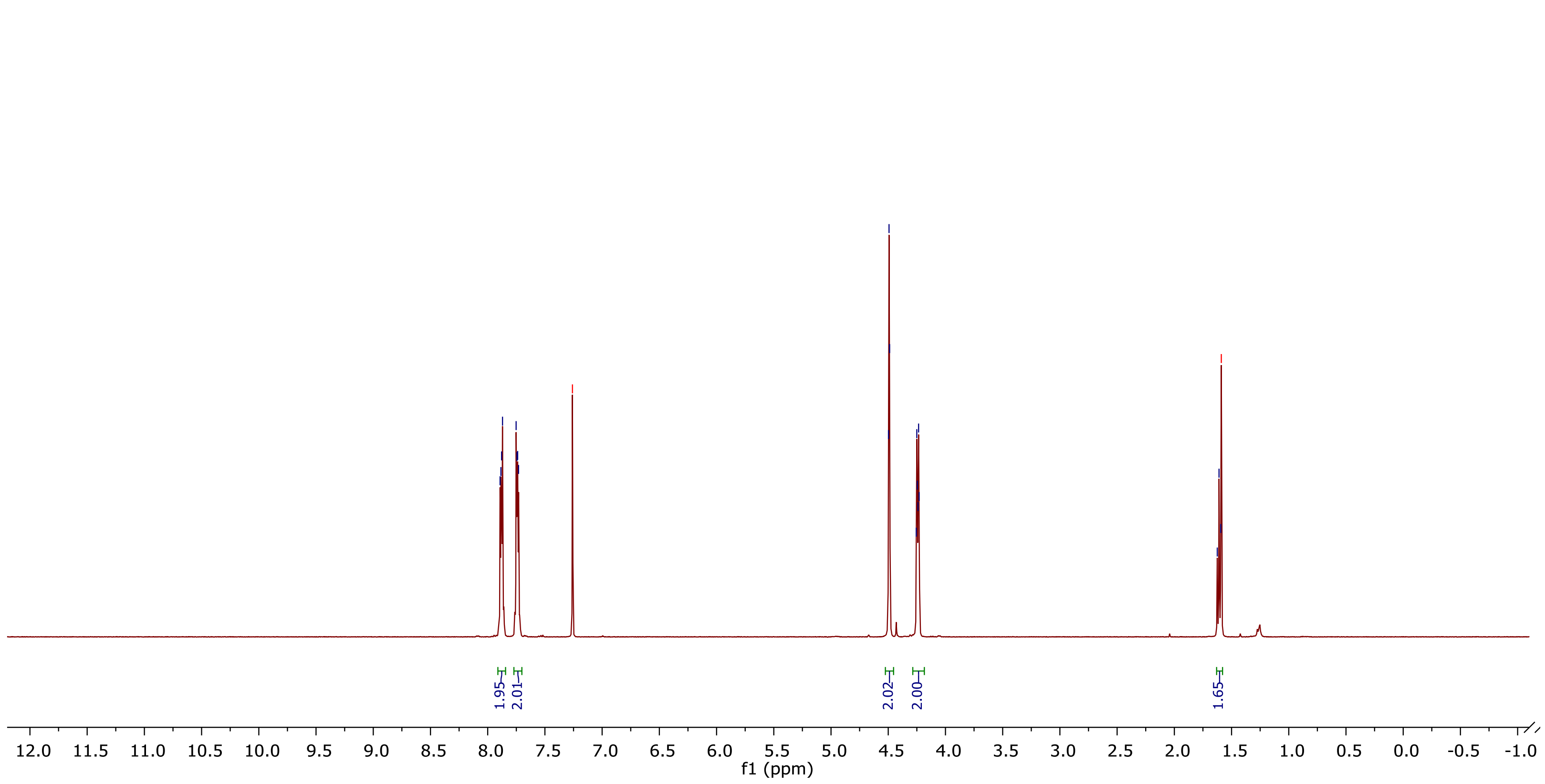

4-(1,3-Dioxoisoindolin-2-yl)but-2-yn-1-yl methanesulfonate (3).

^{1}H NMR (400 MHz, $CDCl_3$)

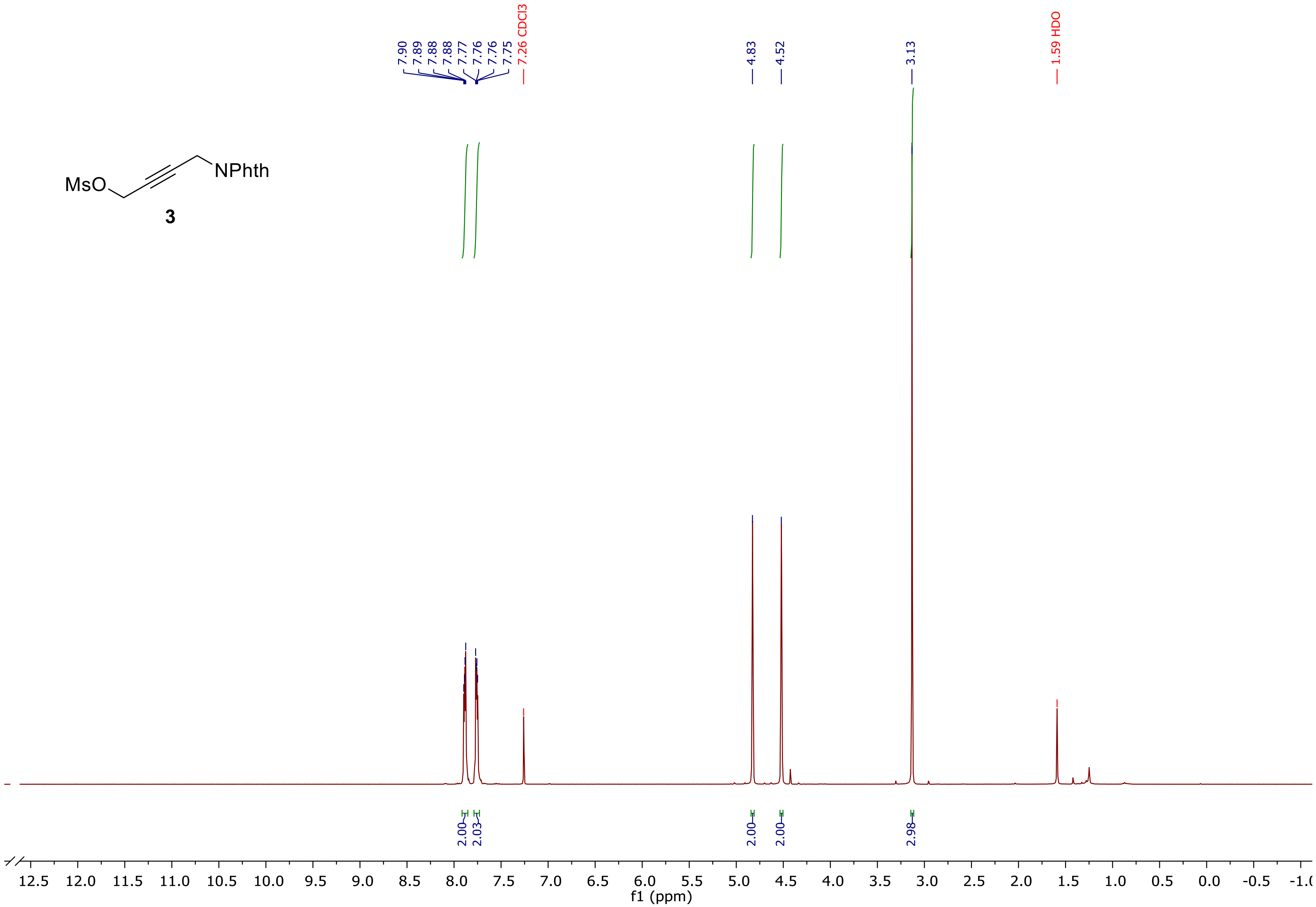

4-(1,3-Dioxoisoindolin-2-yl)but-2-yn-1-yl methanesulfonate (3).

^{1}H-COSY NMR (400 MHz, $CDCl_3$)

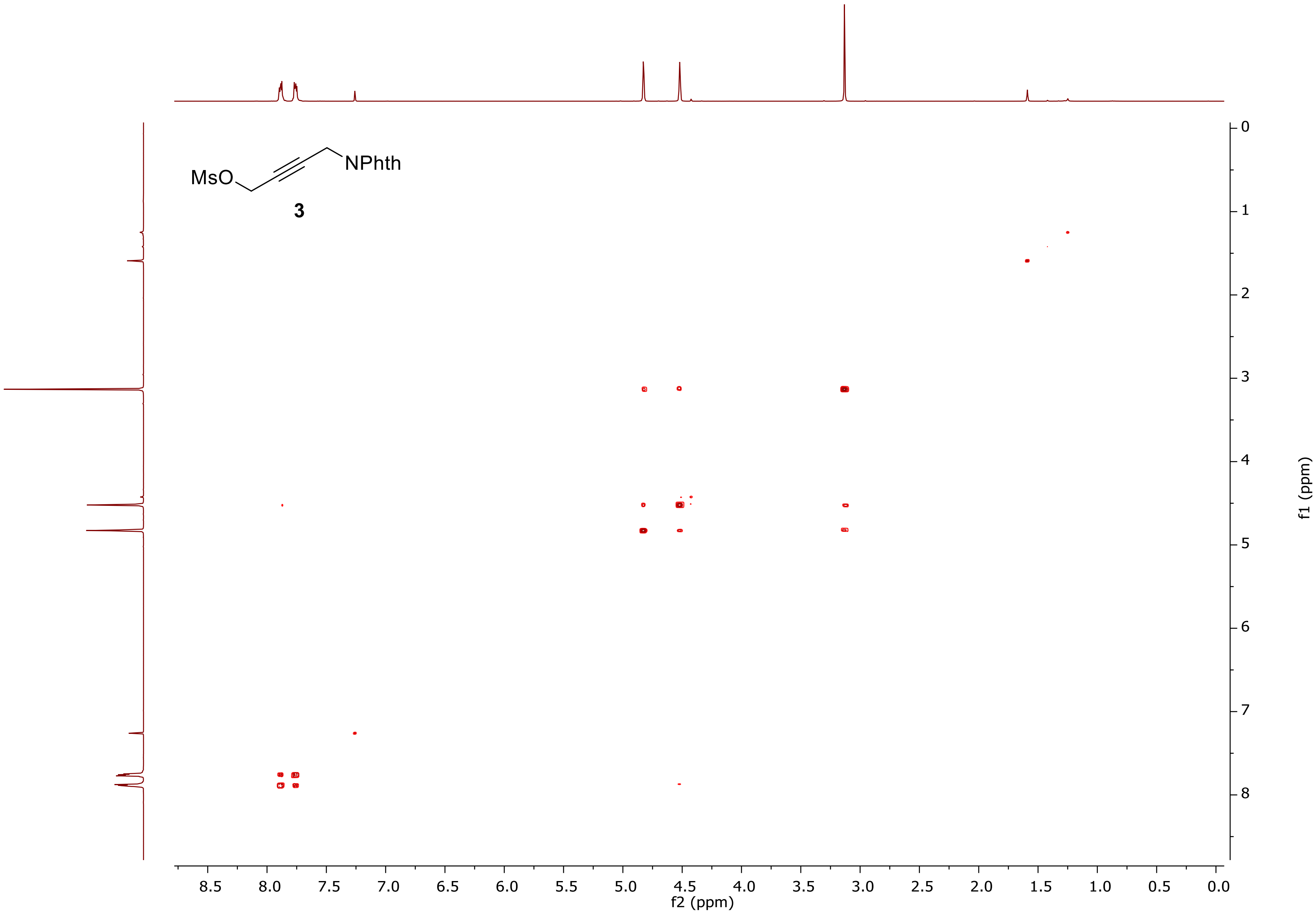

4-(1,3-Dioxoisoindolin-2-yl)but-2-yn-1-yl methanesulfonate (3).

^{13}C NMR (100 MHz, $CDCl_3$)

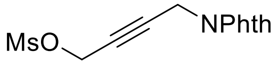

3

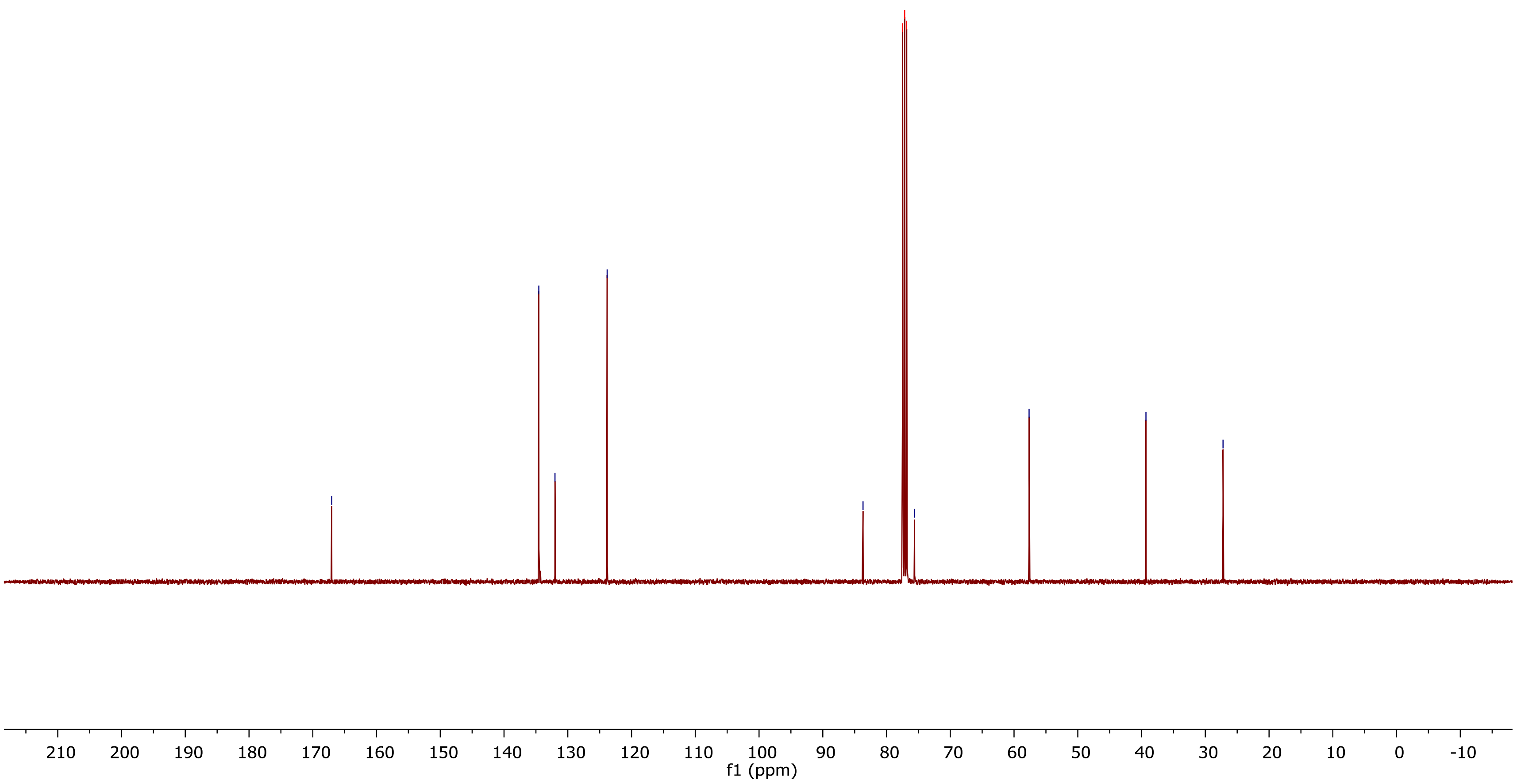

4-(1,3-Dioxoisoindolin-2-yl)but-2-yn-1-yl methanesulfonate (3).

Comparison between DEPT 135 and ^{13}C NMR (100 MHz, $CDCl_3$)

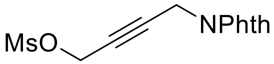

3

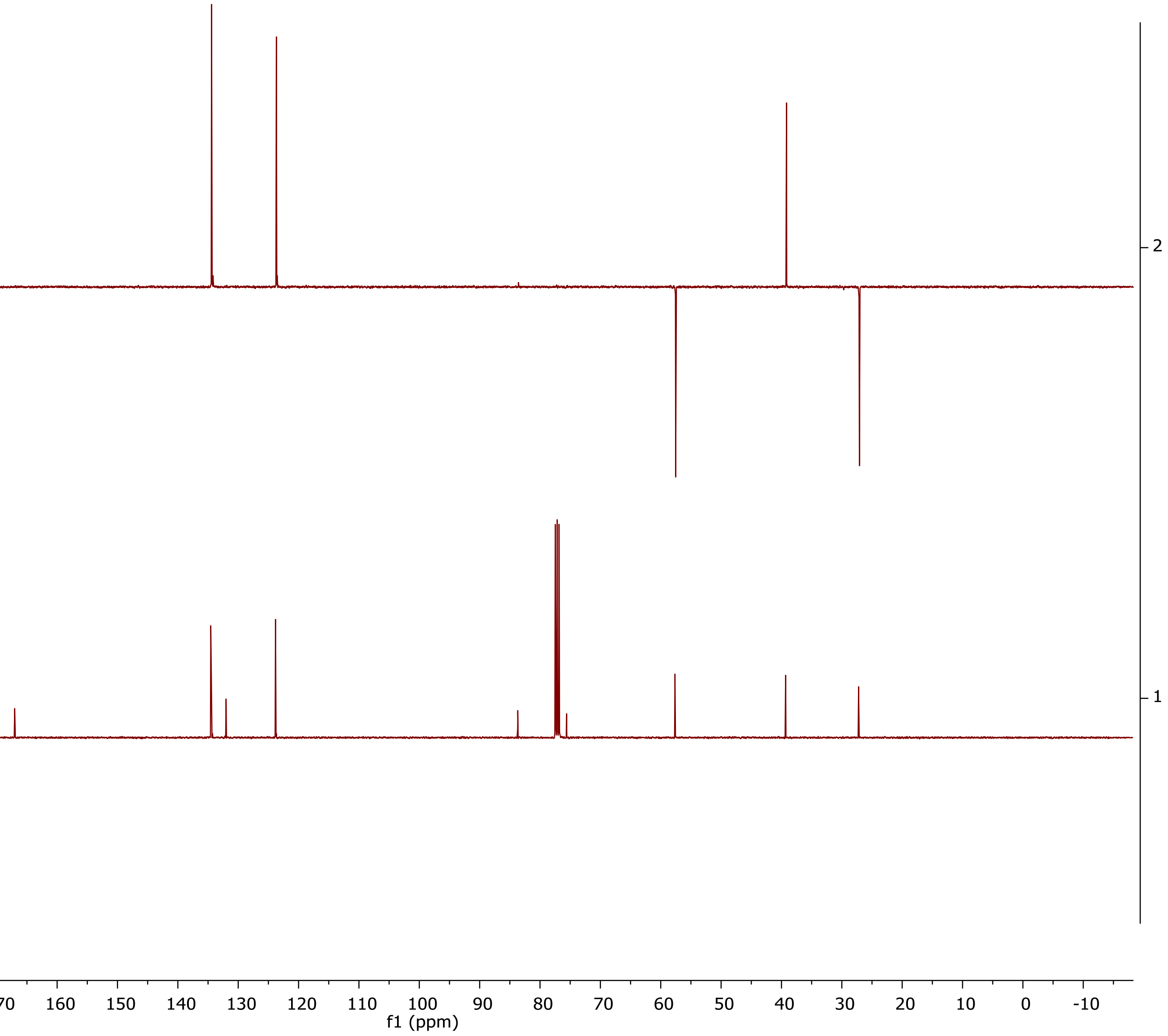

***tert*-Butyl(*tert*-butoxycarbonyl)(4-(1,3-dioxoisoindolin-2-yl)but-2-yn-1-yl)carbamate (4).**

^{1}H NMR (400 MHz, $CDCl_3$)

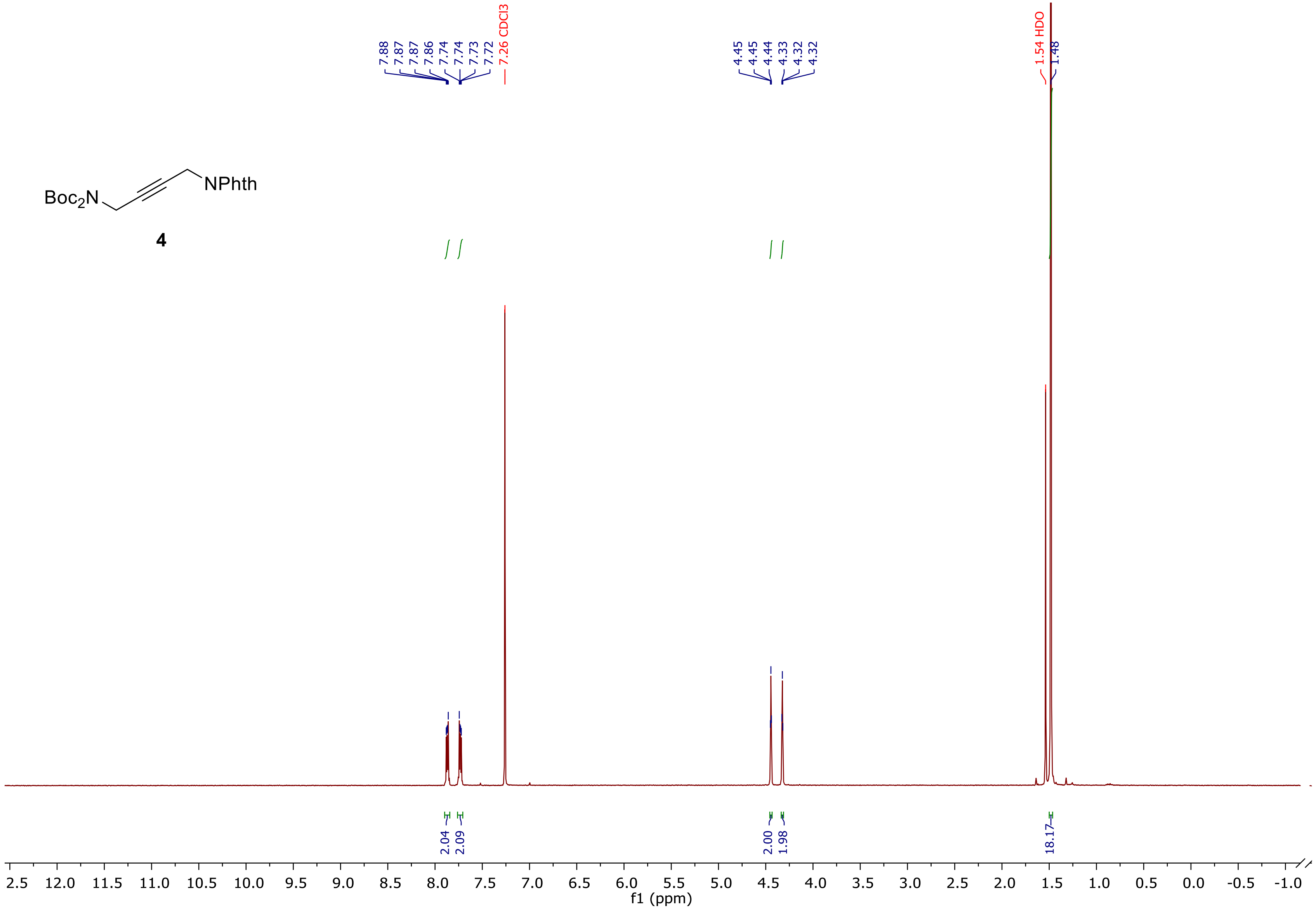

***tert*-Butyl(*tert*-butoxycarbonyl)(4-(1,3-dioxoisoindolin-2-yl)but-2-yn-1-yl)carbamate (4).**

^{1}H-COSY NMR (400 MHz, $CDCl_3$)

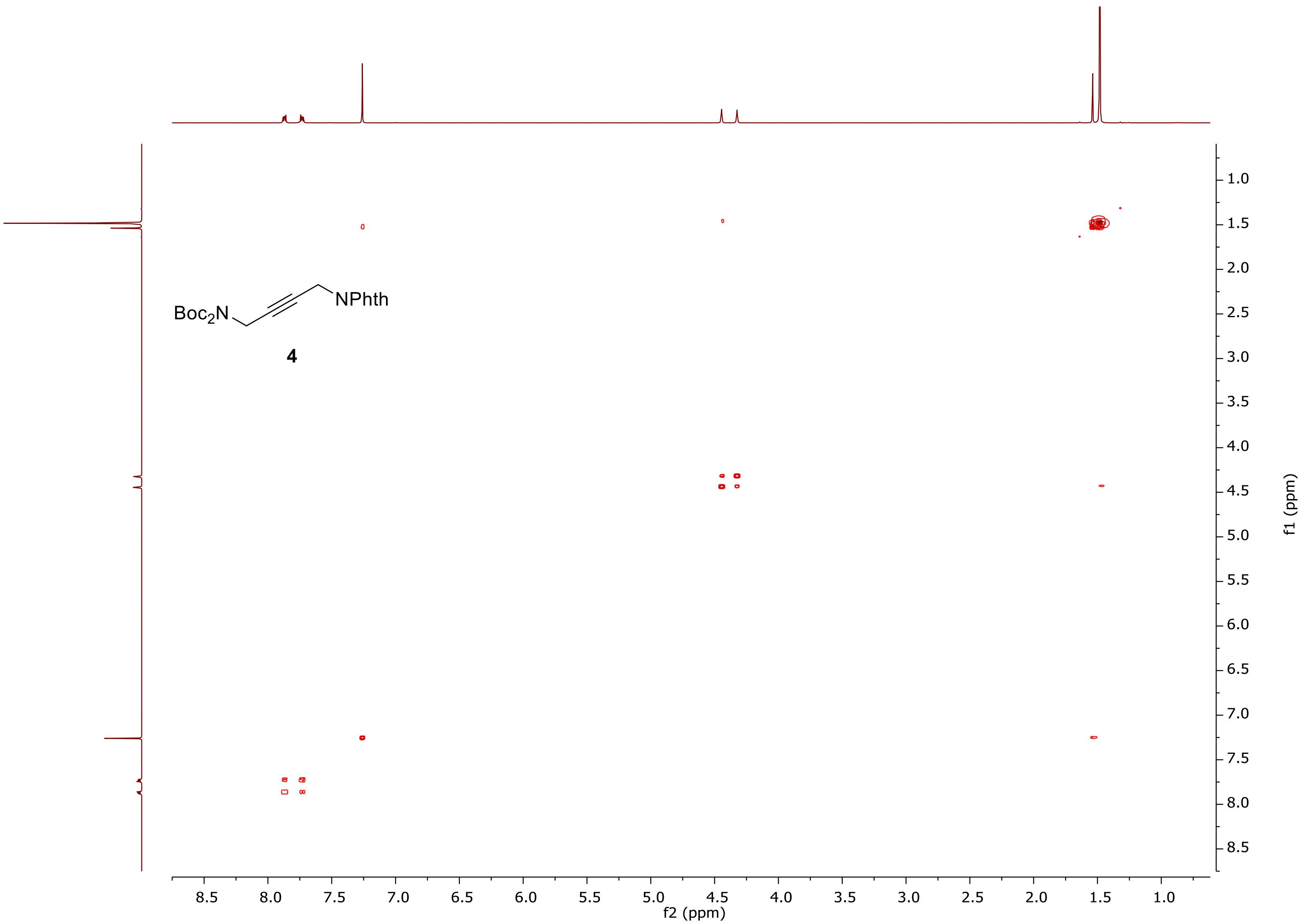

***tert*-Butyl(*tert*-butoxycarbonyl)(4-(1,3-dioxoisoindolin-2-yl)but-2-yn-1-yl)carbamate (4).**

^{13}C NMR (100 MHz, $CDCl_3$)

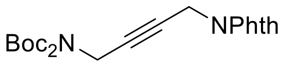

4

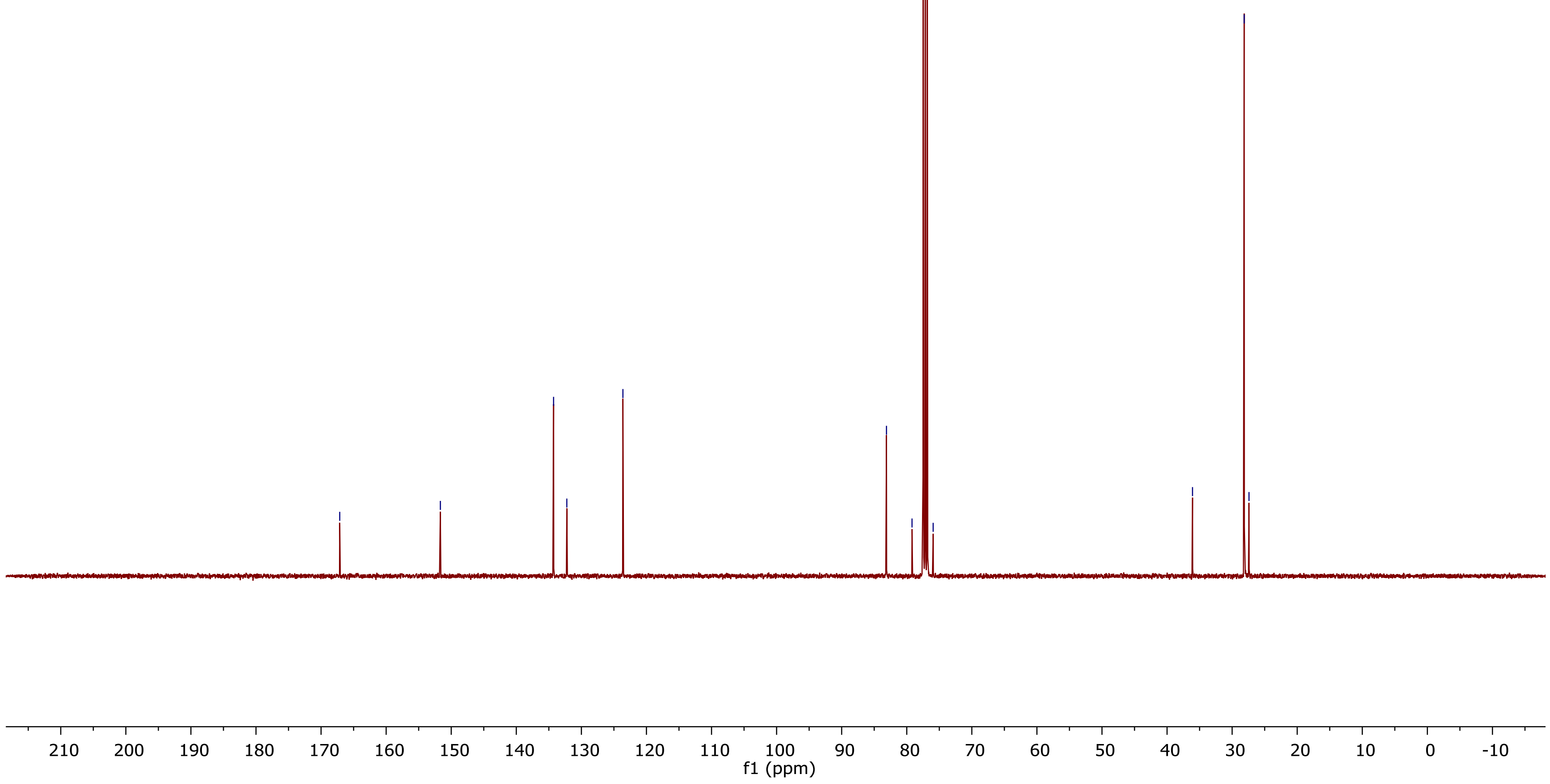

***tert*-Butyl(*tert*-butoxycarbonyl)(4-(1,3-dioxoisoindolin-2-yl)but-2-yn-1-yl)carbamate (4).**

Comparison between DEPT 135 and ^{13}C NMR (100 MHz, $CDCl_3$)

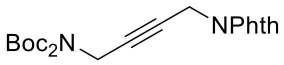

4

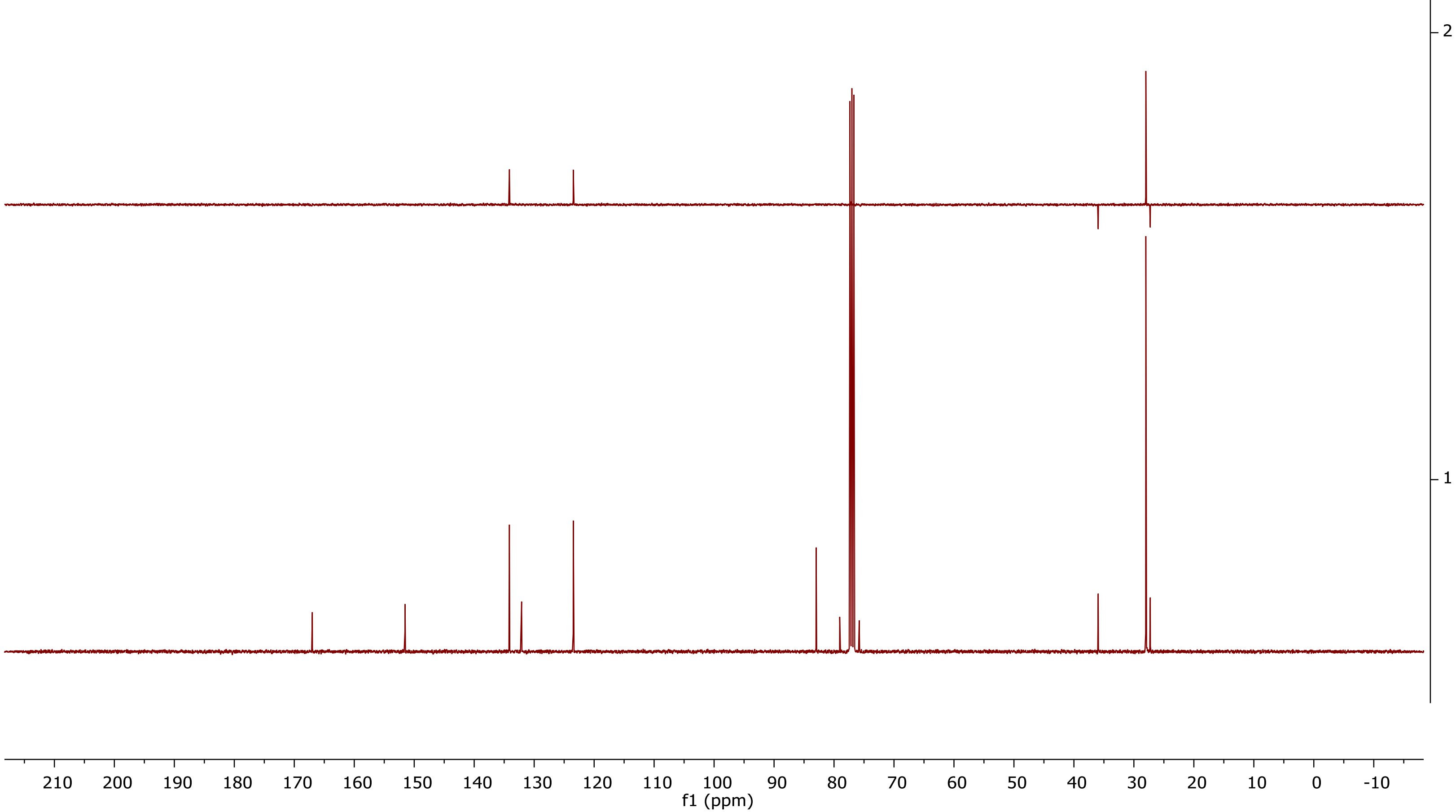

***C*-(*N*-(1,3-dioxoisoindolin-2-yl))methyl-*C'*-2-(*N,N*-di-*tert*-butoxycarbonyl)aminomethyl-*o*-carborane (5).**

^{1}H NMR (400 MHz, $CDCl_3$)

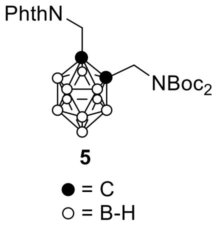

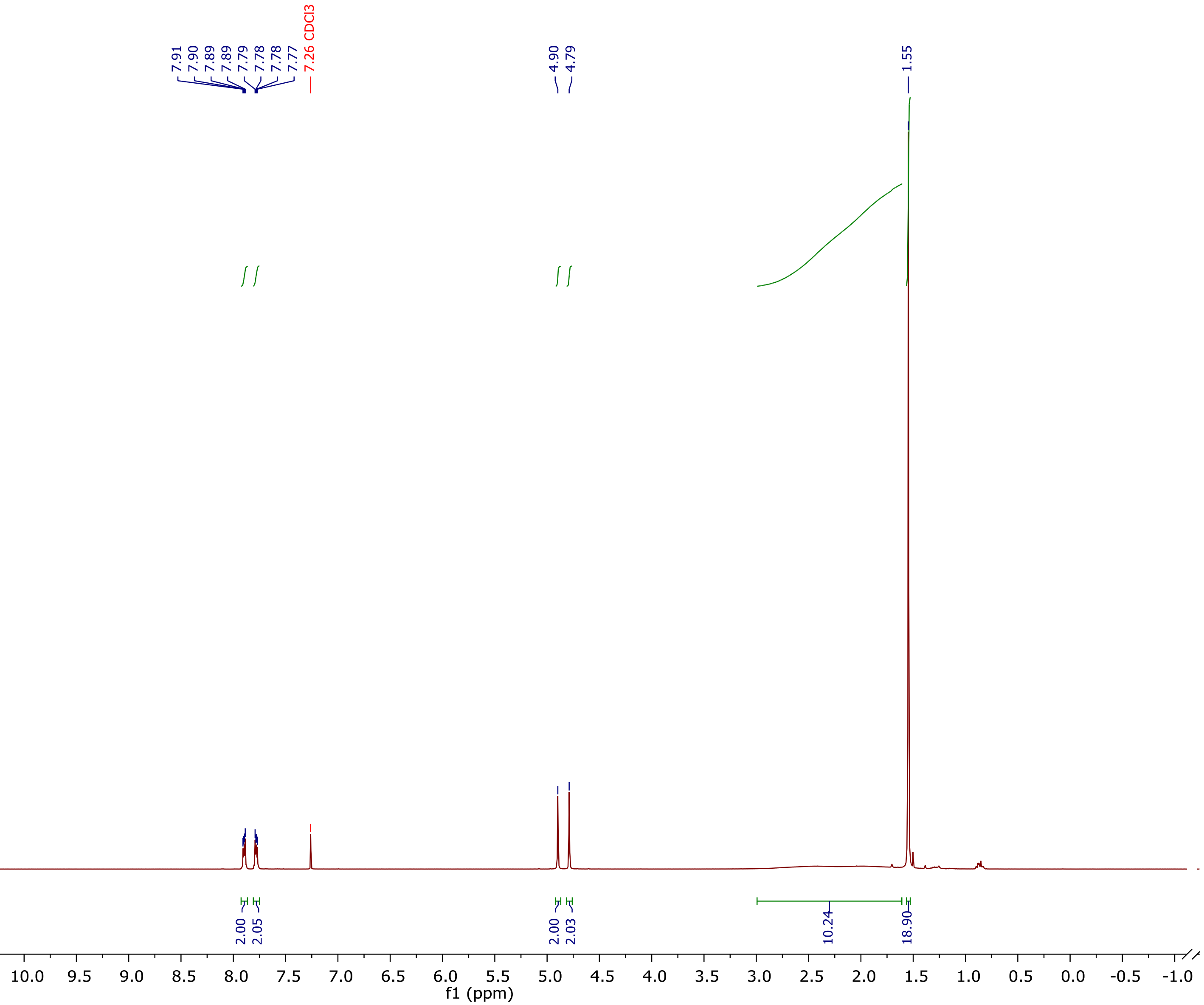

_C_-(_N_-(1,3-dioxoisoindolin-2-yl))methyl-_C'_-2-(_N,N_-di-_tert_-butoxycarbonyl)aminomethyl-_o_-carborane (5).

^{1}H-COSY NMR (400 MHz, $CDCl_3$)

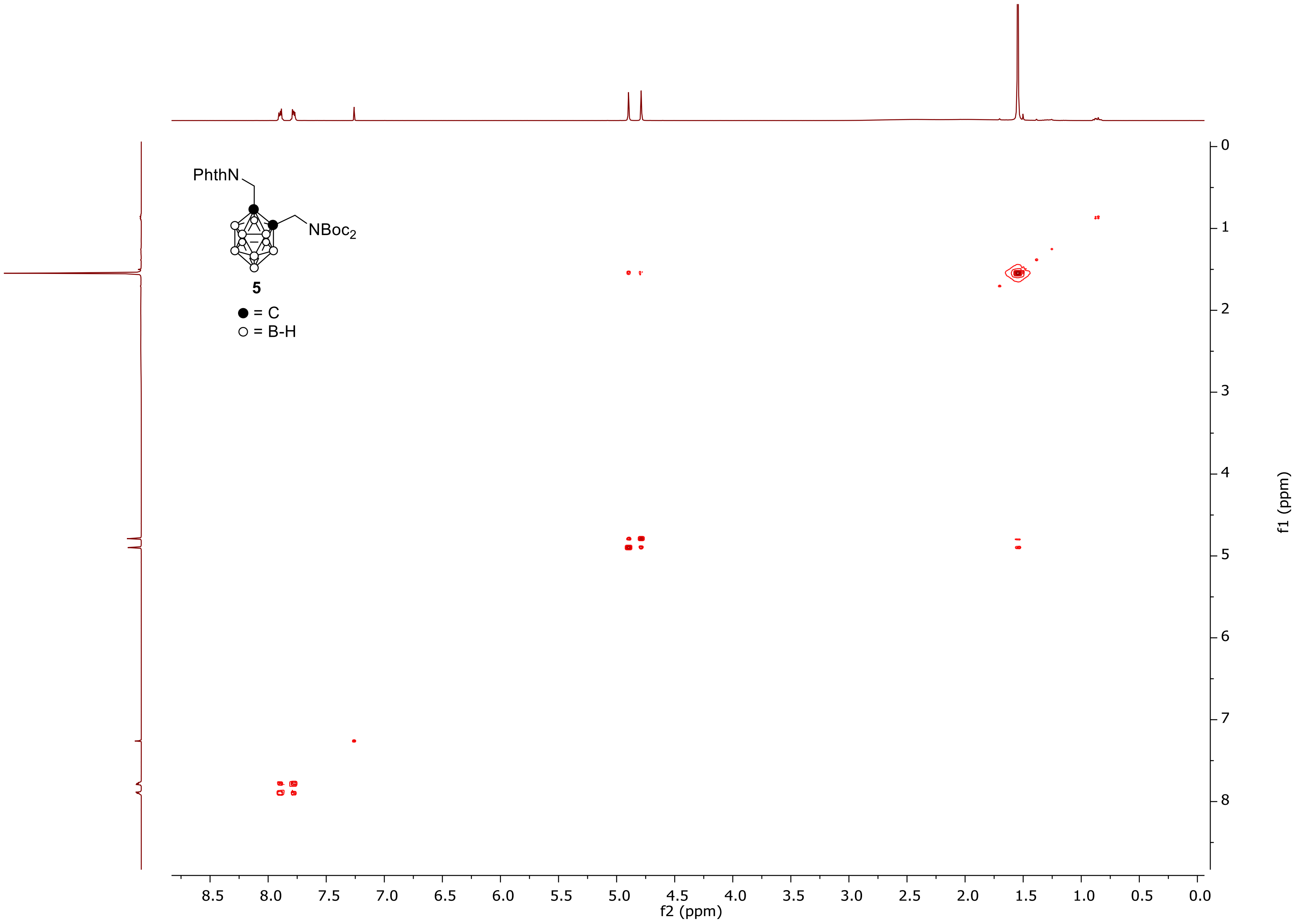

***C*-(*N*-(1,3-dioxoisoindolin-2-yl))methyl-*C'*-2-(*N,N*-di-*tert*-butoxycarbonyl)aminomethyl-*o*-carborane (5).**

^{13}C NMR (100 MHz, $CDCl_3$)

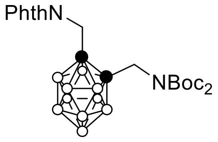

5

● = C
○ = B-H

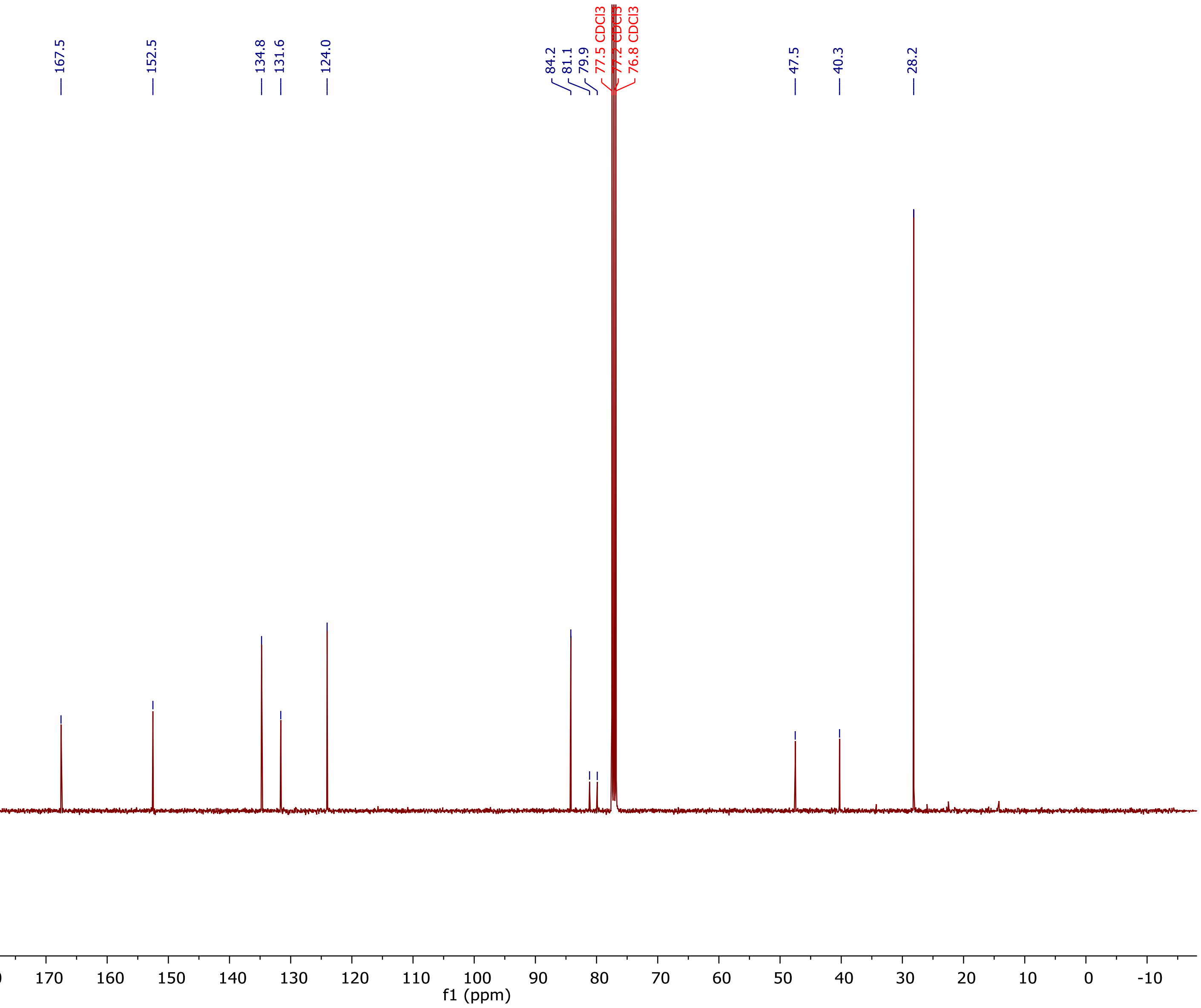

***C*-(*N*-(1,3-dioxoisoindolin-2-yl))methyl-*C'*-2-(*N,N*-di-*tert*-butoxycarbonyl)aminomethyl-*o*-carborane (5).**

Comparison between DEPT 135 and ^{13}C NMR (100 MHz, $CDCl_3$)

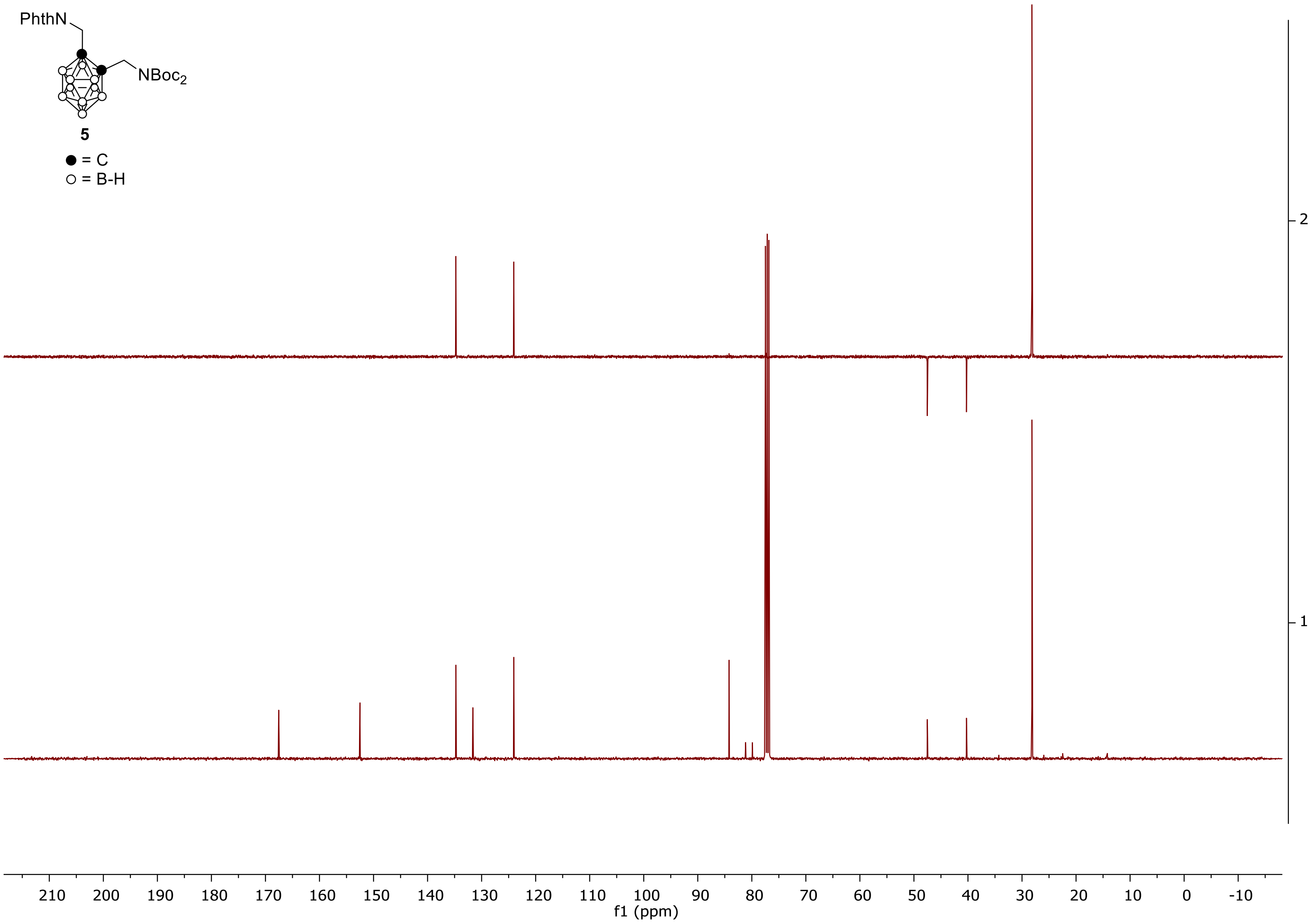

_C_-(_N_-(1,3-dioxoisoindolin-2-yl))methyl-_C'_-2-(_N,N_-di-_tert_-butoxycarbonyl)aminomethyl-_o_-carborane (5).

^{11}B NMR (192.5 MHz, $CDCl_3$)

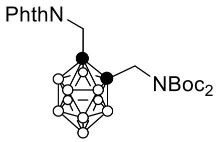

5

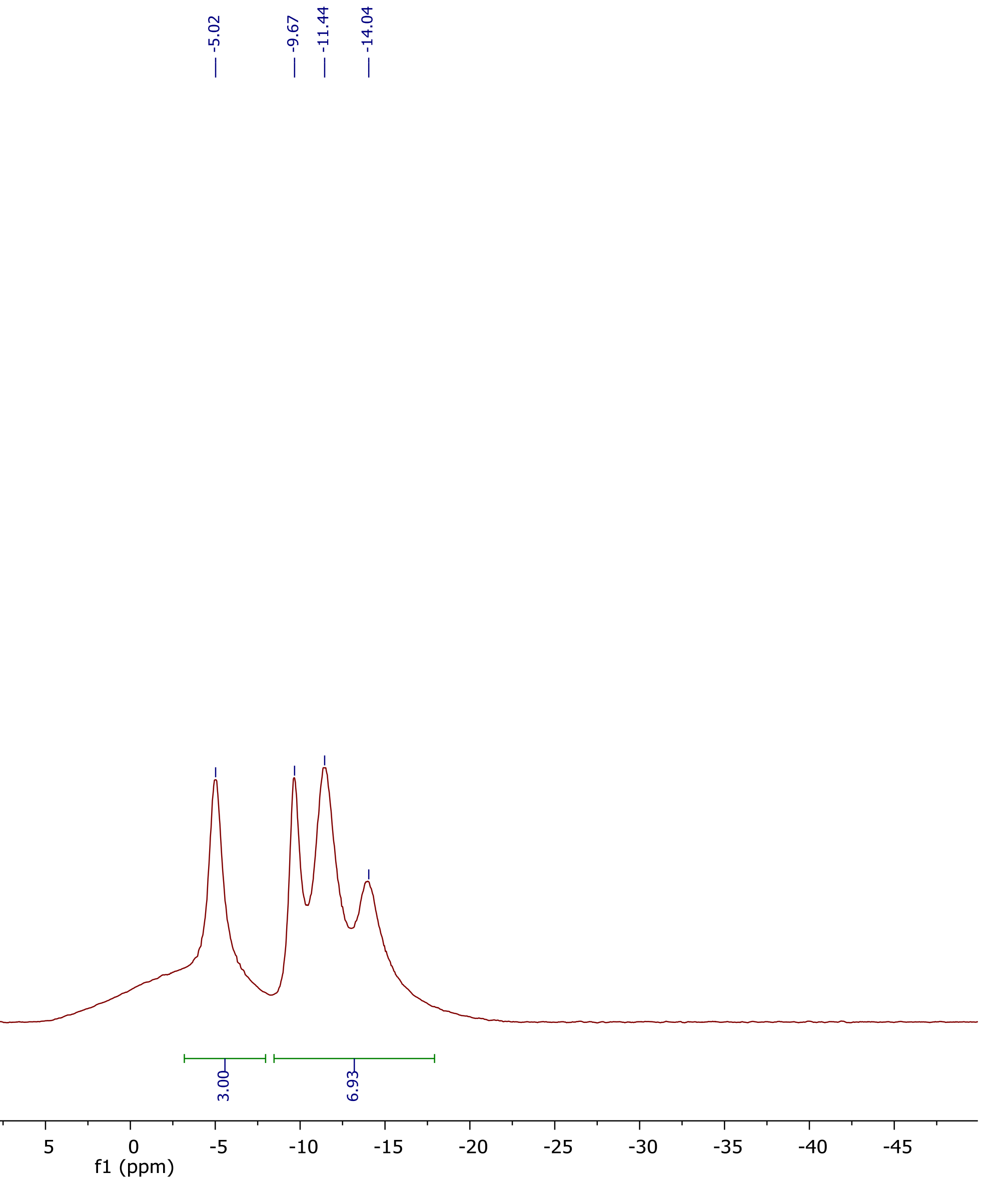

^{10}B-enriched-*C*-(*N*-(1,3-dioxoisoindolin-2-yl))methyl-*C'*-2-(*N,N*-di-*tert*-butoxycarbonyl)aminomethyl-*o*-carborane (^{10}B-5).

^{1}H NMR (400 MHz, $CDCl_3$)

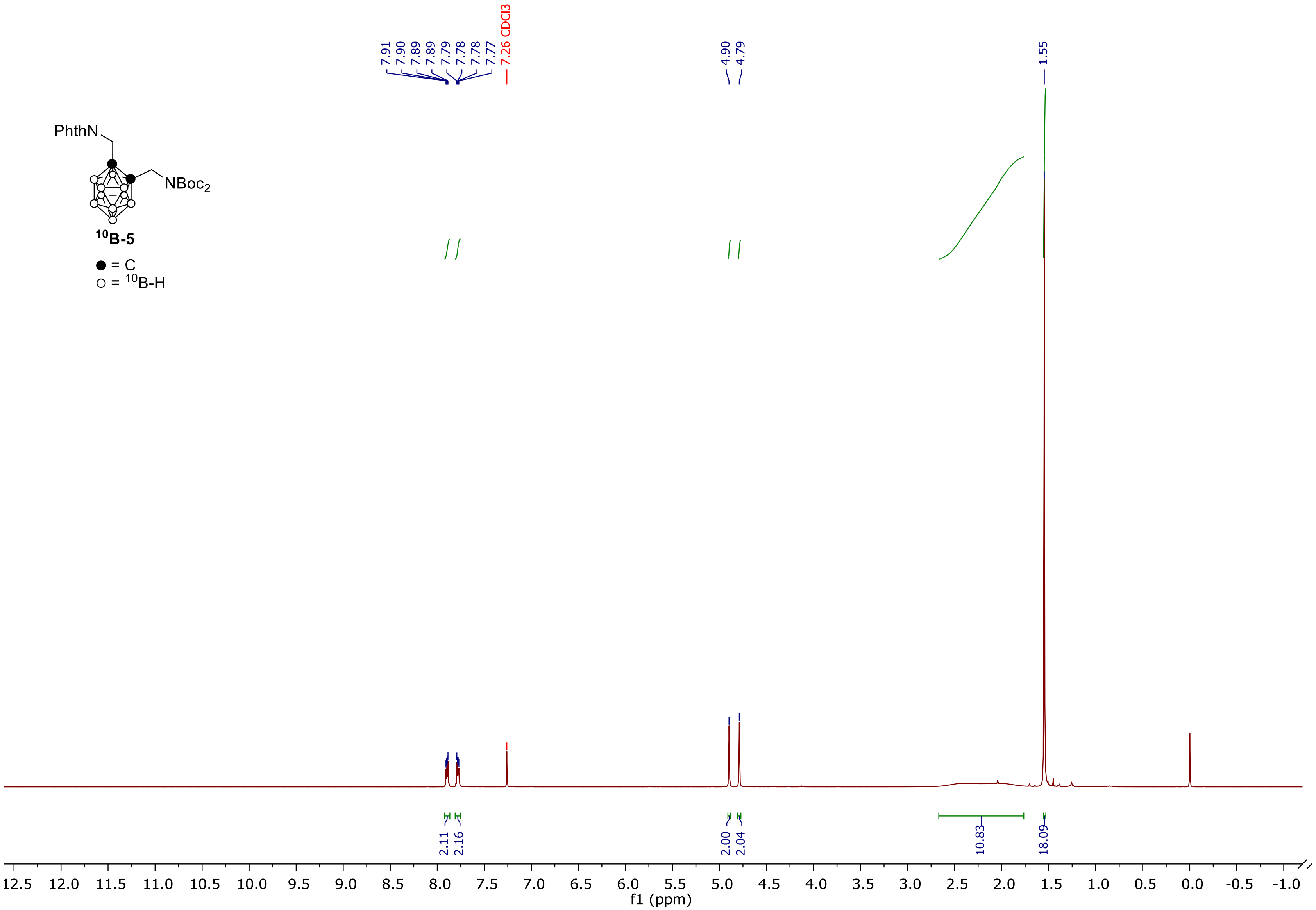

^{10}B-enriched-*C*-(*N*-(1,3-dioxoisoindolin-2-yl))methyl-*C'*-2-(*N,N*-di-*tert*-butoxycarbonyl)aminomethyl-*o*-carborane (^{10}B-5).

^{1}H-COSY NMR (400 MHz, $CDCl_3$)

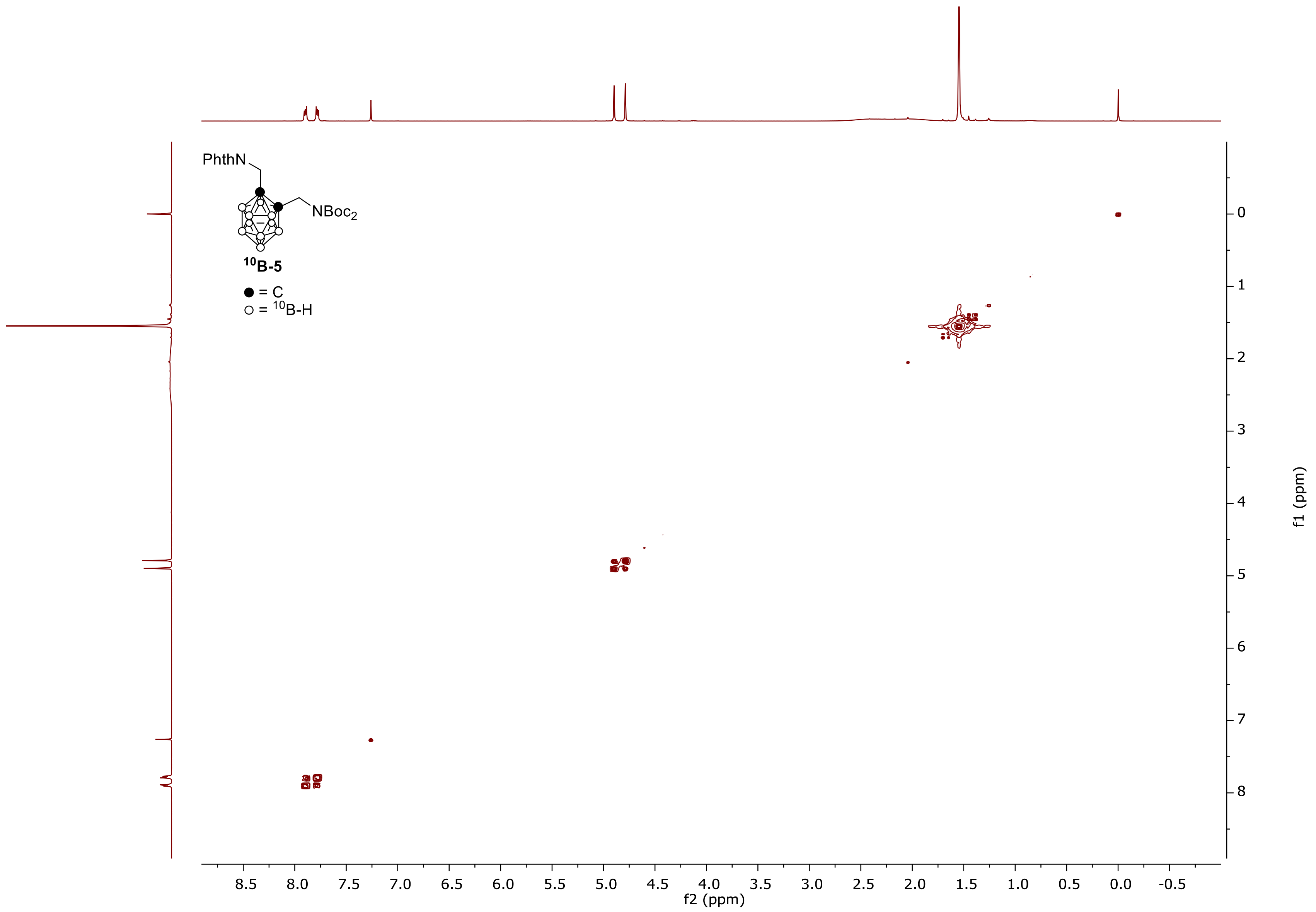

^{10}B-enriched-*C*-(*N*-(1,3-dioxoisoindolin-2-yl))methyl-*C'*-2-(*N,N*-di-*tert*-butoxycarbonyl)aminomethyl-*o*-carborane (^{10}B-5).

^{13}C NMR (100 MHz, $CDCl_3$)

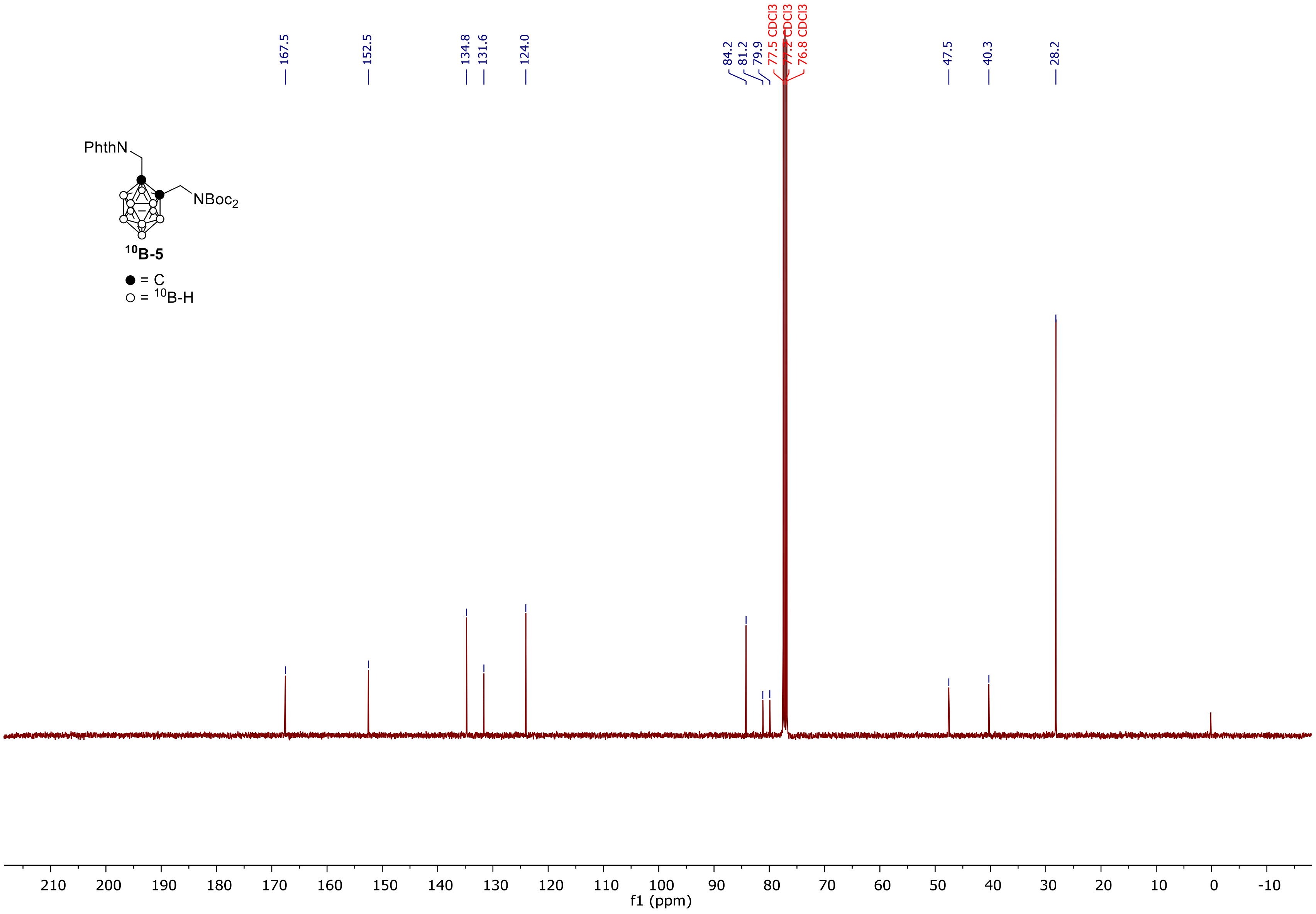

^{10}B-enriched-*C*-(*N*-(1,3-dioxoisoindolin-2-yl))methyl-*C'*-2-(*N,N*-di-*tert*-butoxycarbonyl)aminomethyl-*o*-carborane (^{10}B-5).

Comparison between DEPT 135 and ^{13}C NMR (100 MHz, $CDCl_3$)

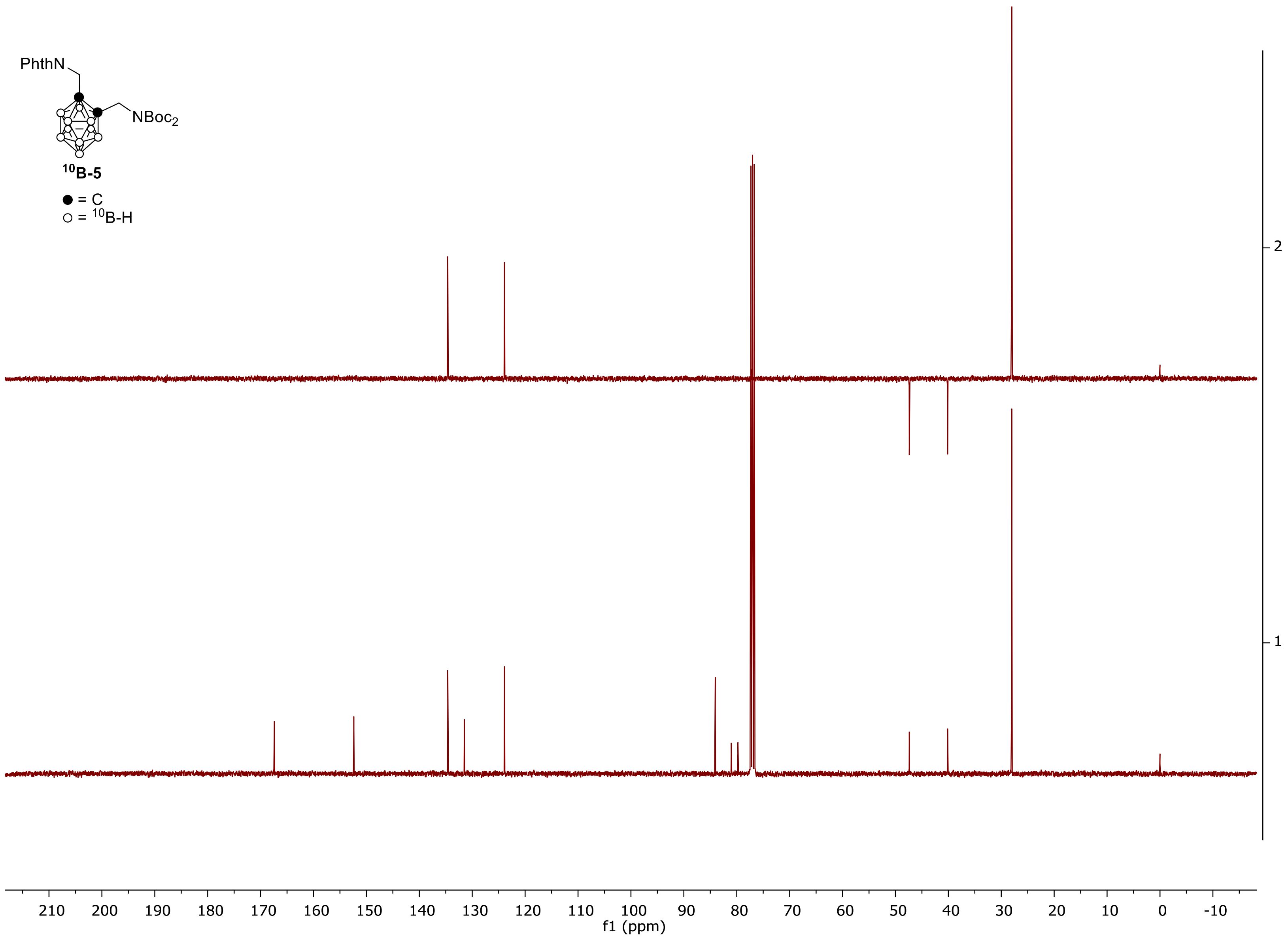

***C*-(*N*-(1,3-dioxoisoindolin-2-yl))methyl-*C'*-2-aminomethyl-*o*-carborane hydrochloric acid salt (6•HCl).**

^{1}H NMR (400 MHz, CD_3OD)

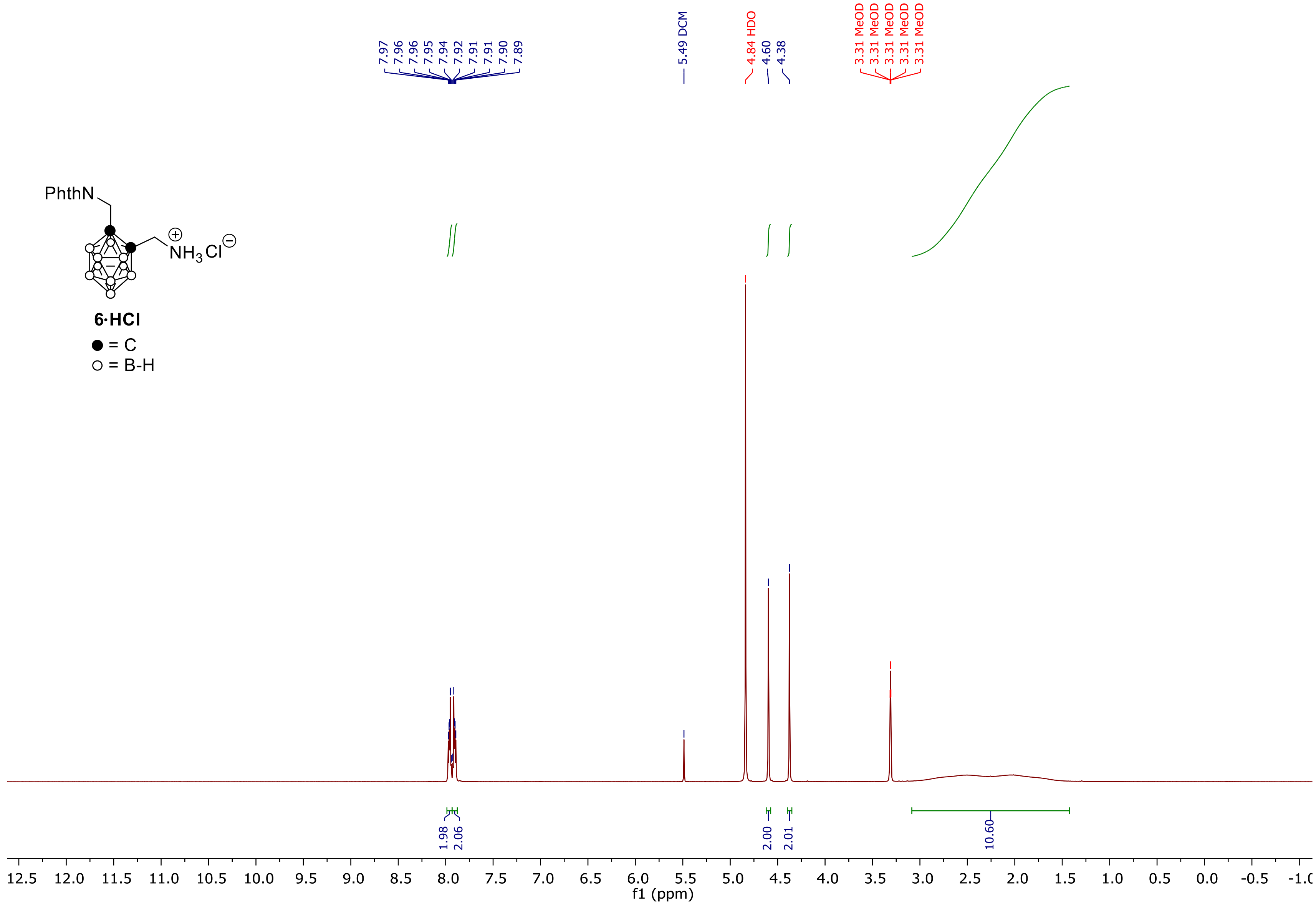

6·HCl

● = C

○ = B-H

C-(N-(1,3-dioxoisoindolin-2-yl))methyl-C'-2-aminomethyl-o-carborane hydrochloric acid salt (6•HCl).

^{1}H-COSY NMR (400 MHz, CD_3OD)

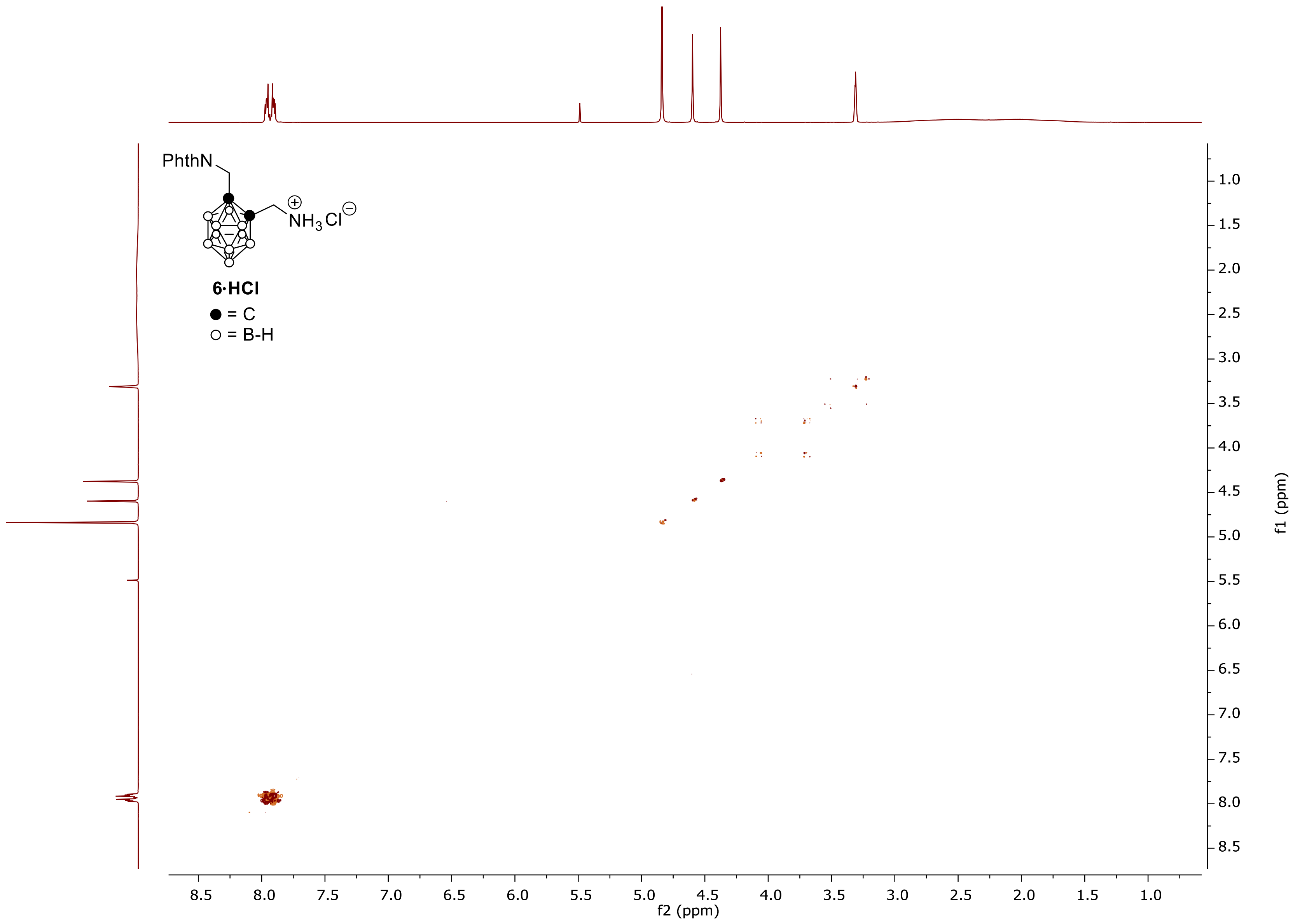

_C_-(_N_-(1,3-dioxoisoindolin-2-yl))methyl-_C'_-2-aminomethyl-_o_-carborane hydrochloric acid salt (6•HCl).

^{13}C NMR (100 MHz, CD_3OD)

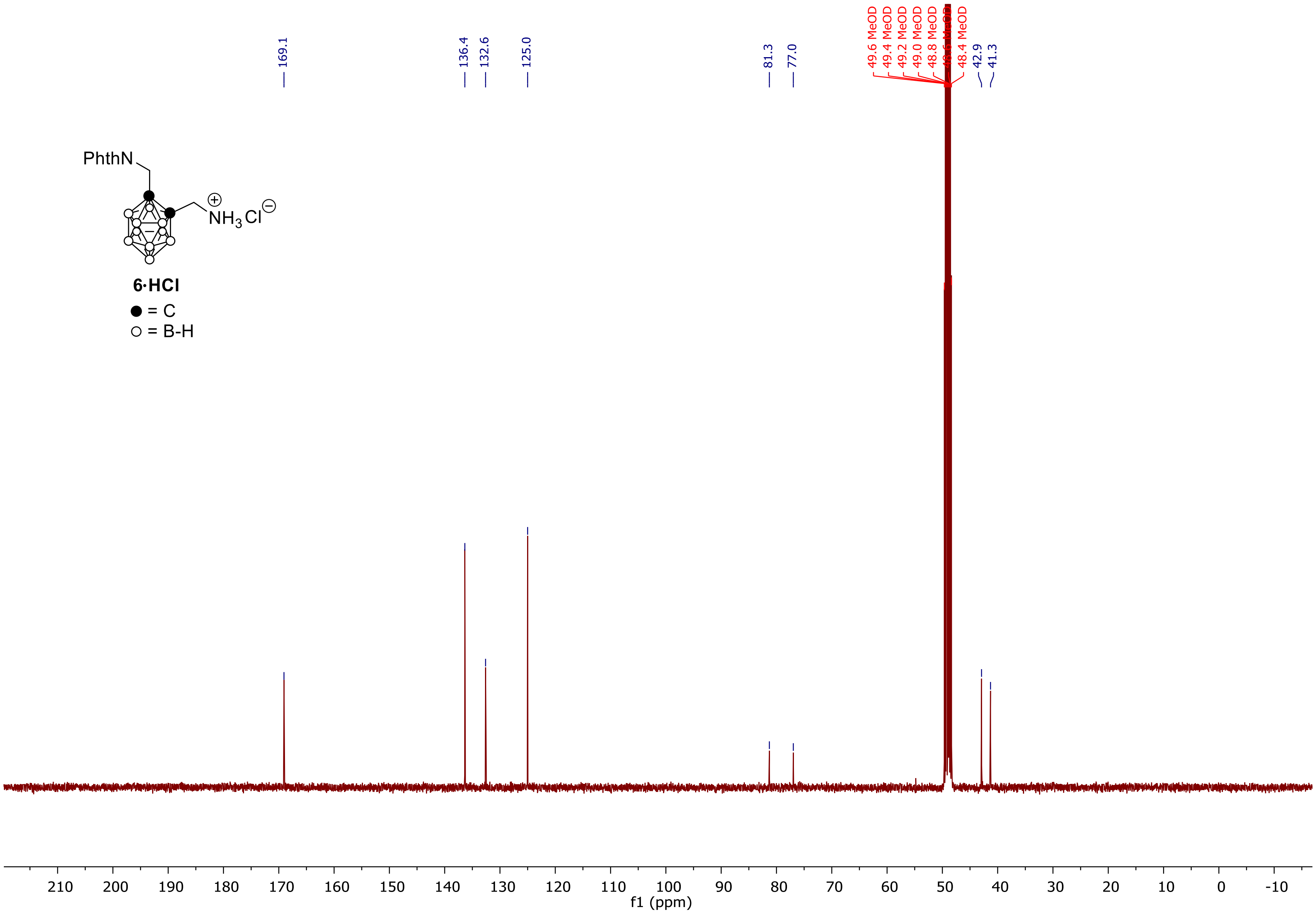

C-(N-(1,3-dioxoisoindolin-2-yl))methyl-C'-2-aminomethyl-o-carborane hydrochloric acid salt (6•HCl).

Comparison between DEPT 135 and ^{13}C NMR (100 MHz, CD_3OD)

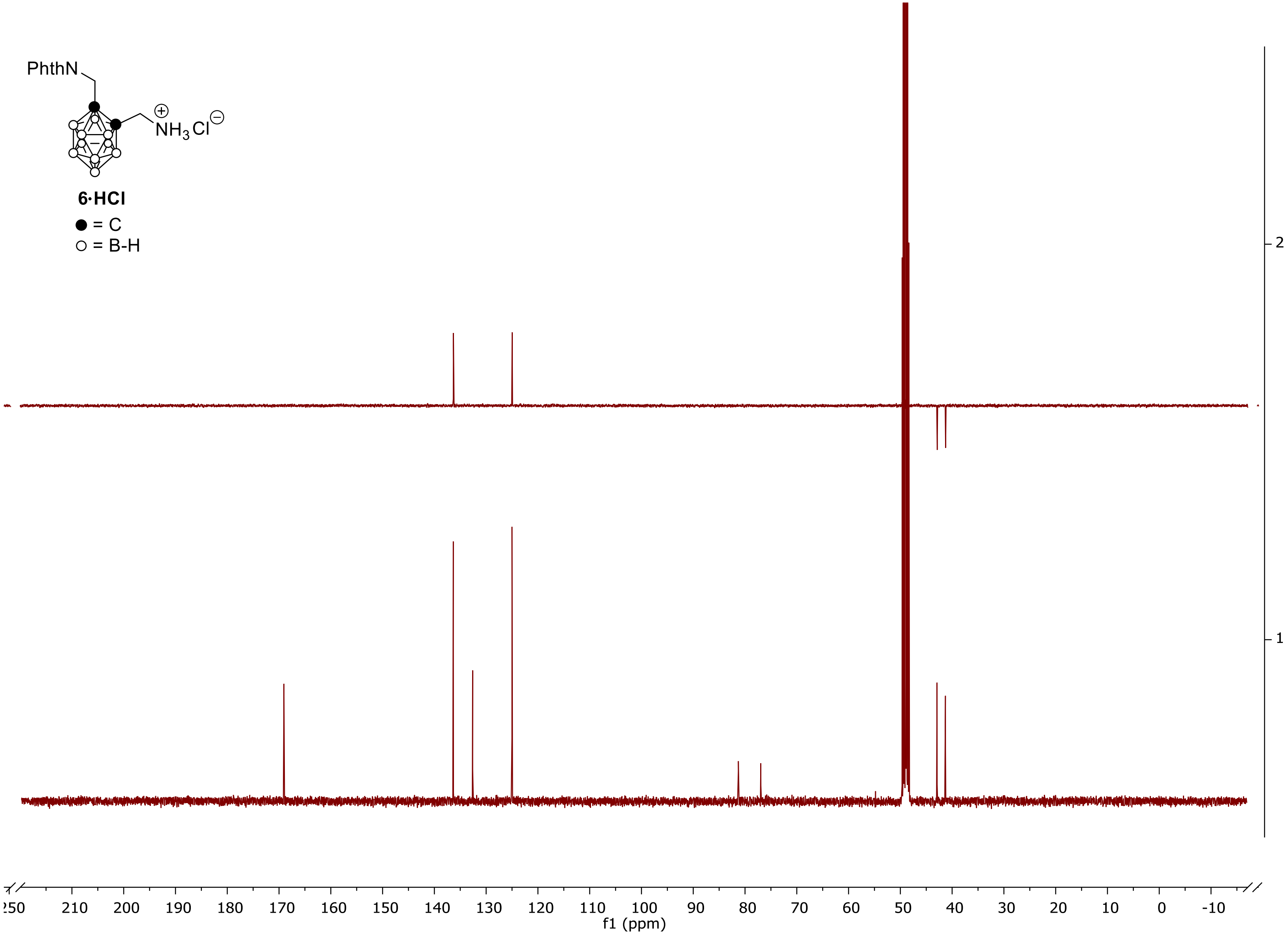

C-(N-(1,3-dioxoisoindolin-2-yl))methyl-C′-2-aminomethyl-o-carborane hydrochloric acid salt (6•HCl).

^{11}B NMR (128 MHz, CD_3OD)

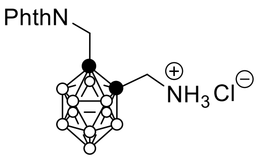

6·HCl

● = C

○ = B-H

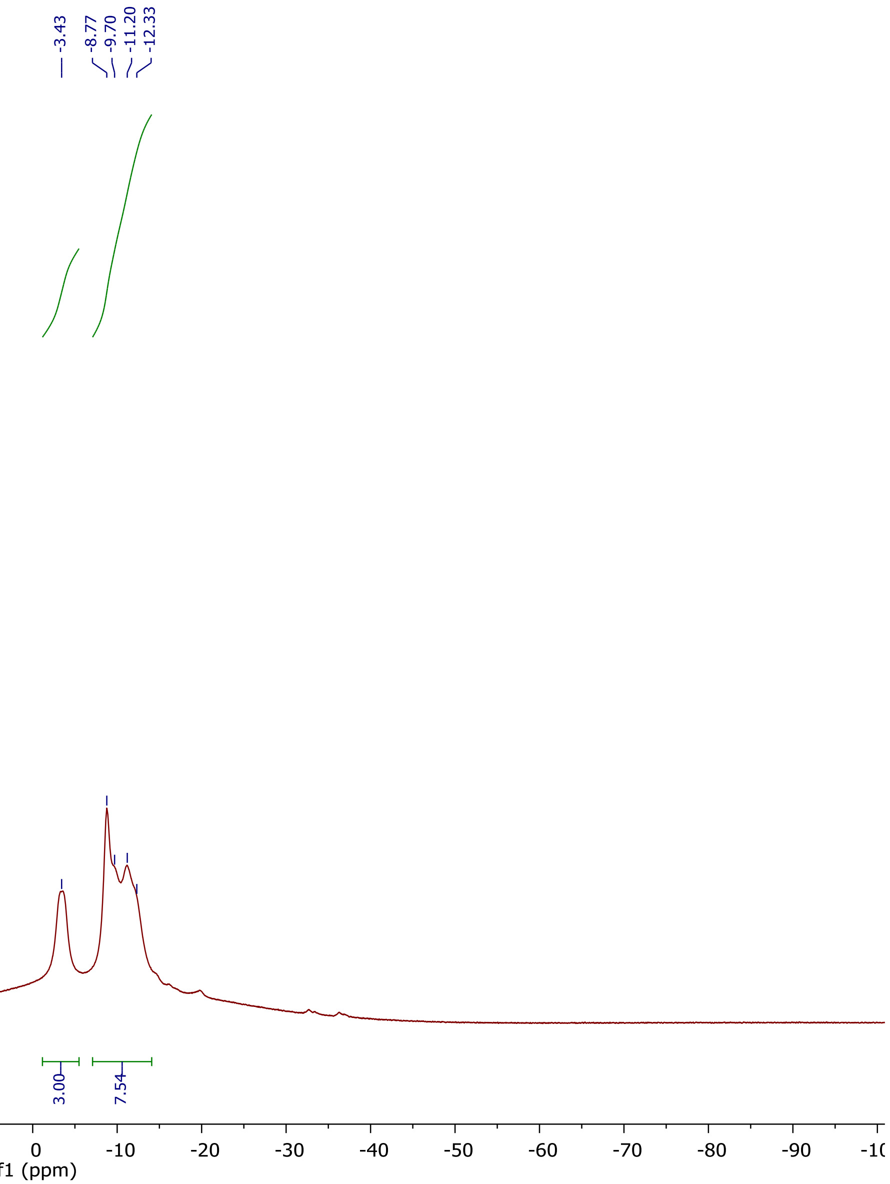

^{10}B-enriched-*C*-(*N*-(1,3-dioxoisoindolin-2-yl))methyl-*C'*-2-aminomethyl-*o*-carborane hydrochloric acid salt (^{10}B-6•HCl).

^{1}H NMR (400 MHz, CD_3OD)

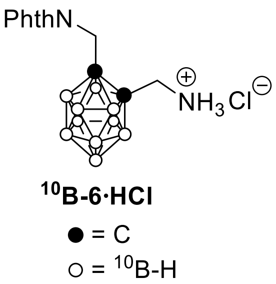

^{10}B-6·HCl

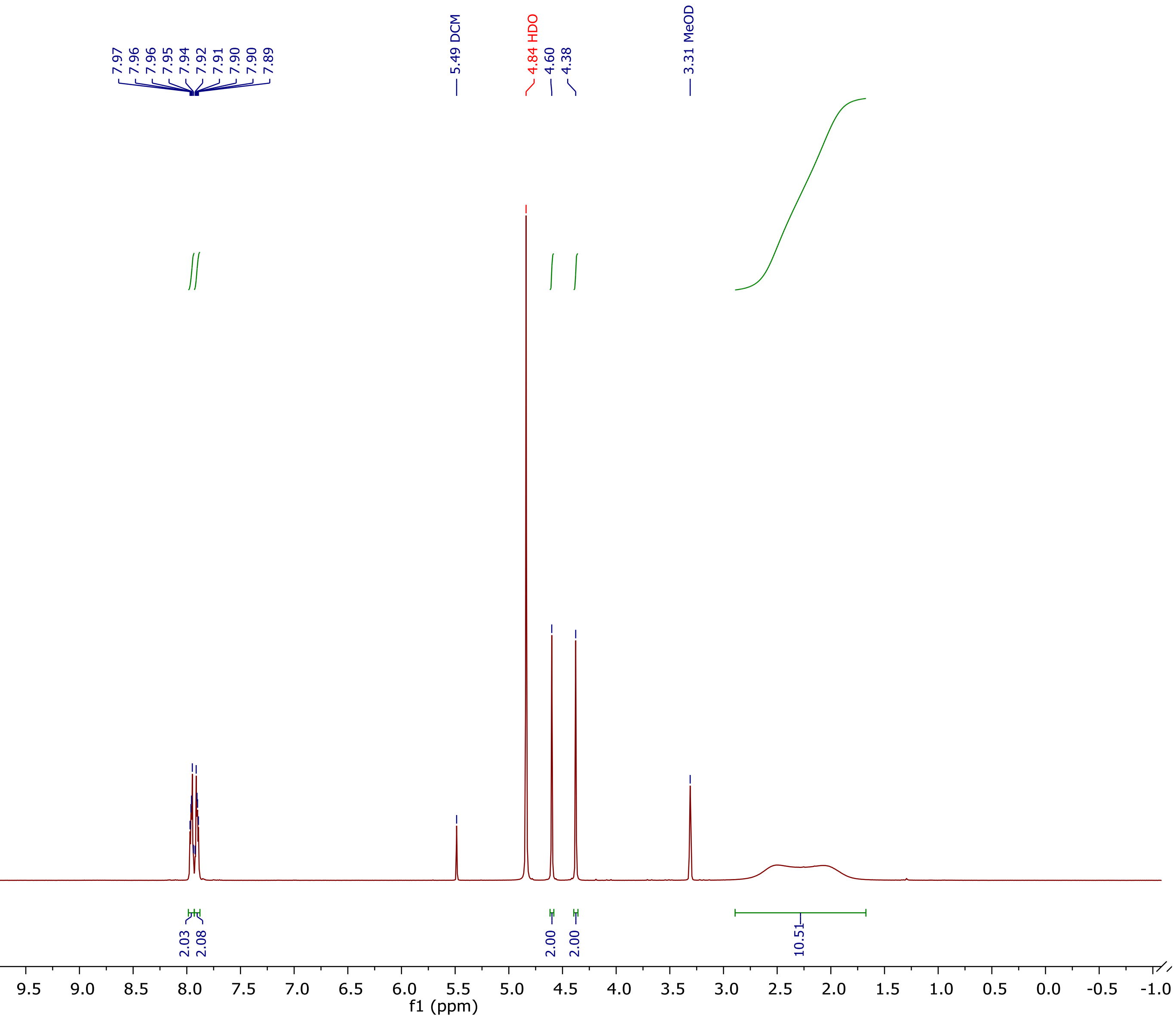

^{10}B-enriched-*C*-(*N*-(1,3-dioxoisoindolin-2-yl))methyl-*C'*-2-aminomethyl-*o*-carborane hydrochloric acid salt (^{10}B-6•HCl).

^{1}H-COSY NMR (400 MHz, CD_3OD)

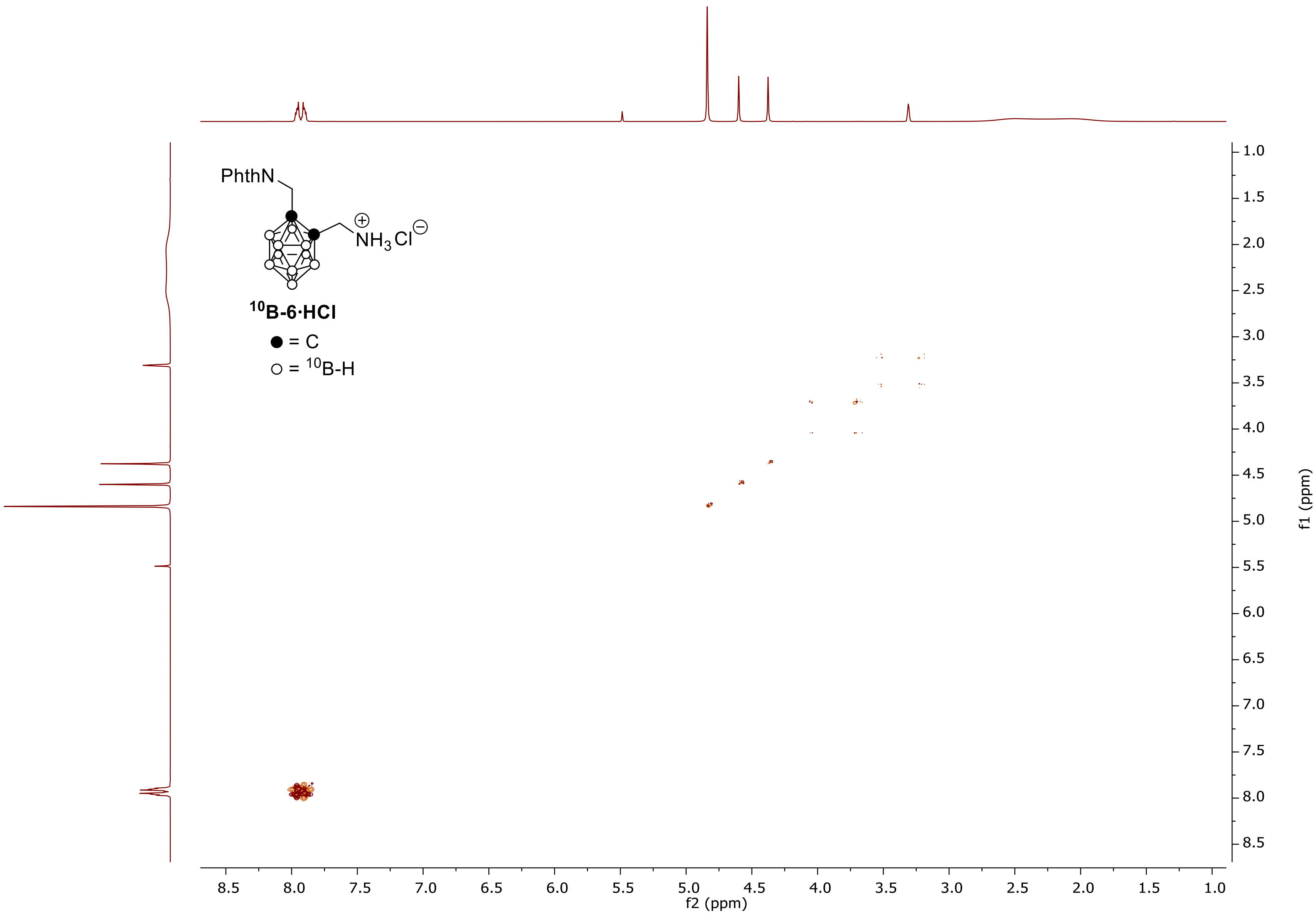

^{10}B-enriched-*C*-(*N*-(1,3-dioxoisoindolin-2-yl))methyl-*C'*-2-aminomethyl-*o*-carborane hydrochloric acid salt (^{10}B-6•HCl).

^{13}C NMR (100 MHz, CD_3OD)

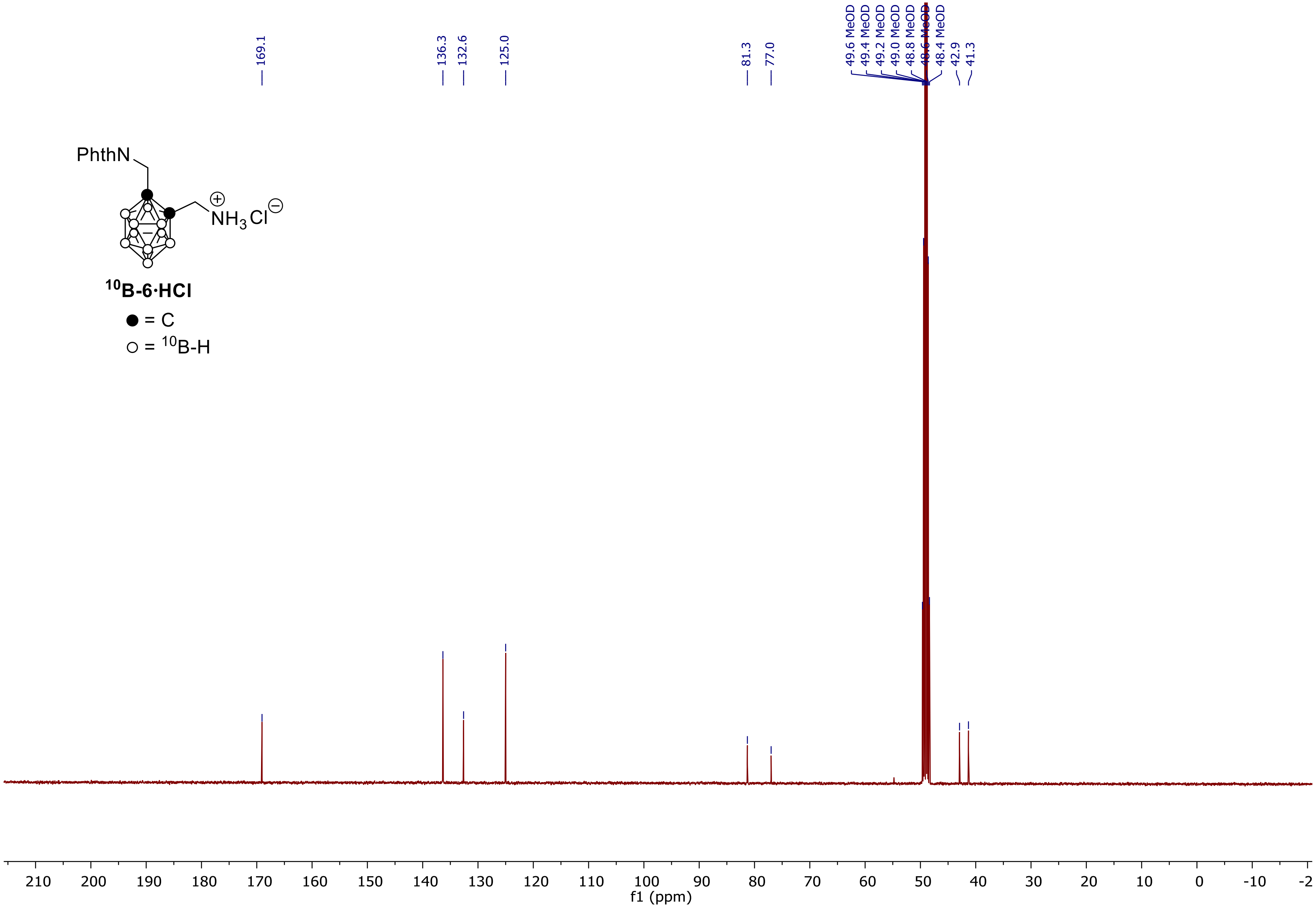

^{10}B-enriched-*C*-(*N*-(1,3-dioxoisoindolin-2-yl))methyl-*C'*-2-aminomethyl-*o*-carborane hydrochloric acid salt (^{10}B-6•HCl).

Comparison between DEPT 135 and ^{13}C NMR (100 MHz, CD_3OD)

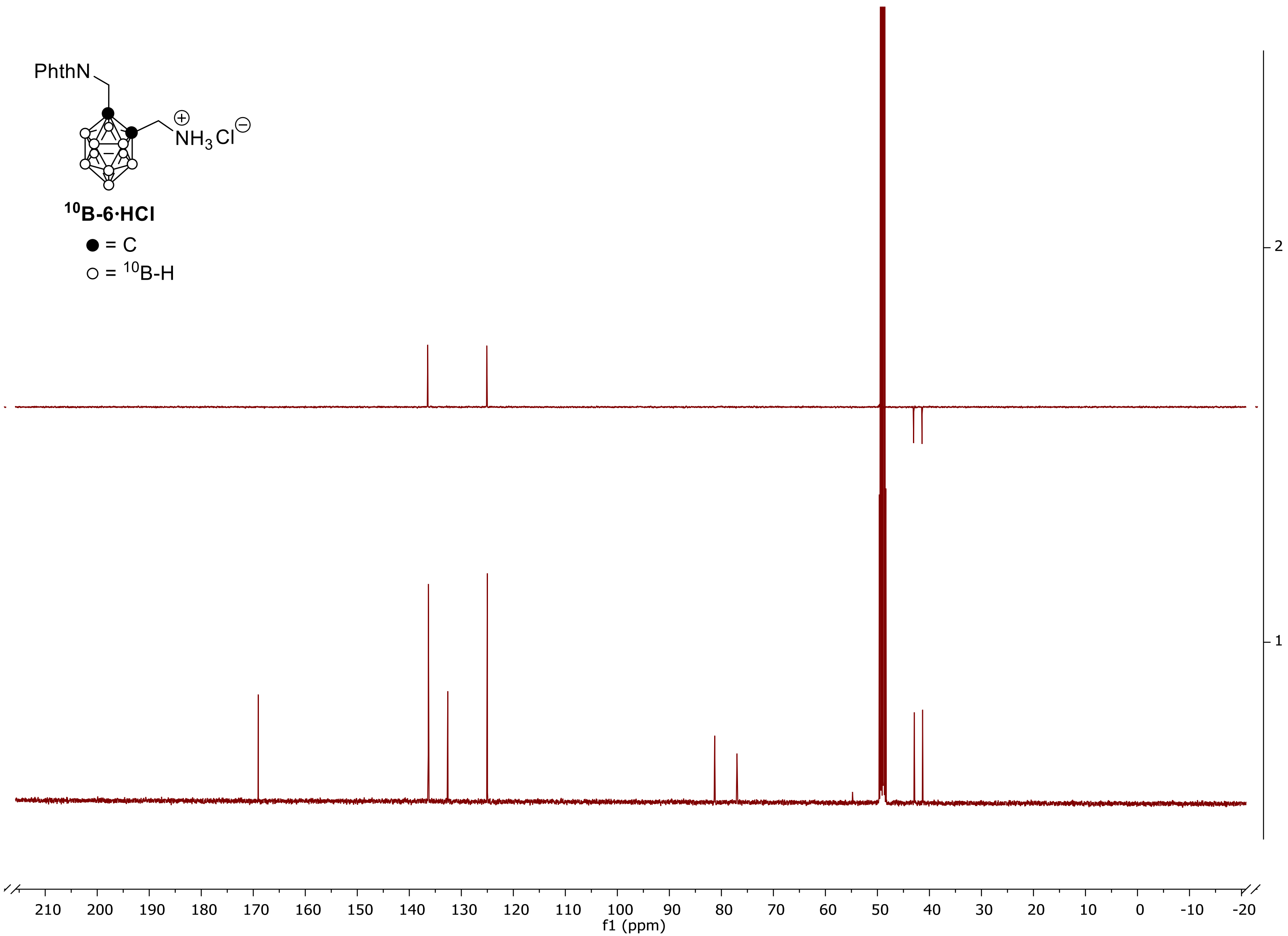

_C_-(_N_-(1,3-dioxoisoindolin-2-yl))methyl-_C'_-(sulfamoylamino)methyl-_o_-carborane (7).

^{1}H NMR (400 MHz, acetone-*d6*)

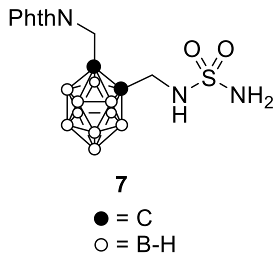

7

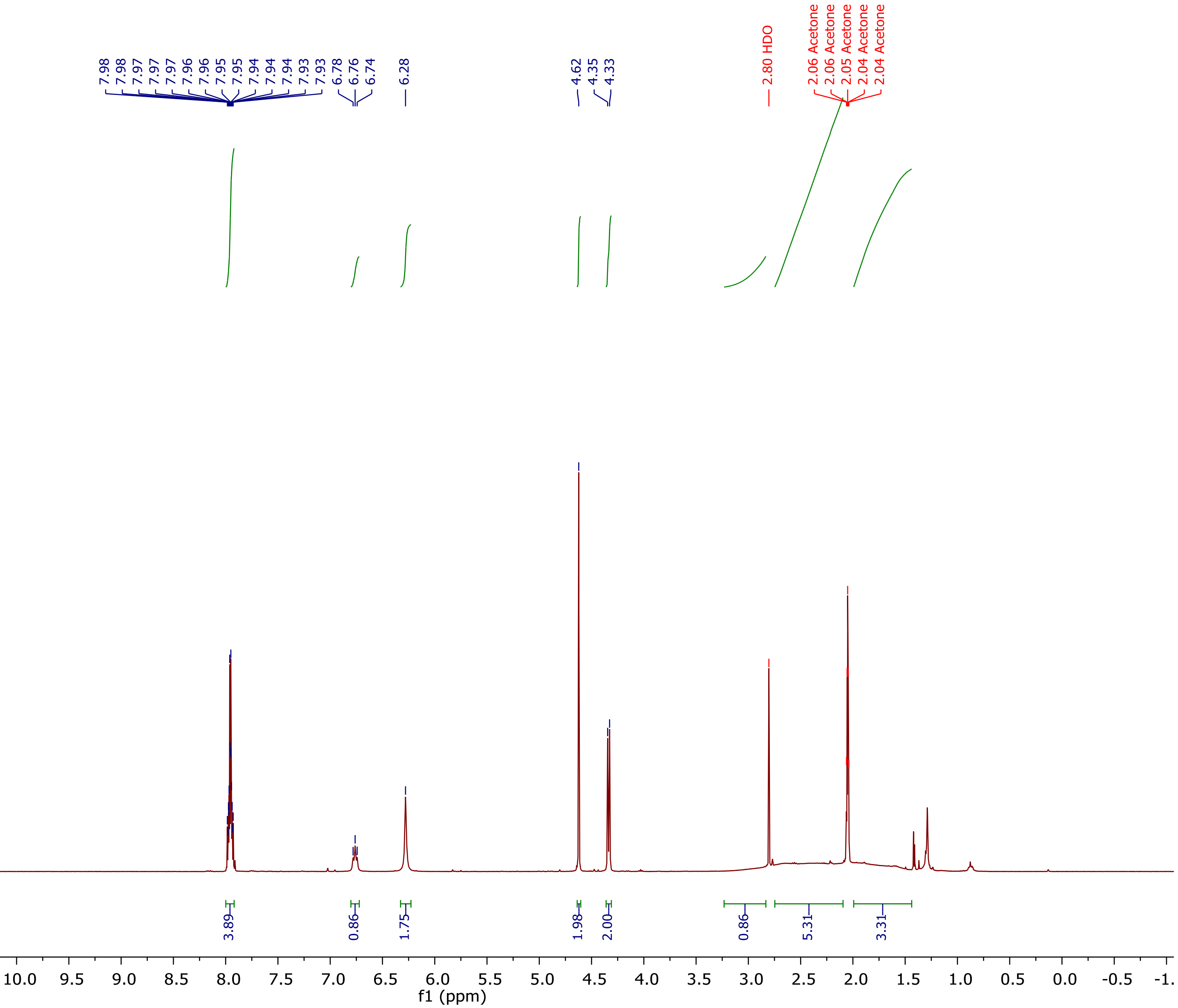

C-(N-(1,3-dioxoisoindolin-2-yl))methyl-C'-(sulfamoylamino)methyl-o-carborane (7).

^{1}H-COSY NMR (400 MHz, acetone-*d6*)

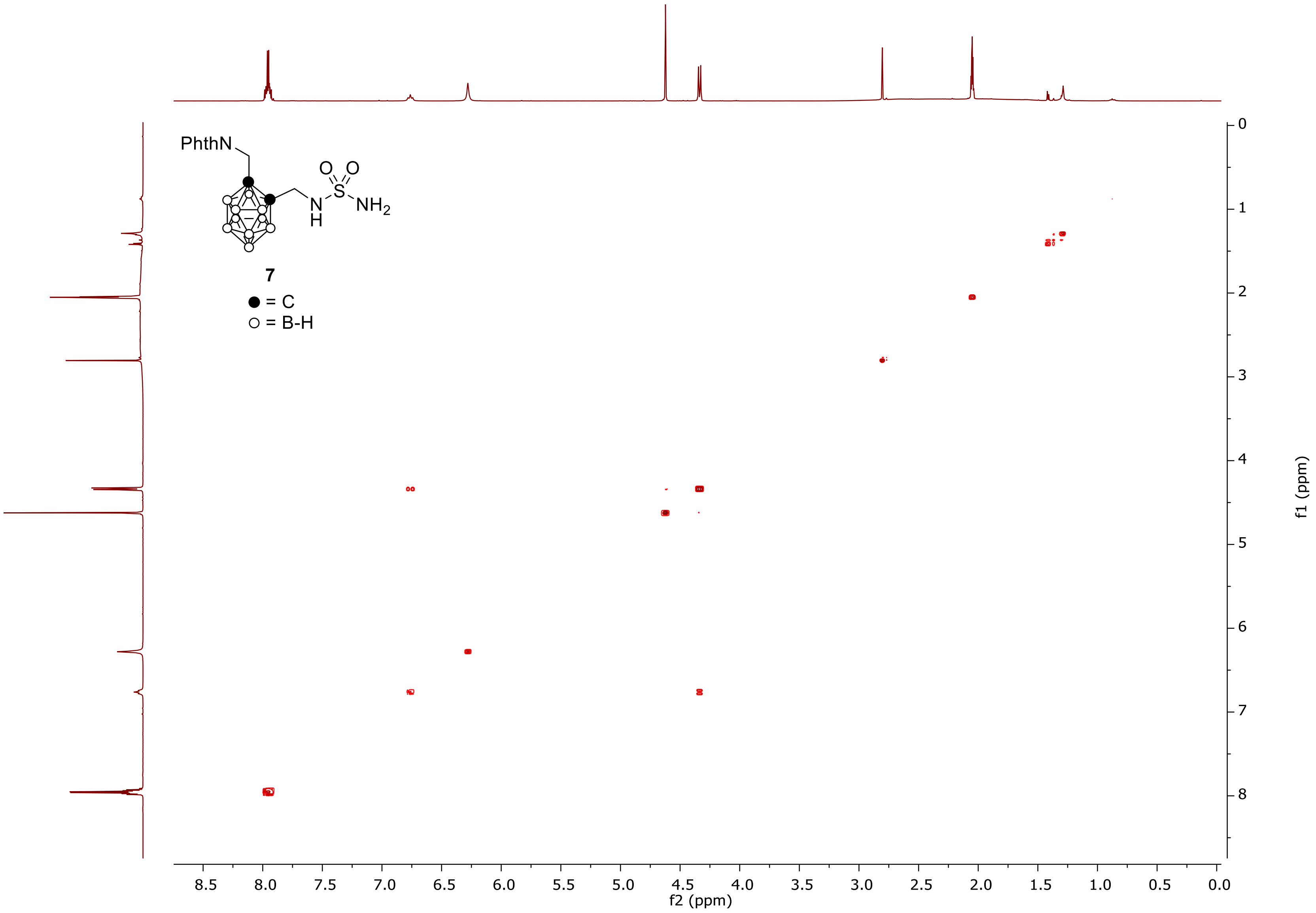

_C_-(_N_-(1,3-dioxoisoindolin-2-yl))methyl-_C'_-(sulfamoylamino)methyl-_o_-carborane (7).

^{13}C NMR (100 MHz, acetone-*d6*)

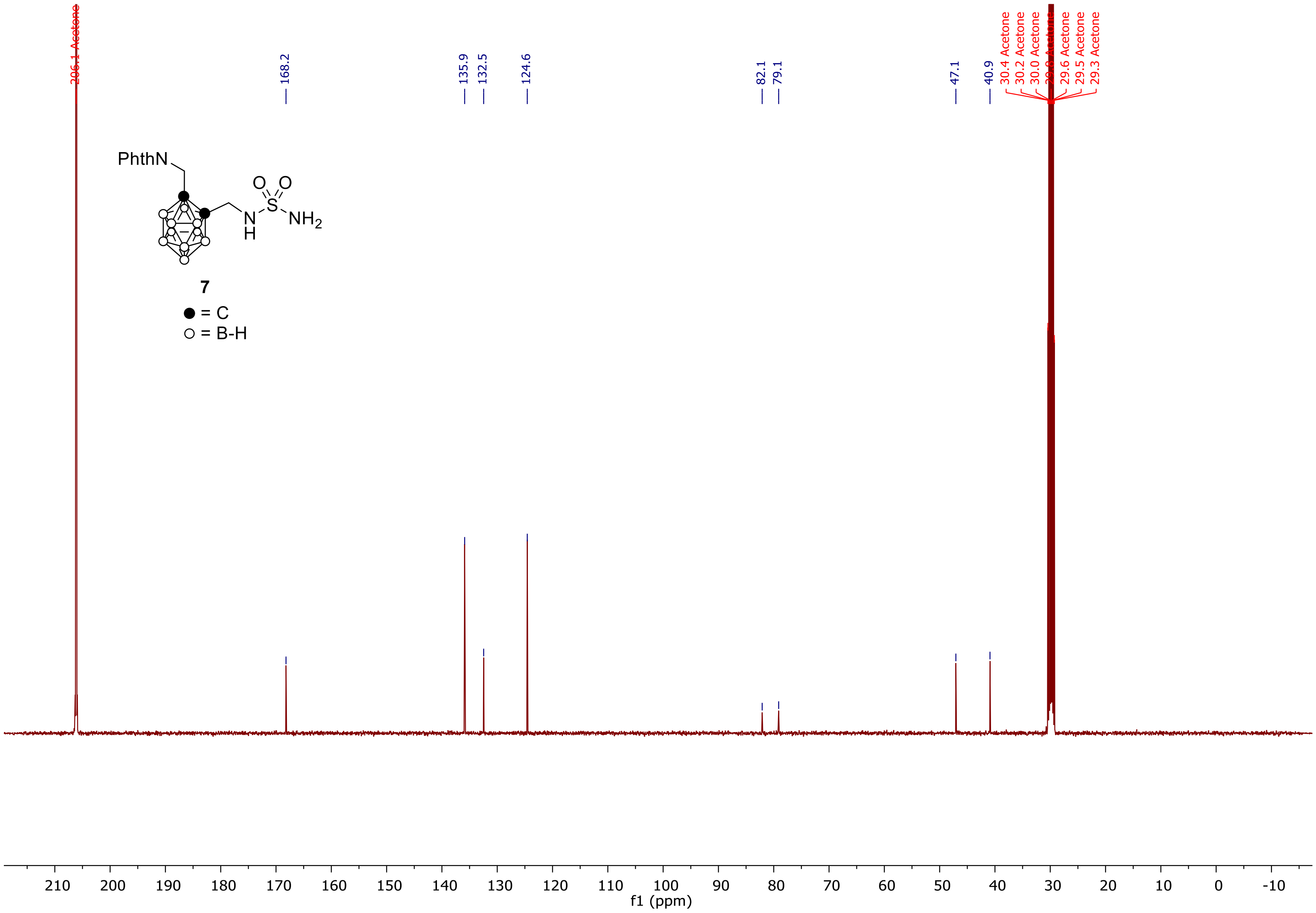

_C_-(_N_-(1,3-dioxoisoindolin-2-yl))methyl-_C'_-(sulfamoylamino)methyl-_o_-carborane (7).

Comparison between DEPT 135 and ^{13}C NMR (100 MHz, acetone-*d6*)

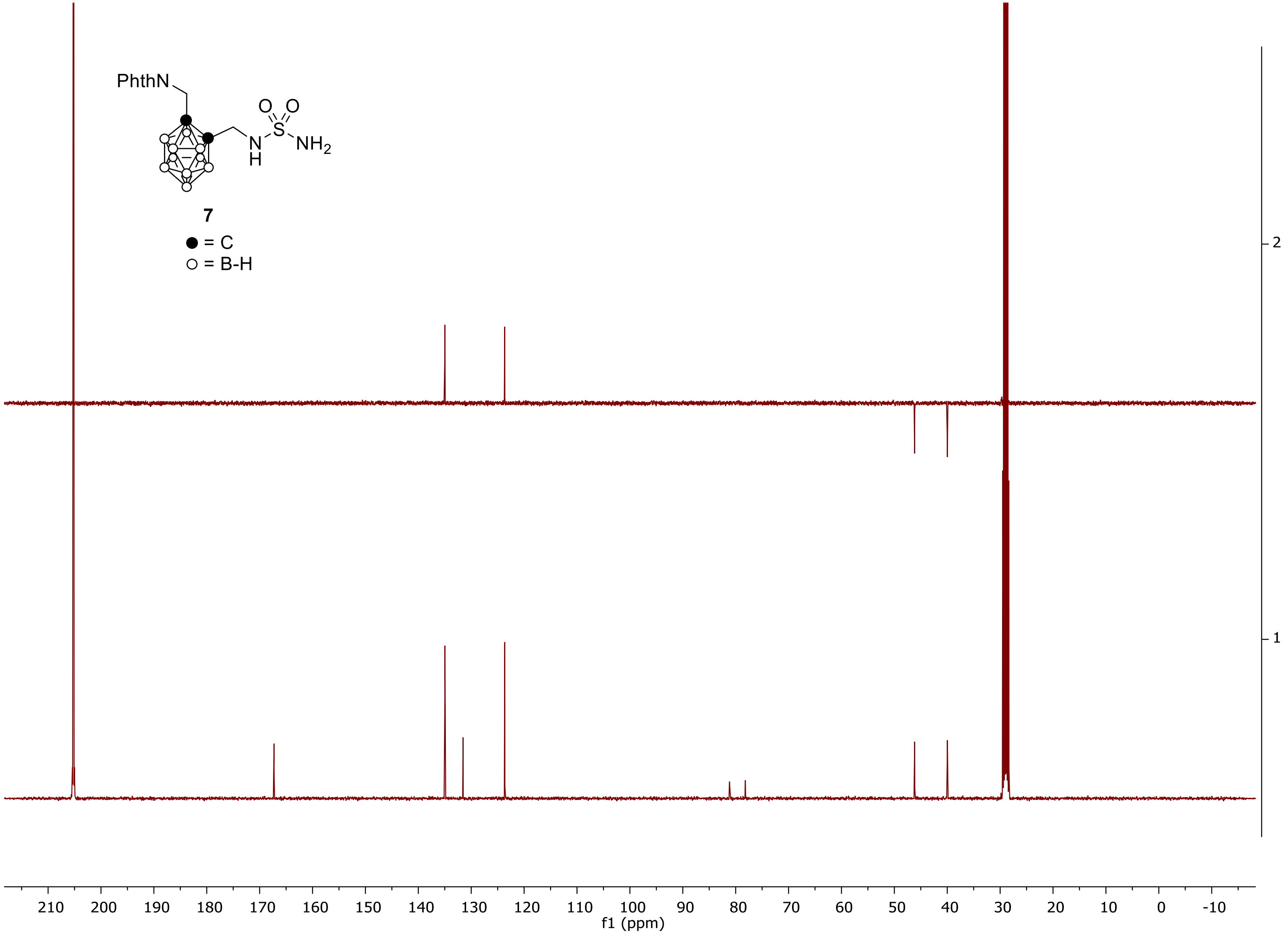

***C*-(*N*-(1,3-dioxoisoindolin-2-yl))methyl-*C'*-(sulfamoylamino)methyl-*o*-carborane (7).**

^{11}B NMR (128 MHz, acetone-*d6*)

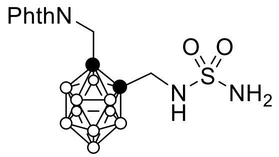

7

● = C
○ = B-H

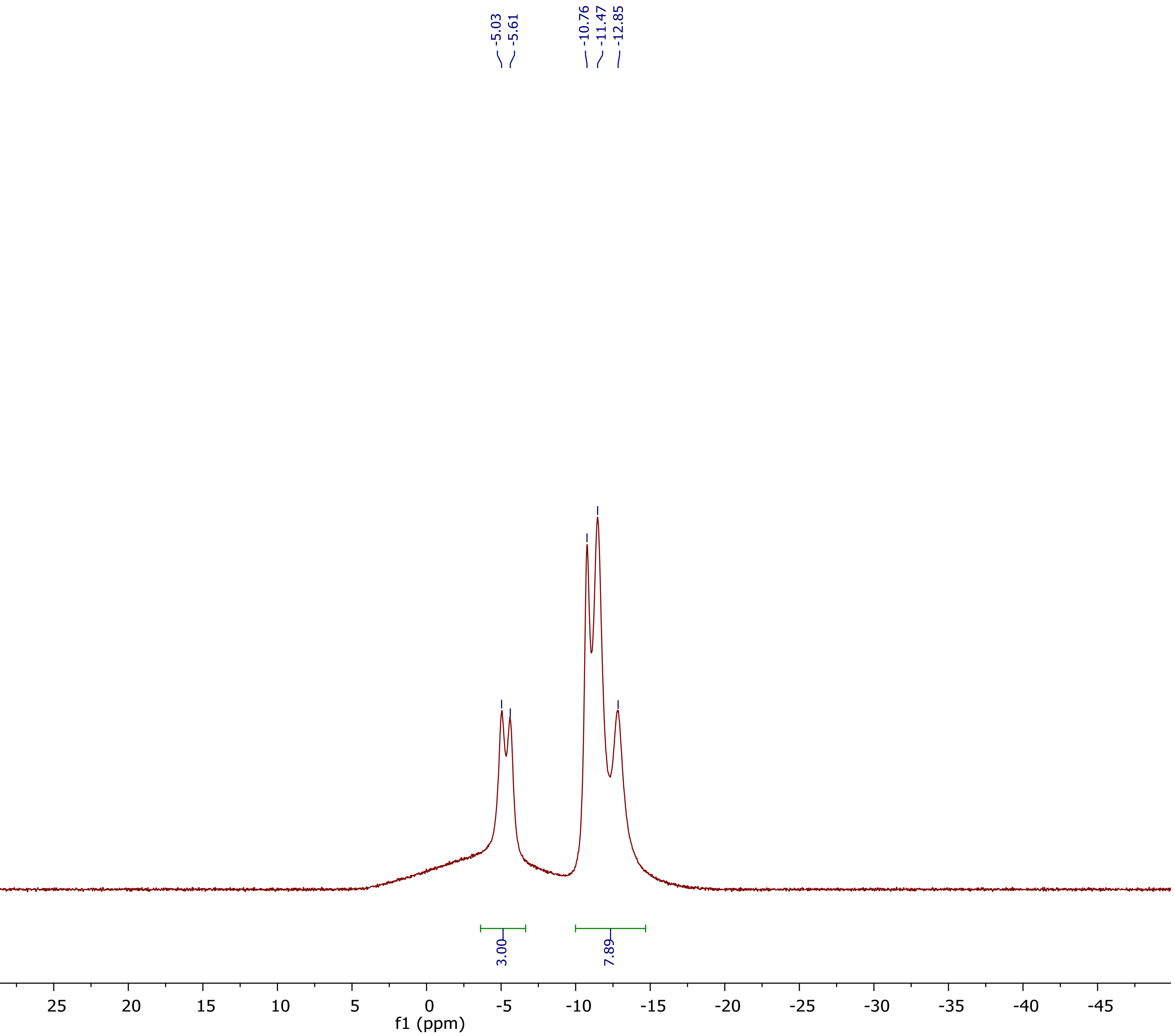

^{10}B-enriched-*C*-(*N*-(1,3-dioxoisoindolin-2-yl))methyl-*C'*-(sulfamoylamino)methyl-*o*-carborane (^{10}B-7).

^{1}H NMR (400 MHz, $CDCl_3$)

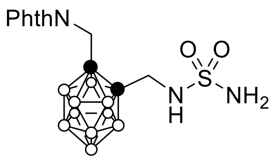

^{10}B-7

● = C

○ = ^{10}B-H

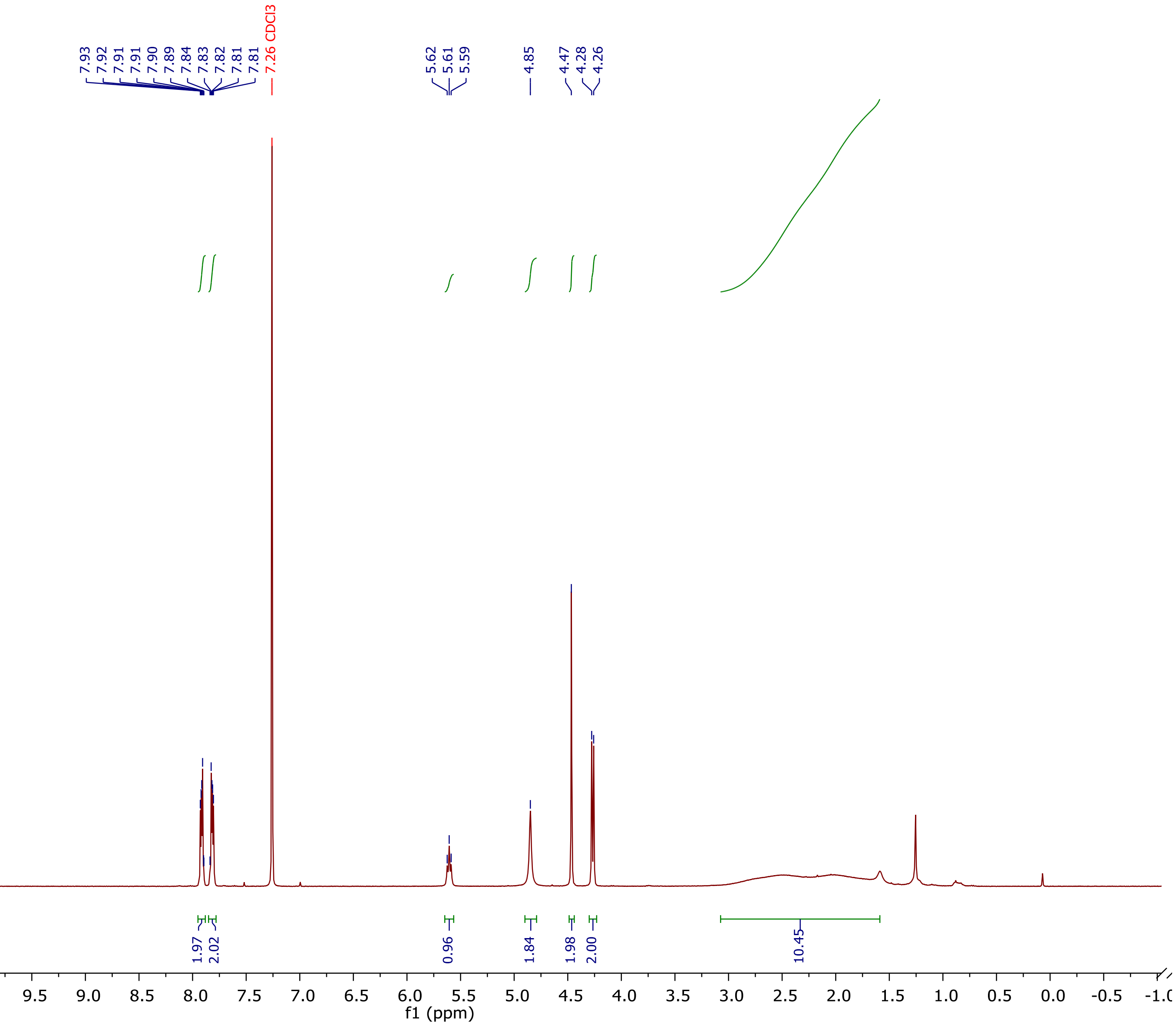

^{10}B-enriched-*C*-(*N*-(1,3-dioxoisoindolin-2-yl))methyl-*C'*-(sulfamoylamino)methyl-*o*-carborane (^{10}B-7).

^{1}H-COSY NMR (400 MHz, $CDCl_3$)

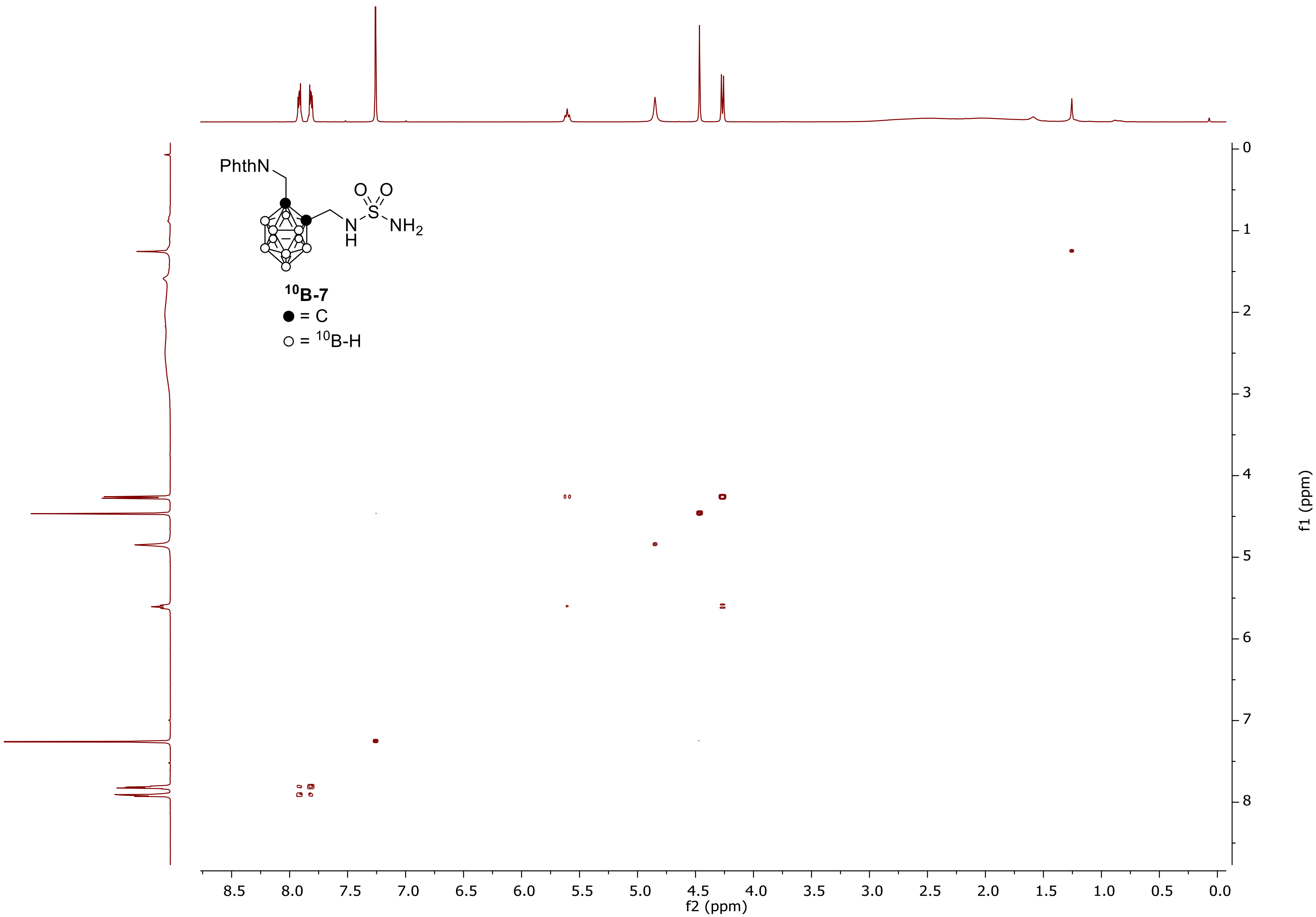

^{10}B-enriched-*C*-(*N*-(1,3-dioxoisoindolin-2-yl))methyl-*C'*-(sulfamoylamino)methyl-*o*-carborane (^{10}B-7).

^{13}C NMR (100 MHz, acetone-*d6*)

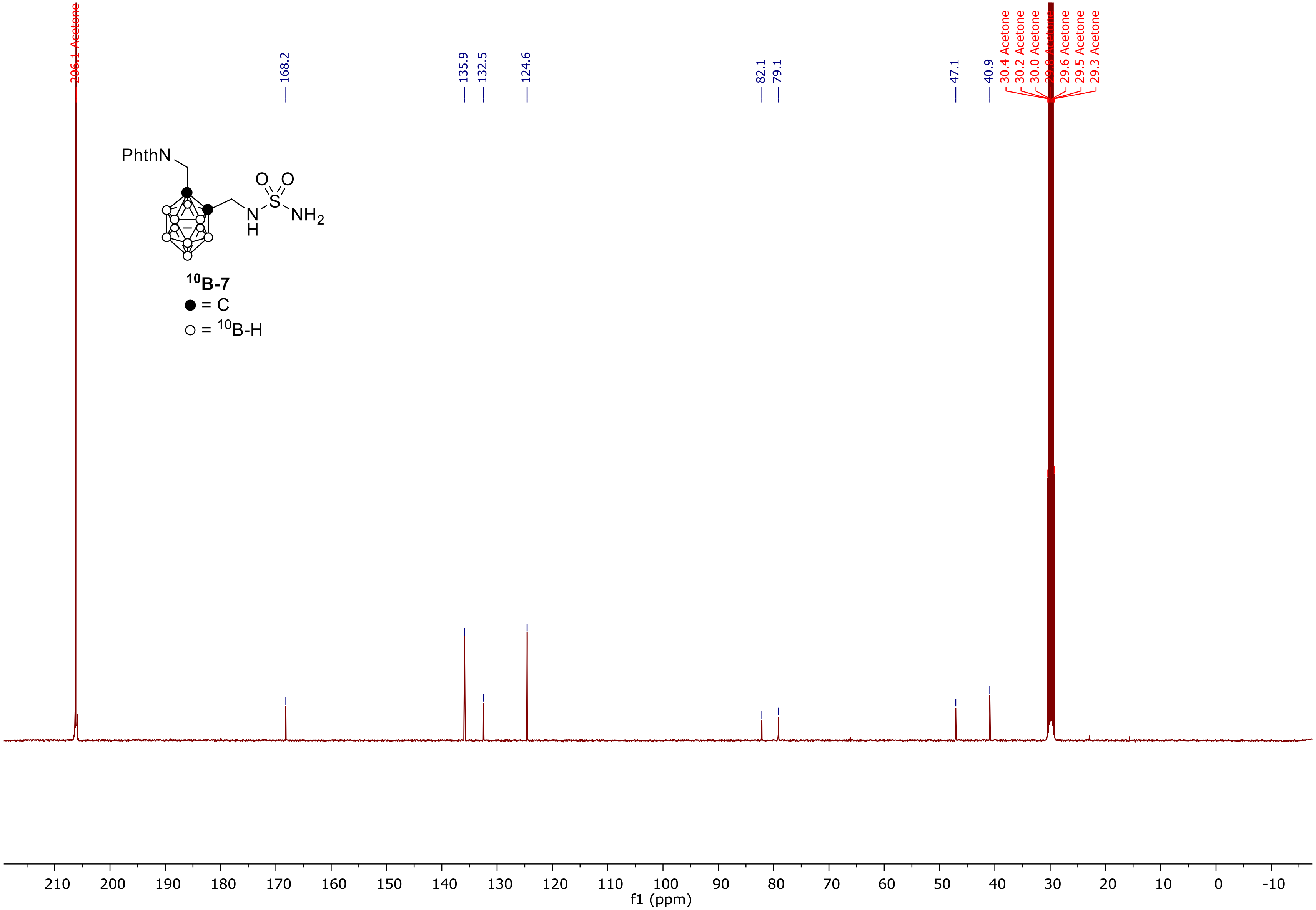

^{10}B-enriched-*C*-(*N*-(1,3-dioxoisoindolin-2-yl))methyl-*C'*-(sulfamoylamino)methyl-*o*-carborane (^{10}B-7).

Comparison between DEPT 135 and ^{13}C NMR (100 MHz, acetone-*d6*)

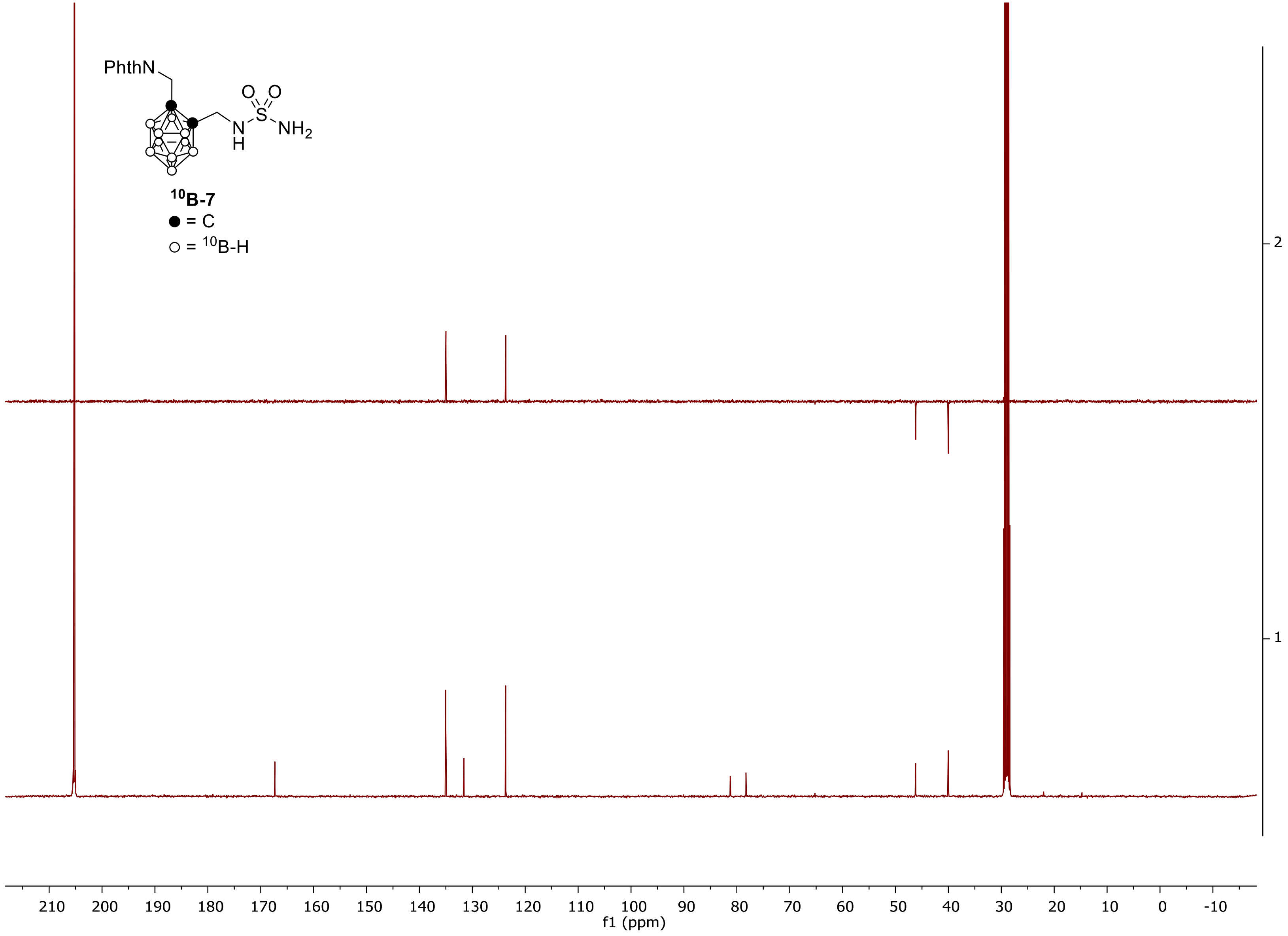

C-aminomethyl-C'-(sulfamoylamino)methyl-o-carborane hydrochloric salt (8).

^{1}H NMR (400 MHz, CD_3OD)

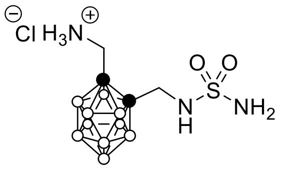

8·HCl

● = C
○ = B-H

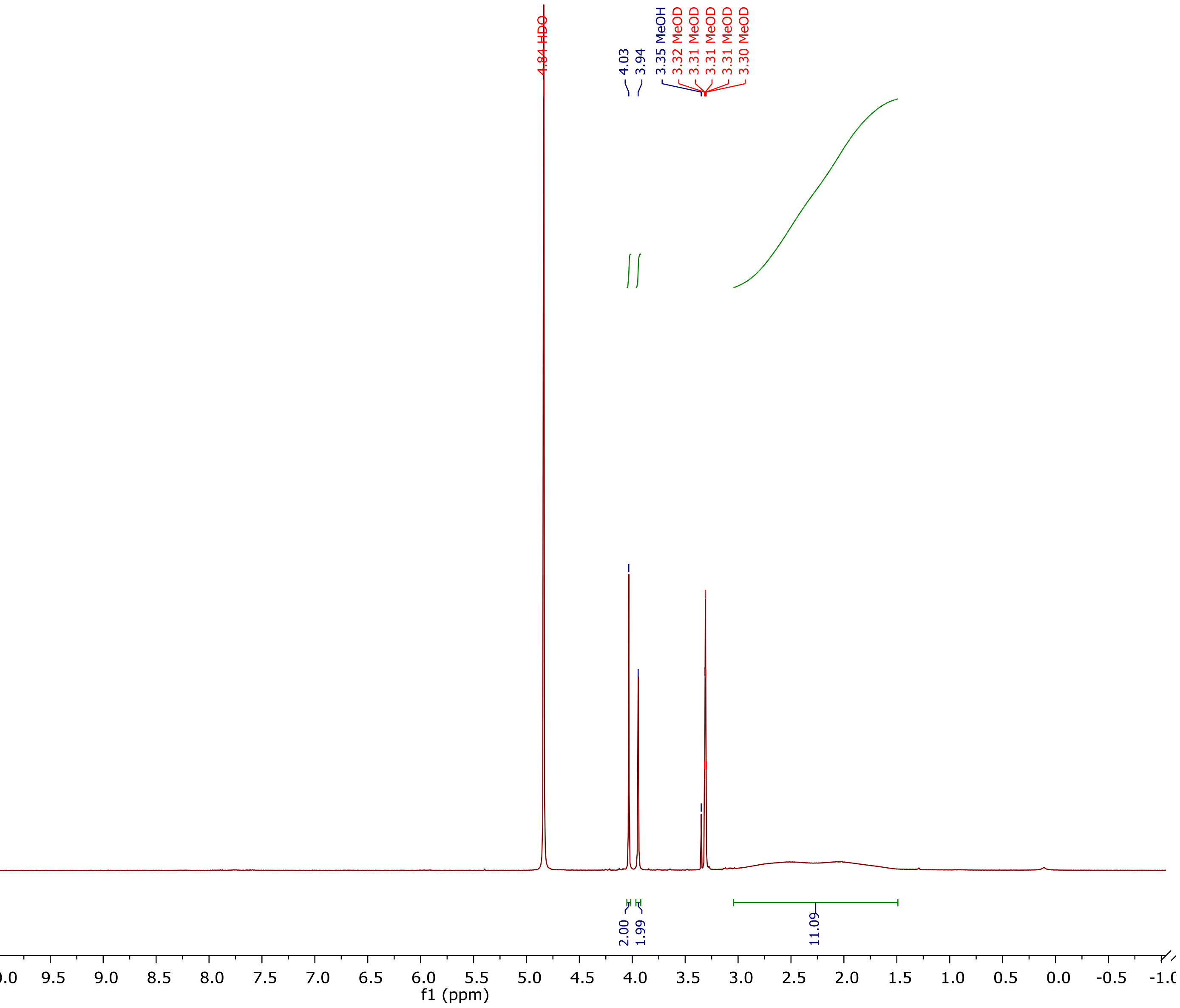

C-aminomethyl-C'-(sulfamoylamino)methyl-o-carborane hydrochloric salt (8).

^{1}H-COSY NMR (400 MHz, CD_3OD)

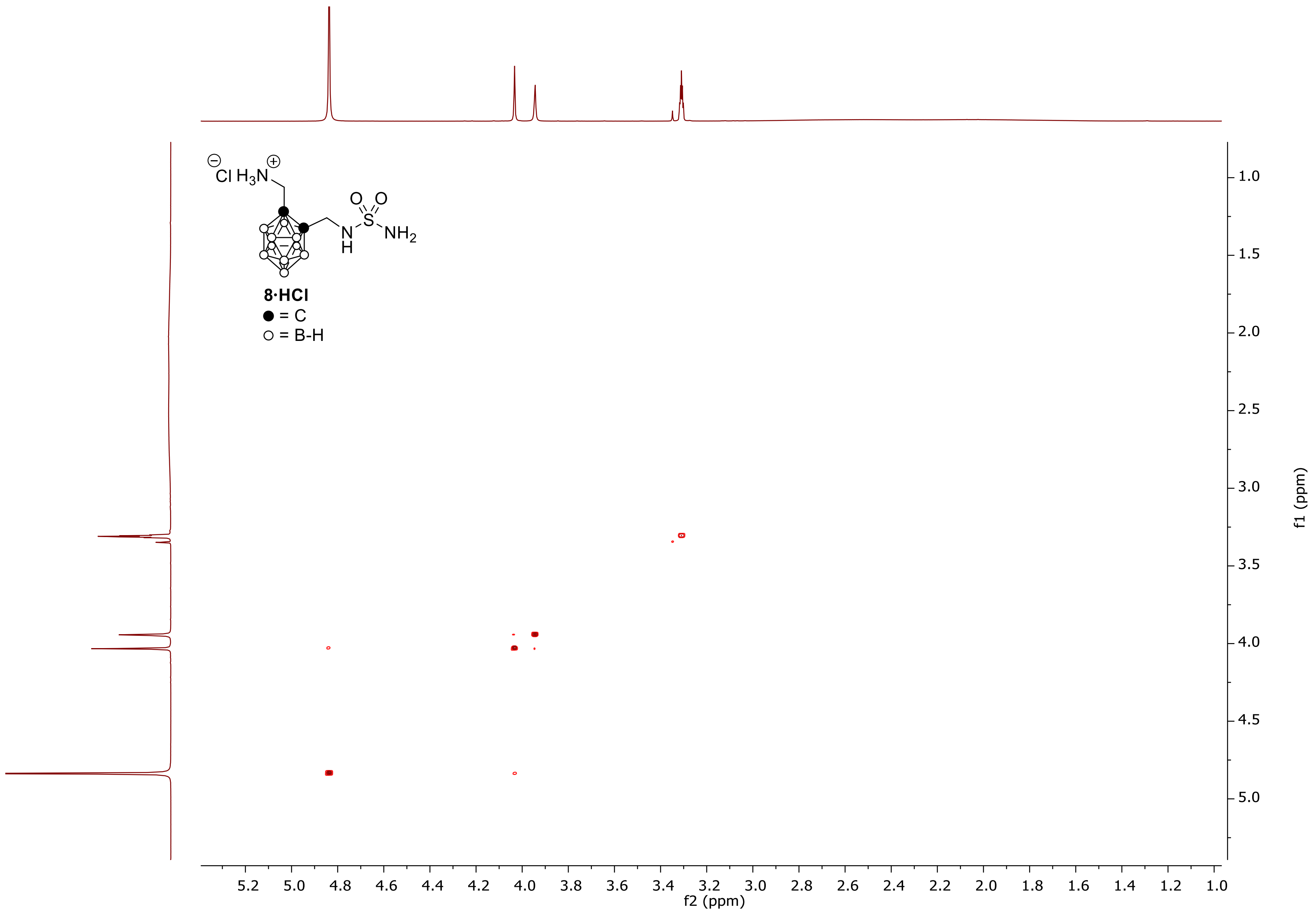

***C*-aminomethyl-*C'*-(sulfamoylamino)methyl-*o*-carborane hydrochloric salt (8).**

^{13}C NMR (100 MHz, CD_3OD)

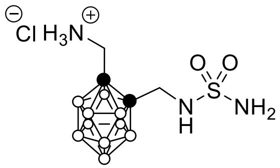

8·HCl

● = C

○ = B-H

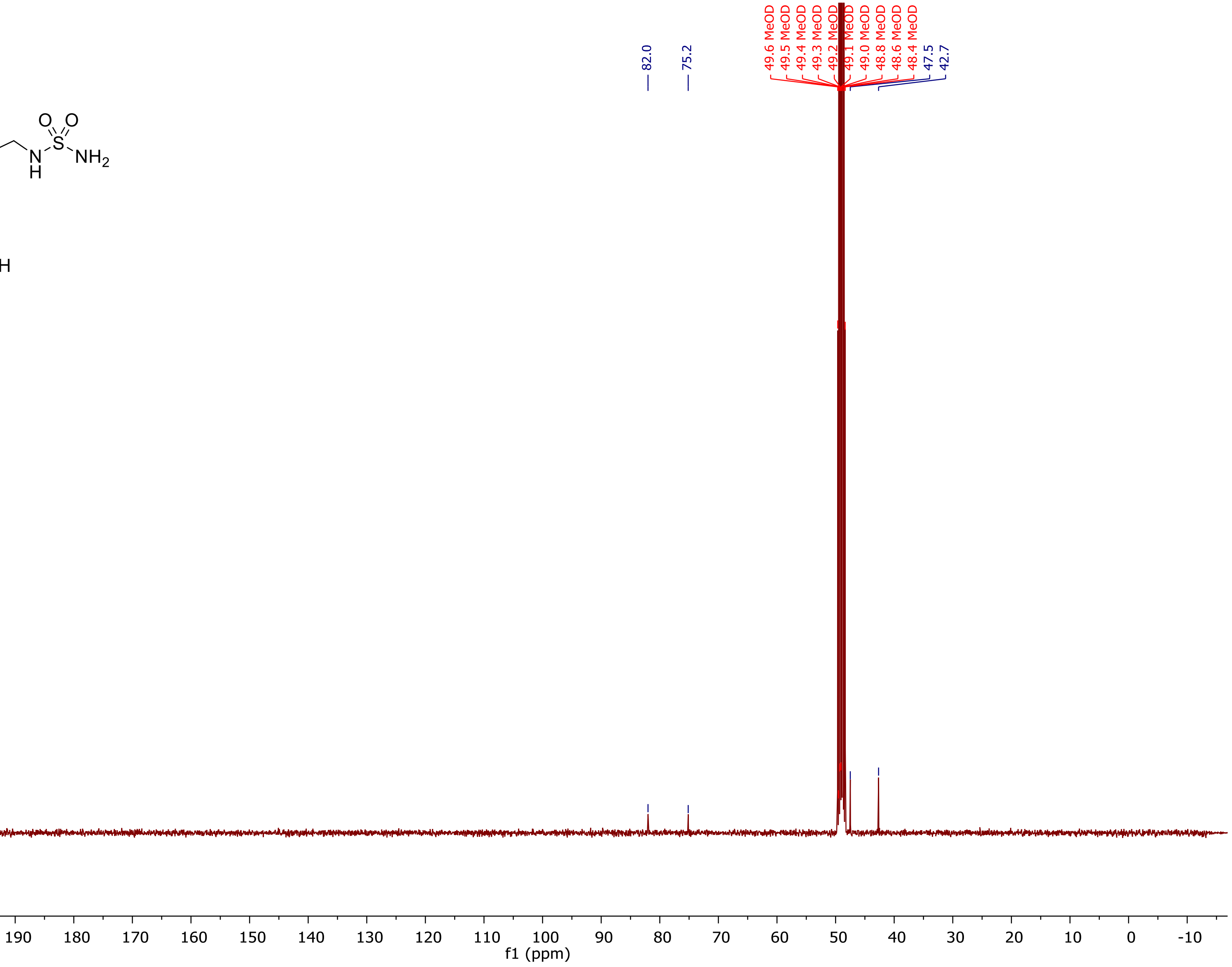

C-aminomethyl-C'-(sulfamoylamino)methyl-o-carborane hydrochloric salt (8).

Comparison between DEPT 135 and ^{13}C NMR (100 MHz, CD_3OD)

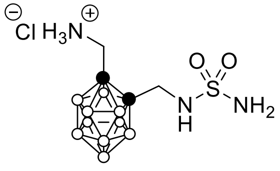

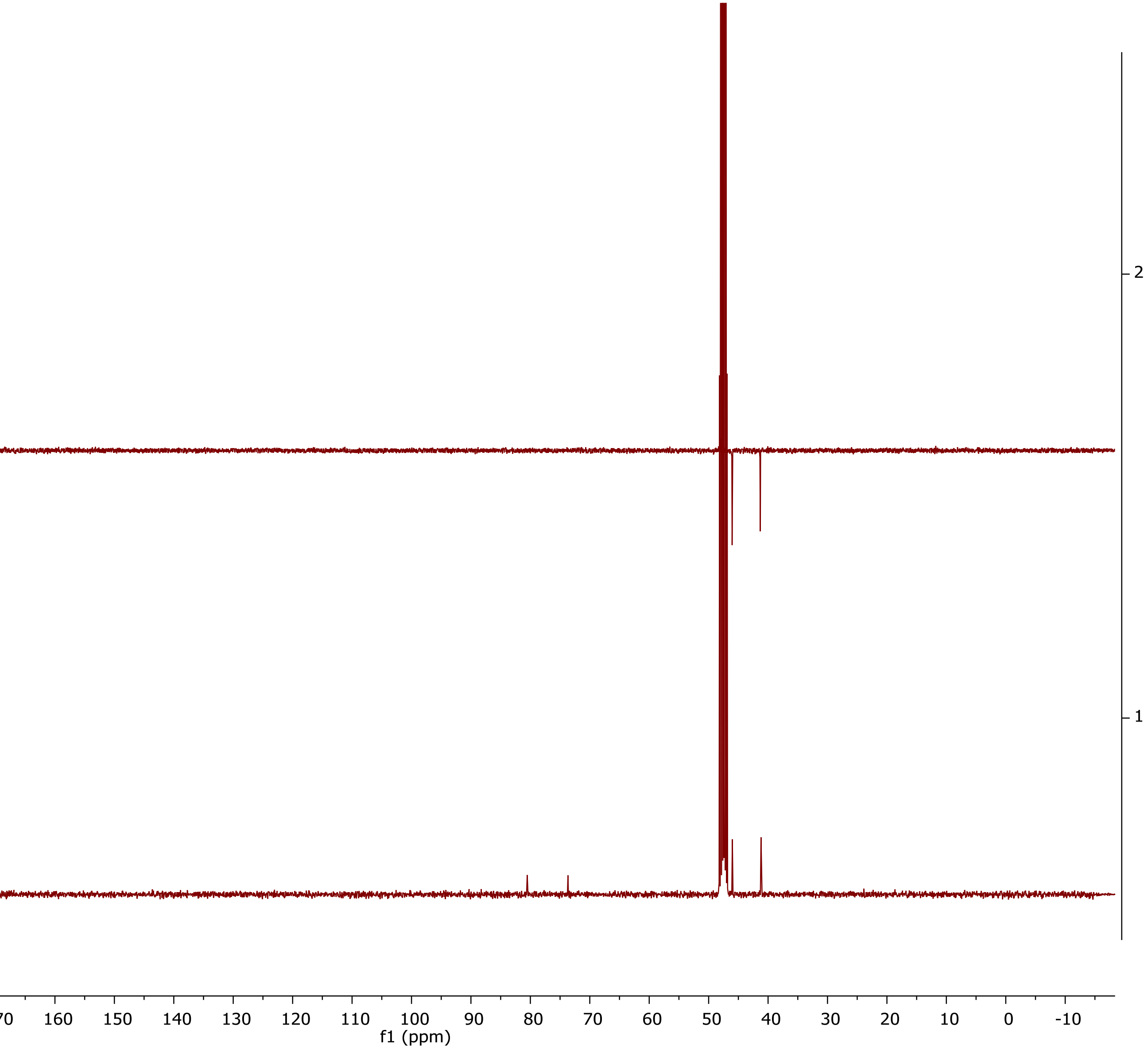

***C*-aminomethyl-*C'*-(sulfamoylamino)methyl-*o*-carborane hydrochloric salt (8).**

^{11}B NMR (128 MHz, CD_3OD)

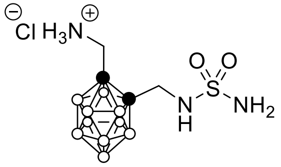

8·HCl
● = C
○ = B-H

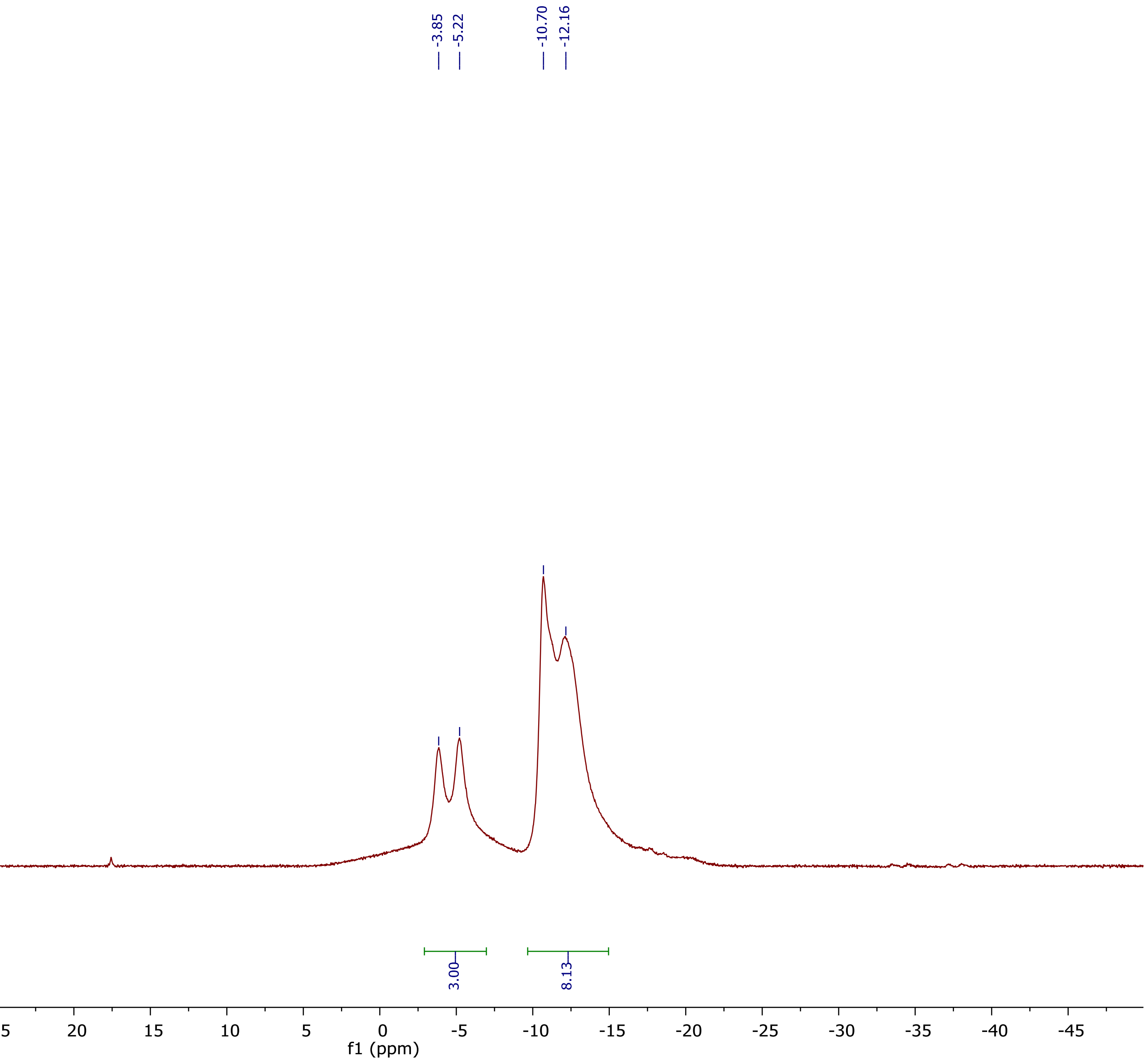

[10]B-enriched-*C*-aminomethyl-*C'*-(sulfamoylamino)methyl-*o*-carborane hydrochloric salt ([10]B-8).

^{1}H NMR (400 MHz, CD_3OD)

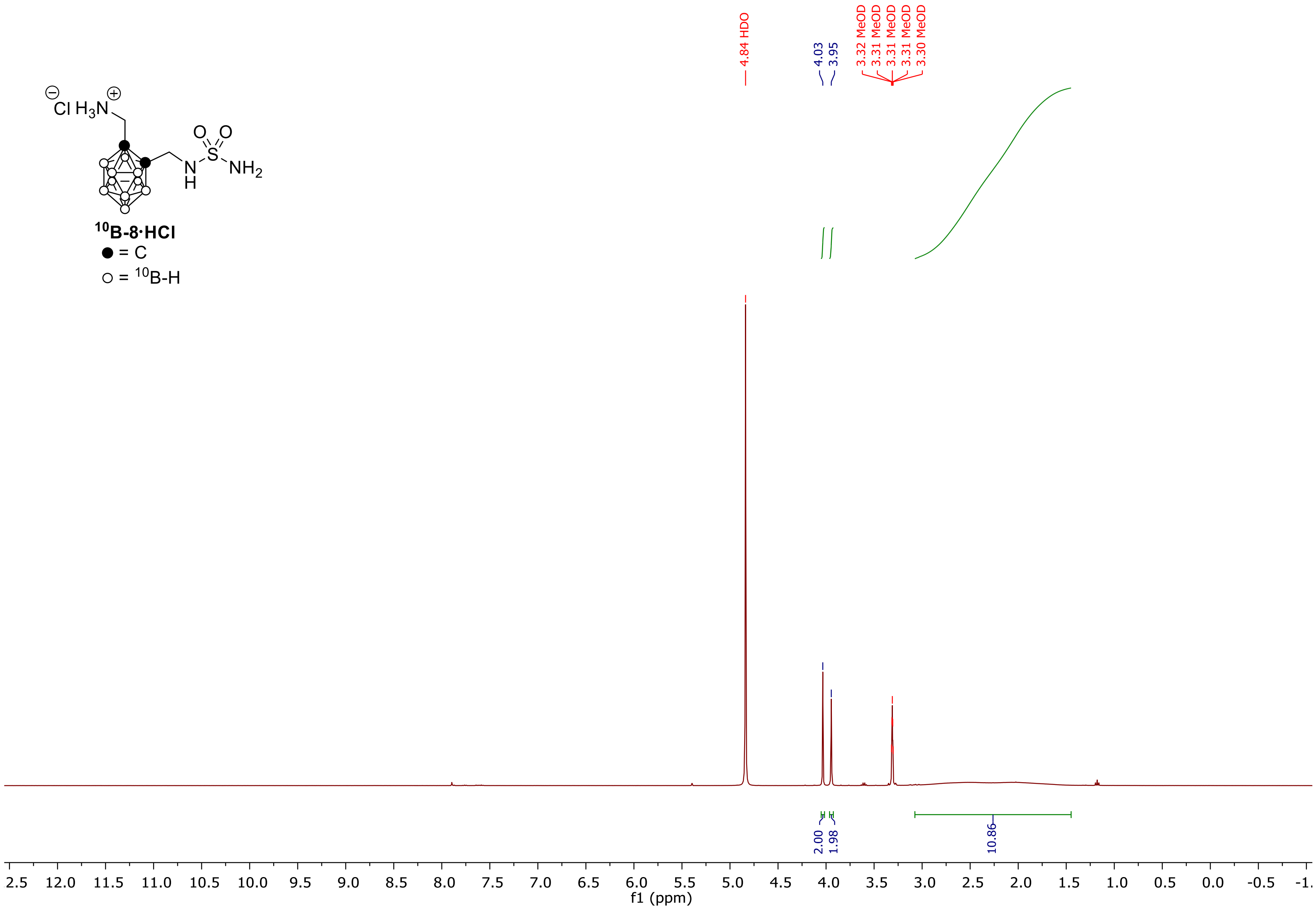

[10]B-enriched-*C*-aminomethyl-*C'*-(sulfamoylamino)methyl-*o*-carborane hydrochloric salt ([10]B-8).

^{1}H-COSY NMR (400 MHz, CD_3OD)

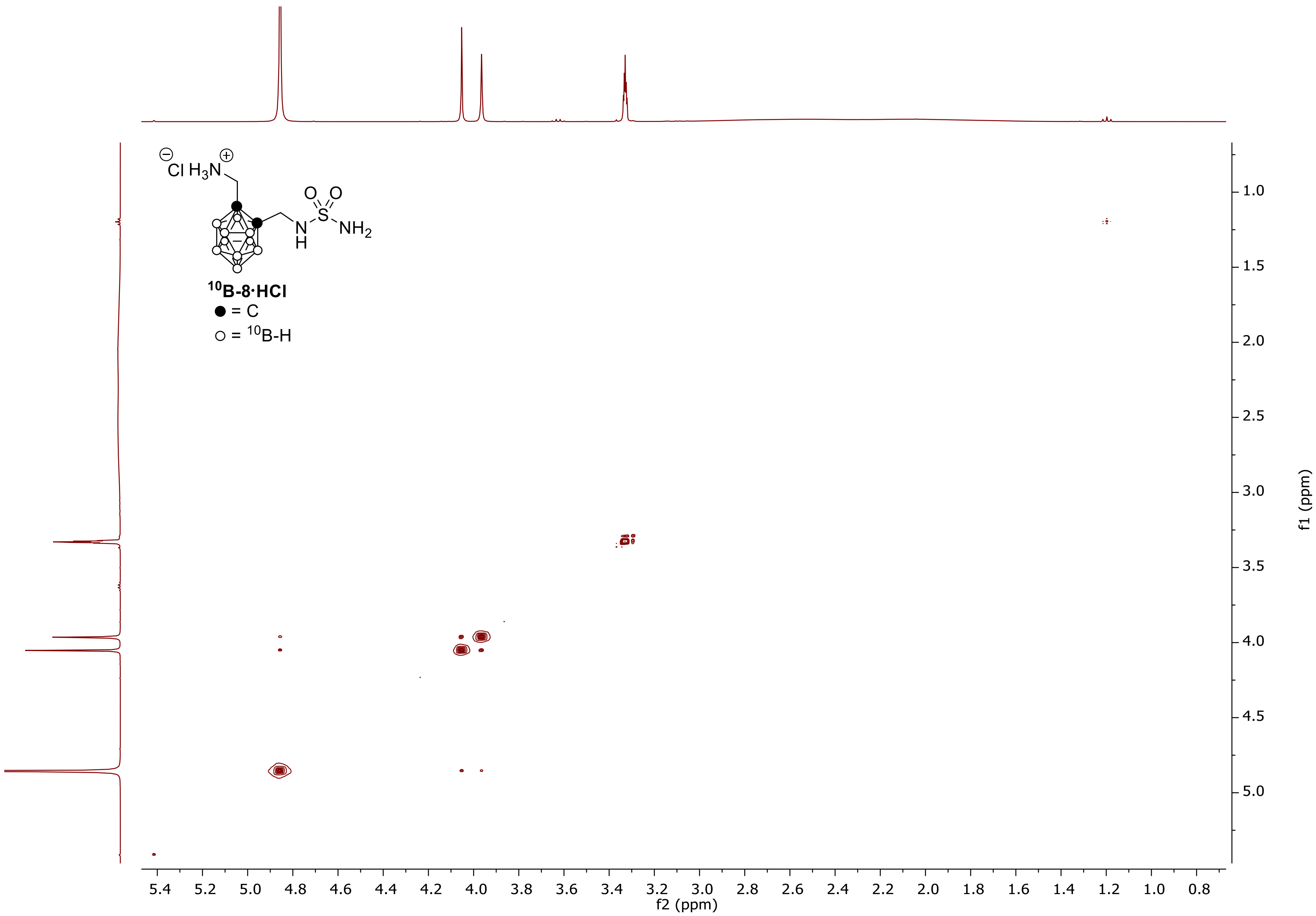

^{10}B-enriched-*C*-aminomethyl-*C'*-(sulfamoylamino)methyl-*o*-carborane hydrochloric salt (^{10}B-8).

^{13}C NMR (100 MHz, CD_3OD)

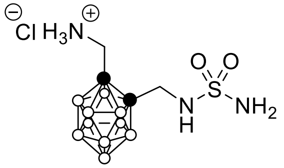

^{10}B-8·HCl

● = C

○ = ^{10}B-H

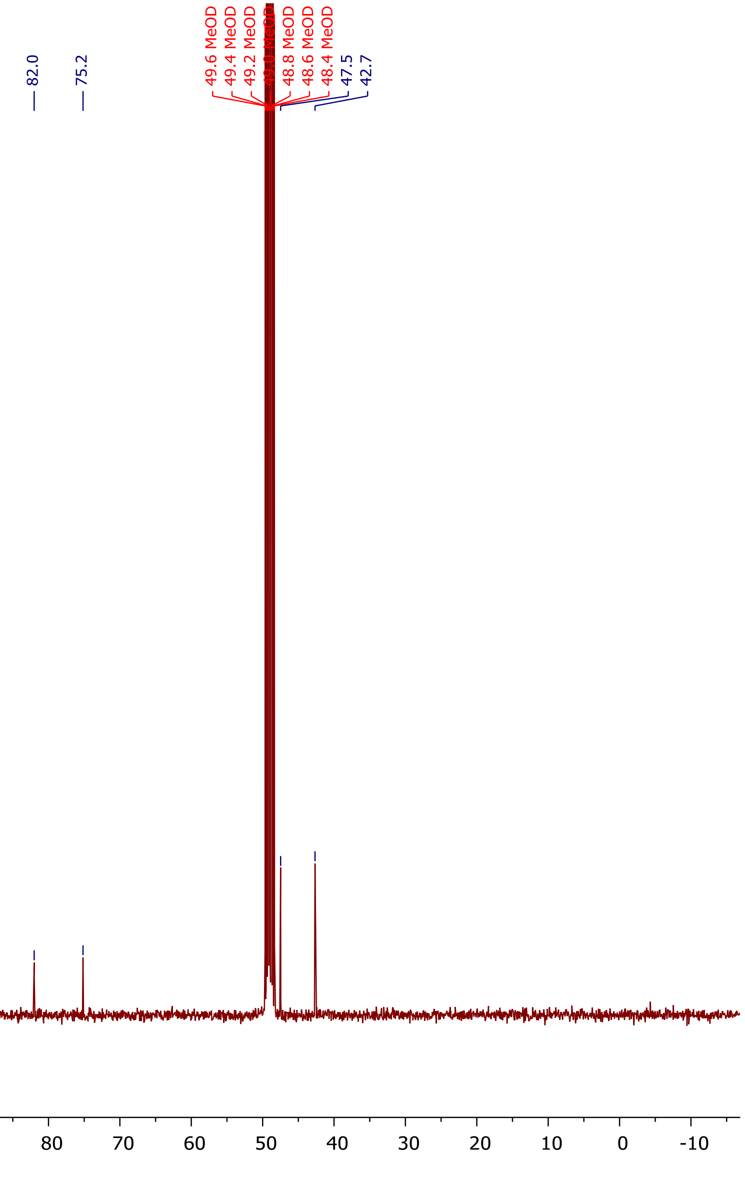

^{10}B-enriched-*C*-aminomethyl-*C'*-(sulfamoylamino)methyl-*o*-carborane hydrochloric salt (^{10}B-8).

Comparison between DEPT 135 and ^{13}C NMR (100 MHz, CD_3OD)

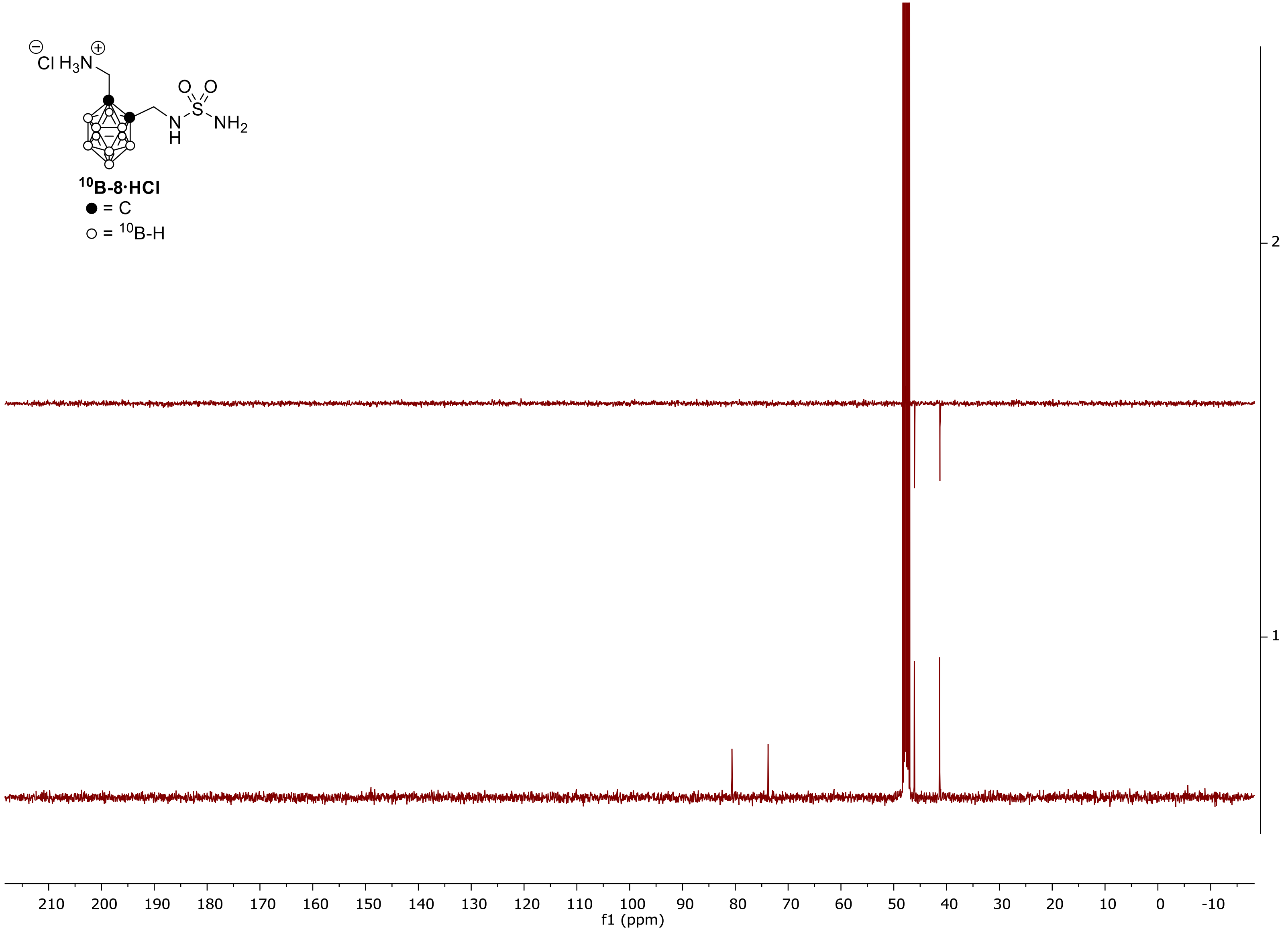

C-(6-(2-(4,7,10-tris(2-(*tert*-butoxy)-2-oxoethyl)-1,4,7,10-tetraazacyclododecan-1-yl)acetamido)hexanamido)methyl-*C'*-(sulfamoylamino)methyl-*o*-carborane (9).

^{1}H NMR (600 MHz, $CDCl_3$)

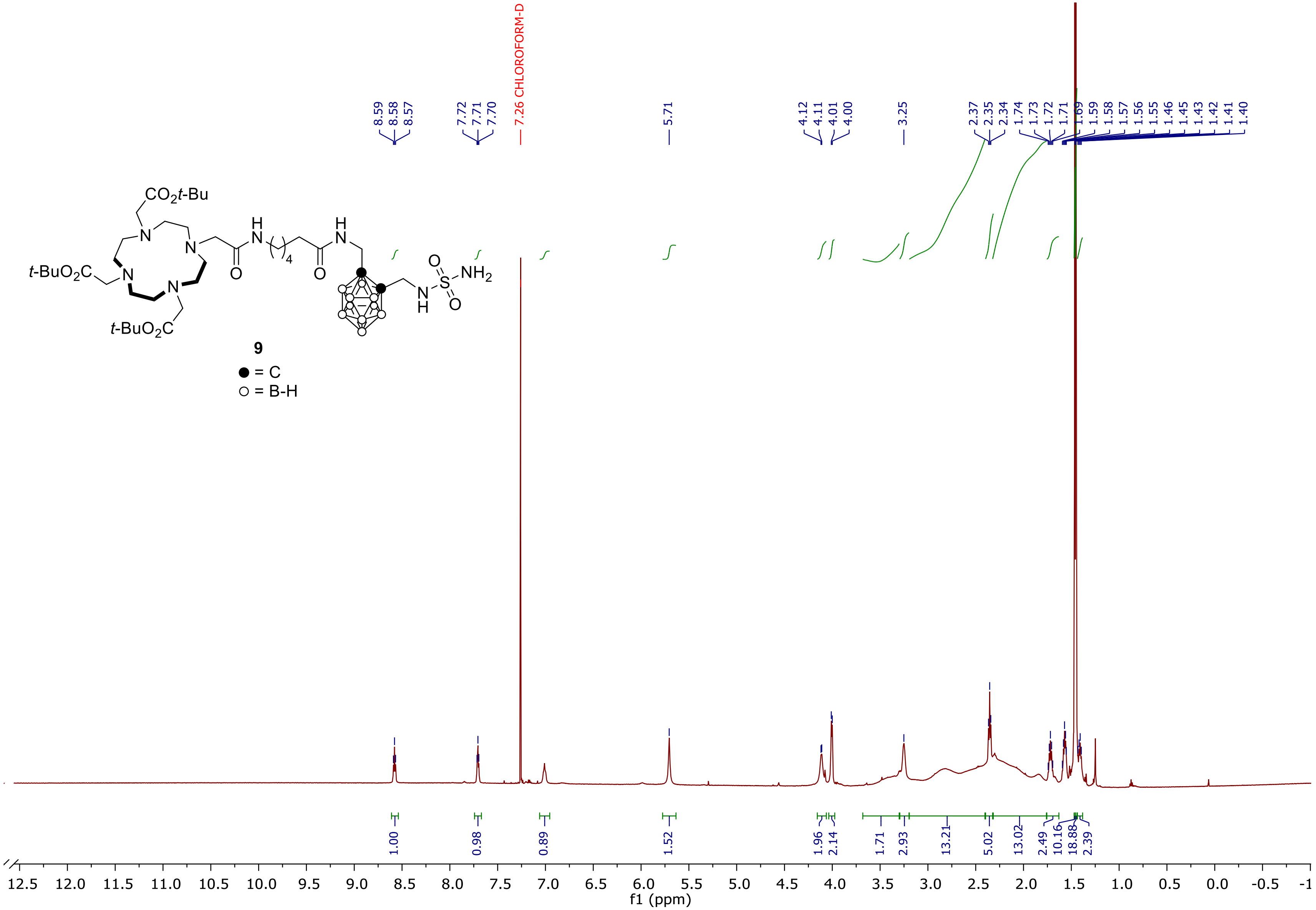

_C_-(6-(2-(4,7,10-tris(2-(_tert_-butoxy)-2-oxoethyl)-1,4,7,10-tetraazacyclododecan-1-yl)acetamido)hexanamido)methyl-_C'_-(sulfamoylamino)methyl-_o_-carborane (9).

^{1}H-COSY NMR (600 MHz, $CDCl_3$)

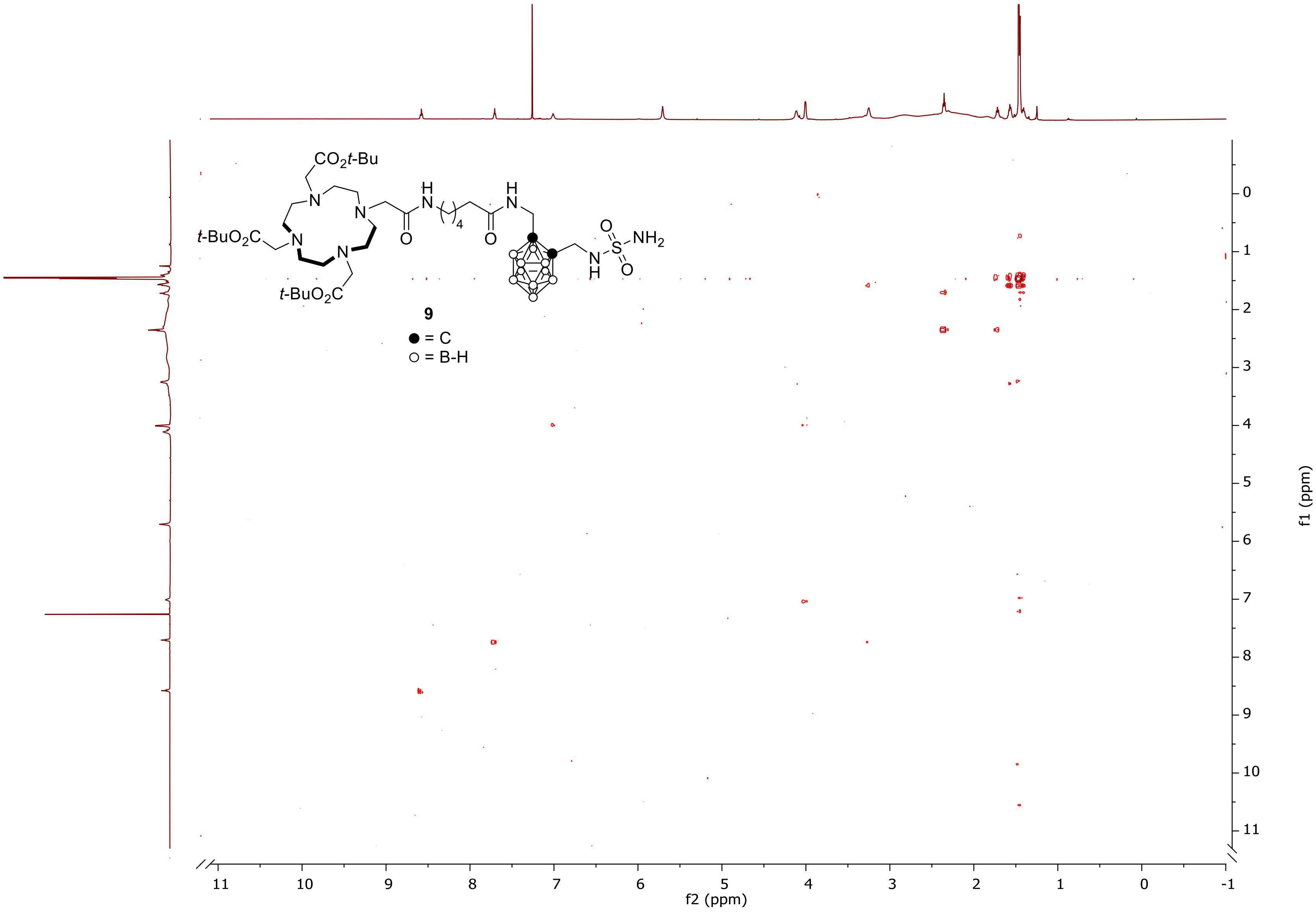

***C*-(6-(2-(4,7,10-tris(2-(*tert*-butoxy)-2-oxoethyl)-1,4,7,10-tetraazacyclododecan-1-yl)acetamido)hexanamido)methyl-*C'*-(sulfamoylamino)methyl-*o*-carborane (9).**

^{13}C NMR (150 MHz, $CDCl_3$)

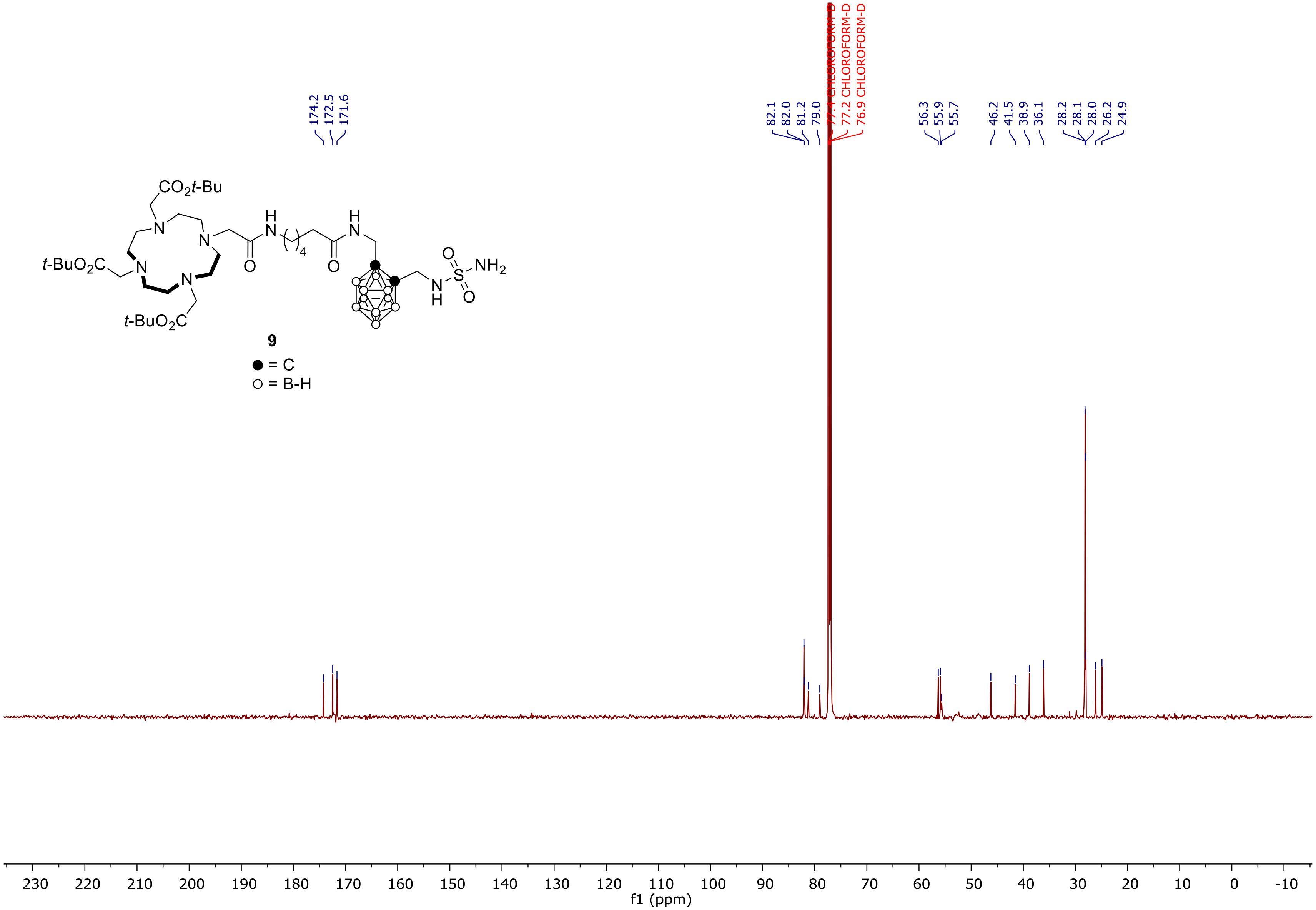

C-(6-(2-(4,7,10-tris(2-(*tert*-butoxy)-2-oxoethyl)-1,4,7,10-tetraazacyclododecan-1-yl)acetamido)hexanamido)methyl-*C'*-(sulfamoylamino)methyl-*o*-carborane (9).

Comparison between DEPT 135 and ^{13}C NMR (150 MHz, $CDCl_3$)

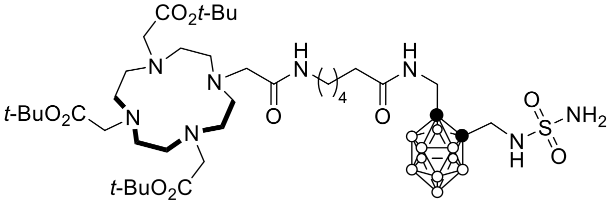

9

● = C

○ = B-H

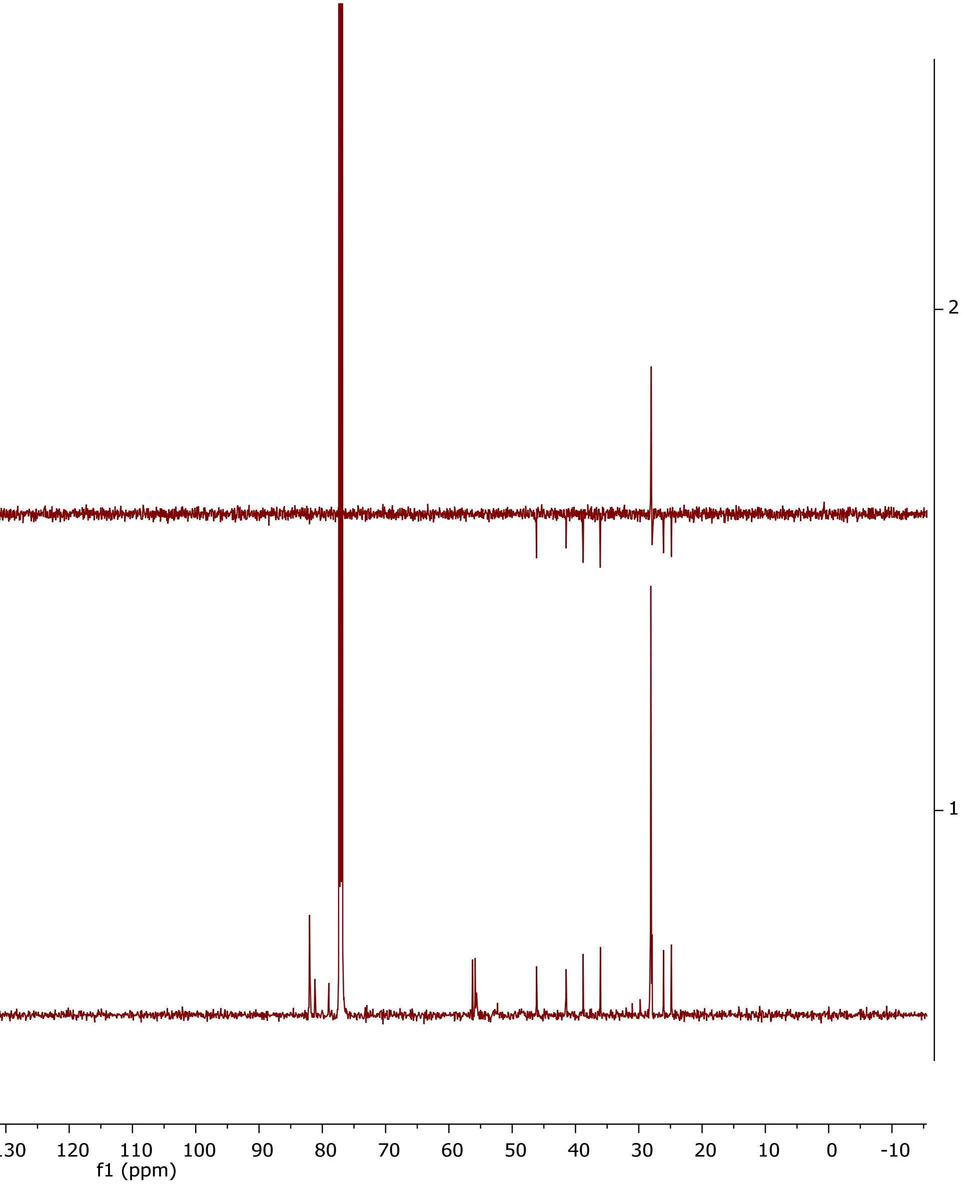

_C_-(6-(2-(4,7,10-tris(2-(_tert_-butoxy)-2-oxoethyl)-1,4,7,10-tetraazacyclododecan-1-yl)acetamido)hexanamido)methyl-_C'_-(sulfamoylamino)methyl-_o_-carborane (9).

^{11}B NMR (192.5 MHz, $CDCl_3$)

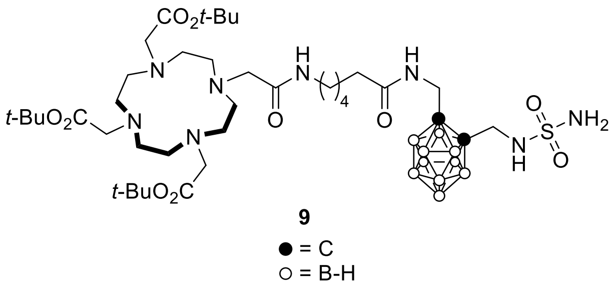

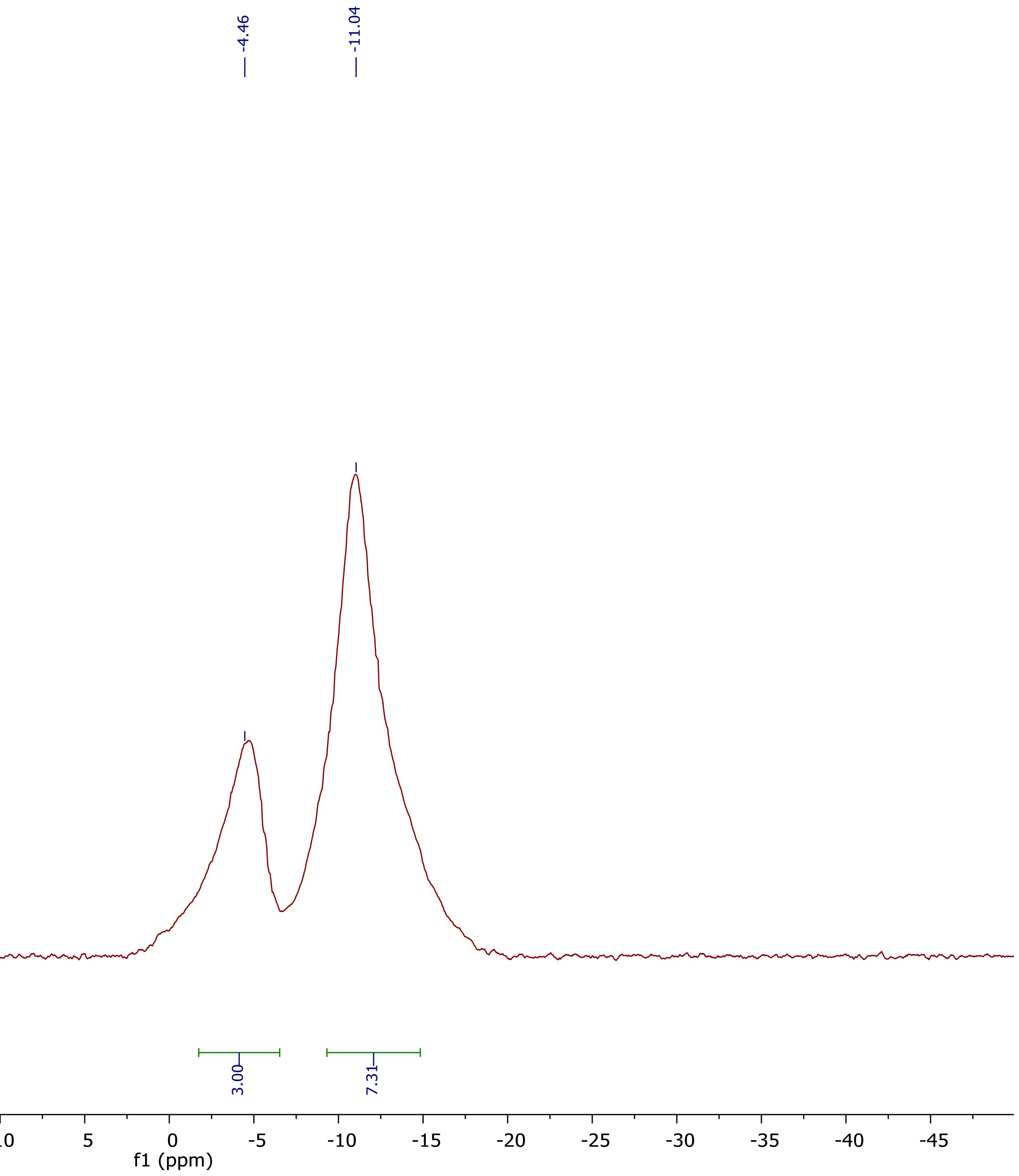

^{10}B-enriched-*C*-(6-(2-(4,7,10-tris(2-(*tert*-butoxy)-2-oxoethyl)-1,4,7,10-tetraazacyclododecan-1-yl)acetamido)hexanamido)methyl-*C'*-(sulfamoylamino)methyl-*o*-carborane (^{10}B-9).

^{1}H NMR (600 MHz, $CDCl_3$)

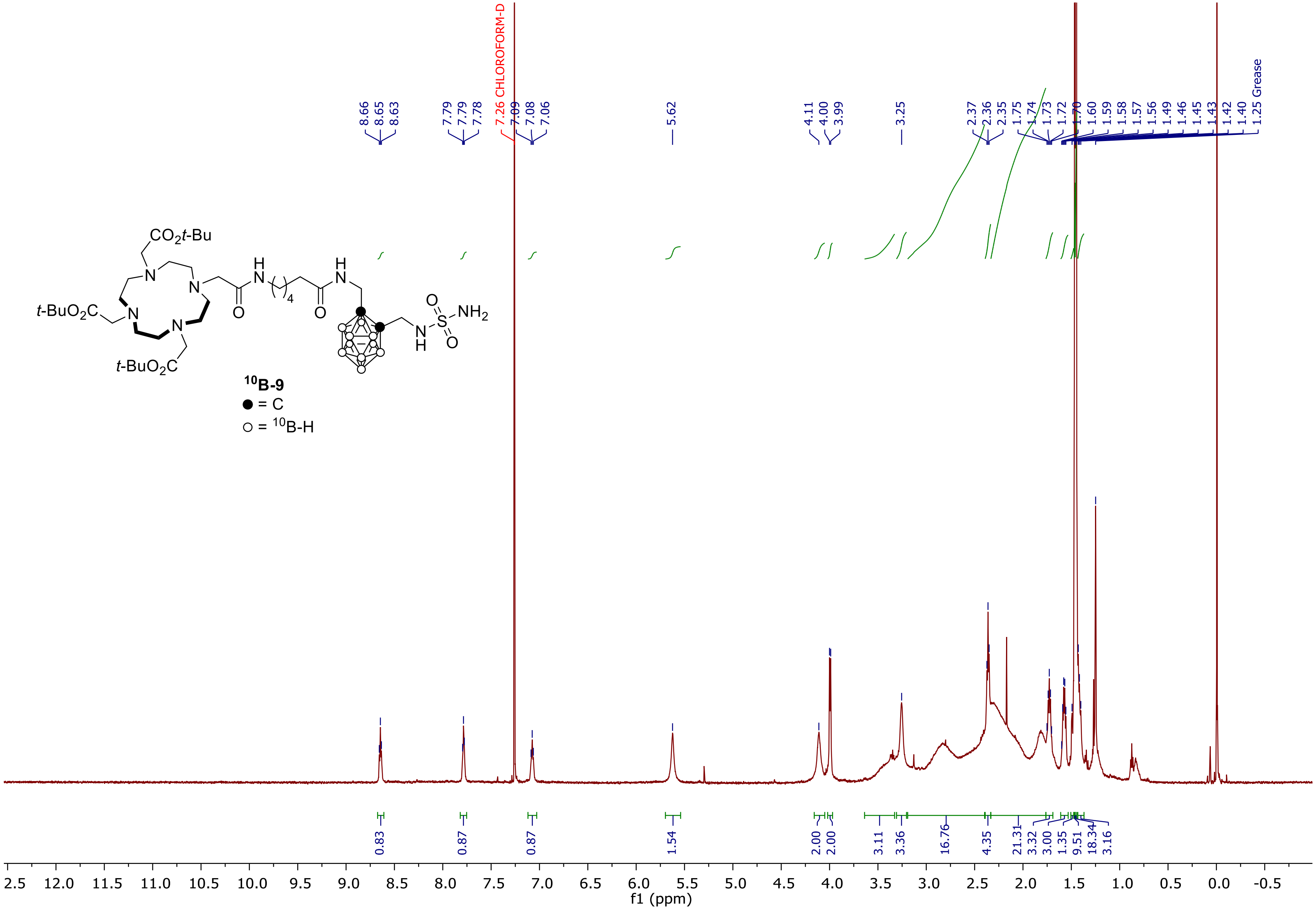

^{10}B-enriched-*C*-(6-(2-(4,7,10-tris(2-(*tert*-butoxy)-2-oxoethyl)-1,4,7,10-tetraazacyclododecan-1-yl)acetamido)hexanamido)methyl-*C'*-(sulfamoylamino)methyl-*o*-carborane (^{10}B-9).

^{1}H-COSY NMR (600 MHz, $CDCl_3$)

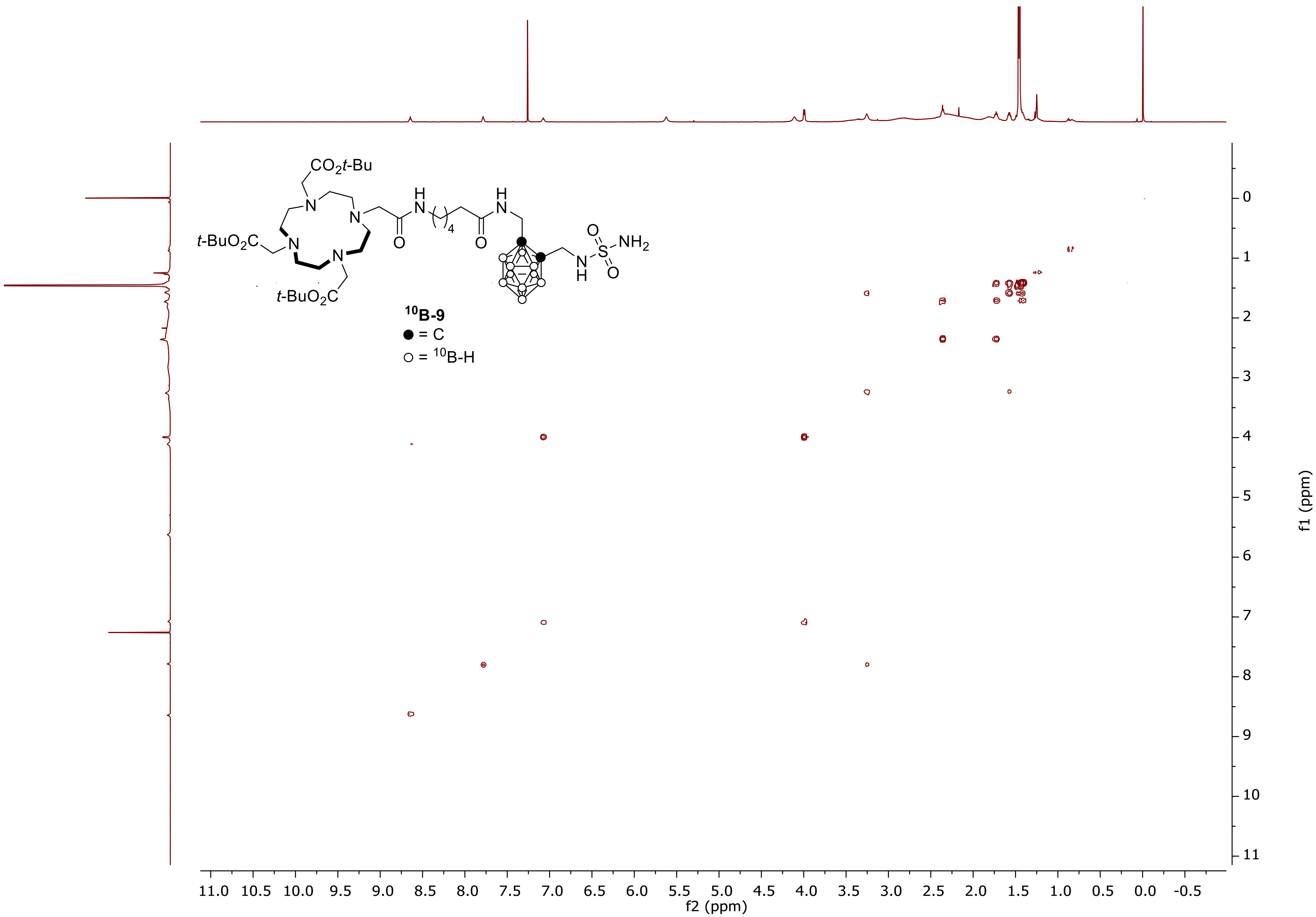

^{10}B-enriched-*C*-(6-(2-(4,7,10-tris(2-(*tert*-butoxy)-2-oxoethyl)-1,4,7,10-tetraazacyclododecan-1-yl)acetamido)hexanamido)methyl-*C'*-(sulfamoylamino)methyl-*o*-carborane (^{10}B-9).

^{13}C NMR (150 MHz, $CDCl_3$)

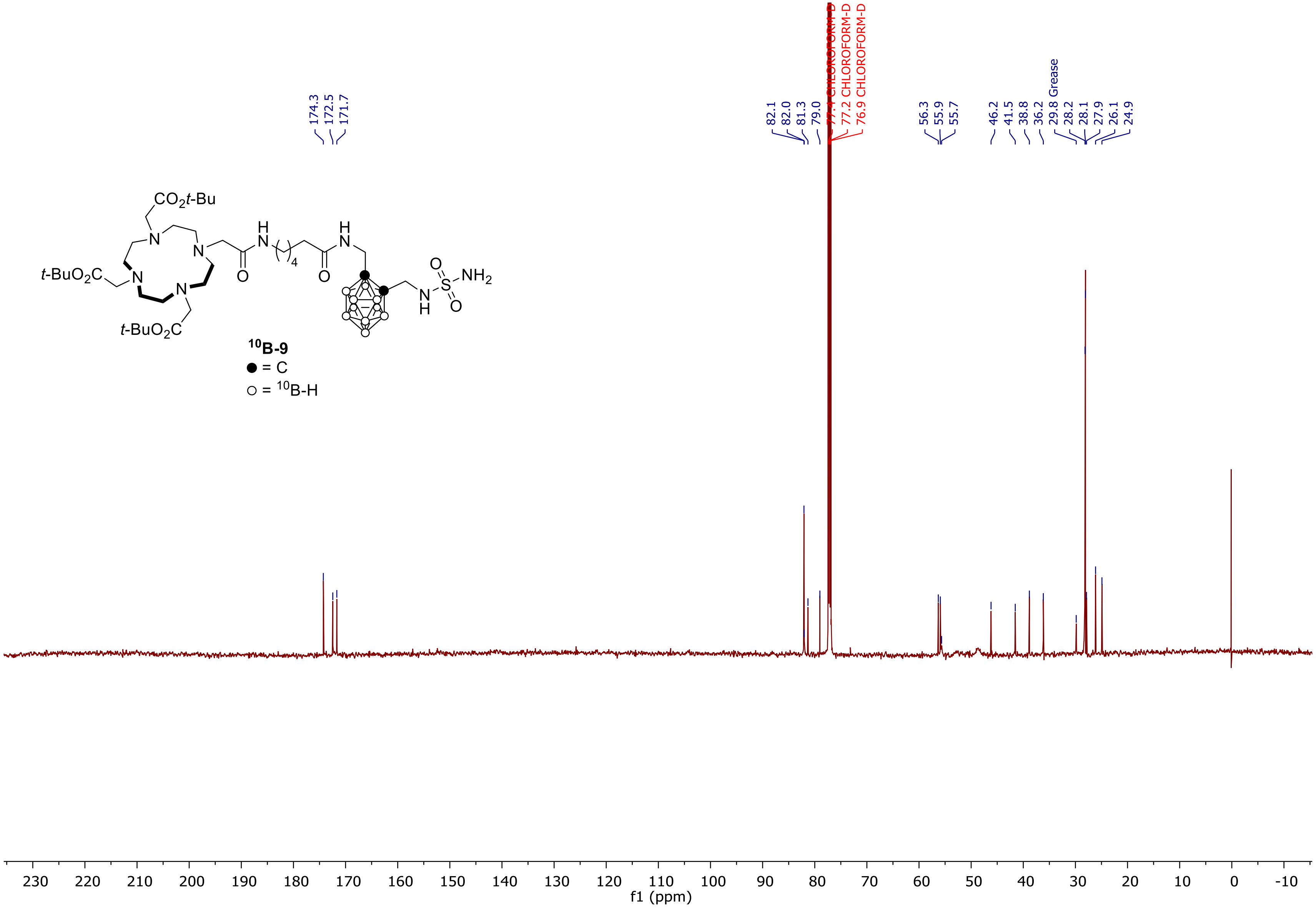

^{10}B-enriched-*C*-(6-(2-(4,7,10-tris(2-(*tert*-butoxy)-2-oxoethyl)-1,4,7,10-tetraazacyclododecan-1-yl)acetamido)hexanamido)methyl-*C'*-(sulfamoylamino)methyl-*o*-carborane (^{10}B-9).

Comparison between DEPT 135 and ^{13}C NMR (150 MHz, $CDCl_3$)

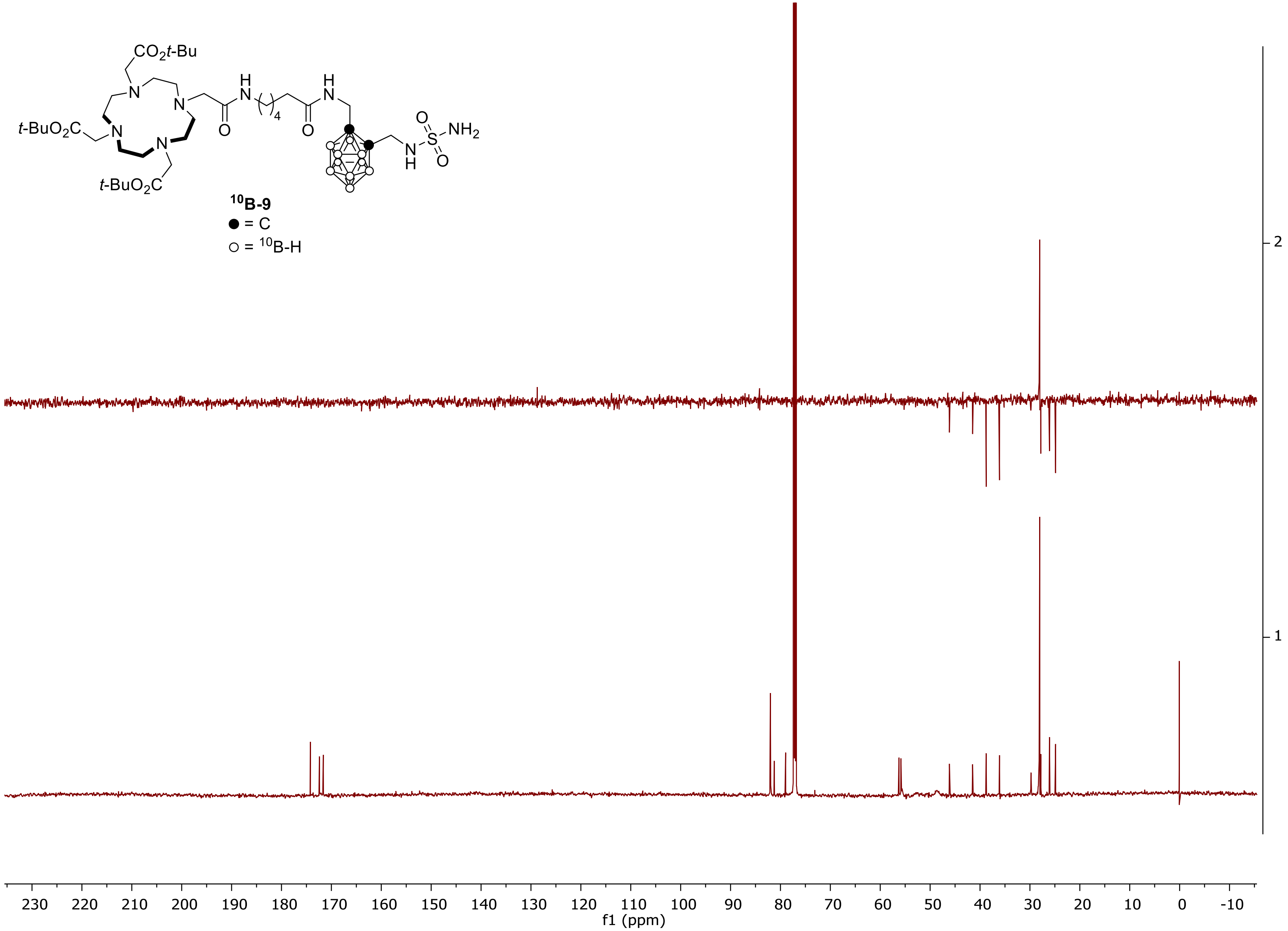

C-(6-(2-(4,7,10-tris-carboxymethyl-1,4,7,10-tetraazacyclododecan-1-yl)acetamido)hexanamido)methyl-C′-(sulfamoylamino)methyl-o-carborane (10).

^{1}H NMR (400 MHz, CD_3OD)

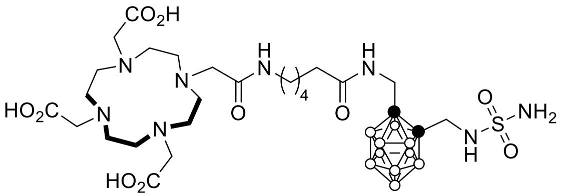

10

● = C
○ = B-H

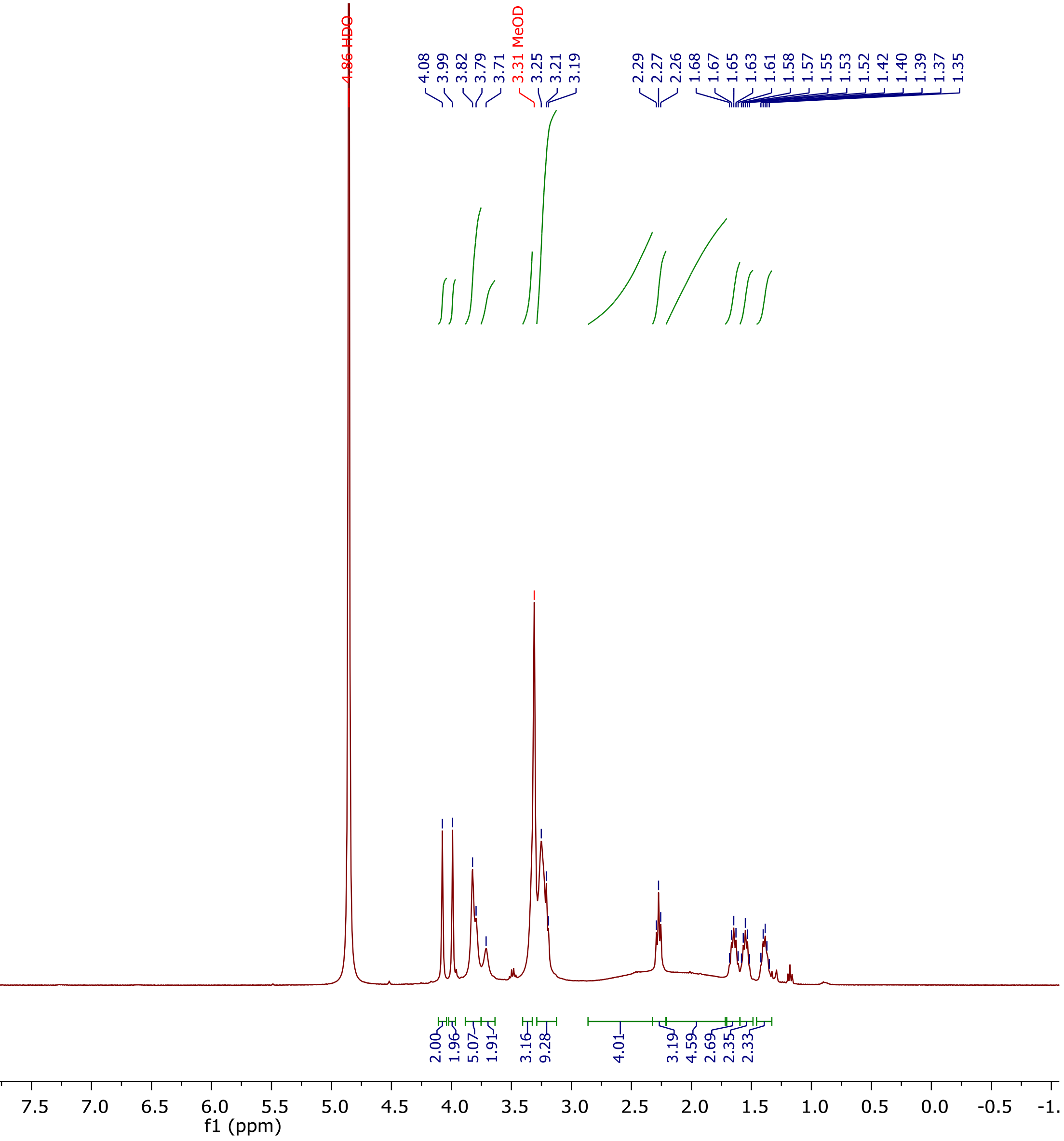

C-(6-(2-(4,7,10-tris-carboxymethyl-1,4,7,10-tetraazacyclododecan-1-yl)acetamido)hexanamido)methyl-C'-(sulfamoylamino)methyl-o-carborane (10).

^{1}H-COSY NMR (400 MHz, CD_3OD)

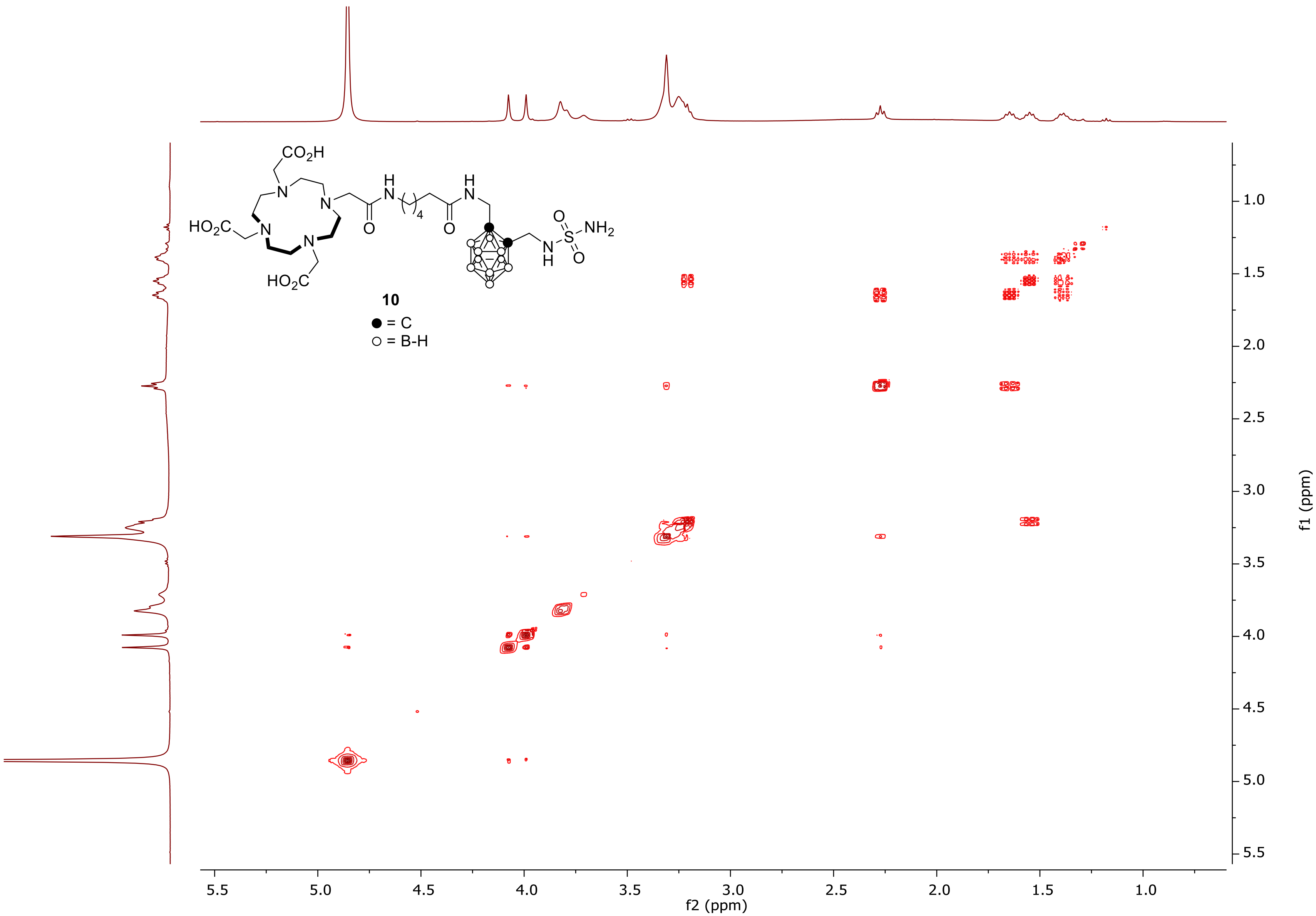

C-(6-(2-(4,7,10-tris-carboxymethyl-1,4,7,10-tetraazacyclododecan-1-yl)acetamido)hexanamido)methyl-C′-(sulfamoylamino)methyl-o-carborane (10).

^{13}C NMR (100 MHz, CD_3OD)

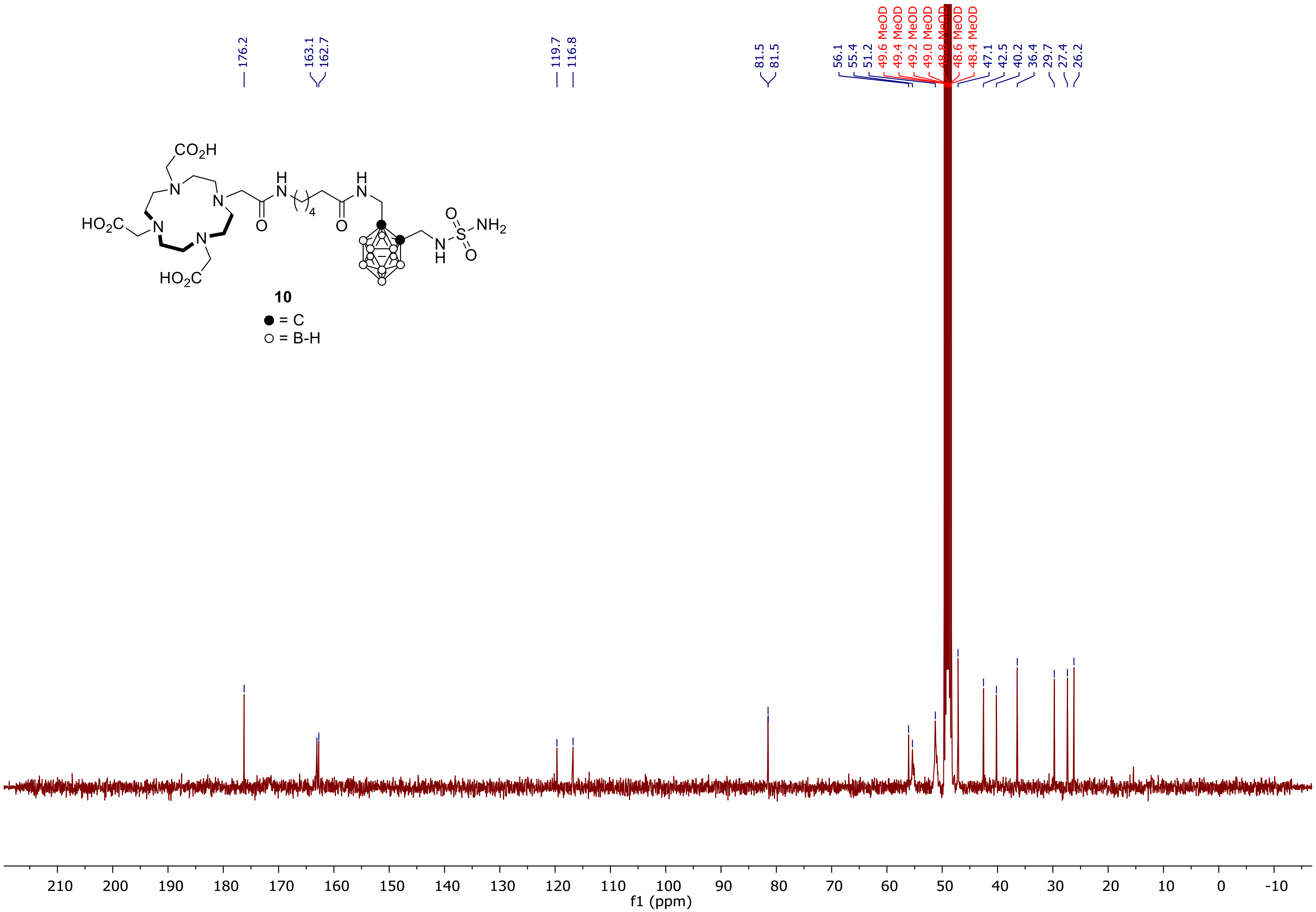

***C*-(6-(2-(4,7,10-tris-carboxymethyl-1,4,7,10-tetraazacyclododecan-1-yl)acetamido)hexanamido)methyl-*C'*-(sulfamoylamino)methyl-*o*-carborane (10).**

Comparison between DEPT 135 and ^{13}C NMR (100 MHz, CD_3OD)

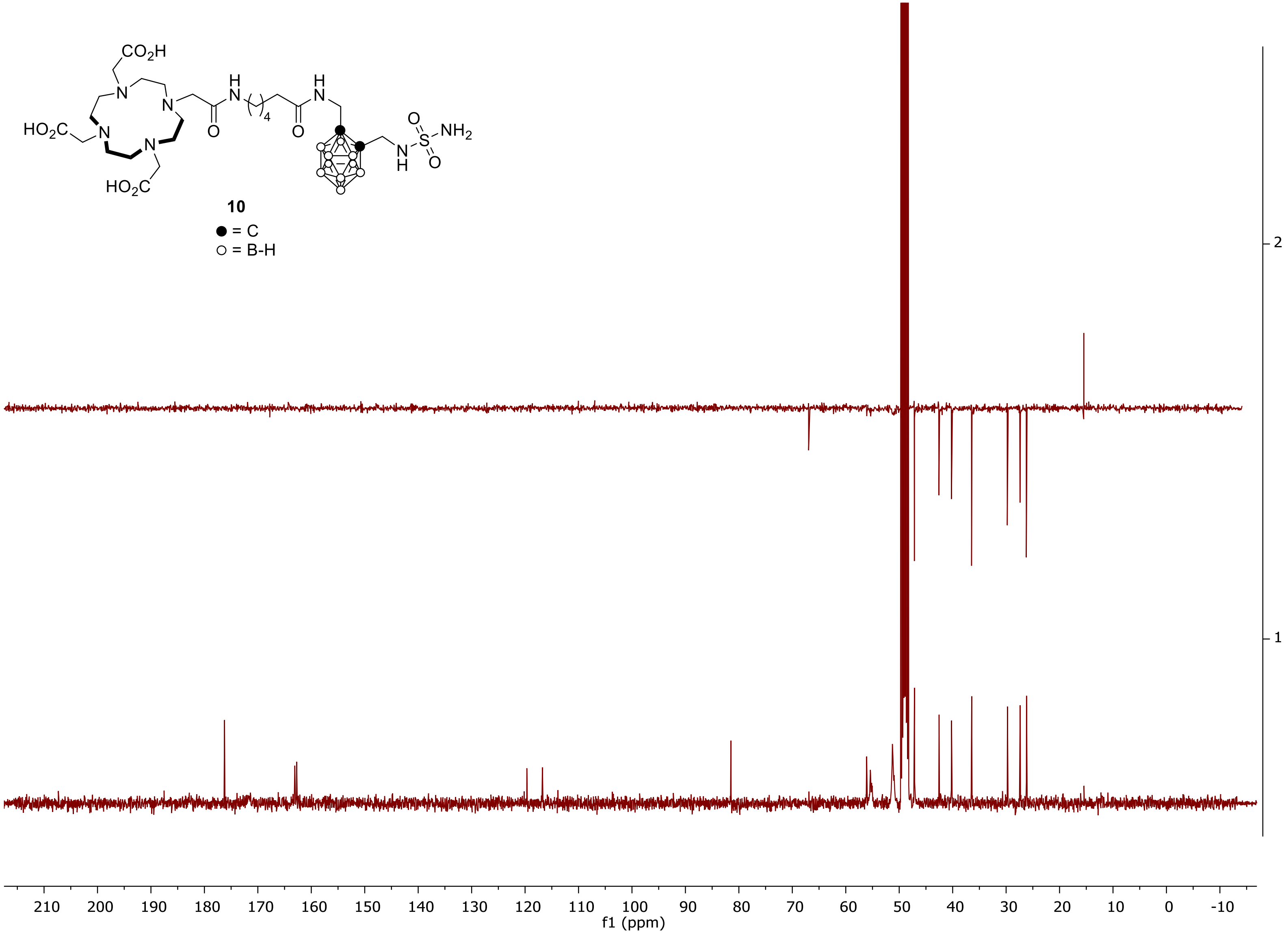

C-(6-(2-(4,7,10-tris-carboxymethyl-1,4,7,10-tetraazacyclododecan-1-yl)acetamido)hexanamido)methyl-C′-(sulfamoylamino)methyl-o-carborane (10).

^{11}B NMR (192.5 MHz, CD_3OD)

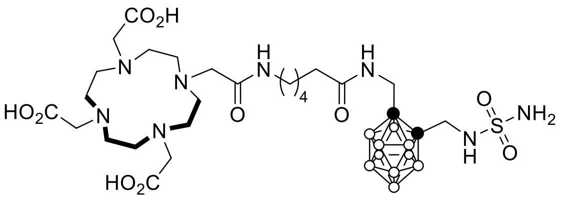

10

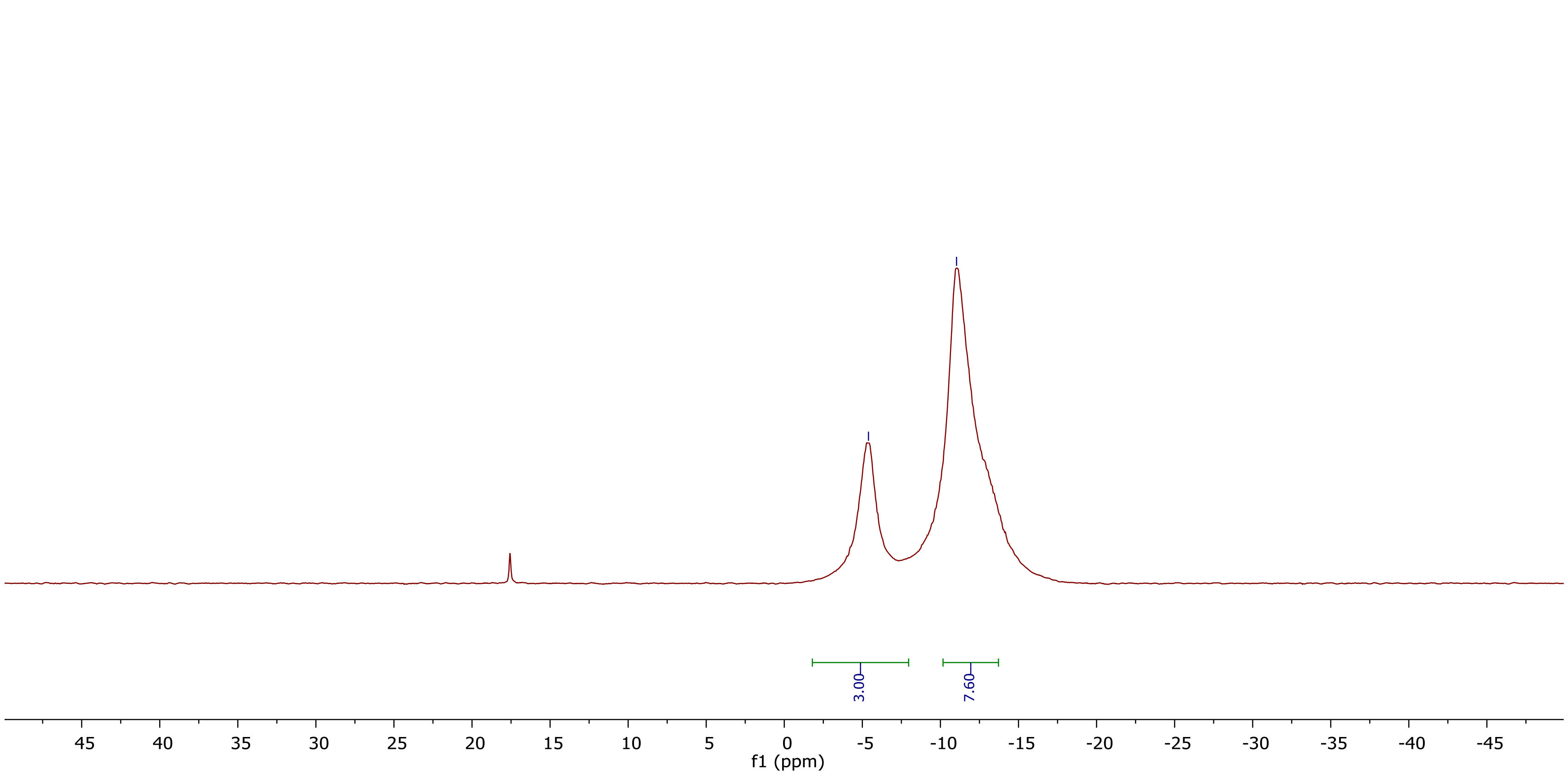

[10]B-enriched-*C*-(6-(2-(4,7,10-tris-carboxymethyl-1,4,7,10-tetraazacyclododecan-1-yl)acetamido)hexanamido)methyl-*C'*-(sulfamoylamino)methyl-*o*-carborane ([10]B-10).

^{1}H NMR (400 MHz, CD_3OD)

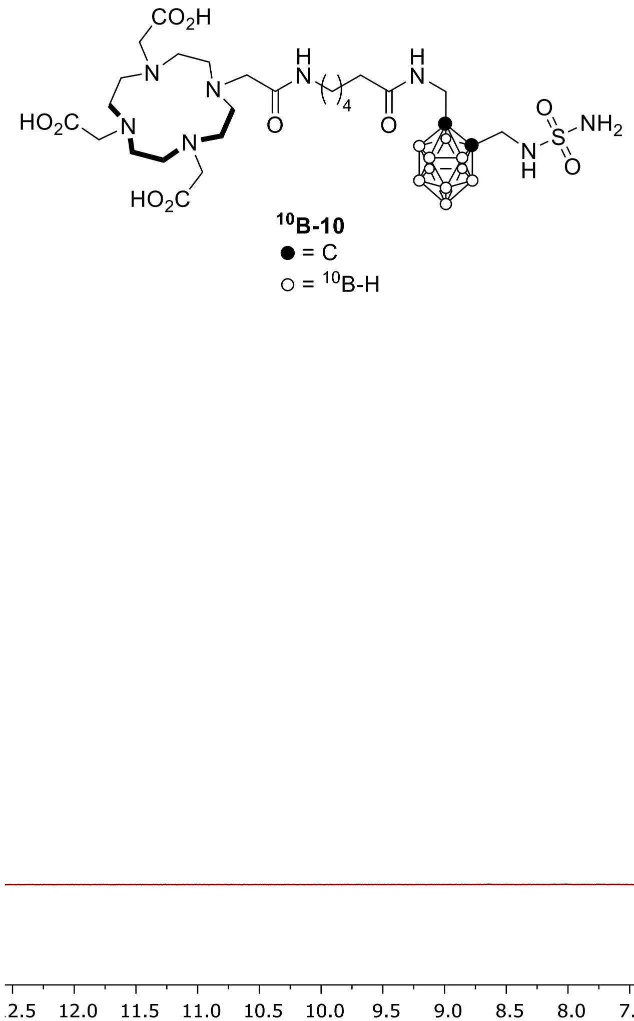

[10]B-10

● = C

○ = [10]B-H

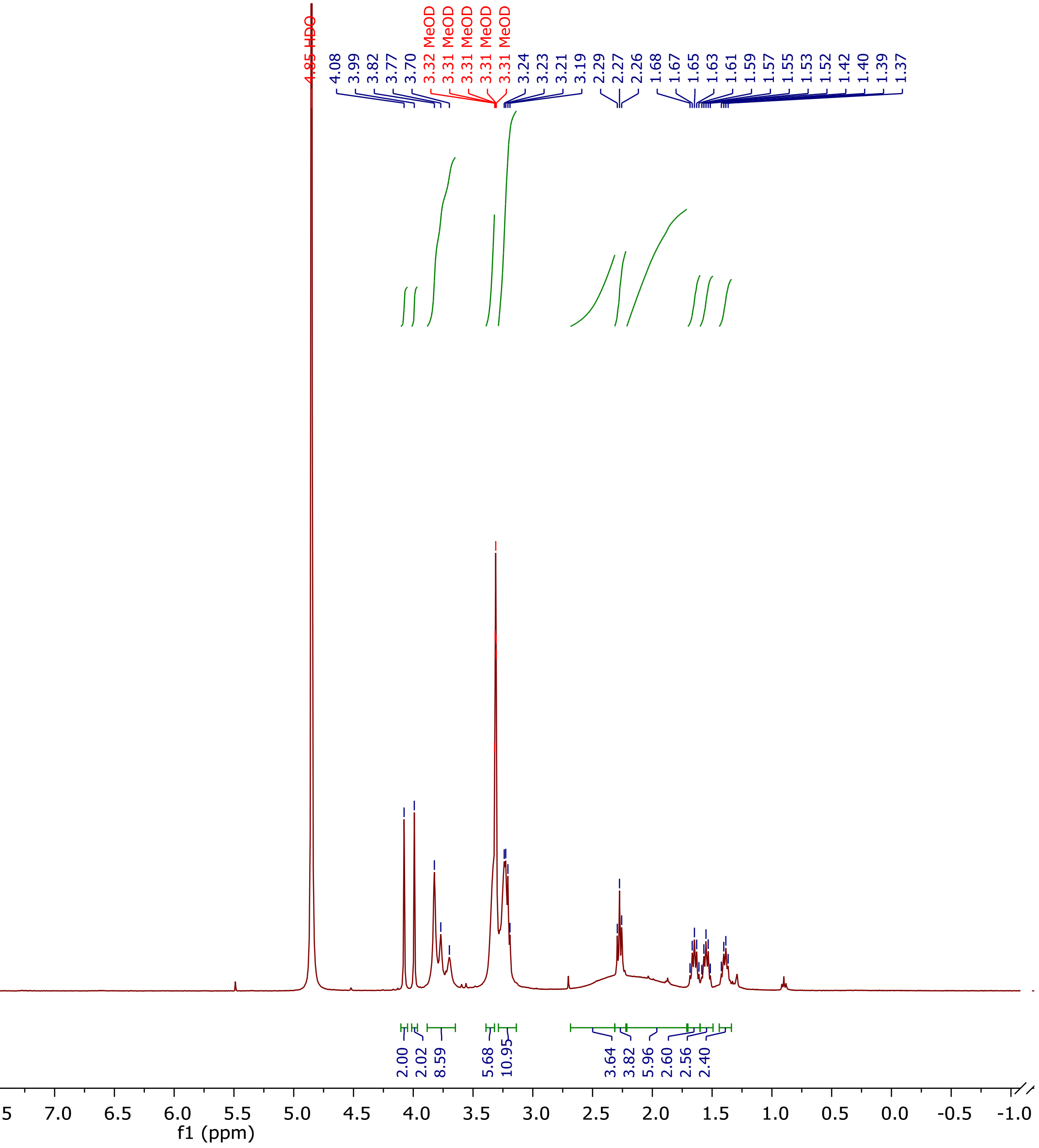

^{10}B-enriched-*C*-(6-(2-(4,7,10-tris-carboxymethyl-1,4,7,10-tetraazacyclododecan-1-yl)acetamido)hexanamido)methyl-*C'*-(sulfamoylamino)methyl-*o*-carborane (^{10}B-10).

^{1}H-COSY NMR (400 MHz, CD_3OD)

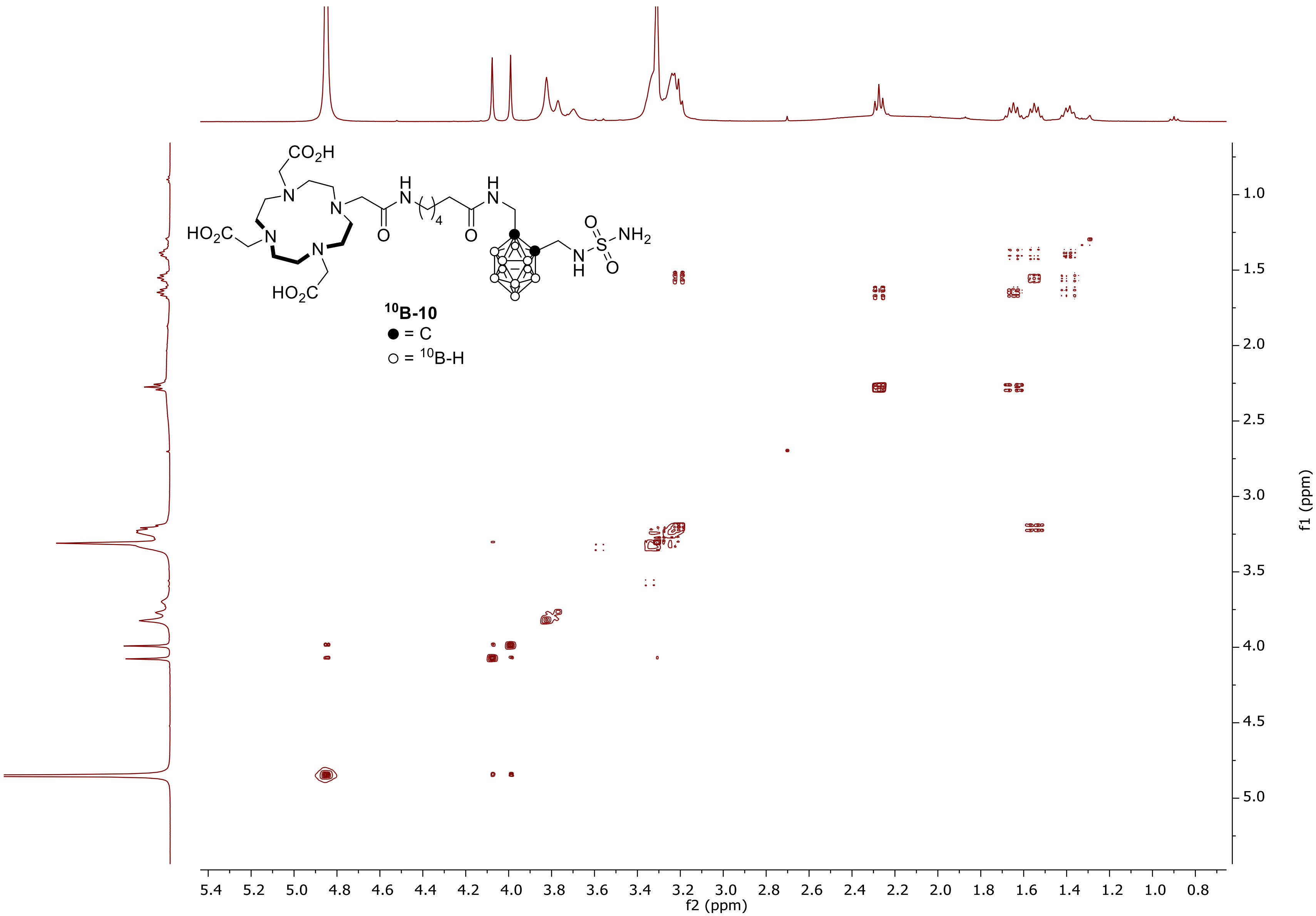

^{10}B-enriched-*C*-(6-(2-(4,7,10-tris-carboxymethyl-1,4,7,10-tetraazacyclododecan-1-yl)acetamido)hexanamido)methyl-*C'*-(sulfamoylamino)methyl-*o*-carborane (^{10}B-10).

^{13}C NMR (100 MHz, CD_3OD)

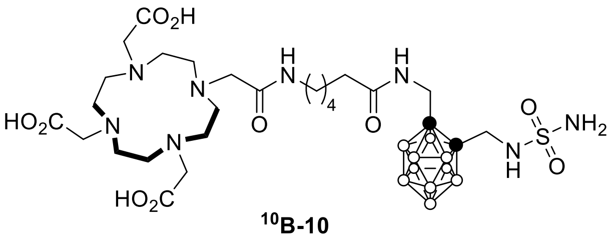

^{10}B-10
● = C
○ = ^{10}B-H

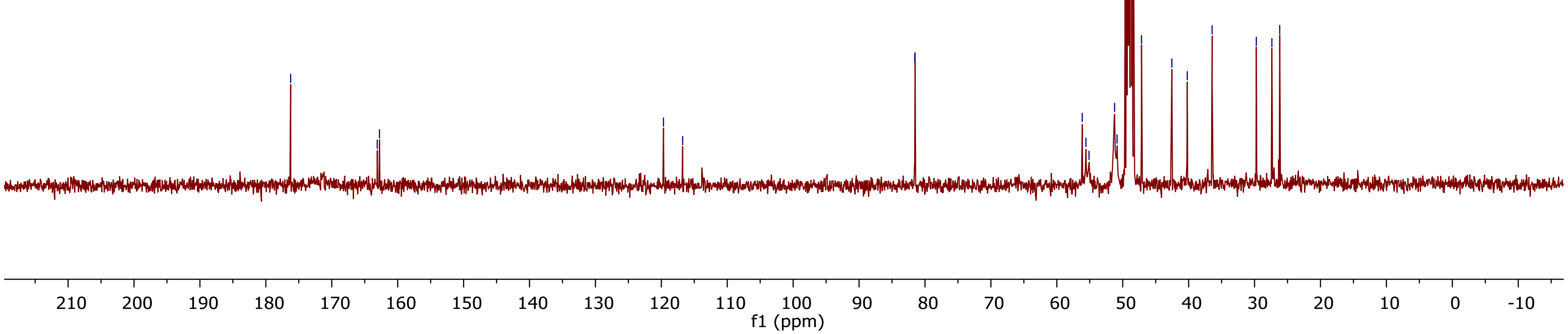

[10]B-enriched-*C*-(6-(2-(4,7,10-tris-carboxymethyl-1,4,7,10-tetraazacyclododecan-1-yl)acetamido)hexanamido)methyl-*C'*-(sulfamoylamino)methyl-*o*-carborane ([10]B-10).

Comparison between DEPT 135 and ^{13}C NMR (100 MHz, CD_3OD)

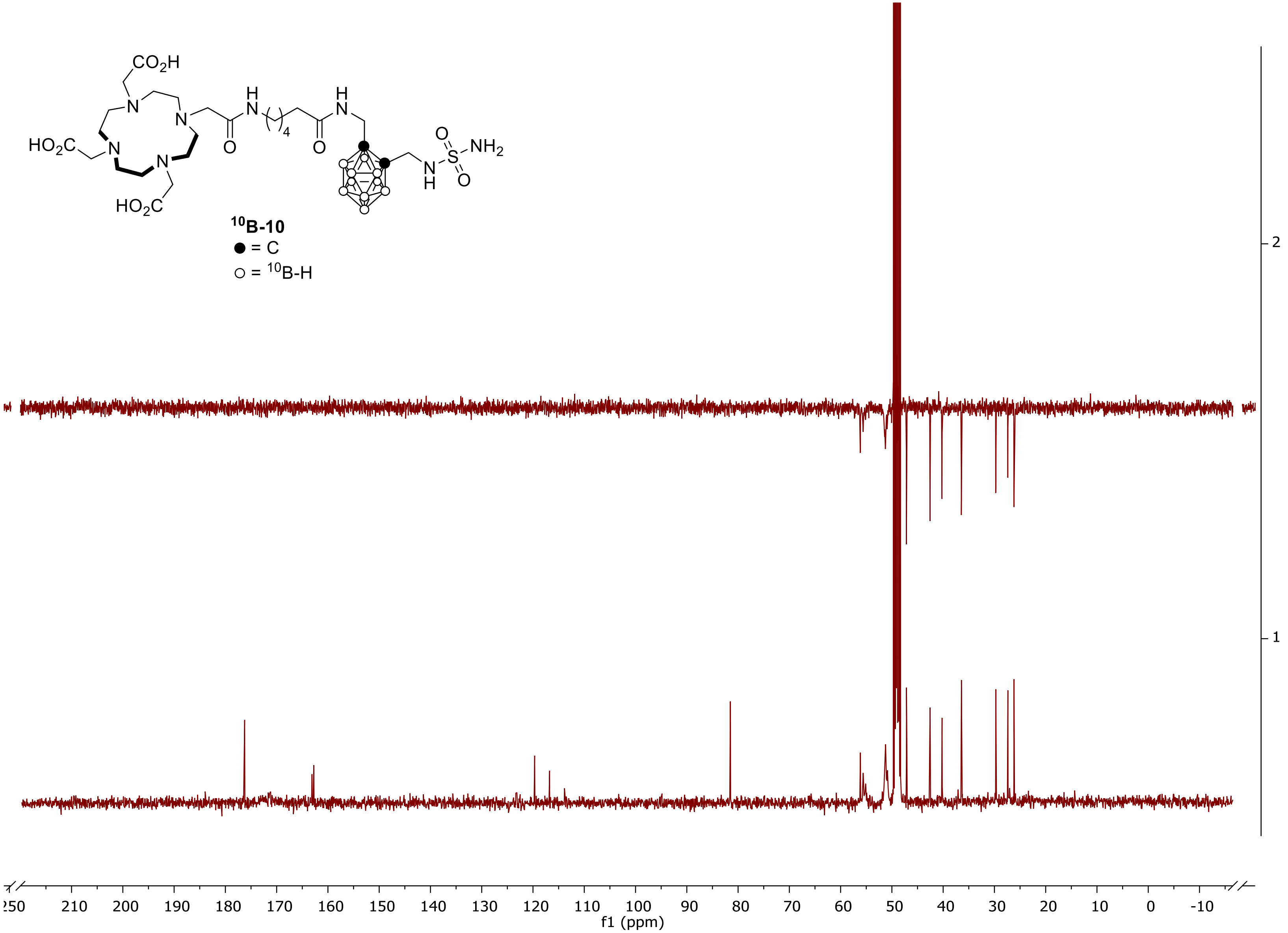

Supplement: Supplementary file 1 [file oc5c01632_si_001.pdf]
